# Synthesis and Microbiological Activities of 3-Nitropyrazolo-[1,5-d][1,2,4]triazin-7(6*H*)-ones and Derivatives †

**DOI:** 10.3390/molecules30183792

**Published:** 2025-09-18

**Authors:** Viktor A. Zapol’skii, Diana C. Munoz Castillo, Brigitte Pawletta, Ursula Bilitewski, Mimoza Gjikaj, Christoff Brüdigam, Dieter E. Kaufmann

**Affiliations:** 1 Institute of Organic Chemistry, Clausthal University of Technology, Leibnizstraße 6, 38678 Clausthal-Zellerfeld, Germany; viktor.zapolskii@tu-clausthal.de (V.A.Z.); christoff.bohlen@tu-clausthal.de (C.B.); 2 Helmholtz Centre for Infection Research (HZI), Inhoffenstraße 7, 38124 Braunschweig, Germany; dianacamila.munozcastillo@helmholtz-hzi.de (D.C.M.C.); brigitte.pawletta@helmholtz-hzi.de (B.P.); ursula.bilitewski@helmholtz-hzi.de (U.B.); 3 Institute of Inorganic and Analytical Chemistry, Clausthal University of Technology, Paul-Ernst-Straße 4, 38678 Clausthal-Zellerfeld, Germany; mimoza.gjikaj@tu-clausthal.de

**Keywords:** 2-nitroperchlorobutadiene, nitropyrazoles, pyrazolotriazinones, nucleophilic vinylic substitution, intramolecular cyclization, cellular activities

## Abstract

A new synthetic strategy for pyrazolo[1,5-*d*][1,2,4]triazin-7(6*H*)-ones **4** through intramolecular cyclization of alkyl 2-(4-nitro-1*H*-pyrazol-3-yl)methylene)hydrazine-1-carboxylates **3** is described, allowing us to selectively modify the *N*-substituent in 3-position. The reduction in nitro compounds **4** with tin(II) chloride leads to amines **5**, and their acetylation leads to acetamides **6**. Via alkylation of **4** with bromoacetic acid alkyl esters and 2-chloro-5-(chloromethyl)pyridine, and the subsequent reduction in alkylated nitro compounds **7,** the corresponding amines **8** and amides **9** were accessible in very good yields. The molecular structure of ethyl 2-(2-morpholino-3-nitro-7-oxopyrazolo[1,5-*d*][1,2,4]triazin-6(7*H*)-yl)acetate (**7b**) was confirmed by single-crystal X-Ray diffraction analysis. Antibacterial and cytotoxic properties were evaluated for 61 synthesized compounds.

## 1. Introduction

The readily accessible perchloro-2-nitro-1,3-butadiene (**1**) is one of the most attractive members of halogenated nitrobutadienes—a rather new and small group of unsaturated nitro compounds [1]. Studies have shown the enormous synthetic potential of diene **1** as a potent precursor for a variety of highly functionalized acyclic, as well as 4–7-cyclic *N*,*O*,*S*-compounds [2].

**Figure 1:**
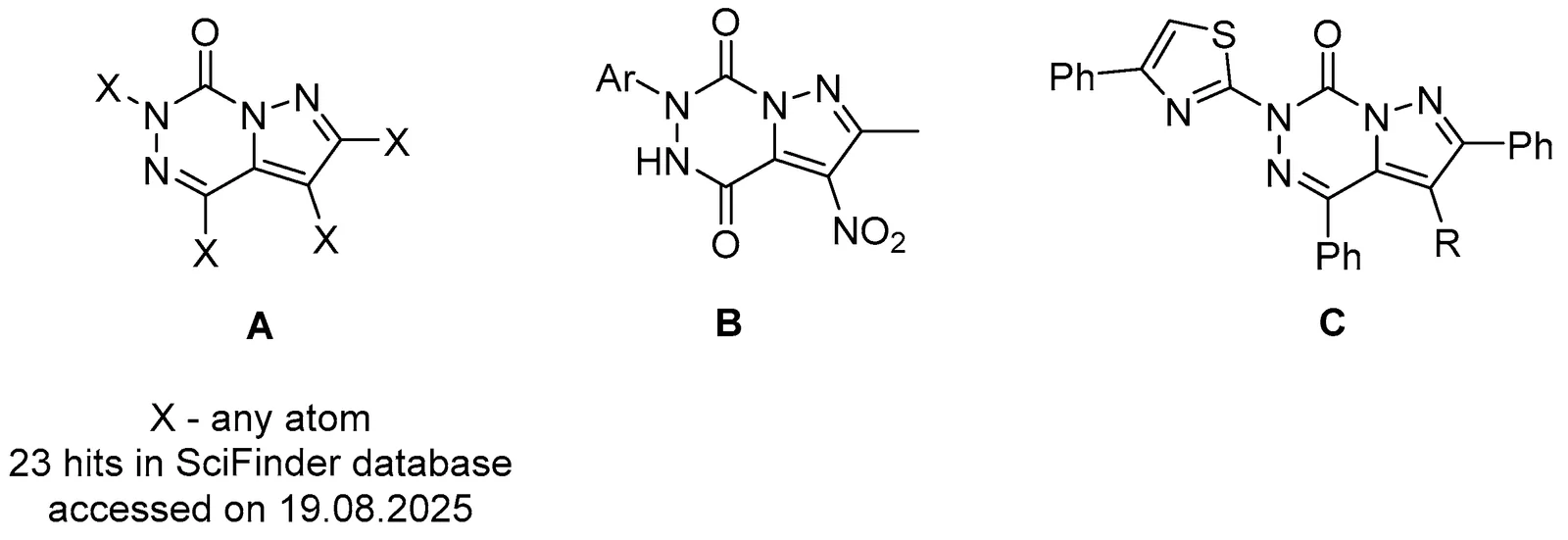
Score of compounds with a pyrazolo[1,5-*d*][1,2,4]triazin-7(6*H*)-one moiety and structures of some microbiologically active members.

Pyrazolo[1,5-*d*][1,2,4]triazin-7(6*H*)-ones **A** represent a very small class of organic heterocycles. An actual SciFinder [3] search on these compounds showed only 23 hits (Figure 1). For nine of these products, bioactivities were described. Pyrazolotriazinones such as **B** constitute a new class of human leukocyte elastase inhibitors [4], and bicyclic heterocycles such as **C** can be used as an activator of pro-apoptotic BAX [5]. The BAX gene was the first identified pro-apoptotic member of the Bcl-2 protein family [6].

**Scheme 1:**
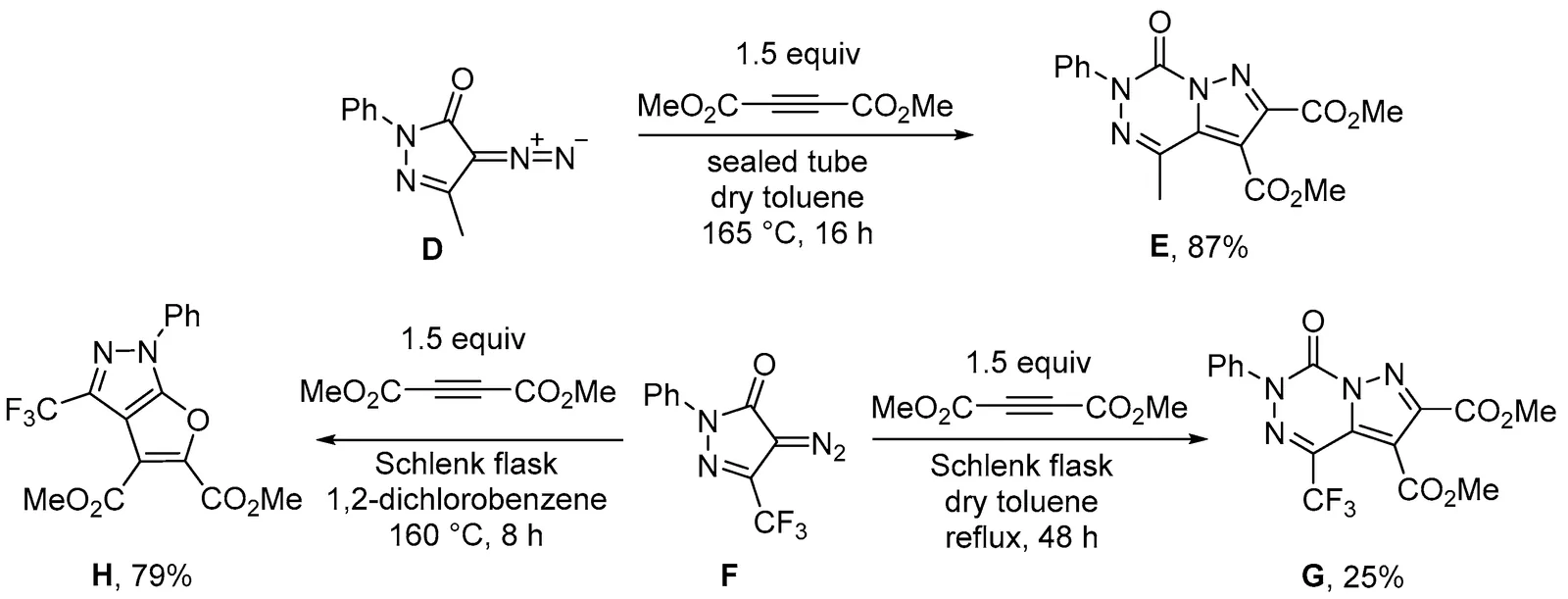
Reactions of diazopyrazolinones **D** and **F** with dimethyl acetylenedicarboxylate.

Until now, pyrazolo[1,5-*d*][1,2,4]triazin-7(6*H*)-one derivatives were synthesized, to the best of our knowledge, by only 1,3-dipolar cycloaddition reactions of diazopyrazolinones with electron-deficient dipolarophiles [7]. Padwa and coworkers showed that via the interaction of 3-methyl-4-diazo-1-phenylpyrazolin-5-one **D** with dimethyl acetylenedicarboxylate in toluene at 165 °C in a sealed tube, dimethyl 4-methyl-7-oxo-6-phenyl-6,7-dihydropyrazolo[1,5-*d*][1,2,4]triazine-2,3-dicarboxylate **E** was obtained in 87% yield. Using 3-trifluoromethyl-4-diazo-1-phenylpyrazolinone **F** as a starting material (refluxing toluene) led to the corresponding pyrazolotriazinone **G** in only 25% yield [8]. However, with 1,2-dichlorobenzene as a solvent, nitrogen was released, and another type of [3 + 2] cycloaddition resulted in a furo[2,3-*c*]pyrazole derivative **H** (Scheme 1).

In the present study, a more general, efficient three-step synthesis of pyrazolo[1,5-*d*][1,2,4]triazin-7(6*H*)-ones was developed, starting from nitrodiene **1**. Our main focus was (a) the recent progress in the synthesis of 3-nitropyrazolotriazinones **4**; (b) the subsequent reactions of **4**—reduction in the nitro group in position 3, acetylation of the newly formed amino group, and alkylation of the -NH-group in position 6 of the bicyclic pyrazolotriazinone; (c) the determination of cellular activities; and (d) the comparison of the microbiological activities of the synthetized pyrazolotriazinones with a nitro, an amino, and an acetamido group in 3-position.

## 2. Results and Discussion

### 2.1. Synthesis of the Pyrazolotriazinones

**Scheme 2:**
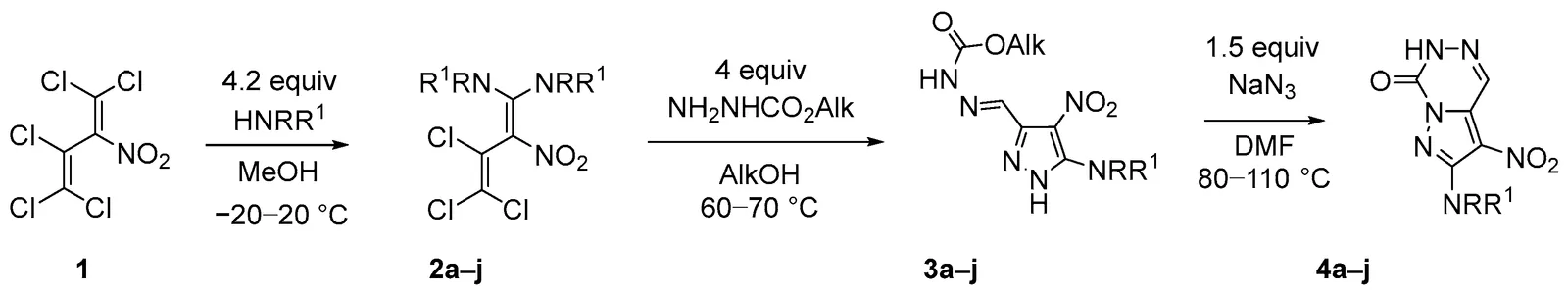
Synthesis of endiamines **2**, pyrazoles **3,** and pyrazolotriazinones **4**.

The butadienes **2** were obtained in yields of 55–95%, according to previous literature [9], via a vinylic S_N_ reaction of nitrodiene **1** with 4.2-fold excess of the corresponding amine in methanol at −20 °C within 3–8 h. The reaction of dienes **2** with a 4-fold excess of alkyl (Alk=Me or Et) hydrazine carboxylate in refluxing alcohol for 3–12 h led to the formation of nitropyrazoles **3** in 52–93% yield. The conceivable mechanism for the formation of the compounds **3** is presented in the literature [10]. Previously, we could show via X-Ray crystallographic analysis that through the reaction of *E*-(1-(benzotriazol-1-yl)-1-(2-methylphenylamino)-3,4,4-trichloro-2-nitrobuta-1,3-diene with phenylhydrazine (MeOH, rt, 2 d), the 4-nitro-1-phenyl-3-((2-phenylhydrazinylidene)-methyl)-5-(2-methylphenylamino)-1*H*-pyrazole was formed as a *Z*-isomer only. However, no single crystal of pyrazole **3** suitable for single-crystal X-Ray diffraction could be obtained. Upon heating pyrazoles **3** in DMF at 80–110 °C within 4–12 h using 1.5 equivalents of sodium azide as a nucleophile, the triazinones **4** were obtained in 42–95% yields (Scheme 2 and Table 1). When carrying out the reaction without the azide or using other nucleophiles, such as sodium alcoholates or triethylamine and/or other solvents, the formation of the triazinones **4** was either not observed or the yields were lower (5–30%). Interestingly, 2-((2-chlorophenyl)amino)-3-nitropyraz-olo[1,5-*d*][1,2,4]triazin-7(6*H*)-one (**4h**) was formerly identified as a Type I Interferons (species-specific glycoproteins) activity modulator compound [11].

**Scheme 3:**
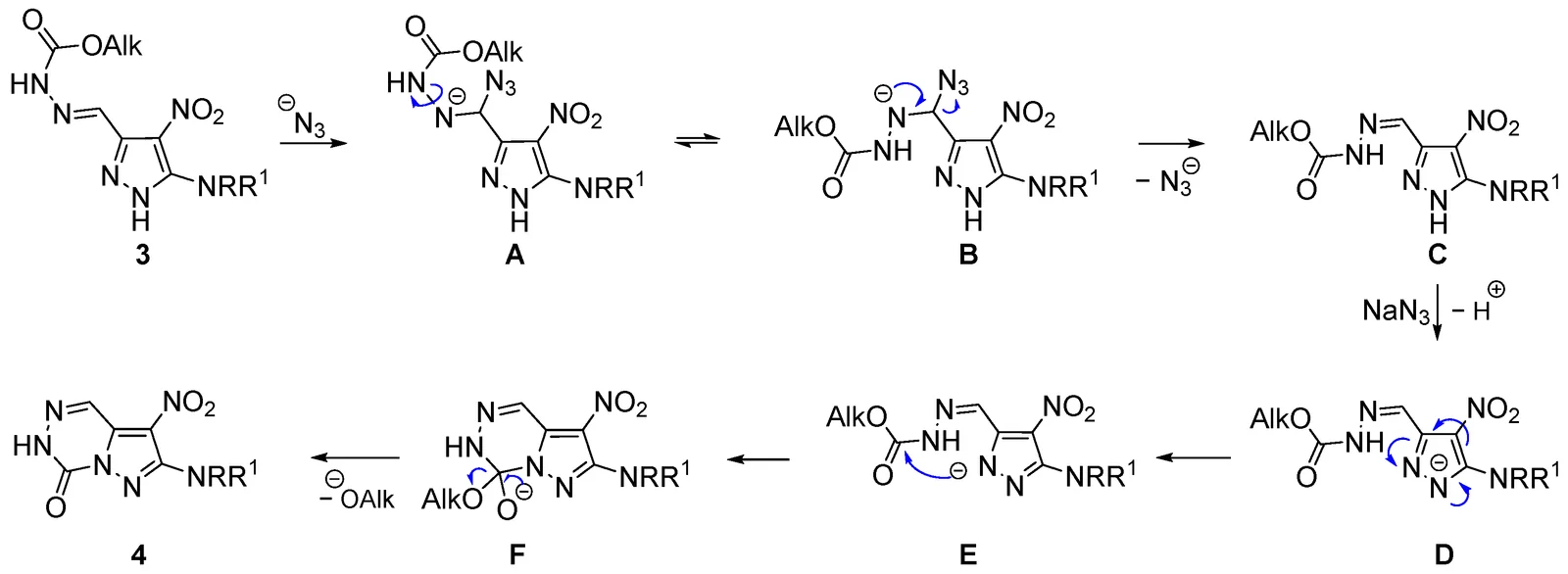
Plausible mechanism for the formation of pyrazolotriazinones **4**.

A conceivable mechanistic pathway for the reaction cascade to triazinones **4** is shown in Scheme 3. The azide anion is both a very good nucleophile and a rather weak base, with a pK_b_ of 9.24, comparable with aniline (9.37) and the acetate ion (9.24) [12]. Initially, upon addition of the azide anion to the double bond of the E-NH-N=CH-group in **3,** the intermediate **A** is formed. Due to the free rotation of the alkylcarbamate unit to **B** with subsequent elimination of the azide anion, the *Z*-pyrazole **C** is formed under isomerization. Further, deprotonation of N-1 in the pyrazole cycle (intermediate **D**) and a mesomeric shift to N-2 lead to the heterocycle **E**. Finally, pyrazolotriazinones **4** are obtained by intramolecular cyclisation to **F** and elimination of an alkoxide.

**Scheme 4:**
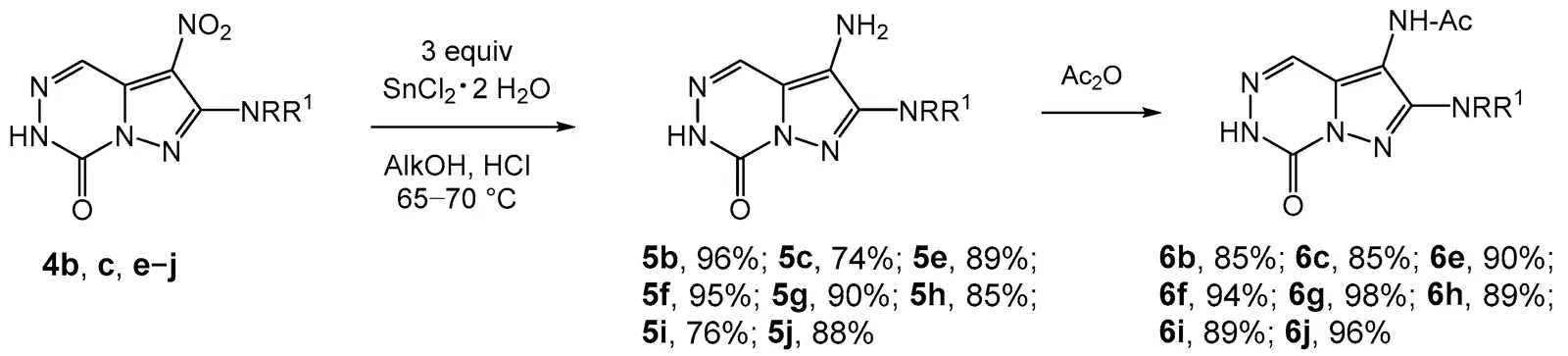
Synthesis of 3-aminopyrazolotriazinones **5** and acetamides **6**.

To change the electronic characteristic of the group in the 3 position from EW to ED, the nitro group was reduced in pyrazolotriazinones **4b**, **c**, and **e**–**j** with tin(II) chloride in methanol or ethanol at 40–80 °C in the presence of aqueous hydrochloric acid. Using three equivalents of SnCl_2_ 2H_2_O, within 4–18 h, the yields of the amines **5** reached 96%. Solventless acetylation of amines **5** with acetic anhydride ran smoothly at rt, already providing the acetamides **6** in very good yields (85–98%) (Scheme 4).

**Scheme 5:**
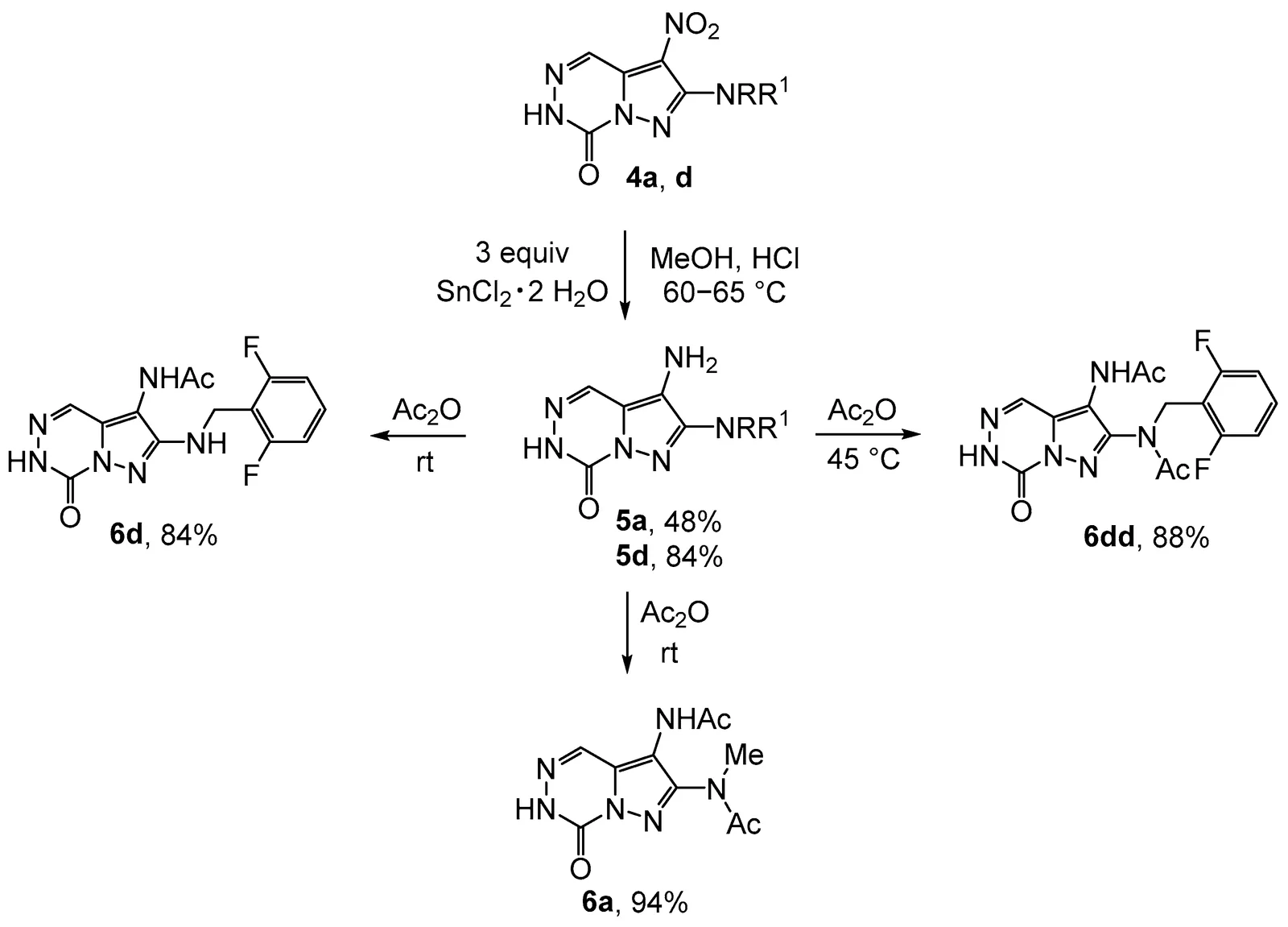
Synthesis of amines **5a** and **5d** and acetamides **6a**, **6d,** and **6dd**.

A reduction in the heterocycles **4a** and **4d** with tin(II) chloride in methanol at 60 °C led to the formation of the amines **5a** and **5d** in yields of 48% and 84%, respectively. The di(acetyl)amino product **6a** was obtained (94% yield) via acetylation of **5a** at rt. Treatment of the 2,6-difluorobenzylamino derivative **5d** with acetic anhydride at rt led to (monoacetyl)pyrazolotriazinones **6d** in 84% yield, and after increasing the temperature up to 45 °C, the corresponding di(acetyl)amino product **6dd** was isolated in 88% yield (Scheme 5).

**Scheme 6:**
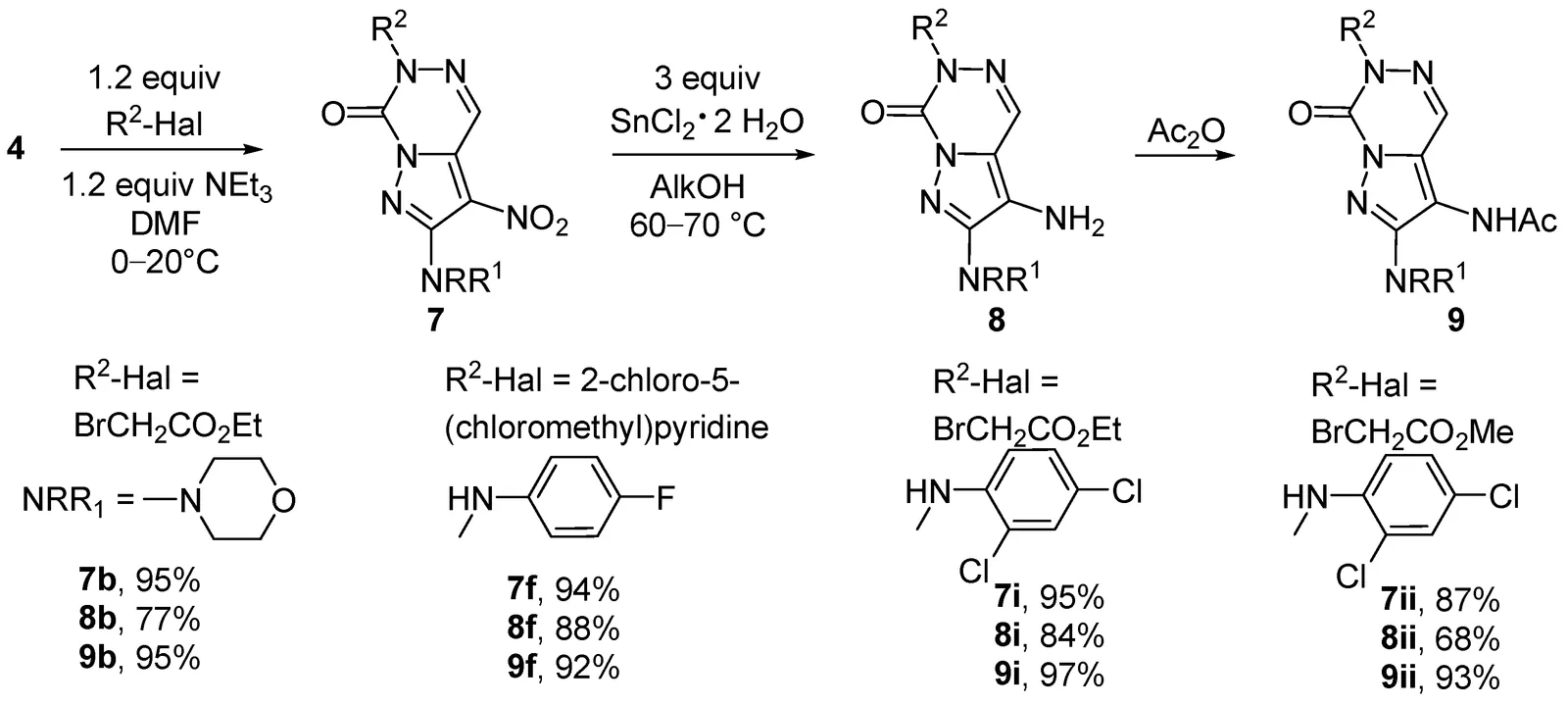
Alkylation of nitrotriazinones **4b**, **f**, **i,** and **7,** and synthesis of the corresponding amines **8** and acetamides **9**.

To expand the compound library of pyrazolotriazinones **4** for biological screening, the heterocycles **4** were also alkylated with bromoacetic acid methyl and ethyl ester and with 2-chloro-5-(chloromethyl)pyridine in the *N*-6-position. Under optimized reaction conditions in DMF, the *N*-alkylated nitrotriazinones **7b**, **f**, **i**, and **ii** were obtained in 87–95% yields. A reduction in the nitro group in compounds **7** with tin(II) chloride dihydrate led to the amino derivatives **8b**, **f**, **i**, and **ii** in good yields (68–88%). Acetylation of the amines **8** with acetic anhydride at rt (45 °C in case of **9f**) gave the corresponding acetamides **9b**, **f**, **i**, and **ii**, isolated in 92–97% yields (Scheme 6).

**Figure 2:**
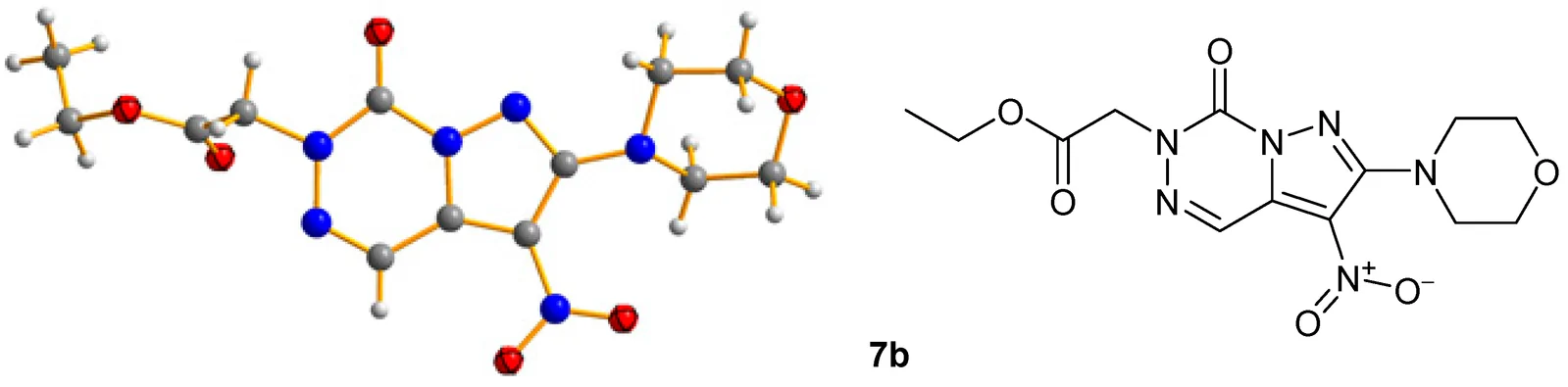
Single-crystal X-Ray diffraction structure of ethyl 2-(2-morpholino-3-nitro-7-oxopyrazolo[1,5-*d*][1,2,4]triazin-6(7*H*)-yl)acetate (**7b**).

The structure of pyrazolotriazinone **7b** was confirmed by single-crystal X-Ray diffraction analysis (Figure 2).

All synthesized compounds **3**–**9** have a characteristic imino group, CH=N, which is isoelectronic to the oxo group and present in many nucleobases. It was demonstrated [13] that, through its interaction with a substrate in precatalytic and transition-state conformations, an imino group is indispensable for cleavage by a small ribozyme. Table 2 shows the ^1^H- and ^13^C-shifts and direct ^1^*J*^13^_C-H_ satellites coupling constants of the CH=N moiety of compounds **3**–**9**. The ^1^H-shifts are mainly in the range of 8.2 to 8.8 ppm, with a relatively large, expected difference between nitro (**4**/**7**, 8.7–8.8 ppm) and amino/amido (**5**–**9**, 8.2–8.3 ppm) derivatives. The ^13^C-shifts of imino groups are in the narrow range of 130–131 ppm, with the exception of the nitro compounds **4**/**7** (about 128–129 ppm). The ^1^*J*^13^_C_-_H_-coupling constants show large diversity between pyrazole (about 176 Hz) and pyrazolotriazinone (between 191 and 204 Hz) cycles, which can be explained by the structural differences in both heterocycles. Within pyrazolotriazinones, nitro compounds **4**/**7** show the highest coupling constants (203–204 Hz), followed by amides **6**/**9** (197–198 Hz) and amines **5**/**8** (191–192 Hz).

### 2.2. Evaluation of the Microbiological Activities

The microbiological activity of representative members of the different groups of compounds was evaluated, including the butadienes **2** as a starting material and the pyrazoles **3** as intermediates. All compounds were applied to growth inhibition assays using the Gram-negative bacteria *Escherichia coli* (ATCC 25922), (LGC, Teddington, Middlesex, TW11 0LY, UK) and *Klebsiella pneumonia* (DSM681) (DSMZ, Braunschweig, Germany) and the Gram-positive bacteria *Staphylococcus aureus*, particularly the methicillin-resistant strain USA300 (MRSA) (kindly provided by E. Medina, INI, HZI Braunschweig). Gram-negative bacteria frequently possess efficient efflux pumps, which export the compounds from the cytosol so that the intracellular compound concentration is so low that growth of the bacteria is not affected. That is why we used the *E. coli* delta TolC-mutant in addition to the wild-type strains, in which the outer channel protein TolC is inactivated so that the efflux pump system AcrA/AcrB/TolC is not functional and intracellular compound concentrations are likely increased.

In addition to the evaluation of the antibiotic properties of compounds, the cytotoxic potential of the compounds was studied using the murine fibroblast cell line L929.

**Figure 3:**
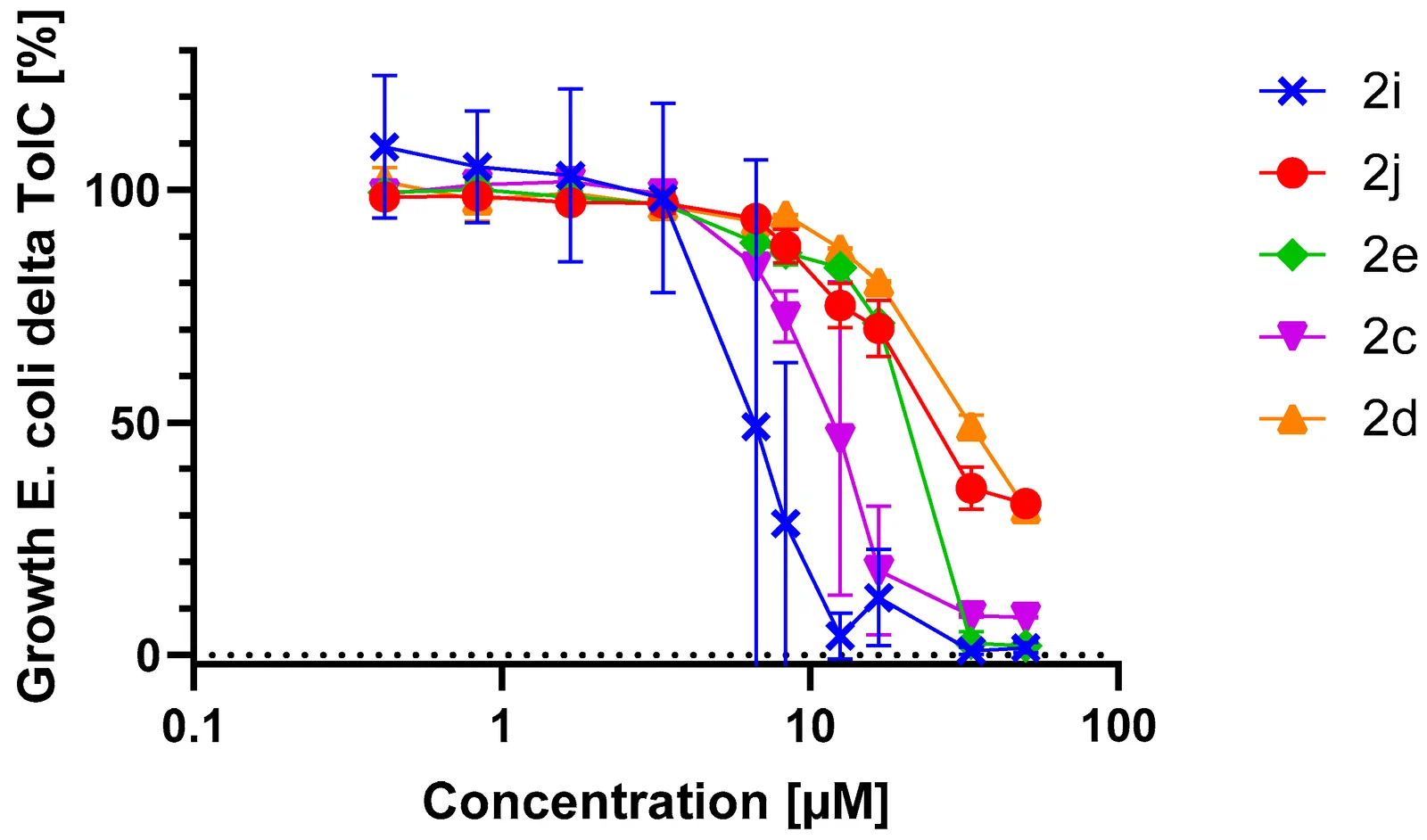
Dose–response curves showing the effects of compound concentrations on growth of the *E. coli* delta TolC mutant. The dotted line indicates the “0”-level.

**Figure 4:**
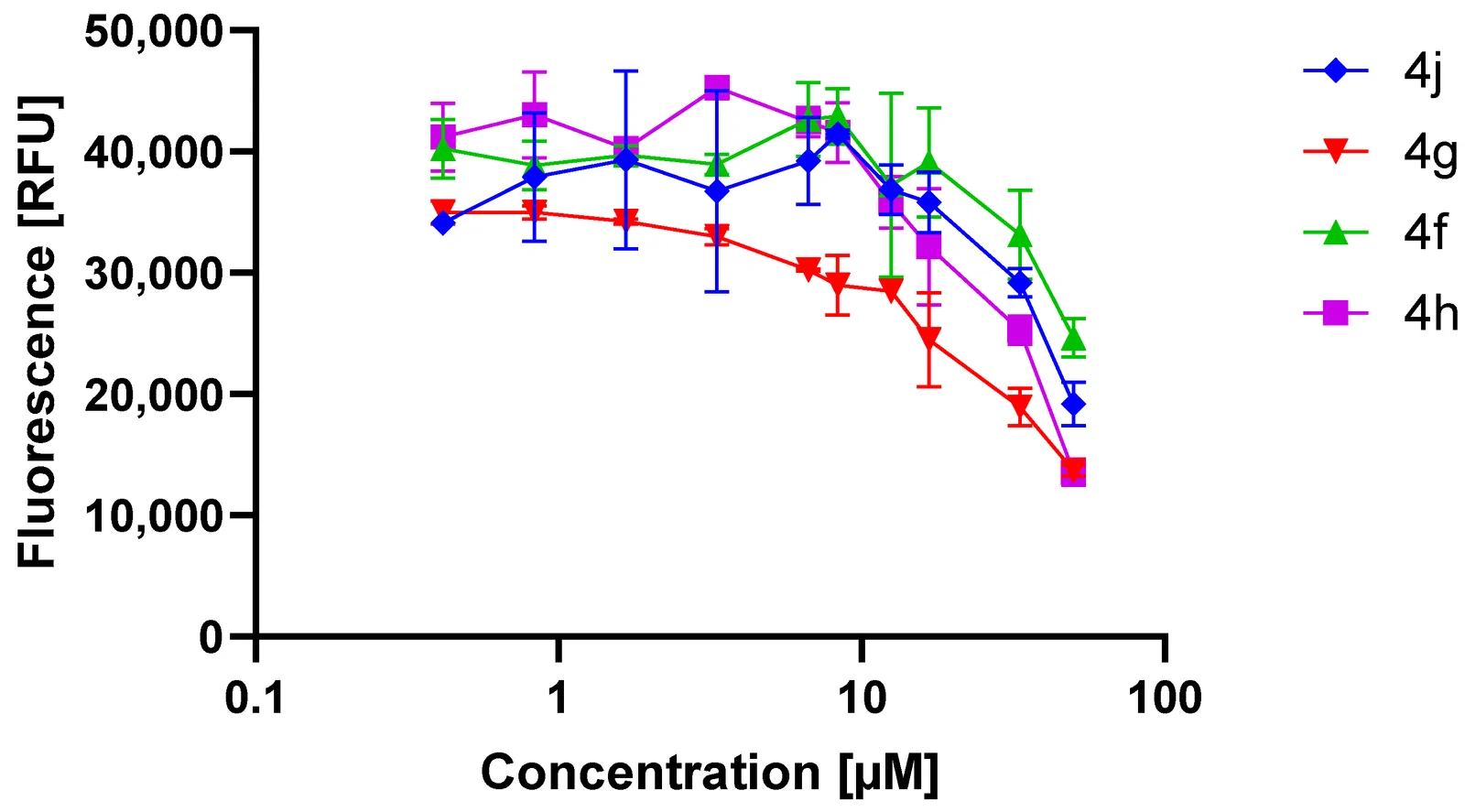
Influence of the concentrations of selected nitrotriazinones on the viability of the murine fibroblast cell line L929, indicating the cytotoxicity of the compounds. The viability of the cells was determined using the alamarBlue^TM^ assay, which is based on the reduction of blue, non-fluorescent resazurin to red, highly fluorescent resorufin by viable cells.

All assays were performed in transparent 96-well half-area microtiter plates (Costar 3697) (Corning GmbH, Kaiserslautern, Germany) with assay volumes of 60 µL. The cytotoxicity of compounds was determined using the alamarBlue^TM^ (Resazurin: Sigma-Aldrich via Merck GmbH, Darmstadt, Germany) assay for the assessment of cell viability (determination of fluorescence Ex: 540 nm; Em: 600 nm), whereas the growth of bacteria was determined via the turbidity of the bacterial suspension at 600 nm. Compounds were dissolved in DMSO (Sigma-Aldrich via Merck GmbH, Darmstadt, Germany), resulting in 10 mM stock solutions. To rapidly identify active compounds, each compound was used in a single screening concentration of 50 µM, and all compounds were combined on one microtiter plate. Three plates were used per organism to result in triplicate measurements. The screening concentrations resulted from the stock solutions in a two-step dilution protocol, with 2 mM solutions in buffer +20% DMSO as intermediate solutions. In the second step, dose–response curves were generated for the active compounds, which were identified by the screening format. The respective compound plates were generated by the acoustic dispenser Echo 550 (Beckman Coulter) (Beckman Coulter as part of Danaher Corp. Brea, USA). From the resulting curves, the IC_50_-values (concentration leading to 50% inhibition) were calculated using non-linear regression algorithms of the software GraphPad Prism (Version 10.5.0). All compounds showed no antibiotic activity against the Gram-negative bacteria *Escherichia coli* and *Klebsiella pneumoniae.* As in other studies, the activity profiles for the *Escherichia coli* delta TolC mutant and the Gram-positive bacteria *Staphylococcus aureus* were very similar and resembled the cytotoxic profile. Surprisingly, most of the compounds did not show any biological activity. This included the intermediate pyrazoles **3**, the aminated pyrazolotriazinones **5**, **8**, and the derived acetylated amines **6**, **9**. Only representatives of the parent butadienes **2** and the nitro derivatives of the pyrazolotriazones **4** and **7** showed some biological activity. Generally, the IC_50_-values obtained for the three organisms (*S. aureus, E. coli* delta TolC (Figure 3), and the cell line L929 (Figure 4)) were in the same order of magnitude.

The exceptions were the compounds **7b, 4h,** and **4f,** which were only cytotoxic (IC_50_ = 11 µM, 32.2 µM, and 36.5 µM), but did not show any antibacterial activity. For the butadienes **2,** IC_50_-values ranged from IC_50_ < 10 µM (**2c**, **2i**) to IC_50_s between 10 and 20 µM (**2e**, **2j**) (Figure 3) and to even higher values of IC_50_ > 30 µM (**2d**), at least in the antibacterial assays. The nitrotriazinones **4g** and **4j** were less active than the butadienes, as IC_50_-values were between approximately 20 and 40 µM (Figure 4).

## 3. Materials and Methods

### 3.1. Chemistry

#### 3.1.1. General Remarks

The solvents and reagents were used as-received from commercial sources without further purification. TLC was performed with Merck aluminum-backed TLC plates using silica gel 60, F_254_. Flash column chromatography was performed using Macherey–Nagel silica gel 60 M (0.040–0.063 mm) with appropriate mixtures of petroleum ether (PE, boiling range 60–70 °C) and ethyl acetate (EA) as the eluents. The melting points (mp) were determined in capillary tubes with a Büchi B-520. The FTIR spectra were recorded with a Bruker “Alpha-T” spectrometer (Bruker, Ettlingen, Germany) with the solid compounds measured as KBr pellets. The ATR-IR spectra were measured on the same instrument with a Bruker “Alpha Platinum ATR” single-reflection diamond ATR module. The ^1^H-NMR and ^13^C-NMR spectra at 600 and 150 MHz, respectively, were recorded with an “Avance III” 600 MHz FTNMR spectrometer (Bruker, Rheinstetten, Germany). The ^1^H-NMR and ^13^C-NMR spectra at 400 and 100 MHz, respectively, were recorded with an “Avance 400” MHz FT-NMR spectrometer (also Bruker). The ^1^H and ^13^C-NMR spectra were examined with reference to the residual solvent peak: CDCl_3_ at δ 7.26 (^1^H) and 77.0 ppm (^13^C), and DMSO-*d_6_* at δ = 2.50 (^1^H) and 39.7 ppm (^13^C). The-NMR spectra and HR-MS data of the newly synthesized compounds are available in the Supplementary Materials. The mass spectra were obtained with a Hewlett–Packard MS 5989B spectrometer (HP Inc., Palmer, MA, USA), usually in direct mode with the electron impact (70 eV). For the chlorinated compounds, all peak values of molecular ions and fragments refer to the isotope ^35^Cl. High-resolution mass spectra were recorded with the “Impact II” from BRUKER (Bruker Daltonik GmbH, Bremen, Germany).

#### 3.1.2. Synthesis Protocols

*2-Nitro-pentachloro-1,3-butadiene* (**1**). The product was prepared from 2*H*–pentachloro-1,3-butadiene at a 53% yield (bp 69–71 °C/1 mbar), according to the literature [14].

*1,1-Bis(methylamino)-3,4,4-trichloro-2-nitrobuta-1,3-diene* (**2a**). A 33% methylamine (3.92 g, 42 mmol) solution (soln.) in ethanol (EtOH) was added dropwise to a soln. of nitrodiene **1** (2.71 g, 10 mmol) in 30 mL methanol (MeOH) at −20 °C within 15 min. The resulting mixture was kept at the same temperature for an additional 1 h and 2 h at room temperature (rt) with stirring. Subsequently, the supernatant liquid was concentrated in vacuo to a volume of 10 mL and cooled to 10 °C. The resulting precipitate was filtered off, washed with water (2 × 10 mL), and then dried in vacuo to give **2a** as a colorless solid; yield: 1.44 g (5.50 mmol, 55%) mp 130–131 °C. IR (KBr): *ṽ*_max_ = 3218, 1630, 1573, 1409, 1318, 1138, 1039, 1012, 950, 840, 793, 698, 5940 cm^−1^. ^1^H-NMR (CDCl_3_, 400 MHz): *δ* = 3.11 (d, ^1^*J*_C-H_ = 139.8 Hz, 3H), 3.09 (d, ^1^*J*_C-H_ = 139.6 Hz, 3H) ppm, NH groups could not be detected. ^1^H-NMR (DMSO-*d_6_*, 400 MHz): *δ* = 8.33 (br. s, 2H, NH), 2.83 (d, *J* = 5.5 Hz, 6H) ppm. ^13^C-NMR (DMSO-*d_6_*, 101 MHz): *δ* = 158.9 (NCN), 127.2 and 113.2 (CCl_2_ = CCl), 121.4 (C-NO_2_), 30.5 (2 CH_3_) ppm. In DMSO-*d_6_* compound **2a** slowly hydrolyzes to 1,1-bis(methylamino)-4,4-dichloro-2-nitrobut-1-en-3-one: ^1^H-NMR (400 MHz): *δ* = 9.46 (q, *J* = 5.5 Hz, 1H, NH), 8.91 (q, *J* = 5.5 Hz, 1H, NH), 7.53 (d, ^1^*J*_C-H_ = 185.1 Hz, 1H, CHCl_2_), 2.72 (d, *J* = 5.5 Hz, 6H) ppm. ^13^C-NMR (101 MHz): *δ* = 174.6 (C=O), 160.3 (NCN), 104.4 (C-NO_2_), 69.5 (CHCl_2_), 29.9 (CH_3_), 29.2 (CH_3_) ppm. MS: *m*/*z* (%) = 259 (6) [M^+^] 224 (12) [M—Cl]^+^, 177 (12) [M—Cl—HNO_2_]^+^, 114 (100) [M—C_2_Cl_3_—CH_4_]^+^. HRMS-ESI: *m*/*z* was calc. for C6H8Cl3N3O2Na [M + Na]^+^ 281.9574 and found 281.9569.

*1,1-Bis(morpholino)-3,4,4-trichloro-2-nitrobuta-1,3-diene* (**2b**). A soln. of morpholine (3.66 g, 42 mmol) in 10 mL MeOH was added dropwise to a soln. of nitrodiene **1** (2.71 g, 10 mmol) in 30 mL MeOH at −20 °C within 15 min. The resulting mixture was kept at the same temperature for an additional 1 h and 2 h at rt with stirring. Subsequently, the resulting precipitate was filtered off, washed with water (2 × 10 mL) and MeOH (2 × 10 mL), and then dried in vacuo to give **2b** as a yellowish solid; yield: 3.20 g (8.60 mmol, 86%); mp 251–252 °C. IR (KBr): *ṽ*_max_ = 2909, 2862, 1536, 1497, 1383, 1290, 1108, 1030, 876, 800, 716, 575 cm^−1^. ^1^H-NMR (CDCl_3_, 400 MHz): *δ* = 4.09–3.64 (m, 6H), 3.64–3.49 (m, 4H), 3.47–3.33 (m, 5H), 3.32–3.21 (m, 1H) ppm. ^1^H-NMR (DMSO-*d*_6_, 400 MHz): *δ* = 3.88–3.13 (m, 16H) ppm. All spectral data and the mp are in accordance with the literature [15].

*1,1-Bis(2-(4-chlorophenoxy)ethylamino)-3,4,4-trichloro-2-nitrobuta-1,3-diene* (**2c**). A soln. of 2-(4-chlorophenoxy)ethan-1-amine (7.21 g, 42 mmol) in 20 mL MeOH was added dropwise to a soln. of nitrodiene **1** (2.71 g, 10 mmol) in 30 mL MeOH at −20 °C within 15 min. The resulting mixture was kept at the same temperature for an additional 1 h and 2 h at rt with stirring. Subsequently, the supernatant liquid was concentrated in vacuo to a volume of 20 mL, cooled to 10 °C, and diluted with 100 mL cold water. The resulting precipitate was filtered off, washed with water (3 × 10 mL) and diethyl ether (2 × 10 mL), and then dried in vacuo to give **2c** as a colorless solid, yield: 4.77 g (8.80 mmol, 88%) mp 118–120 °C. IR (ATR): *ṽ*_max_ = 002993, 2930, 2870, 1632, 1489, 1338, 1234, 1048, 819, 716, 611, 505 cm^−1^. ^1^H-NMR (CDCl_3_, 400 MHz): *δ* = 8.43 (br.s, 2H, 2NH), 7.24 (d, *J* = 8.9 Hz, 4CH), 6.82 (d, *J* = 8.9 Hδz, 4CH), 4.14 (t, *J* = 4.8 Hz, 4H, 2OCH_2_) 3.72 (q, *J* = 5.4 Hz, 4H, 2NCH_2_) ppm. ^13^C-NMR (CDCl_3_, 101 MHz): *δ* = 159.2 (NCN), 156.2 (Cq-O), 129.7 (4CH), 127.1 (2C_Ar_-Cl), 125.5 and 125.1 (CCl_2_ = CCl), 115.9 (4CH), 107.4 (C-NO_2_), 67.9 (2OCH_2_), 43.9 (2NCH_2_) ppm. MS: *m*/*z* (%) = 541 (1) [M^+^], 422 (12) [M—Cl—HCl—NO_2_]^+^, 352 (4) [M—ArOCH_2_—NO_2_]^+^, 128 (100) [*p*-Cl-C_6_H_4_-OH]^+^. HRMS (ESI): *m*/*z* was calc. for C_20_H_18_Cl_5_N_3_O_4_Na [M + Na]^+^ 563.9603 and found 563.9608.

*1,1-Bis(2,6-difluorobenzylamino)-3,4,4-trichloro-2-nitrobuta-1,3-diene* (**2d**). The product was prepared according to diene **2c** from nitrodiene **1** (2.71 g, 10 mmol) and (2,6-difluorophenyl)methanamine (6.01 g, 42 mmol): colorless solid, yield 4.56 g (9.40 mmol, 94%) mp 130–131 °C. IR (ATR): *ṽ*_max_ = 3340, 1593, 1538, 1469, 1336, 1136, 1013, 783, 608, 497, 467, 411 cm^−1^. ^1^H-NMR (CDCl_3_, 400 MHz): *δ* = 7.52 (br.s, 2H, 2NH), 7.35 (tt, *J* = 6.8 (C-F), 8.1 (C-H) Hz, 2CH), 6.95 (t, *J* = 8.1 Hz, 4CH), 4.59 (d, *J* = 5.6 Hz, 4H, 2NCH_2_) ppm. ^13^C-NMR (CDCl_3_, 101 MHz): *δ* = 161.2 (d, ^1^*J*_C-F_ = 249.8 Hz, 2Cq-F), 161.1 (d, ^1^*J*_C-F_ = 249.9 Hz, 2Cq-F), 158.0 (NCN), 131.0 (t, ^3^*J*_C-F_ = 10.5 Hz, 2CH), 127.2 and 123.8 (CCl_2_=CCl), 111.9 (t, ^2^*J*_C-F_ = 17.4 Hz, 2Cq), 111.8 (dd, ^2^*J*_C-F_ = 19.3 Hz, ^4^*J*_C-F_ = 6.2 Hz, 4CH), 108.7 (C-NO_2_), 37.0 (2NCH_2_) ppm. MS: *m*/*z* (%) = 483 (1) [M^+^], 448 (1) [M—Cl]^+^, 413 (3) [M—2 Cl]^+^, 127 (100) [CH_2_C_6_H_4_F_2_^+^].

*1,1-Bis(phenylamino)-3,4,4-trichloro-2-nitrobuta-1,3-diene* (**2e**). The product was prepared according to diene **2c** from nitrodiene **1** (2.71 g, 10 mmol) and aniline (3.91 g, 42 mmol): pale solid, yield 3.39 g (8.80 mmol, 88%), mp 165–166 °C. IR (ATR): *ṽ*_max_ = 3032, 1630, 1580, 1496, 1380, 1327, 1125, 856, 804, 689, 588 cm^−1^. ^1^H-NMR (DMSO-*d*_6_, 400 MHz): *δ* = 10.25 (br.s, 2H, 2NH), 7.27–7.07 (m, 10CH) ppm. ^13^C-NMR (DMSO-*d*_6_, 101 MHz): *δ* = 152.2 (NCN), 138.5 (2Cq-NH), 129.2 (4CH), 126.2 and 122.5 (CCl_2_ = CCl), 115.2 (2CH), 121.9 (4CH), 109.8 (C-NO_2_) ppm. All spectral data are in accordance with the literature [16].

*1,1-Bis(4-fluorophenylamino)-3,4,4-trichloro-2-nitrobuta-1,3-diene* (**2f**) was prepared according to the literature [9]. All spectral data are in accordance with the literature [9].

*1,1-Bis(4-chlorophenylamino)-3,4,4-trichloro-2-nitrobuta-1,3-diene* (**2g**) was prepared according to the literature [9]. All spectral data are in accordance with the literature [9].

*1,1-Bis(2-chlorophenylamino)-3,4,4-trichloro-2-nitrobuta-1,3-diene* (**2h**) was prepared according to the literature [9]. All spectral data are in accordance with the literature [17].

*1,1-Bis(2,4-dichlorophenylamino)-3,4,4-trichloro-2-nitrobuta-1,3-diene* (**2i**). The product was prepared according to diene **2b** from nitrodiene **1** (2.71 g, 10 mmol) and 2,4-dichloroaniline (6.81 g, 42 mmol): light brown solid, yield 4.56 g (8.60 mmol, 86%), mp 156–157 °C. IR (KBr): *ṽ*_max_ = 3087, 1614, 1571, 1476, 1282, 1127, 1101, 1058, 868, 792, 690, 600 cm^−1^. ^1^H-NMR (DMSO-*d*_6_, 400 MHz): *δ* = 10.31 (br.s, 2H, 2NH), 7.62 (d, *J* = 2.1 Hz, 2CH), 7.36 (dd, *J* = 8.6, 2.1 Hz, 2CH), 7.27 (d, *J* = 8.6 Hz, 2CH) ppm. ^13^C-NMR (DMSO-*d*_6_, 101 MHz): *δ* = 154.8 (NCN), 133.7 (2Cq-NH), 132.1 (2Cq-Cl), 131.1 (2Cq-Cl), 129.5 (2CH), 129.2 (2CH), 128.1 (2CH), 125.1 and 125.0 (CCl_2_ = CCl), 108.9 (C-NO_2_) ppm. MS: *m*/*z* (%) = 521 (5) [M^+^], 484 (12) [M—Cl]^+^, 475 (12) [M—NO_2_]^+^, 161 (100) [2,4-dichloroaniline—H]^+^.

*1,1-Bis(2-methyl-3-trifluoromethylphenylamino)-3,4,4-trichloro-2-nitrobuta-1,3-diene* (**2j**). A soln. of 2-methyl-3-trifluoromethylaniline (7.36 g, 42 mmol) in 20 mL MeOH was added dropwise to a soln. of nitrodiene **1** (2.71 g, 10 mmol) in 30 mL MeOH at −20 °C within 15 min. The resulting mixture was kept at the same temperature for an additional 1 h and 1 d at rt with stirring. Subsequently, the supernatant liquid was concentrated in vacuo to a volume of 20 mL, cooled to 10 °C, and diluted with 100 mL hydrochloric acid (5% in water). The resulting precipitate was filtered off, washed with water (3 × 10 mL) and PE (2 × 10 mL), and then dried in vacuo to give **2j** as a colorless solid; yield: 4.66 g (8.50 mmol, 85%) mp 155–156 °C. IR (ATR): *ṽ*_max_ = 3234, 1605, 1581, 1518, 1313, 1233, 1171, 1111, 1063, 1014, 803, 614, 522, 477 cm^−1^. ^1^H-NMR (CDCl_3_, 400 MHz): *δ* = 11.86 (br.s, 1H, NH), 7.40 (d, *J* = 7.6 Hz, 2CH), 7.14 (d, *J* = 7.8 Hz, 2CH), 7.09 (dd, *J* = 7.8, 7.6 Hz, 2CH), 6.19 (br.s, 1H, NH), 2.33 (s, 6H, 2CH_3_) ppm. ^13^C-NMR (CDCl_3_, 101 MHz): *δ* = 154.7 (NCN), 136.0 (2Cq), 133.1 (2Cq), 130.6 (q, ^2^*J*_C-F_ = 30.3 Hz, 2Cq), 129.7 (2CH), 128.9 and 122.7 (CCl_2_ = CCl), 126.5 (2CH), 125.5 (q, ^3^*J*_C-F_ = 5.3 Hz, 2CH), 123.5 (q, ^1^*J*_C-F_ = 274.0 Hz, 2Cq), 109.9 (C-NO_2_), 14.1 (q, ^4^*J*_C-F_ = 2.4 Hz, 2CH_3_) ppm. MS: *m*/*z* (%) = 548 (3) [M^+^], 512 (5) [M—Cl]^+^, 477 (40) [M—2 Cl]^+^, 417 (10) [M—C_2_Cl_3_]^+^, 109 (100).

*Methyl 2-((5-(methylamino)-4-nitro-1H-pyrazol-3-yl)methylene)hydrazine-1-carboxylate* (**3a**). To a suspension of 261 mg (1.00 mmol) diene **2a** in 20 mL MeOH, 361 mg (4.00 mmol) methyl hydrazinecarboxylate was added, and the resulting mixture was refluxed for 8 h. Subsequently, the supernatant liquid was concentrated in vacuo to a volume of 10 mL and cooled to rt. The precipitate was filtered off, washed with MeOH (3 mL), 5% hydrochloric acid (3 × 5 mL), water (5 mL), and MeOH again (3 mL), and finally dried under reduced pressure; yield: 162 mg (0.67 mmol, 67%) pyrazole **2a** as a single isomer, yellow solid, mp 243–245 °C. IR (ATR): *ṽ*_max_ = 3401, 3195, 1744, 1656, 1579, 1538, 1507, 1489, 1425, 1354, 1309, 1237, 1207, 1163, 1125, 1092, 1043, 1004, 969, 891, 854, 752, 710, 648, 578, 544, 457, 413 cm^−1^. ^1^H-NMR (400 MHz, DMSO-*d_6_*): *δ* = 13.33 (br. s, 1H, NH), 11.92 (br. s, 1H, NH), 7.94 (q, *J* = 5.1 Hz, 1H, N*H*-Me), 7.86 (s, 1H, HC=N), 3.73 (s, 3H, OMe), 2.94 (d, *J* = 5.1 Hz, 3H, NH-*Me*) ppm. ^13^C-NMR (101 MHz, DMSO-*d_6_*): *δ* = 153.8 (C=O), 148.1 (Cq), 140.6 (Cq), 127.9 (HC=N), 114.5 (CNO_2_), 52.8 (OCH_3_), 30.1 (NCH_3_) ppm. MS: *m*/*z* (%) = 242 (25) [M^+^], 196 (10) [M—NO_2_]^+^, 102 (100) [M—C_3_H_5_N_2_O_2_]^+^.

*Methyl 2-((5-(morpholino)-4-nitro-1H-pyrazol-3-yl)methylene)hydrazine-1-carboxylate* (**3b**). The product was prepared according to pyrazole **3a** from diene **2b** (373 mg, 1.00 mmol) and methyl hydrazinecarboxylate (361 mg, 4.00 mmol). Reaction time was 12 h. The product was a yellow solid, yield 254 mg (0.85 mmol, 85%), mp 234–236 °C. IR (ATR): *ṽ*_max_ = 3236, 2854, 1726 (CO), 1547 (NO_2_), 1457, 1350 (NO_2_), 1249, 1114, 1060, 919, 770, 609 cm^−1^. ^1^H-NMR (400 MHz, DMSO-*d_6_*): *δ* = 13.84 (br. s, 1H, NH), 11.65 (br. s, 1H, NH), 8.46 (d, ^1^*J*_C-H_ = 176.8 Hz, 1H, CH=N), 3.72 (br. s, 7H, OMe + 2OCH_2_), 3.16 (br. s, 4H, 2NCH_2_) ppm. ^13^C-NMR (101 MHz, DMSO-*d_6_*): *δ* = 153.8 (C=O), 153.6 (Cq), 137.8 (Cq), 132.6 (HC=N), 122.9 (CNO_2_), 65.9 (2OCH_2_), 52.5 (OCH_3_), 49.9 (2NCH_2_) ppm. MS: *m*/*z* (%) = 298 (10) [M^+^], 384 (22), 267 (100) [M—OMe]^+^.

*Methyl 2-((5-((2-(4-chlorophenoxy)ethyl)amino)-4-nitro-1H-pyrazol-3-yl)methylene)-hydrazine-1-carboxylate* (**3c**). The product was prepared according to pyrazole **3a** from diene **2c** (542 mg, 1.00 mmol) and methyl hydrazinecarboxylate (361 mg, 4.00 mmol). Reaction time was 8 h. Yellow solid, yield 249 mg (0.65 mmol, 65%) mp 236–238 °C. IR (ATR): *ṽ*_max_ = 3398, 3293, 2951, 1745, 1646, 1550, 1491, 1244, 1052, 819, 764, 669, 502 cm^−1^. ^1^H-NMR (400 MHz, DMSO-*d_6_*): *δ* = 13.63 (br. s, 0.4H, NH) and 13.01 (br. s, 0.6H, NH), 11.62 (br. s, 0.4H, NH) and 11.31 (br. s, 0.6H, NH), 8.44 (d, ^1^*J*_C-H_ = 173.5 Hz, 1H, CH=N), 7.81 (br. s, 1H, NH), 7.31 (d, *J* = 8.8 Hz, 2CH), 6.97 (d, *J* = 8.8 Hz, 2CH), 6.69 (br. s, 1H, NH), 4.16 (t, *J* = 5.7 Hz, 2H, OCH_2_), 3.77 (br. s, 5H, OMe + NHC*H_2_*) ppm. ^13^C-NMR (101 MHz, DMSO-*d_6_*): *δ* = 157.7 (Cq-O), 154.0 (C=O), 148.1 (Cq), 135.9 (Cq), 129.7 (HC=N), 129.5 (2CH), 124.6 (C-Cl), 116.4 (2CH), 114.6 (CNO_2_), 66.6 (OCH_2_), 52.3 (OCH_3_), 42.4 (HNCH_2_) ppm. MS: *m*/*z* (%) = 382 (25) [M^+^], 347 (21) [M—Cl]^+^, 255 (45) [M—C_6_H_4_OCl]^+^, 241 (80) [M—C_7_H_4_OCl]^+^, 128 (60) [M—C_6_H_4_OCl—C_2_H_3_O]^+^.

*Methyl 2-((5-(2,6-difluorobenzylamino)-4-nitro-1H-pyrazol-3-yl)methylene)hydrazine-1-carboxylate* (**3d**). The product was prepared according to pyrazole **3a** from diene **2d** (485 mg, 1.00 mmol) and methyl hydrazinecarboxylate (361 mg, 4.00 mmol). Reaction time was 12 h. The product was purified by column chromatography (CC) using a mixture of PE/EA 2:1 as eluent. Orange solid, yield 301 mg (0.85 mmol, 85%), mp 238–241 °C. IR (ATR): *ṽ*_max_ = 3421, 3369, 3222, 3131, 3064, 1704, 1592, 1469, 1383, 1337, 1260, 1165, 1041, 763, 663, 495 cm^−1^. ^1^H-NMR (400 MHz, DMSO-*d_6_*): *δ* = 13.65 (br. s, 0.65H, NH) and 13.16 (br. s, 0.35H, NH), 11.60 (br. s, 0.65H, NH) and 11.32 (br. s, 0.35H, NH), 8.43 (d, ^1^*J*_C-H_ = 175.4 Hz, 1H, CH=N), 7.86 (br. s, 0.35H, NH) and 6.68 (br. s, 0,65H, NH), 7.40 (br. s, 1CH), 7.09 (br. s, 2CH), 4.65 (br. s, 0.70H, NCH_2_) and 4.58 (br. s, 1.30H, NCH_2_), 3.71 (s, 3H, OMe) ppm. ^13^C-NMR (101 MHz, DMSO-*d_6_*): *δ* = 161.2 (d, ^1^*J*_C-F_ = 246.4 Hz, 2Cq-F), 153.7 (Cq), 151.4 (Cq), 147.8 (Cq), 135.7 (CH), 130.2 (HC=N), 118.7 (Cq), 114.8 (C-NO_2_), 111.9 (2CH), 52.5 (OCH_3_), 34.6 (NCH_2_) ppm. MS: *m*/*z* (%) = 354 (2) [M^+^], 308 (5) [M—NO_2_]^+^, 267 (100).

*Methyl 2-((5-(phenylamino)-4-nitro-1H-pyrazol-3-yl)methylene)hydrazine-1-carboxylate* (**3e**). The product was prepared according to pyrazole **3a** from diene **2e** (385 mg, 1.00 mmol) and methyl hydrazinecarboxylate (361 mg, 4.00 mmol). Reaction time was 7 h. Red solid, yield 225 mg (0.74 mmol, 74%), mp 233–235 °C. IR (ATR): *ṽ*_max_ = 3381, 3247, 3180, 3055, 1731, 1596, 1539, 1366, 1306, 1285, 1232, 1157, 1054, 958, 916, 740, 648, 559, 498 cm^−1^. ^1^H-NMR (400 MHz, DMSO-*d_6_*): *δ* = 13.95 (br. s, 1H, NH), 11.70 (br. s, 1H, NH), 8.63 (br. s, 1H, NH), 8.51 (d, ^1^*J*_C-H_ = 177.2 Hz, 1H, CH=N), 7.70 (d, *J* = 7.4 Hz, 2CH), 7.31 (t, *J* = 7.4 Hz, 2CH), 6.96 (t, *J* = 7.3 Hz, 1CH), 3.73 (s, ^1^*J*_C-H_ = 147.4 Hz, 3H, OMe) ppm. ^13^C-NMR (101 MHz, DMSO-*d_6_*): *δ* = 153.8 (C=O), 147.6 (Cq), 140.3 (Cq-NH), 135.7 (Cq), 132.2 (CH=N), 129.0 (2CH), 121.6 (1CH), 119.4 (C-NO_2_), 118.2 (2CH), 52.6 (OCH_3_) ppm. MS: *m*/*z* (%) = 304 (70) [M^+^], 273 (15) [M—CH_3_O]^+^, 272 (100) [M—CH_3_O—H]^+^.

*Ethyl 2-((5-(phenylamino)-4-nitro-1H-pyrazol-3-yl)methylene)hydrazine-1-carboxylate* (**3ee**). The product was prepared in EtOH according to pyrazole **3a** from diene **2e** (385 mg, 1.00 mmol) and ethyl hydrazinecarboxylate (417 mg, 4.00 mmol). Reaction time was 7 h. Red solid, yield 191 mg (0.60 mmol, 60%) mp 249–251 °C. IR (KBr): *ṽ*_max_ = 3199, 1713 (CO), 1603, 1554 (NO_2_), 1362 (NO_2_), 1254, 1168, 1058, 916, 741, 563 cm^−1^. ^1^H-NMR (400 MHz, DMSO-*d_6_*): *δ* = 13.94 (br. s, 1H, NH), 11.69 (br. s, 1H, NH), 8.64 (br. s, 1H, NH), 8.51 (s, 1H, CH=N), 7.69 (d, *J* = 7.4 Hz, 2CH), 7.31 (t, *J* = 7.4 Hz, 2CH), 6.97 (t, *J* = 7.4 Hz, 1CH), 4.19 (q, *J* = 7.0 Hz, 2H, OCH_2_), 1.25 (t, *J* = 7.0 Hz, 3H, CH_3_) ppm. ^13^C-NMR (101 MHz, DMSO-*d_6_*): *δ* = 153.2 (C=O), 147.6 (Cq), 140.3 (Cq-NH), 135.9 (Cq), 132.2 (CH=N), 129.0 (2CH), 121.6 (1CH), 119.3 (C-NO_2_), 118.2 (2CH), 61.2 (OCH_2_), 14.6 (CH_3_) ppm. MS: *m*/*z* (%) = 318 (50) [M^+^], 272 (100) [M—NO_2_]^+^. HRMS-ESI: *m*/*z* calcd. for C13H14N6O4Na [M + Na]^+^: 341.0969; found: 341.0965.

*Methyl 2-((5-(4-fluorophenylamino)-4-nitro-1H-pyrazol-3-yl)methylene)hydrazine-1-carboxylate* (**3f**). The product was prepared according to pyrazole **3a** from diene **2f** (421 mg, 1.00 mmol) and methyl hydrazinecarboxylate (361 mg, 4.00 mmol). Reaction time was 5 h. Orange solid, yield 168 mg (0.52 mmol, 52%) mp 225–227 °C. IR (KBr): *ṽ*_max_ = 3378, 3230, 1720 (CO), 1613, 1554 (NO_2_), 1510, 1364 (NO_2_), 1255, 1165, 1055, 827, 790, 506 cm^−1^. ^1^H-NMR (400 MHz, DMSO-*d_6_*): *δ* = 13.93 (br. s, 1H, NH), 11.70 (br. s, 1H, NH), 8.66 (br. s, 1H, NH), 8.51 (s, 1H, CH=N), 7.74 (dd, *J* = 8.4, 5.4 Hz, 2CH), 7.14 (t, *J* = 8.4 Hz, 2CH), 3.73 (s, 3H, OMe) ppm. ^13^C-NMR (101 MHz, DMSO-*d_6_*): *δ* = 157.3 (d, ^1^*J*_C-F_ = 237.2 Hz, Cq-F), 153.8 (C=O), 147.7 (Cq), 136.9 (Cq-NH), 135.8 (Cq), 132.2 (CH=N), 120.0 (d, ^3^*J*_C-F_ = 8.1 Hz, 2CH), 119.6 (C-NO_2_), 115.4 (d, ^2^*J*_C-F_ = 22.5 Hz, 2CH), 52.6 (OCH_3_) ppm. MS: *m*/*z* (%) = 322 (90) [M^+^], 290 (100) [M—OMe—H]^+^, 247 [M—NHCO_2_Me]^+^.

*Ethyl 2-((5-(4-fluorophenylamino)-4-nitro-1H-pyrazol-3-yl)methylene)hydrazine-1-carboxylate* (**3ff**). The product was prepared in EtOH according to pyrazole **3a** from diene **2f** (421 mg, 1.00 mmol) and ethyl hydrazinecarboxylate (417 mg, 4.00 mmol). Reaction time was 4 h. Orange solid, yield 185 mg (0.55 mmol, 55%) mp 251–253 °C. IR (KBr): *ṽ*_max_ = 3381, 3196, 1715 (CO), 1612, 1587, 1557 (NO_2_), 1510, 1362 (NO_2_), 1258, 1169, 1061, 826, 791, 561, 509 cm^−1^. ^1^H-NMR (400 MHz, DMSO-*d_6_*): *δ* = 13.92 (br. s, 1H, NH), 11.71 (br. s, 1H, NH), 8.68 (br. s, 1H, NH), 8.50 (s, 1H, CH=N), 7.74 (br. s, 2CH), 7.15 (t, *J* = 8.1 Hz, 2CH), 4.18 (q, *J* = 6.9 Hz, 2H, OCH_2_), 1.25 (t, *J* = 6.9 Hz, 3H, CH_3_) ppm. ^13^C-NMR (101 MHz, DMSO-*d_6_*): *δ* = 157.3 (d, ^1^*J*_C-F_ = 238.2 Hz, Cq-F), 153.3 (C=O), 147.7 (Cq), 136.9 (Cq-NH), 135.8 (Cq), 132.1 (CH=N), 119.9 (d, ^3^*J*_C-F_ = 7.7 Hz, 2CH), 119.3 (C-NO_2_), 115.4 (d, ^2^*J*_C-F_ = 22.7 Hz, 2CH), 61.3 (OCH_2_), 14.7 (CH_3_) ppm. MS: *m*/*z* (%) = 322 (90) [M^+^], 290 (100) [M—OMe—H]^+^, 247 [M—NHCO_2_Me]^+^.

*Methyl 2-((5-(4-chlorophenylamino)-4-nitro-1H-pyrazol-3-yl)methylene)hydrazine-1-carboxylate* (**3g**). The product was prepared according to pyrazole **3a** from diene **2g** (454 mg, 1.00 mmol) and methyl hydrazinecarboxylate (361 mg, 4.00 mmol). Reaction time was 8 h. Orange solid, yield 305 mg (0.90 mmol, 90%) mp 248–251 °C. IR (ATR): *ṽ*_max_ = 3365, 3279, 3211, 1729, 1599, 1545,1359, 1248, 1164, 1052, 815, 763, 606, 506 cm^−1^. ^1^H-NMR (400 MHz, DMSO-*d_6_*): *δ* = 13.93 (br. s, 1H, NH), 11.70 (br. s, 1H, NH), 8.76 (br. s, 1H, NH), 8.50 (s, 1H, CH=N), 7.75 (d, *J* = 8.6 Hz, 2CH), 7.34 (d, *J* = 8.6 Hz, 2CH), 3.73 (s, 3H, CH_3_) ppm. ^13^C-NMR (101 MHz, DMSO-*d_6_*): *δ* = 153.7 (C=O), 147.2 (Cq), 139.4 (Cq-NH), 135.8 (Cq), 132.1 (CH=N), 128.7 (2CH), 125.0 (C-Cl), 119.8 (2CH), 119.4 (C-NO_2_), 52.6 (OCH_3_) ppm. MS: *m*/*z* (%) = 338 (75) [M^+^], 308 (50) [M—CH_3_O]^+^, 305 (100) [M—Cl]^+^, 250 (55) [M—C_2_H_4_N_2_O_2_]^+^.

*Ethyl 2-((5-(4-chlorophenylamino)-4-nitro-1H-pyrazol-3-yl)methylene)hydrazine-1-carboxylate* (**3gg**). The product was prepared in EtOH according to pyrazole **3a** from diene **2g** (454 mg, 1.00 mmol) and ethyl hydrazinecarboxylate (417 mg, 4.00 mmol). Reaction time was 4 h. Red solid, yield 282 mg (0.80 mmol, 80%) mp 251–253 °C. IR (KBr): *ṽ*_max_ = 3375, 3193, 1711 (CO), 1604, 1564 (NO_2_), 1493, 1363 (NO_2_), 1255, 1169, 1058, 916, 822, 764, 569, 506 cm^−1^. ^1^H-NMR (400 MHz, DMSO-*d_6_*): *δ* = 13.98 (br. s, 1H, NH), 11.71 (br. s, 1H, NH), 8.76 (br. s, 1H, NH), 8.50 (s, 1H, CH=N), 7.76 (d, *J* = 8.5 Hz, 2CH), 7.35 (d, *J* = 8.5 Hz, 2CH), 4.19 (q, *J* = 7.1 Hz, 2H, OCH_2_), 1.25 (t, *J* = 7.1 Hz, 3H, CH_3_) ppm. ^13^C-NMR (101 MHz, DMSO-*d_6_*): *δ* = 153.2 (C=O), 147.2 (Cq), 139.4 (Cq-NH), 135.9 (Cq), 132.0 (CH=N), 128.7 (2CH), 125.0 (C-Cl), 119.8 (2CH), 119.4 (C-NO_2_), 61.3 (OCH_2_), 14.6 (CH_3_) ppm. MS: *m*/*z* (%) = 352 (18) [M^+^], 306 (28) [M—NO_2_]^+^, 267 (100).

*Methyl 2-((5-(2-chlorophenylamino)-4-nitro-1H-pyrazol-3-yl)methylene)hydrazine-1-carboxylate* (**3h**) was prepared according to the literature [17]. All spectral data are in accordance with the literature [17].

*Methyl 2-((5-(2,4-dichlorophenylamino)-4-nitro-1H-pyrazol-3-yl)methylene)hydrazine-1-carboxylate* (**3i**). The product was prepared according to pyrazole **3a** from diene **2i** (522 mg, 1.00 mmol) and methyl hydrazinecarboxylate (361 mg, 4.00 mmol). Reaction time was 5 h. Orange solid, yield 347 mg (0.93 mmol, 93%) mp 237–238 °C. IR (KBr): *ṽ*_max_ = 3278, 1718 (CO), 1601, 1556 (NO_2_), 1453, 1375 (NO_2_), 1256, 1168, 1058, 850, 765, 583 cm^−1^. ^1^H-NMR (400 MHz, DMSO-*d_6_*): *δ* = 14.19 (br. s, 1H, NH), 11.76 (br. s, 1H, NH), 9.03 (br. s, 1H, NH), 8.50 (d, *J* = 178.5 Hz, 1H, CH=N), 8.39 (d, *J* = 9.0 Hz, 1CH), 7.69 (d, *J* = 2.5 Hz, 1CH), 7.49 (dd, *J* = 9.0, 2.5 Hz, 1CH), 3.74 (s, 3H, OMe) ppm. ^13^C-NMR (101 MHz, DMSO-*d_6_*): *δ* = 153.7 (C=O), 146.4 (Cq), 136.1 (Cq), 135.5 (Cq), 131.8 (CH=N), 128.7 (1CH), 128.4 (1CH), 125.1 (C-Cl), 121.1 (C-Cl), 119.7 (C-NO_2_), 119.0 (1CH), 52.6 (OCH_3_) ppm. MS: *m*/*z* (%) = 372 (12) [M^+^], 337 (30) [M—Cl]^+^, 302 [M—2Cl]^+^.

*Methyl 2-((5-(2-methyl-3-(trifluoromethyl)phenylamino)-4-nitro-1H-pyrazol-3-yl)methylene)hydrazine-1-carboxylate* (**3j**). The product was prepared according to pyrazole **3a** from diene **2j** (549 mg, 1.00 mmol) and methyl hydrazinecarboxylate (361 mg, 4.00 mmol). Reaction time was 5 h. Red solid, yield 301 mg (0.78 mmol, 78%) mp 233–234 °C. IR (ATR): *ṽ*_max_ = 3375, 3284, 3165, 1737, 1569, 1537, 1366, 1311, 1238, 113, 1020, 788, 623, 578 cm^−1^. ^1^H-NMR (400 MHz, DMSO-*d_6_*): *δ* = 14.01 (br. s, 1H, NH), 11.73 (br. s, 1H, NH), 8.59 (br. s, 1H, NH), 8.51 (s, 1H, CH=N), 8.32 (d, *J* = 7.9 Hz, 1CH), 7.44 (t, *J* = 7.9 Hz, 1CH), 7.38 (d, *J* = 7.9 Hz, 1CH), 3.73 (s, 3H, OMe), 2.38 (s, 3H, Me) ppm. ^13^C-NMR (101 MHz, DMSO-*d_6_*): *δ* = 153.7 (C=O), 147.8 (Cq), 140.1 (Cq), 136.0 (Cq), 132.0 (CH=N), 128.4 (q, ^2^*J*_C-F_ = 29.7 Hz, Cq), 127.0 (CH), 125.1 (Cq), 123.2 (q, ^1^*J*_C-F_ = 274.3 Hz, CF_3_), 123.2 (CH), 119.6 (C-NO_2_), 119.4 (CH), 52.6 (OMe), 13.3 (2CH_3_) ppm. MS: *m*/*z* (%) = 400 (1) [M^+^], 386 (100) [M—CH_3_ + H]^+^, 354 [M—NO_2_]^+^, 323 (20) [M—NO_2_—CH_3_O]^+^, 267 (85) [M—C_4_H_7_N_2_O_2_—F + H]^+^.

*2-(Methylamino)-3-nitropyrazolo[1,5-d][1,2,4]triazin-7(6H)-one* (**4a**). To a soln. of 242 mg (1.00 mmol) pyrazole **3a** in 10 mL DMF, 98 mg (1.50 mmol) sodium azide was added at rt, and the resulting mixture was stirred at 85–90 °C for 10 h. Subsequently, after cooling to rt and the addition of 60 mL cold water, 1 mL conc. hydrochloric acid was added dropwise. After 5 min of stirring, the precipitate was filtered off, washed with 5 mL water and cold MeOH (2 × 3 mL), and finally dried under reduced pressure to give 198 mg (0.94 mmol, 94%) of the desired triazinone **4a** as a yellow solid, mp 307–309 °C. IR (ATR): *ṽ*_max_ = 3411, 3235, 1765, 1623, 1580, 1470, 1413, 1370, 1318, 1295, 1175, 1141, 1037, 899, 765, 728, 638, 614, 528, 457, 415 cm^−1^. ^1^H-NMR (400 MHz, DMSO-*d_6_*): *δ* = 13.48 (br. s, 1H, NH), 8.58 (d, ^1^*J*_C-H_ = 201.5 Hz, 1H, HC=N), 7.29 (q, *J* = 4.6 Hz, 1H, NH), 2.94 (d, *J* = 4.6 Hz, 3H, Me) ppm. ^13^C-NMR (101 MHz, DMSO-*d_6_*): *δ* = 153.3 (C=O), 142.9 (Cq-NH), 134.3 (Cq-N), 128.1 (CH=N), 114.9 (CNO_2_), 29.5 (Me) ppm. MS: *m*/*z* (%) = 210 (100) [M^+^], 164 (15) [M—NO_2_]^+^, 135 (15) [M—NO_2_—NHCH_3_]^+^, 96 (25) [M—C_3_H_4_N_3_O_2_]^+^. HRMS-ESI: *m*/*z* calcd. for C6H6N6O3Na [M + Na]^+^: 233.0394; found: 233.0393.

*2-(Morpholino)-3-nitropyrazolo[1,5-d][1,2,4]triazin-7(6H)-one* (**4b**). The product was prepared according to triazinone **4a** from pyrazole **3b** (298 mg, 1.00 mmol) and sodium azide (98 mg, 1.50 mmol) at 95–100 °C. Reaction time was 4 h. Yellow solid, yield 347 mg (0.90 mmol, 90%) mp 251–253 °C. IR (ATR): *ṽ*_max_ = 3178, 2968, 1748 (CO), 1601, 1514 (NO_2_), 1423, 1358 (NO_2_), 1323, 1194, 1105, 955, 849, 735, 643 cm^−1^. ^1^H-NMR (400 MHz, DMSO-*d_6_*): *δ* = 13.57 (br. s, 1H, NH), 8.66 (d, ^1^*J*_C-H_ = 204.5 Hz, 1H, HC=N), 3.92–3.56 (m, 4H, 2OCH_2_), 3.43–3.27 (m, 4H, 2NCH_2_) ppm. ^13^C-NMR (101 MHz, DMSO-*d_6_*): *δ* = 154.7 (C=O), 142.6 (Cq-N), 136.0 (Cq-N), 128.9 (CH=N), 118.7 (CNO_2_), 65.7 (2OCH_2_), 49.6 (2NCH_2_) ppm. MS: *m*/*z* (%) = 266 (30) [M^+^], 249 (100) [M—OH]^+^, 231 (37) [M—OH—NO_2_]^+^.

*2-(2-(4-Chlorophenoxy)ethylamino)-3-nitropyrazolo[1,5-d][1,2,4]triazin-7(6H)-one* (**4c**). The product was prepared according to triazinone **4a** from pyrazole **3c** (383 mg, 1.00 mmol) and sodium azide (98 mg, 1.50 mmol) at 95–100 °C. Reaction time was 12 h. Yellow solid, yield 235 mg (0.67 mmol, 67%) mp 198 °C. IR (ATR): *ṽ*_max_ = 3395, 3260, 1757, 1703, 1613, 1578, 1491, 1464, 1297, 1242, 1169, 1092, 1057, 892, 820, 765, 722, 666, 504 cm^−1^. ^1^H-NMR (400 MHz, DMSO-*d_6_*): *δ* = 13.53 (br. s, 1H, NH), 8.60 (s, 1H, HC=N), 7.41 (t, *J* = 6.0 Hz, 1H, NH), 7.31 (d, *J* = 8.2 Hz, 2CH), 7.02 (d, *J* = 8.2 Hz, 2CH), 4.22 (t, *J* = 6.2 Hz, 2H, OCH_2_), 3.77 (q, *J* = 6.0 Hz, 2H, NHC*H_2_*) ppm. ^13^C-NMR (101 MHz, DMSO-*d_6_*): *δ* = 157.4 (Cq-O), 152.7 (C=O), 142.8 (NCN), 134.3 (Cq), 129.4 (2CH), 128.3 (HC=N), 124.6 (C-Cl), 116.5 (2CH), 115.1 (CNO_2_), 65.7 (OCH_2_), 41.7 (NCH_2_) ppm. MS: *m*/*z* (%) = 350 (15) [M^+^], 305 (10) [M—NO_2_ + H]^+^, 223 (100) [M—C_6_H_4_ClO]^+^.

*2-(2,6-Difluorobenzylamino)-3-nitropyrazolo[1,5-d][1,2,4]triazin-7(6H)-one* (**4d**). The product was prepared according to triazinone **4a** from pyrazole **3d** (354 mg, 1.00 mmol) and sodium azide (98 mg, 1.50 mmol) at 80–85 °C. Reaction time was 12 h. Yellow solid, yield 277 mg (0.86 mmol, 86%) mp 236–238 °C. IR (ATR): *ṽ*_max_ = 3415, 31, 25, 2929, 1728, 1621, 1580, 1467, 1326, 1174, 1022, 873, 808785, 768, 730, 640, 532, 489, 439 cm^−1^. ^1^H-NMR (400 MHz, DMSO-*d_6_*): *δ* = 13.52 (br. s, 1H, NH), 8.58 (d, ^1^*J*_C-H_ = 202.8 Hz, 1H, HC=N), 7.50 (t, *J* = 5.6 Hz, 1NH), 7.39 (tt, *J* = 6.8, 8.2 Hz, 1CH), 7.09 (t, *J* = 8.2 Hz, 2CH), 4.68 (d, ^1^*J*_C-H_ = 142.2 Hz, d *J* = 5.6 Hz, 2H, CH_2_) ppm. ^13^C-NMR (101 MHz, DMSO-*d_6_*): *δ* = 161.5 (d, ^1^*J*_C-F_ = 248.8 Hz, Cq-F), 161.4 (d, ^1^*J*_C-F_ = 248.0 Hz, Cq-F), 152.2 (NCN), 142.8 (C=O), 134.4 (Cq), 130.2 (t, ^3^*J*_C-F_ = 10.5 Hz, CH), 128.0 (HC=N), 115.0 (C-NO_2_), 114.0 (t, ^2^*J*_C-F_ = 18.5 Hz, Cq), 111.7 (dd, ^2^*J*_C-F_ = 19.3 Hz, ^4^*J*_C-F_ = 6.5 Hz, 2CH), 34.8 (t, ^3^*J*_C-F_ = 4.0 Hz, NCH_2_) ppm. MS: *m*/*z* (%) = 292 (50) [M—CH_2_NH—H]^+^ 164 (60) [M—C_7_H_5_F_2_—NH—NO_2_]^+^, 149 (25) [M—C_7_H_5_F_2_—NO_2_]^+^, 127 (100) [C_7_H_5_F_2_]^+^.

*2-(Phenylamino)-3-nitropyrazolo[1,5-d][1,2,4]triazin-7(6H)-one* (**4e**). The product was prepared according to triazinone **4a** either from pyrazole **3e** (304 mg, 1.00 mmol) or from pyrazole **3ee** (318 mg, 1.00 mmol) and sodium azide (98 mg, 1.50 mmol) at 105–110 °C. Reaction time was 4 h. Orange solid, yield 253 mg (0.93 mmol, 93%) from **3e** and 240 mg (0.88 mmol, 88%) from 3**ee**, mp 275–277 °C. IR (KBr): *ṽ*_max_ = 3345, 1721 (CO), 1615, 1501 (NO_2_), 1376 (NO_2_), 1321, 1293, 1173, 1019, 903, 840, 764, 693, 642 cm^−1^. ^1^H-NMR (400 MHz, DMSO-*d_6_*): *δ* = 13.64 (br. s, 1H, NH), 9.04 (br. s, 1H, NH), 8.70 (d, ^1^*J*_C-H_ = 204.7 Hz, 1H, HC=N), 7.84 (d, *J* = 7.9 Hz, 2CH), 7.39 (t, *J* = 7.9 Hz, 2CH), 7.08 (t, *J* = 7.9 Hz, 1CH) ppm. ^13^C-NMR (101 MHz, DMSO-*d_6_*): *δ* = 148.9 (Cq), 142.7 (Cq), 139.3 (Cq), 133.9 (Cq), 129.0 (2CH), 128.4 (HC=N), 123.0 (CH), 119.4 (2CH), 115.8 (CNO_2_) ppm. MS: *m*/*z* (%) = 272 (100) [M^+^], 226 (5) [M—NO_2_]^+^, 149 (10) [M—C_6_H_5_—NO_2_]^+^.

*2-(4-Fluorophenylamino)-3-nitropyrazolo[1,5-d][1,2,4]triazin-7(6H)-one* (**4f**). The product was prepared according to triazinone **4a** either from pyrazole **3f** (322 mg, 1.00 mmol) or from pyrazole **3ff** (336 mg, 1.00 mmol) and sodium azide (98 mg, 1.50 mmol) at 105–110 °C. Reaction time was 4 h. Orange solid, yield 276 mg (0.95 mmol, 95%) from **3e** and 261 mg (0.90 mmol, 90%) from 3**ee**, mp 290 °C. IR (KBr): *ṽ*_max_ = 3368, 3151, 1727 (CO), 1616, 1585, 1509, 1371 (NO_2_), 1315, 1171, 907, 834, 765, 644, 509 cm^−1^. ^1^H-NMR (400 MHz, DMSO-*d_6_*): *δ* = 13.64 (br. s, 1H, NH), 9.01 (br. s, 1H, NH), 8.68 (d, ^1^*J*_C-H_ = 204.7 Hz, 1H, HC=N), 7.84 (dd, *J* = 8.1, 5.2 Hz, 2CH), 7.22 (t, *J* = 8.1 Hz, 2CH) ppm. ^13^C-NMR (101 MHz, DMSO-*d_6_*): *δ* = 158.2 (d, ^1^*J*_C-F_ = 239.3 Hz, Cq-F), 149.1 (Cq), 142.7 (Cq), 135.8 (d, ^4^*J*_C-F_ = 2.2 Hz, Cq-NH), 133.9 (Cq), 128.4 (CH=N), 121.5 (d, ^3^*J*_C-F_ = 7.7 Hz, 2CH), 115.6 (C-NO_2_), 115.5 (d, ^2^*J*_C-F_ = 22.3 Hz, 2CH) ppm. MS: *m*/*z* (%) = 290 (100) [M^+^], 256 (2), 244 (1) [M—NO_2_]^+^.

*2-(4-Chlorophenylamino)-3-nitropyrazolo[1,5-d][1,2,4]triazin-7(6H)-one* (**4g**). The product was prepared according to triazinone **4a** either from pyrazole **3g** (339 mg, 1.00 mmol) or from pyrazole **3gg** (353 mg, 1.00 mmol) and sodium azide (98 mg, 1.50 mmol) at 105–108 °C. Reaction time was 12 h. Orange solid, yield 261 mg (0.85 mmol, 85%) from **3g** and 255 mg (0.83 mmol, 83%) from **3gg**, mp 275–278 °C. IR (KBr): *ṽ*_max_ = 3348, 3177, 1746 (CO), 1613, 1572, 1371 (NO_2_), 1320, 1172, 906, 822, 766, 639, 507 cm^−1^. ^1^H-NMR (400 MHz, DMSO-*d_6_*): *δ* = 13.66 (br. s, 1H, NH), 9.20 (br. s, 1H, NH), 8.71 (s, 1H, HC=N), 7.88 (d, *J* = 8.8 Hz, 2CH), 7.43 (d, *J* = 8.8 Hz, 2CH) ppm. ^13^C-NMR (101 MHz, DMSO-*d_6_*): *δ* = 148.7 (Cq), 142.6 (Cq), 138.5 (Cq-NH), 133.9 (Cq), 128.9 (2CH), 128.4 (CH=N), 126.6 (C-Cl), 121.1 (2CH), 115.9 (C-NO_2_) ppm. MS: *m*/*z* (%) = 305 (100) [M—H]^+^, 271 (4) [M—Cl]^+^, 225 (4) [M—Cl—NO_2_]^+^.

*2-(2-Chlorophenylamino)-3-nitropyrazolo[1,5-d][1,2,4]triazin-7(6H)-one* (**4h**) was prepared according to literature [17]. All spectral data are in accordance with the literature [17].

*2-(2,4-Dichlorophenylamino)-3-nitropyrazolo[1,5-d][1,2,4]triazin-7(6H)-one* (**4i**). The product was prepared according to triazinone **4a** from pyrazole **3i** (373 mg, 1.00 mmol) and sodium azide (98 mg, 1.50 mmol) at 106–108 °C. Reaction time was 6 h. Orange solid, yield 290 mg (0.85 mmol, 85%), mp 274 °C. IR (KBr): *ṽ*_max_ = 3340, 3148, 1726 (CO), 1620, 1594, 1552 (NO_2_), 1476, 1374 (NO_2_), 1319, 1173, 907, 854, 764, 638, 611 cm^−1^. ^1^H-NMR (400 MHz, DMSO-*d_6_*): *δ* = 13.76 (br. s, 1H, NH), 9.18 (br. s, 1H, NH), 8.73 (s, 1H, HC=N), 8.38 (d, *J* = 9.0 Hz, 1CH), 7.74 (d, *J* = 2.3 Hz, 1CH), 7.55 (dd, *J* = 9.0, 2.3 Hz, 1CH) ppm. ^13^C-NMR (101 MHz, DMSO-*d_6_*): *δ* = 148.0 (Cq), 142.4 (Cq), 134.4 (Cq-NH), 133.7 (Cq), 129.0 (CH), 128.6 (CH), 128.3 (CH=N), 127.1 (C-Cl), 122.7 (C-Cl), 120.4 (CH), 115.9 (C-NO_2_) ppm. MS: *m*/*z* (%) = 340 (85) [M^+^], 305 (100) [M—Cl]^+^, 289 (5) [M—Cl—O]^+^.

*2-(2-Methyl-3-trifluoromethylphenylamino)-3-nitropyrazolo[1,5-d][1,2,4]triazin-7(6H)-one* (**4j**). The product was prepared according to triazinone **4a** from pyrazole **3j** (386 mg, 1.00 mmol) and sodium azide (98 mg, 1.50 mmol) at 105–107 °C. Reaction time was 12 h. Orange solid, yield 149 mg (0.42 mmol, 42%) mp 218 °C. IR (KBr): *ṽ*_max_ = 3376, 3254, 1739, 1701, 1611, 1579, 1504, 1313, 1278, 1165, 1133, 1110, 1021, 898, 799, 723, 643, 580, 531 cm^−1^. ^1^H-NMR (400 MHz, DMSO-*d_6_*): *δ* = 13.63 (br. s, 1H, NH), 8.94 (br. s, 1H, NH), 8.69 (s, 1H, CH=N), 8.10 (d, *J* = 8.0 Hz, 1CH), 7.56 (d, *J* = 8.0 Hz, 1CH), 7.50 (dd, *J* = 8.0, 8.0 Hz, 1CH), 2.39 (s, 3H, Me) ppm. ^13^C-NMR (101 MHz, DMSO-*d_6_*): *δ* = 150.0 (Cq), 142.6 (Cq), 139.2 (Cq), 134.1 (Cq), 129.4 (q, ^3^*J*_C-F_ = 1.7 Hz, Cq), 128.6 (q, ^2^*J*_C-F_ = 29.1 Hz, Cq), 128.3 (CH), 127.4 (CH), 127.1 (CH=N), 124.6 (q, ^1^*J*_C-F_ = 273.9 Hz, CF_3_), 122.2 (q, ^3^*J*_C-F_ = 5.8 Hz, CH), 115.7 (C-NO_2_), 13.6 (q, ^4^*J*_C-F_ = 2.4 Hz, CH_3_) ppm. MS: *m*/*z* (%) = 354 (100) [M^+^], 307 (25) [M—NO_2_]^+^, 194 (25) [M—ArCH_3_CF_3_]^+^.

*3-Amino-2-(methylamino)pyrazolo[1,5-d][1,2,4]triazin-7(6H)-one* (**5a**). To a suspension of 210 mg (1.00 mmol) of triazinone **4a** in a mixture of MeOH (10 mL) and conc. hydrochloric acid (1 mL), 677 mg (3.00 mmol) tin(II) chloride dihydrate was added at rt, and the resulting mixture was stirred at 60–65 °C for 15 h. Subsequently, after cooling to rt and the addition of 60 mL cold water and 5 mL saturated sodium bicarbonate soln. under stirring, the resulting mixture was extracted with EA (6 × 50 mL), washed with water (1 × 100 mL), and dried with sodium sulfate (Na_2_SO_4_). After purification with CC (EA/PE 3:1), evaporation of the solvents, and drying under reduced pressure, the amine **5a** was obtained as a brownish solid, yield 87 mg (0.48 mmol, 48%), mp 316–318 °C. IR (ATR): *ṽ*_max_ = 3379, 3321, 3197, 1672, 1566, 14, 16, 12, 32, 1048, 717, 591 cm^−1^. ^1^H-NMR (400 MHz, DMSO-*d_6_*): *δ* = 11.47 (br. s, 1H, NH), 8.04 (d, ^1^*J*_C-H_ = 189.8 Hz, 1H, HC=N), 5.76 (q, *J* = 5.0 Hz, 1H, NH), 4.80 (br. s, 2H, NH_2_), 2.83 (d, *J* = 5.0 Hz, 3H, Me) ppm. ^13^C-NMR (101 MHz, DMSO-*d_6_*): *δ* = 152.7 (C=O), 144.5 (Cq-NH), 130.1 (CH=N), 118.8 (Cq), 115.5 (Cq), 29.7 (Me) ppm. MS: *m*/*z* (%) = 180 (100) [M^+^], 163 (15) [M—NH_2_—H]^+^, 95 (20) [M—C_7_H_3_N_3_]^+^.

*3-Amino-2-morpholinopyrazolo[1,5-d][1,2,4]triazin-7(6H)-one* (**5b**). To a suspension of 266 mg (1.00 mmol) of triazinone **4b** in a mixture of EtOH (15 mL) and conc. hydrochloric acid (1.5 mL), 677 mg (3.00 mmol) tin(II) chloride dihydrate was added at rt, and the resulting mixture was stirred at rt for 1 d and at 40–45 °C for 8 h. Subsequently, after cooling to rt, the precipitate was filtered off, washed with 5 mL water and cold MeOH (3 mL), and finally dried under reduced pressure to give 227 mg (0.96 mmol, 96%) of the desired triazinone **5b** as a brownish solid, mp 314–318 °C. IR (ATR): *ṽ*_max_ = 3391, 3221, 2844, 1694, 1498, 1358, 1231, 1109, 1050, 953, 851, 743, 717, 673, 588, 566, 517, 440 cm^−1^. ^1^H-NMR (400 MHz, DMSO-*d_6_*): *δ* = 11.83 (br. s, 1H, NH), 8.23 (d, ^1^*J*_C-H_ = 191.1 Hz, 1H, HC=N), 4.91 (br. s, 2H, NH_2_), 3.79–3.68 (m, 4H, 2OCH_2_), 3.23–3.12 (m, 4H, 2NCH_2_) ppm. ^13^C-NMR (101 MHz, DMSO-*d_6_*): *δ* = 153.0 (C=O), 144.3 (Cq-NH), 130.5 (CH=N), 121.2 (Cq), 118.7 (Cq), 66.0 (OCH_2_), 48.3 (NCH_2_) ppm. MS: *m*/*z* (%) = 236 (100) [M^+^], 151 (50) [M—NC_4_H_8_O + H]^+^. HRMS-ESI: calcd. for C9H12N6O2Na [M + Na]^+^: 259.0914; found: 259.0914.

*3-Amino-2-((2-(4-chlorophenoxy)ethyl)amino)pyrazolo[1,5-d][1,2,4]triazin-7(6H)-one* (**5c**). To a suspension of 351 mg (1.00 mmol) of triazinone **4c** in a mixture of MeOH (20 mL) and conc. hydrochloric acid (2 mL), 677 mg (3.00 mmol) tin(II) chloride dihydrate was added at rt, and the resulting mixture was stirred at 50–55 °C for 12 h. Subsequently, after cooling to rt, the precipitate was filtered off, washed with 7 mL water and cold MeOH (5 mL), and finally dried under reduced pressure to give 237 mg (0.74 mmol, 74%) of the triazinone **5c** as a colorless solid, mp 221–223 °C. IR (ATR): *ṽ*_max_ = 3387, 3270, 1689, 1551, 1491, 1395, 1357, 1245, 1058, 823, 666 cm^−1^. ^1^H-NMR (400 MHz, DMSO-*d_6_*): *δ* = 11.55 (br. s, 1H, NH), 8.06 (d, ^1^*J*_C-H_ = 190.0 Hz, 1H, HC=N), 7.32 (d, *J* = 9.0 Hz, 2CH), 7.02 (d, *J* = 9.0 Hz, 2CH), 6.08 (t, *J* = 5.6 Hz, 1H, NH), 4.90 (br. s, 2H, NH_2_), 4.16 (t, *J* = 5.5 Hz, 2H, OCH_2_), 3.62 (q, *J* = 5.5 Hz, 2H, NHC*H_2_*) ppm. ^13^C-NMR (101 MHz, DMSO-*d_6_*): *δ* = 157.5 (Cq-O), 151.4 (C=O), 144.4 (NCN), 130.1 (HC=N), 129.4 (2CH), 124.5 (C-Cl), 118.9 (Cq), 116.5 (2CH), 115.6 (Cq), 66.6 (OCH_2_), 42.3 (NCH_2_). MS: *m*/*z* (%) = 320 (25) [M^+^], 193 (80) [M—C_6_H_4_OCl]^+^, 179 (100) [M—CO—C_6_H_4_Cl]^+^.

*3-Amino-2-((2,6-difluorobenzyl)amino)pyrazolo[1,5-d][1,2,4]triazin-7(6H)-one* (**5d**). To a suspension of 322 mg (1.00 mmol) triazinone **4d** in a mixture of MeOH (20 mL) and conc. hydrochloric acid (2 mL), 677 mg (3.00 mmol) tin(II) chloride dihydrate was added at rt, and the resulting mixture was stirred at 60–65 °C for 18 h. Subsequently, after cooling to rt, 120 mL cold water was added, and the resulting mixture was extracted with EA (6 × 50 mL), washed with water (1 × 100 mL), and dried with Na_2_SO_4_. After purification with CC (EA/PE 2:1), evaporation of solvents, and drying under reduced pressure, the amine **5d** was obtained as a brownish solid, yield 246 mg (0.84 mmol, 84%), mp 217–219 °C. IR (ATR): *ṽ*_max_ = 3340, 2863, 1680, 1556, 1465, 1390, 1345, 1216, 1046, 900, 796, 723, 694, 663, 585, 491, 433 cm^−1^. ^1^H-NMR (400 MHz, DMSO-*d_6_*): *δ* = 12.13 (br. s, 1H, NH), 8.25 (d, ^1^*J*_C-H_ = 195.8 Hz, 1H, HC=N), 7.44 (tt, *J* = 6.7, 7.9 Hz, 1CH), 7.14 (t, *J* = 7.9 Hz, 2CH), 6.37 (br. s, 1NH), 5.60 (br. s, 2H, NH_2_), 4.48 (d, ^1^*J*_C-H_ = 141.9 Hz, 2H, CH_2_) ppm. ^13^C-NMR (101 MHz, DMSO-d_6_): *δ* = 161.4 (d, ^1^*J*_C-F_ = 248.3 Hz, Cq-F), 161.3 (d, ^1^*J*_C-F_ = 247.9 Hz, Cq-F), 152.2 (Cq), 143.9 (Cq), 130.5 (t, ^3^*J*_C-F_ = 10.5 Hz, 1CH), 129.2 (HC=N), 125.0 (Cq), 114.3 (t, ^2^*J*_C-F_ = 19.0 Hz, Cq), 111.8 (dd, ^2^*J*_C-F_ = 19.1 Hz, ^4^*J*_C-F_ = 6.3 Hz, 2CH), 105.5 (Cq), 34.6 (t, ^3^*J*_C-F_ = 3.8 Hz, NCH_2_) ppm. MS: *m*/*z* (%) = 292 (55) [M^+^], 179 (5) [M—C_6_H_3_F_2_]^+^, 164 (70) [M—C_6_H_3_F_2_CH_2_]^+^, 127 (100) [C_6_H_3_F_2_CH_2_]^+^.

*3-Amino-2-(phenylamino)pyrazolo[1,5-d][1,2,4]triazin-7(6H)-one* (**5e**). The product was prepared according to **5a** from **4e** (272 mg, 1.00 mmol) and tin(II) chloride dihydrate (677 mg, 3.00 mmol). Reaction time was 9 h at 70–75 °C in EtOH. Yellowish solid, yield 216 mg (0.89 mmol, 89%), mp 280–282 °C. IR (ATR): *ṽ*_max_ = 3400, 3325, 3220, 1690, 1598, 1566, 1495, 1387, 1351, 1306, 1206, 1066, 901, 121, 734, 682, 597, 541, 461 cm^−1^. ^1^H-NMR (400 MHz, DMSO-*d_6_*): *δ* = 11.78 (br. s, 1H, NH), 8.46 (br. s, 1H, NH), 8.19 (d, ^1^*J*_C-H_ = 190.8 Hz, 1H, HC=N), 7.65 (dd, *J* = 8.6, 1.0 Hz, 2CH), 7.30 (dd, *J* = 8.6, 7.4 Hz, 2CH), 6.88 (tt, *J* = 7.4, 1.0 Hz, 1CH), 5.12 (br. s, 2H, NH_2_) ppm. ^13^C-NMR (101 MHz, DMSO-*d_6_*): *δ* = 146.7 (Cq), 144.4 (Cq), 141.7 (Cq), 130.3 (HC=N), 129.0 (2CH), 120.2 (1CH), 118.7 (Cq), 117.0 (Cq), 116.4 (2CH) ppm. MS: *m*/*z* (%) = 242 (100) [M^+^], 150 (25) [M—C_6_H_6_N]^+^, 93 (60) [C_6_H_6_N]^+^.

*3-Amino-2-(4-fluorophenylamino)pyrazolo[1,5-d][1,2,4]triazin-7(6H)-one* (**5f**). The product was prepared according to **5a** from **4f** (290 mg, 1.00 mmol) and tin(II) chloride dihydrate (677 mg, 3.00 mmol). Reaction time was 10 h at 65–70 °C. Light brown solid, yield 247 mg (0.95 mmol, 95%), mp 295–297 °C. IR (KBr): *ṽ*_max_ = 3407, 3243, 1690 (CO), 1615, 1579 (NO_2_), 1510, 1394 (NO_2_), 1209, 1069, 819, 712, 500 cm^−1^. ^1^H-NMR (400 MHz, DMSO-*d_6_*): *δ* = 11.97 (br. s, 1H, NH), 8.72 (br. s, 1H, NH), 8.23 (s, 1H, HC=N), 7.66 (dd, *J* = 8.8, 4.8 Hz, 2CH), 7.17 (t, *J* = 8.8 Hz, 2CH), 4.33 (br. s, 2H, NH_2_)m ppm. ^13^C-NMR (101 MHz, DMSO-*d_6_*): *δ* = 156.3 (d, ^1^*J*_C-F_ = 236.3 Hz, Cq-F), 147.3 (Cq), 144.2 (Cq), 138.1 (d, ^4^*J*_C-F_ = 2.1 Hz, Cq-NH), 130.0 (CH=N), 120.6 (Cq), 117.8 (d, ^3^*J*_C-F_ = 7.5 Hz, 2CH), 115.6 (d, ^2^*J*_C-F_ = 22.1 Hz, 2CH), 113.6 (Cq) ppm. MS: *m*/*z* (%) = 260 (100) [M^+^], 244 (10) [M—NH_2_]^+^, 231 (10).

*3-Amino-2-(4-chlorophenylamino)pyrazolo[1,5-d][1,2,4]triazin-7(6H)-one* (**5g**). The product was prepared according to **5a** from **4g** (307 mg, 1.00 mmol) and tin(II) chloride dihydrate (677 mg, 3.00 mmol). Reaction time was 12 h at 65–70 °C. Brown solid, yield 249 mg (0.90 mmol, 90%) mp 327–330 °C. IR (KBr): *ṽ*_max_ = 3406, 3241, 1696 (CO), 1602, 1565 (NO_2_), 1492, 1390 (NO_2_), 1205, 1067, 815, 713, 459 cm^−1^. ^1^H-NMR (400 MHz, DMSO-*d_6_*): *δ* = 11.84 (br. s, 1H, NH), 8.65 (br. s, 1H, NH), 8.20 (s, 1H, HC=N), 7.67 (d, *J* = 8.9 Hz, 2CH), 7.34 (d, *J* = 8.9 Hz, 2CH), 5.14 (br. s, 2H, NH_2_) ppm. ^13^C-NMR (101 MHz, DMSO-*d_6_*): *δ* = 144.6 (Cq), 144.3 (Cq), 140.7 (Cq), 130.4 (CH=N), 128.9 (2CH), 123.4 (C-Cl), 118.8 (Cq), 117.9 (2CH), 117.1 (Cq) ppm. MS: *m*/*z* (%) = 276 (44) [M^+^], 241 (20) [M—Cl]^+^, 225 (12) [M—Cl—NH_2_]^+^, 58 (100).

*3-Amino-2-(2-chlorophenylamino)pyrazolo[1,5-d][1,2,4]triazin-7(6H)-one* (**5h**). The product was prepared according to **5a** from **4h** (307 mg, 1.00 mmol) and tin(II) chloride dihydrate (677 mg, 3.00 mmol). Reaction time was 12 h at 65–70 °C. Light brown solid, yield 235 mg (0.85 mmol, 85%), mp 277–279 °C. IR (KBr): *ṽ*_max_ = 3387, 3278, 1709 (CO), 1601, 1561 (NO_2_), 1475, 1386 (NO_2_), 1212, 1051, 898, 737, 603 cm^−1^. ^1^H-NMR (400 MHz, DMSO-*d_6_*): *δ* = 11.94 (br. s, 1H, NH), 8.27 (s, 1H, HC=N), 8.05 (dd, *J* = 8.2, 1.2 Hz, 1CH), 7.62 (br. s, 1H, NH), 7.45 (dd, *J* = 7.8, 1.2 Hz, 1CH), 7.31 (ddd, *J* = 8.2, 7.4, 1.2 Hz, 1CH), 6.95 (ddd, *J* = 7.8, 7.4, 1.2 Hz, 1CH), 5.24 (br. s, 2H, NH_2_) ppm. ^13^C-NMR (101 MHz, DMSO-*d_6_*): *δ* = 146.4 (Cq), 144.3 (Cq), 138.1 (Cq), 130.5 (CH=N), 129.6 (CH), 127.9 (CH), 121.9 (CH), 121.1 (Cq), 120.2 (Cq), 118.8 (CH), 118.1 (Cq) ppm. MS: *m*/*z* (%) = 276 (100) [M]^+^, 241 (70) [M—Cl]^+^, 225 (15) [M—Cl—NH_2_]^+^, 212 (35).

*3-Amino-2-(2,4-dichlorophenylamino)pyrazolo[1,5-d][1,2,4]triazin-7(6H)-one* (**5i**). The product was prepared according to **5a** from **4i** (341 mg, 1.00 mmol) and tin(II) chloride dihydrate (677 mg, 3.00 mmol). Reaction time was 8 h at 75–80 °C in EtOH. Brown solid, yield 236 mg (0.76 mmol, 76%), mp 198–200 °C. IR (ATR): *ṽ*_max_ = 3387, 3346, 3312, 2899, 1697, 1599, 1553, 1469, 1378, 1306, 1205, 1050, 807, 707, 581, 544, 473, 430 cm^−1^. ^1^H-NMR (400 MHz, DMSO-*d_6_*): *δ* = 11.96 (br. s, 1H, NH), 8.27 (s, 1H, HC=N), 8.05 (d, *J* = 8.8 Hz, 1CH), 7.74 (br. s, 1H, NH), 7.60 (d, *J* = 2.3 Hz, 1CH), 7.39 (dd, *J* = 8.8, 2.3 Hz, 1CH), 5.25 (br. s, 2H, NH_2_) ppm. ^13^C-NMR (101 MHz, DMSO-*d_6_*): *δ =* 145.8 (Cq), 144.2 (Cq), 137.4 (Cq), 130.5 (CH=N), 128.9 (CH), 128.0 (CH), 124.3 (Cq), 121.7 (Cq), 120.2 (Cq), 119.5 (CH), 118.3 (Cq) ppm. MS: *m*/*z* (%) = 314 (100) [M^+^], 275 (18) [M—Cl]^+^, 243 (20) [M—2 Cl]^+^.

*3-Amino-2-(2,4-dichlorophenylamino)pyrazolo[1,5-d][1,2,4]triazin-7(6H)-one* (**5j**). The product was prepared according to **5a** from **4j** (354 mg, 1.00 mmol) and tin(II) chloride dihydrate (677 mg, 3.00 mmol). Reaction time was 8 h at 60–65 °C. Yellowish solid, yield 285 mg (0.88 mmol, 88%) mp 223–225 °C. IR (ATR): *ṽ*_max_ = 3220, 1693, 1557, 1505, 1378, 1316, 1280, 1217, 1168, 1110, 1020, 894, 793, 717, 567, 537 cm^−1^. ^1^H-NMR (400 MHz, DMSO-*d_6_*): *δ* = 11.87 (br. s, 1H, NH), 8.24 (d, ^1^*J*_C-H_ = 190.8 Hz, 1H, HC=N), 7.87 (d, *J* = 7.7 Hz, 1CH), 7.60 (br. s, 1H, NH), 7.39–7.27 (m, 2CH), 5.12 (br. s, 2H, NH_2_), 2.37 (s, 3H, Me) ppm. ^13^C-NMR (101 MHz, DMSO-*d_6_*): *δ* = 146.9 (Cq), 144.3 (Cq), 141.9 (Cq), 130.5 (CH), 128.5 (q, ^2^*J*_C-F_ = 28.6 Hz, Cq), 126.7 (CH=N), 125.6 (q, ^3^*J*_C-F_ = 1.7 Hz, Cq), 124.9 (q, ^1^*J*_C-F_ = 273.9 Hz, CF_3_), 122.9 (CH), 119.9 (Cq), 118.7 (q, ^3^*J*_C-F_ = 5.8 Hz, CH), 118.3 (Cq), 13.8 (q, ^4^*J*_C-F_ = 2.4 Hz, CH_3_) ppm. MS: *m*/*z* (%) = 324 (100) [M^+^], 308 (60) [M—NH_2_]^+^, 268 (20) [M—3 F]^+^.

*N-(3-Acetamido-7-oxo-6,7-dihydropyrazolo[1,5-d][1,2,4]triazin-2-yl)-N-methylacetamide* (**6a**). A soln. of 180 mg (1.00 mmol) amine **5a** in 3 mL acetic anhydride (Ac_2_O) was stirred at rt for 2 d. The formed precipitate was filtered off, washed with cold MeOH (1 mL) and water (2 × 3 mL), and finally dried under reduced pressure to give 248 mg (0.94 mmol, 94%) of the acetamide **6a** as a colorless solid, mp 244–246 °C. IR (ATR): *ṽ*_max_ = 3087, 1747, 1646, 1578, 1489, 13, 25, 1268, 1236, 1147, 1005, 818, 728, 666, 616, 566, 530, 465 cm^−1^. ^1^H-NMR (400 MHz, DMSO-*d_6_*): *δ* = 12.81 (br. s, 1H, NH), 10.05 (br. s, 1H, NH), 8.40 (d, ^1^*J*_C-H_ = 199.2 Hz, 1H, HC=N), 3.10 (s, 3H, N-Me), 2.10 (s, 3H, Me), 1.80 (s, 3H, Me) ppm. ^13^C-NMR (101 MHz, DMSO-*d_6_*): *δ* = 169.8 (C=O), 168.7 (C=O), 149.2 (C=O), 143.5 (Cq), 132.2 (CH=N), 129.0 (Cq), 111.8 (Cq), 34.8 (N-Me), 22.8 (C-Me), 21.8 (C-Me) ppm. MS: *m*/*z* (%) = 264 (15) [M^+^], 178 (20) [M—2 C_2_H_3_O]^+^, 149 (85) [M—C_2_H_3_O—C_7_H_6_F_2_N]^+^, 96 (100) [C_3_H_2_N_3_O]^+^.

*N-(2-Morpholino-7-oxo-6,7-dihydropyrazolo [1,5-d][1,2,4]triazin-3-yl)acetamide* (**6b**). The product was prepared according to **6a** from **5b** (236 mg, 1.00 mmol) and 3 mL Ac_2_O. Reaction time was 6 h at 40–45 °C. Colorless solid, yield 245 mg (0.88 mmol, 88%), mp 318–321 °C. IR (ATR): *ṽ*_max_ = 3277, 3090, 2994, 1716, 1665, 14, 89, 1370, 1264, 119, 971, 810, 725, 677, 611, 584, 519 cm^−1^. ^1^H-NMR (400 MHz, DMSO-*d_6_*): *δ* = 12.52 (br. s, 1H, NH), 9.62 (br. s, 1H, NH), 8.06 (d, ^1^*J*_C-H_ = 195.7 Hz, 1H, HC=N), 3.78–3.67 (m, 4H, 2OCH_2_), 3.30–3.21 (m, 4H, 2NCH_2_), 2.06 (d, ^1^*J*_C-H_ = 123.6 Hz, 3H, Me) ppm. ^13^C-NMR (101 MHz, DMSO-*d_6_*): *δ* = 169.2 (C=O), 156.0 (C=O), 143.7 (NCN), 131.6 (Cq), 130.4 (CH=N), 104.5 (Cq), 65.8 (2OCH_2_), 47.8 (2NCH_2_), 22.9 (Me) ppm. MS: *m*/*z* (%) = 278 (75) [M^+^], 235 (100) [M—C_2_H_3_O]^+^.

*N-(2-((2-(4-Chlorophenoxy)ethyl)amino)-7-oxo-6,7-dihydropyrazolo[1,5-d][1,2,4]triazin-3-yl)acetamide* (**6c**). The product was prepared according to **6a** from **5c** (321 mg, 1.00 mmol) and 3 mL Ac_2_O. Reaction time was 24 h at 40–45 °C. Colorless solid, yield 308 mg (0.85 mmol, 85%), mp 250–252 °C. IR (ATR): *ṽ*_max_ = 3266, 29, 32, 1713, 1657, 1631, 1577, 1517, 1492, 1376, 1281, 1247, 1028, 902, 824, 725, 665, 613, 587, 425 cm^−1^. ^1^H-NMR (400 MHz, DMSO-*d_6_*): *δ =* 12.28 (br. s, 1H, NH), 9.68 (br. s, 1H, NH), 8.15 (d, ^1^*J*_C-H_ = 195.5 Hz, 1H, HC=N), 7.32 (d, *J* = 9.0 Hz, 2CH), 7.03 (d, *J* = 9.0 Hz, 2CH), 6.15 (t, *J* = 5.6 Hz, 1H, NH), 4.17 (t, *J* = 5.6 Hz, 2H, OCH_2_), 3.63 (q, *J* = 5.5 Hz, 2H, NHC*H_2_*), 2.07 (s, 3H, Me) ppm. ^13^C-NMR (101 MHz, DMSO-*d_6_*): *δ* = 168.6 (C=O), 157.5 (Cq-O), 153.5 (C=O), 143.9 (NCN), 130.5 (HC=N), 129.5 (2CH), 127.6 (C-Cl), 124.5 (Cq), 116.5 (2CH), 103.5 (Cq), 66.5 (OCH_2_), 42.2 (NCH_2_), 23.0 (Me) ppm. MS: *m*/*z* (%) = 362 (25) [M^+^], 235 (30) [M—C_6_H_4_ClO]^+^, 221 (90) [M—COC_6_H_4_Cl]^+^, 179 (100) [M—C_2_H_3_O—COC_6_H_4_Cl]^+^.

*N-(2-((2,6-Difluorobenzyl)amino)-7-oxo-6,7-dihydropyrazolo[1,5-d][1,2,4]triazin-3-yl)acetamide* (**6d**). The product was prepared according to **6a** from **5d** (292 mg, 1.00 mmol) and 3 mL Ac_2_O. Reaction time was 2 h at 0 °C and 24 h at rt. After purification by CC (EA/PE 1:1), the complete separation of **6d** and **6dd** proved impossible due to similar physical properties. The isolated acetamide **6d** contained 20% of **6dd**. Brownish solid, yield 281 mg (0.84 mmol, 84%) mp 294–297 °C. IR (ATR): *ṽ*_max_ = 3277, 1719, 1655, 1636, 1578, 1521, 1472, 1350, 1068, 1043, 900, 786, 663, 613, 547, 471, 422 cm^−1^. ^1^H-NMR (400 MHz, DMSO-*d_6_*): *δ* = 12.29 (br. s, 1H, NH), 9.50 (br. s, 1H, NH), 8.17 (s, 1H, HC=N), 7.44 (br. s, 1CH), 7.14 (t, *J* = 7.9 Hz, 2CH), 6.17 (br. s, 1H, NH), 4.49 (br. s, 2H, CH_2_), 2.04 (s, 3H, Me) ppm. ^13^C-NMR (101 MHz, DMSO-*d_6_*): *δ* = 168.5 (C=O), 161.4 (d, ^1^*J*_C-F_ = 248.1 Hz, Cq-F), 161.3 (d, ^1^*J*_C-F_ = 247.6 Hz, Cq-F), 152.9 (Cq), 143.9 (Cq), 130.7 (HC=N), 130.4 (t, ^3^*J*_C-F_ = 10.2 Hz, 1CH), 127.5 (Cq), 114.4 (t, ^2^*J*_C-F_ = 19.6 Hz, Cq), 111.8 (dd, ^2^*J*_C-F_ = 18.5 Hz, ^4^*J*_C-F_ = 6.2 Hz, 2CH), 103.5 (Cq), 34.6 (t, ^3^*J*_C-F_ = 4.4 Hz, NCH_2_), 23.0 (Me) ppm. MS: *m*/*z* (%) = 334 (40) [M^+^], 291 (25) [M—C_2_H_3_O]^+^, 164 (30) [M—C_2_H_3_O—C_7_H_5_F_2_]^+^.

*N-(3-Acetamido-7-oxo-6,7-dihydropyrazolo[1,5-d][1,2,4]triazin-2-yl)-N-(2,6-difluorobenzyl)acetamide* (**6dd**). The product was prepared according to **6a** from **5d** (292 mg, 1.00 mmol) and 3 mL Ac_2_O. Reaction time was 24 h at 40–45 °C. Colorless solid, yield 331 mg (0.88 mmol, 88%), mp 292–295 °C. IR (ATR): *ṽ*_max_ = 3294, 1725, 1700, 1646, 1543, 1469, 1343, 1250, 1145, 991, 769, 670, 610, 471 cm^−1^. ^1^H-NMR (400 MHz, DMSO-*d_6_*): *δ* = 12.79 (br. s, 1H, NH), 10.00 (br. s, 1H, NH), 8.34 (d, ^1^*J*_C-H_ = 198.4 Hz, 1H, HC=N), 7.33 (tt, *J* = 6.9, 8.0 Hz, 1CH), 6.96 (t, *J* = 7.9 Hz, 2CH), 4.92 (br. s, 2H, CH_2_), 2.03 (s, 3H, Me), 1.80 (br. s, 3H, Me) ppm. ^13^C-NMR (101 MHz, DMSO-*d_6_*): *δ* = 169.5 (C=O), 168.2 (C=O), 161.4 (d, ^1^*J*_C-F_ = 249.1 Hz, Cq-F), 161.3 (d, ^1^*J*_C-F_ = 248.9 Hz, Cq-F), 146.7 (Cq), 143.4 (Cq), 132.6 (HC=N), 130.7 (t, ^3^*J*_C-F_ = 10.5 Hz, 1CH), 128.4 (Cq), 112.8 (Cq), 111.9 (t, ^2^*J*_C-F_ = 19.6 Hz, Cq), 111.6 (dd, ^2^*J*_C-F_ = 19.0 Hz, ^4^*J*_C-F_ = 5.9 Hz, 2CH), 37.2 (NCH_2_), 22.7 (Me), 22.4 (Me) ppm. MS: *m*/*z* (%) = 376 (25) [M^+^], 333 (50) [M—C_2_H_3_O]^+^, 127 (100) [C_7_H_5_F_2_]^+^. HRMS-ESI: calcd. for C16H14F2N6O3Na [M + Na]^+^: 399.0988; found: 399.0993.

*N-(7-Oxo-2-(phenylamino)-6,7-dihydropyrazolo[1,5-d][1,2,4]triazin-3-yl)acetamide* (**6e**). The product was prepared according to **6a** from **5e** (242 mg, 1.00 mmol) and 3 mL Ac_2_O. Reaction time was 24 h at 40–45 °C. Colorless solid, yield 256 mg (0.90 mmol, 90%), mp 297–299 °C. IR (ATR): *ṽ*_max_ = 3288, 3106, 2946, 1713, 1658, 1605, 1571, 1503, 1372, 1354, 1281, 1210, 1123, 1023, 896, 822, 744, 686, 622, 493, 435 cm^−1^. ^1^H-NMR (400 MHz, DMSO-*d_6_*): *δ* = 12.49 (br. s, 1H, NH), 9.68 (br. s, 1H, NH), 8.49 (br. s, 1H, NH), 8.20 (s, 1H, HC=N), 7.65 (d, *J* = 8.4, 2CH), 7.32 (dd, *J* = 8.4, 7.4 Hz, 2CH), 6.92 (t, *J* = 7.3, 1CH), 2.13 (d, ^1^*J*_C-H_ = 128.6 Hz, 3H, Me) ppm. ^13^C-NMR (101 MHz, DMSO-*d_6_*): *δ* = 169.1 (C=O), 149.6 (Cq), 143.8 (Cq), 141.2 (Cq), 130.6 (HC=N), 129.1 (2CH), 127.8 (Cq), 120.7 (1CH), 116.9 (2CH), 104.6 (Cq), 23.2 (Me) ppm. MS: *m*/*z* (%) = 284 (100) [M]^+^, 241 (25) [M—C_2_H_3_O]^+^. HRMS-ESI: *m*/*z* calcd. for C13H12N6O2Na [M + Na]^+^: 307.0914; found: 307.0917.

*N-(7-Oxo-2-(4-fluorophenylamino)-6,7-dihydropyrazolo[1,5-d][1,2,4]triazin-3-yl)acetamide* (**6f**). The product was prepared according to **6a** from **5f** (260 mg, 1.00 mmol) and 3 mL Ac_2_O. Reaction time was 8 h at 40–45 °C. Colorless solid, yield 284 mg (0.94 mmol, 94%), mp 312–314 °C. IR (ATR): *ṽ*_max_ = 3292, 3111, 2949, 1714, 1580, 1504, 1373, 1279, 128, 896, 828, 723, 644, 585, 446 cm^−1^. ^1^H-NMR (400 MHz, DMSO-*d_6_*): *δ =* 12.49 (br. s, 1H, NH), 9.65 (br. s, 1H, NH), 8.52 (br. s, 1H, NH), 8.19 (d, ^1^*J*_C-H_ = 198.0 Hz 1H, HC=N), 7.67 (dd, *J* = 8.6, 4.8 Hz, 2CH), 7.17 (t, *J* = 8.7 Hz, 2CH), 2.13 (d, ^1^*J*_C-H_ = 128.2 Hz, 3H, Me) ppm. ^13^C-NMR (101 MHz, DMSO-*d_6_*): *δ* = 169.2 (C=O), 156.7 (d, ^1^*J*_C-F_ = 236.6 Hz, Cq-F), 149.8 (Cq), 143.8 (Cq), 137.8 (d, ^4^*J*_C-F_ = 2.0 Hz, Cq-NH), 130.5 (CH=N), 128.0 (Cq), 118.4 (d, ^3^*J*_C-F_ = 8.3 Hz, 2CH), 115.6 (d, ^2^*J*_C-F_ = 22.2 Hz, 2CH), 104.4 (Cq), 23.2 (Me) ppm. MS: *m*/*z* (%) = 302 (100) [M^+^], 259 (60) [C_2_H_3_O]^+^, 94 (60) [C_6_H_4_F]^+^.

*N-(7-Oxo-2-(4-chlorophenylamino)-6,7-dihydropyrazolo[1,5-d][1,2,4]triazin-3-yl)acetamide* (**6g**). The product was prepared according to **6a** from **5g** (277 mg, 1.00 mmol) and 3 mL Ac_2_O. Reaction time was 24 h at 40–45 °C. Colorless solid, yield 312 mg (0.98 mmol, 98%), mp 328–331 °C. IR (ATR): *ṽ*_max_ = 3288, 3113, 2952, 1718, 1661, 1610, 1562, 1497, 1354, 1275, 1209, 1125, 896, 824, 724, 639, 574, 535, 502, 429 cm^−1^. ^1^H-NMR (400 MHz, DMSO-*d_6_*): *δ* = 12.52 (br. s, 1H, NH), 9.67 (br. s, 1H, NH), 8.66 (br. s, 1H, NH), 8.20 (d, ^1^*J*_C-H_ = 196.9 Hz, 1H, HC=N), 7.68 (d, *J* = 9.0 Hz, 2CH), 7.37 (d, *J* = 9.0 Hz, 2CH), 2.13 (s, 3H, Me) ppm. ^13^C-NMR (101 MHz, DMSO-*d_6_*): *δ* 169.2 (C=O), 149.4 (Cq), 143.7 (Cq), 140.2 (Cq), 130.5 (CH=N), 128.9 (2CH), 128.1 (Cq), 124.1 (C-Cl), 118.4 (2CH), 104.7 (Cq), 23.2 (Me) ppm. MS: *m*/*z* (%) = 318 (100) [M^+^], 275 (25) [M—C_2_H_3_O]^+^, 240 (20) [M—C_2_H_3_O—Cl]^+^.

*N-(7-Oxo-2-(2-chlorophenylamino)-6,7-dihydropyrazolo[1,5-d][1,2,4]triazin-3-yl)acetamide* (**6h**). The product was prepared according to **6a** from **5h** (277 mg, 1.00 mmol) and 3 mL Ac_2_O. Reaction time was 4 h at rt. Brownish solid, yield 284 mg (0.89 mmol, 89%) mp 309–311 °C. IR (ATR): *ṽ*_max_ = 3218, 1712, 16662, 1597, 1557, 1516, 1361, 1313, 1207, 1137, 1035, 905, 746, 723, 636, 532, 438 cm^−1^. ^1^H-NMR (400 MHz, DMSO-*d_6_*): *δ* = 12.60 (br. s, 1H, NH), 10.33 (br. s, 1H, NH), 8.35 (br. s, 1H, NH), 8.27 (s, 1H, HC=N), 8.23 (d, *J* = 8.1 Hz, 1CH), 7.46 (d, *J* = 8.0 Hz, 1CH), 7.33 (dd, *J* = 8.1, 7.6 Hz, 1CH), 6.96 (dd, *J* = 8.0, 7.6 Hz, 1CH), 2.16 (s, 3H, Me) ppm. ^13^C-NMR (101 MHz, DMSO-*d_6_*): *δ* = 169.8 (C=O), 148.5 (Cq), 143.6 (Cq), 137.7 (Cq), 129.8 (CH=N), 129.5 (CH), 128.0 (CH), 127.3 (Cq), 121.9 (CH), 120.7 (C-Cl), 118.1 (CH), 106.3 (Cq), 22.9 (Me) ppm. MS: *m*/*z* (%) = 318 (80) [M^+^], 283 (25) [M—Cl]^+^, 240 (100) [M—C_2_H_3_O—Cl]^+^, 127 (65) [M—C_6_H_4_NH—Cl]^+^.

*N-(7-Oxo-2-(2,4-dichlorophenylamino)-6,7-dihydropyrazolo[1,5-d][1,2,4]triazin-3-yl)acetamide* (**6i**). The product was prepared according to **6a** from **5i** (311 mg, 1.00 mmol) and 3 mL Ac_2_O. Reaction time was 3 h at rt. Yellowish solid, yield 314 mg (0.89 mmol, 89%) mp >340 °C. IR (ATR): *ṽ*_max_ = 3424, 1734 (CO), 1671 (CO), 1596 (NO_2_), 1551, 1524, 1369 (NO_2_), 1248, 1218, 1094, 1052, 820, 566 cm^−1^. ^1^H-NMR (400 MHz, DMSO-*d_6_*): *δ* = 12.63 (br. s, 1H, NH), 10.36 (br. s, 1H, NH), 8.52 (br. s, 1H, NH), 8.28 (s, 1H, HC=N), 8.24 (d, *J* = 8.8 Hz, 1CH), 7.61 (d, *J* = 2.3 Hz, 1CH), 7.42 (dd, *J* = 8.8, 2.3 Hz, 1CH), 2.16 (s, 3H, Me) ppm. ^13^C-NMR (101 MHz, DMSO-*d_6_*): *δ* = 170.0 (C=O), 148.1 (Cq), 143.6 (Cq), 136.9 (Cq), 129.8 (CH=N), 128.9 (CH), 128.1 (CH), 127.3 (Cq), 124.4 (C-Cl), 121.4 (C-Cl), 118.9 (CH), 106.5 (Cq), 22.9 (Me) ppm. MS: *m*/*z* (%) = 352 (100) [M^+^], 317 (10) [M—Cl]^+^, 309 (25) [M—Ac]^+^, 274 (27) [M—Cl—Ac]^+^.

*N-(2-((2-Methyl-3-(trifluoromethyl)phenyl)amino)-7-oxo-6,7-dihydropyrazolo[1,5-d][1,2,4]triazin-3-yl)acetamide* (**6j**). The product was prepared according to **6a** from **5j** (324 mg, 1.00 mmol) and 3 mL Ac_2_O. Reaction time was 6 h at 40–45 °C. Colorless solid, yield 352 mg (0.96 mmol, 96%) mp 313–315 °C. IR (ATR): *ṽ*_max_ = 3262, 3113, 2947, 1711, 1663, 1573, 1503, 1325, 1281, 1109, 1022, 9001, 791, 715, 637, 611, 540, 468, 426 cm^−1^. ^1^H-NMR (400 MHz, DMSO-*d_6_*): *δ* = 12.55 (br. s, 1H, NH), 10.10 (br. s, 1H, NH), 8.24 (s, 1H, HC=N), 8.15 (br. s, 1H, NH), 8.07 (d, *J* = 7.7 Hz, 1CH), 7.42–7.29 (m, 2CH), 2.35 (s, 3H, Me), 2.13 (s, 3H, Me) ppm. ^13^C-NMR (101 MHz, DMSO-*d_6_*): *δ* = 169.7 (C=O), 149.6 (Cq), 143.7 (Cq), 141.3 (Cq), 130.1 (CH), 128.5 (q, ^2^*J*_C-F_ = 28.5 Hz, Cq), 127.8 (Cq), 126.8 (CH), 125.3 (Cq), 124.8 (q, ^1^*J*_C-F_ = 274.3 Hz, CF_3_), 122.6 (CH), 118.9 (q, ^3^*J*_C-F_ = 5.8 Hz, CH), 106.0 (Cq), 23.0 (Me), 13.7 (q, ^4^*J*_C-F_ = 2.4 Hz, CH_3_) ppm. MS: *m*/*z* (%) = 366 (100) [M^+^], 323 (15) [M—C_2_H_3_O]^+^, 308 (50) [M—C_2_H_3_ONH]^+^.

*Ethyl 2-(2-morpholino-3-nitro-7-oxopyrazolo[1,5-d][1,2,4]triazin-6(7H)-yl)acetate* (**7b**). To a soln. of 266 mg (1.00 mmol) of triazinone **4b** and 200 mg (1.20 mmol) of ethyl 2-bromoacetate in 7 mL DMF, 121 mg (1.20 mmol) triethylamine at 10 °C was added, and the resulting mixture was kept off with stirring at rt for 8 h. After the addition of 70 mL cold water and 1 mL conc. hydrochloric acid, the precipitate was filtered off, washed with water (2 × 5 mL) and cold MeOH (3 mL), and was then dried in vacuo to give **7b** as a yellow solid, yield 335 mg (0.95 mmol, 95%) mp 130–131 °C. IR (KBr): *ṽ*_max_ = 2983, 2856, 1762 (CO), 1733 (CO), 1597 (NO_2_), 1515, 1442, 1388, 1312, 1204, 1116, 962, 820, 678 cm^−1^. ^1^H-NMR (400 MHz, CDCl_3_): *δ* = 8.71 (d, ^1^*J*_C-H_ = 204.4 Hz, 1H, HC=N), 4.96 (d, ^1^*J*_C-H_ = 144.4 Hz, 2H, NCH_2_), 4.27 (q, *J* = 7.1 Hz, 2H, OCH_2_), 3.91–3.84 (m, 4H, 2OCH_2_), 3.55–3.49 (m, 4H, 2NCH_2_), 1.30 (t, *J* = 7.1 Hz, 3H, Me) ppm. ^13^C-NMR (101 MHz, CDCl_3_): *δ =* 166.5 (C=O), 155.5 (C=O), 142.4 (Cq), 135.1 (Cq), 128.4 (CH=N), 119.5 (CNO_2_), 66.2 (2OCH_2_), 62.3 (OCH_2_), 53.4 (NCH_2_), 49.7 (2NCH_2_), 14.1 (Me) ppm. MS: *m*/*z* (%) = 352 (20) [M^+^] 334 (70) [M—OH]^+^, 306 (12) [M—NO_2_]^+^, 289 (58) [M—OH—NO_2_]^+^, 279 (12) [M—CO_2_Et]^+^.

*6-((6-Chloropyridin-3-yl)methyl)-2-((4-fluorophenyl)amino)-3-nitropyrazolo[1,5-d][1,2,4]triazin-7(6H)-one* (**7f**). The product was prepared according to **7b** from **4f** (290 mg, 1.00 mmol), 194 mg (1.20 mmol) 2-chloro-5-(chloromethyl)pyridine, 121 mg (1.20 mmol) triethylamine, and 7 mL DMF. Reaction time was 8 h at 75–80 °C. Orange solid, yield 391 mg (0.94 mmol, 94%) mp 205–206 °C. IR (KBr): *ṽ*_max_ = 3365, 3071, 1736 (CO), 1614, 1587 (NO_2_), 1511, 1377 (NO_2_), 1312, 1024, 843, 667, 518 cm^−1^. ^1^H-NMR (400 MHz, DMSO-*d_6_*): *δ* = 9.14 (br. s, 1H, NH), 8.78 (s, 1H, HC=N), 8.48 (d, *J* = 2.0 Hz, CH=N), 7.96–7.71 (m, 3CH), 7.53 (d, *J* = 8.1 Hz, 1CH), 7.23 (t, *J* = 8.1 Hz, 2CH), 5.41 (br. s, 2H, NCH_2_) ppm. ^13^C-NMR (101 MHz, DMSO-*d_6_*): *δ* = 158.2 (d, ^1^*J*_C-F_ = 239.7 Hz, Cq-F), 150.0 (C-Cl), 149.9 (CH=N), 149.3 (Cq), 142.7 (Cq), 140.0 (CH), 135.7 (d, ^4^*J*_C-F_ = 2.7 Hz, Cq-NH), 133.7 (Cq), 131.1 (Cq), 128.3 (CH=N), 124.4 (CH), 121.6 (d, ^3^*J*_C-F_ = 7.9 Hz, 2CH), 115.7 (C-NO_2_), 115.6 (d, ^2^*J*_C-F_ = 22.4 Hz, 2CH), 52.5 (NCH_2_) ppm. MS: *m*/*z* (%) = 415 (70) [M^+^], 167 (65), 126 (100) [C_6_H_5_ClN]^+^. HRMS-ESI: *m*/*z* calcd. for C17H11ClFN7O3Na [M + Na]^+^: 438.0488; found: 438.0492.

*Ethyl 2-(2-((2,4-dichlorophenyl)amino)-3-nitro-7-oxopyrazolo[1,5-d][1,2,4]triazin-6(7H)-yl)acetate* (**7i**). The product was prepared according to **7b** from **4i** (341 mg, 1.00 mmol), 200 mg (1.20 mmol) of ethyl 2-bromoacetate, 121 mg (1.20 mmol) of triethylamine, and 7 mL DMF. Reaction time was 10 h at rt. Yellow solid, yield 406 mg (0.95 mmol, 95%) mp 175–177 °C. IR (KBr): *ṽ*_max_ = 3623, 3333, 1730 (CO), 1610, 1595 (NO_2_), 1556, 1476, 1379 (NO_2_), 1241, 1018, 842, 766, 617 cm^−1^. ^1^H-NMR (400 MHz, DMSO-*d_6_*): *δ* = 9.21 (br. s, 1H, NH), 8.87 (d, ^1^*J*_C-H_ = 202.6 Hz, 1H, HC=N), 8.35 (d, *J* = 8.8 Hz, 1CH), 7.78 (d, *J* = 2.1 Hz, 1CH), 7.59 (dd, *J* = 8.8, 2.1 Hz, 1CH), 5.07 (s, 2H, NCH_2_), 4.21 (q, *J* = 7.1 Hz, 2H, OCH_2_), 1.23 (t, *J* = 7.1 Hz, 3H, Me) ppm. ^13^C-NMR (101 MHz, DMSO-*d_6_*): *δ* = 167.2 (C=O), 148.7 (Cq), 142.3 (Cq), 134.5 (Cq), 133.4 (Cq), 129.2 (CH), 128.6 (CH), 128.2 (CH), 127.5 (C-Cl), 123.3 (C-Cl), 121.0 (CH), 116.6 (C-NO_2_), 61.8 (OCH_2_), 54.0 (NCH_2_), 14.2 (Me) ppm. MS: *m*/*z* (%) = 426 (33) [M^+^], 390 (380) [M—HCl]^+^, 354 (8) [M—2HCl]^+^.

*Methyl 2-(2-((2,4-dichlorophenyl)amino)-3-nitro-7-oxopyrazolo[1,5-d][1,2,4]triazin-6(7H)-yl)acetate* (**7ii**). The product was prepared according to **7b** from **4i** (341 mg, 1.00 mmol), 184 mg (1.20 mmol) methyl 2-bromoacetate, 121 mg (1.20 mmol) triethylamine, and 7 mL DMF. Reaction time was 8 h at rt. Yellow solid, yield 359 mg (0.87 mmol, 87%) mp 201–202 °C. IR (ATR): *ṽ*_max_ = 3315, 1737, 1598, 1558, 1502, 1482, 1375, 1321, 1220, 1134, 1032, 983, 925, 829, 765, 666, 626, 556, 429 cm^−1^. ^1^H-NMR (400 MHz, DMSO-*d_6_*): *δ* = 9.22 (br. s, 1H, NH), 8.88 (d, ^1^*J*_C-H_ = 205.0 Hz, 1H, HC=N), 8.36 (d, *J* = 8.9 Hz, 1CH), 7.79 (d, *J* = 2.3 Hz, 1CH), 7.60 (dd, *J* = 8.9, 2.3 Hz, 1CH), 5.09 (d, ^1^*J*_C-H_ = 145.5 Hz, 2H, NCH_2_), 3.74 (d, ^1^*J*_C-H_ = 148.2 Hz, 3H, OMe) ppm. ^13^C-NMR (101 MHz, DMSO-*d_6_*): *δ* = 167.7 (C=O), 148.7 (Cq), 142.3 (Cq), 134.5 (Cq), 133.4 (Cq), 129.2 (CH), 128.6 (CH), 128.3 (CH), 127.5 (C-Cl), 123.4 (C-Cl), 121.1 (CH), 116.7 (C-NO_2_), 53.8 (NCH_2_), 52.8 (OMe) ppm. MS: *m*/*z* (%) = 412 (100) [M^+^], 378 (70) [M—Cl]^+^, 224 (10) [M—C_6_H_4_NHF—NO_2_—Cl]^+^.

*Ethyl 2-(3-amino-2-morpholino-7-oxopyrazolo[1,5-d][1,2,4]triazin-6(7H)-yl)acetate* (**8b**). The product was prepared according to **5a** from **7b** (352 mg, 1.00 mmol) and tin(II) chloride dihydrate (677 mg, 3.00 mmol) in EtOH. Reaction time was 8 h at 40–45 °C. Brownish solid, yield 248 mg (0.77 mmol, 77%) mp 211–213 °C. IR (ATR): *ṽ*_max_ = 3369, 2970, 1748, 1703, 15009, 1452, 1373, 1205, 1112, 1013, 953, 925, 902, 854, 755, 720, 573, 471, 441 cm^−1^. ^1^H-NMR (400 MHz, DMSO-*d_6_*): *δ* = 8.30 (d, ^1^*J*_C-H_ = 191.5 Hz, 1H, HC=N), 5.09 (br. s, 2H, NH_2_), 4.74 (d, ^1^*J*_C-H_ = 143.0 Hz, 2H, NCH_2_), 4.15 (q, *J* = 7.1 Hz, 2H, OCH_2_), 3.77–3.72 (m, 4H, 2OCH_2_), 3.21–3.16 (m, 4H, 2NCH_2_), 1.20 (t, *J* = 7.1 Hz, 3H, Me) ppm. ^13^C-NMR (101 MHz, DMSO-*d_6_*): *δ* = 168.5 (C=O), 153.5 (C=O), 144.1 (Cq), 130.7 (CH=N), 120.6 (Cq), 119.8 (Cq), 66.0 (2OCH_2_), 61.3 (OCH_2_), 52.1 (NCH_2_), 48.2 (2NCH_2_), 14.2 (Me) ppm. MS: *m*/*z* (%) = 322 (98) [M^+^], 306 (20) [M -NH_2_]^+^, 191 (100) [M—C_2_H_5-_– NC_4_H_8_O—NH_2_]^+^.

*3-Amino-6-((6-chloropyridin-3-yl)methyl)-2-((4-fluorophenyl)amino)pyrazolo[1,5-d][1,2,4]triazin-7(6H)-one* (**8f**). The product was prepared according to **5a** from **7f** (416 mg, 1.00 mmol) and tin(II) chloride dihydrate (677 mg, 3.00 mmol). Reaction time was 1 d at 65–70 °C. Brown solid, yield 339 mg (0.88 mmol, 88%) mp 206–208 °C. IR (KBr): *ṽ*_max_ = 3338, 1698 (CO), 1619, 1581 (NO_2_), 1508, 1385 (NO_2_), 1213, 1096, 826, 716, 586 cm^−1^. ^1^H-NMR (400 MHz, DMSO-*d_6_*): *δ* = 8.73 (br. s, 1H, NH), 8.41 (d, *J* = 1.9 Hz, 1H, HC=N), 8.29 (s, 1H, CH=N), 7.83 (dd, *J* = 8.2, 1.9 Hz, 1CH), 7.65 (dd, *J* = 8.4, 4.7 Hz, 2CH), 7.48 (d, *J* = 8.1 Hz, 1CH), 7.14 (t, *J* = 8.4 Hz, 2CH), 5.18 (br. s, 2H, NCH_2_), 4.07 (br. s, 2H, NH_2_) ppm. ^13^C-NMR (101 MHz, DMSO-*d_6_*): *δ* = 156.3 (d, ^1^*J*_C-F_ = 237.2 Hz, Cq-F), 149.6 (C-Cl), 149.5 (CH=N), 147.3 (Cq), 144.1 (Cq), 139.6 (CH), 138.1 (d, ^4^*J*_C-F_ = 1.9 Hz, Cq-NH), 132.8 (Cq), 130.7 (CH=N), 124.4 (CH), 118.8 (Cq), 117.7 (d, ^3^*J*_C-F_ = 7.5 Hz, 2CH), 116.6 (Cq), 115.3 (d, ^2^*J*_C-F_ = 22.4 Hz, 2CH), 50.5 (NCH_2_) ppm. MS: *m*/*z* (%) = 385 (7) [M^+^], 315 (5) [M—Cl—F—NH_2_]^+^, 243 (5) [M—NH_2_—C_6_H_5_ClN]^+^, 126 (40) [C_6_H_5_ClN]^+^.

*Ethyl 2-(3-amino-2-((2,4-dichlorophenyl)amino)-7-oxopyrazolo[1,5-d][1,2,4]triazin-6(7H)-yl)acetate* (**8i**). The product was prepared according to **5a** from **7i** (427 mg, 1.00 mmol) and tin(II) chloride dihydrate (677 mg, 3.00 mmol) in EtOH. Reaction time was 16 h at 65–70 °C. Beige solid, yield 334 mg (0.84 mmol, 84%), mp 275–278 °C. IR (ATR): *ṽ*_max_ = 3385, 3251, 1732, 1675, 1544, 1474, 1381, 1216, 1102, 1014, 813, 710, 643, 573, 479, 426 cm^−1^. ^1^H-NMR (400 MHz, DMSO-*d_6_*): *δ* = 8.33 (d, ^1^*J*_C-H_ = 192.1 Hz, 1H, HC=N), 8.01 (d, *J* = 9.0 Hz, 1CH), 7.83 (br. s, 1H, NH), 7.61 (d, *J* = 2.3 Hz, 1CH), 7.39 (dd, *J* = 9.0, 2.3 Hz, 1CH), 5.41 (br. s, 2H, NH_2_), 4.77 (d, ^1^*J*_C-H_ = 143.0 Hz, 2H, NCH_2_), 4.16 (q, *J* = 7.1 Hz, 2H, OCH_2_), 1.21 (t, *J* = 7.1 Hz, 3H, Me) ppm. ^13^C-NMR (101 MHz, DMSO-*d_6_*): *δ* = 168.4 (C=O), 146.4 (Cq), 144.0 (Cq), 137.3 (Cq), 130.6 (CH), 129.0 (CH), 128.0 (CH), 124.7 (C-Cl), 122.1 (C-Cl), 120.0 (CH), 119.5 (Cq), 119.4 (Cq), 61.3 (OCH_2_), 52.2 (NCH_2_), 14.2 (Me) ppm. MS: *m/z* (%) = 396 (100) [M^+^], 360 (25) [M—Cl]^+^, 324 (35) [M—2 Cl]^+^. HRMS-ESI: *m*/*z* calcd. for C15H14Cl2N6O3Na [M + Na]^+^: 419.0397; found: 319.0404.

*Methyl 2-(3-amino-2-((2,4-dichlorophenyl)amino)-7-oxopyrazolo[1,5-d][1,2,4]triazin-6(7H)-yl)acetate* (**8ii**). The product was prepared according to **5a** from **7ii** (414 mg, 1.00 mmol) and tin(II) chloride dihydrate (677 mg, 3.00 mmol) in MeOH. Reaction time was 18 h at 65–70 °C. Light brown solid, yield 261 mg (0.68 mmol, 68%) mp 273–275 °C. IR (KBr): *ṽ*_max_ = 3391, 3254, 1742 (CO), 1677 (CO), 1548 (NO_2_), 1475, 1392 (NO_2_), 1228, 1040, 813, 714, 552 cm^−1^. ^1^H-NMR (400 MHz, DMSO-*d_6_*): *δ* = 8.34 (s, 1H, HC=N), 8.00 (d, *J* = 8.9 Hz, 1CH), 7.85 (br. s, 1H, NH), 7.61 (d, *J* = 2.4 Hz, 1CH), 7.39 (dd, *J* = 8.9, 2.4 Hz, 1CH), 5.44 (br. s, 2H, NH_2_), 4.79 (s, 2H, NCH_2_), 3.69 (s, 3H, OCH_3_) ppm. ^13^C-NMR (101 MHz, DMSO-*d_6_*): *δ* = 169.0 (C=O), 146.5 (Cq), 144.0 (Cq), 137.3 (Cq), 130.7 (CH), 129.0 (CH), 128.0 (CH), 124.8 (C-Cl), 122.2 (C-Cl), 122.1 (Cq), 120.0 (CH), 119.5 (Cq), 52.5 (OCH_3_), 52.1 (NCH_2_) ppm. MS: *m*/*z* (%) = 382 (90) [M]^+^, 347 (40) [M—Cl]^+^, 323 (45) [M—CO_2_Me]^+^.

*Ethyl 2-(3-acetamido-2-morpholino-7-oxopyrazolo[1,5-d][1,2,4]triazin-6(7H)-yl)acetate* (**9b**). The product was prepared according to **6a** from **8b** (322 mg, 1.00 mmol) and 3 mL Ac_2_O. Reaction time was 96 h at rt. Colorless solid, yield 346 mg (0.95 mmol, 95%) mp 204–205 °C. IR (ATR): *ṽ*_max_ = 3297, 2954, 2863, 1747, 1698, 1488, 1353, 1213, 1109, 1019, 960, 928, 765, 740, 6001, 546, 523 cm^−1^. ^1^H-NMR (400 MHz, DMSO-*d_6_*): *δ* = 9.77 (br. s, 1H, NH), 8.17 (d, ^1^*J*_C-H_ = 197.3 Hz, 1H, HC=N), 4.86 (d, ^1^*J*_C-H_ = 143.6 Hz, 2H, NCH_2_), 4.17 (q, *J* = 7.1 Hz, 2H, OCH_2_), 3.79–3.67 (m, 4H, 2OCH_2_), 3.32–3.23 (m, 4H, 2NCH_2_), 2.08 (d, ^1^*J*_C-H_ = 128.2 Hz, 3H, CH_3_), 1.21 (t, *J* = 7.1 Hz, 3H, Me) ppm. ^13^C-NMR (101 MHz, DMSO-*d_6_*): *δ* = 169.3 (C=O), 168.1 (C=O), 156.4 (C=O), 143.5 (Cq), 131.1 (Cq), 130.4 (CH=N), 105.3 (Cq), 65.8 (2OCH_2_), 61.5 (OCH_2_), 52.6 (NCH_2_), 47.8 (2NCH_2_), 22.9 (Me), 14.2 (Me) ppm. MS: *m*/*z* (%) = 364 (99) [M^+^], 321 (65) [M—C_2_H_3_O]^+^, 190 (100) [M—C_2_H_5_O—NC_4_H_8_O—C_2_H_3_O]^+^.

*N-(6-((6-Chloropyridin-3-yl)methyl)-2-((4-fluorophenyl)amino)-7-oxo-6,7-dihydropyrazolo[1,5-d][1,2,4]triazin-3-yl)acetamide* (**9f**). The product was prepared according to **6a** from **8f** (386 mg, 1.00 mmol) and 3 mL Ac_2_O. Reaction time was 8 h at 40–45 °C. Colorless solid, yield 394 mg (0.92 mmol, 92%) mp 274–276 °C. IR (ATR): *ṽ*_max_ = 3271, 1718, 1657, 1618, 1582, 1505, 1360, 1211, 116, 826, 745, 722, 664, 511 cm^−1^. ^1^H-NMR (400 MHz, DMSO-*d_6_*): *δ =* 9.71 (br. s, 1H, NH), 8.55 (br. s, 1H, NH), 8.45 (d, *J* = 2.3 Hz, 1H, HC=N), 8.29 (s, 1H, CH=N), 7.85 (dd, *J* = 8.3, 2.3 Hz, 1CH), 7.66 (dd, *J* = 9.0, 4.8 Hz, 2CH), 7.50 (d, *J* = 8.3 Hz, 1CH), 7.17 (t, *J* = 9.0 Hz, 2CH), 5.27 (d, ^1^*J*_C-H_ = 142.6 Hz, 2H, NCH_2_), 2.13 (d, ^1^*J*_C-H_ = 128.3 Hz, 3H, CH_3_) ppm. ^13^C-NMR (101 MHz, DMSO-*d_6_*): *δ* = 169.0 (C=O), 156.8 (d, ^1^*J*_C-F_ = 236.5 Hz, Cq-F), 149.85 (Cq), 149.75 (Cq), 149.7 (CH=N), 143.6 (Cq), 139.7 (CH), 137.7 (d, ^4^*J*_C-F_ = 2.2 Hz, Cq-NH), 132.2 (Cq), 130.9 (CH=N), 127.5 (Cq), 124.4 (CH), 118.4 (d, ^3^*J*_C-F_ = 7.5 Hz, 2CH), 115.6 (d, ^2^*J*_C-F_ = 22.2 Hz, 2CH), 105.0 (Cq), 51.0 (NCH_2_), 23.2 (Me) ppm. MS: *m*/*z* (%) = 427 (100) [M]^+^, 385 (15) [M -C_2_H_3_O]^+^, 126 (75) [C_5_NH_3_CH_2_Cl]^+^. HRMS-ESI: *m*/*z* calcd. for C19H15ClFN7O2Na [M + Na]^+^: 450.0852; found: 450.0853.

*Ethyl 2-(3-acetamido-2-((2,4-dichlorophenyl)amino)-7-oxopyrazolo[1,5-d][1,2,4]triazin-6(7H)-yl)acetate* (**9i**). The product was prepared according to **6a** from **8i** (397 mg, 1.00 mmol) and 3 mL Ac_2_O. Reaction time was 1 d at rt. Beige solid, yield 426 mg (0.97 mmol, 97%) mp 280–282 °C. IR (ATR): *ṽ*_max_ = 3251, 3009, 1730, 1656, 1593, 1548, 1355, 1213, 1144, 1016, 817, 730, 621, 601, 538, 434 cm^−1^. ^1^H-NMR (400 MHz, DMSO-*d_6_*): *δ* = 10.39 (br. s, 1H, NH), 8.54 (br. s, 1H, NH), 8.37 (s, 1H, HC=N), 8.18 (d, *J* = 9.0 Hz, 1CH), 7.62 (br. s, 1CH), 7.43 (d, *J* = 9.0 Hz, 1CH), 4.88 (d, ^1^*J*_C-H_ = 145.0 Hz, 2H, NCH_2_), 4.18 (q, *J* = 7.3 Hz, 2H, OCH_2_), 2.17 (s, 3H, Me), 1.22 (t, *J* = 7.3 Hz, 3H, Me) ppm. ^13^C-NMR (101 MHz, DMSO-*d_6_*): *δ* = 169.9 (C=O), 168.1 (C=O), 148.7 (Cq), 143.3 (Cq), 136.8 (Cq), 130.0 (CH), 128.9 (CH), 128.1 (CH), 126.9 (Cq), 124.8 (C-Cl), 121.8 (C-Cl), 119.2 (CH), 107.3 (Cq), 61.5 (OCH_2_), 52.7 (NCH_2_), 22.9 (Me), 14.2 (Me) ppm. MS: (%) = 439 (100) [M^+^], 360 (75) [M—C_2_H_3_O—Cl]^+^.

*Methyl 2-(3-acetamido-2-((2,4-dichlorophenyl)amino)-7-oxopyrazolo[1,5-d][1,2,4]triazin-6(7H)-yl)acetate* (**9ii**). The product was prepared according to **6a** from **8ii** (383 mg, 1.00 mmol) and 3 mL Ac_2_O. Reaction time was 6 h at rt. Colorless solid, yield 395 mg (0.93 mmol, 93%) mp 289–292 °C. IR (KBr): *ṽ*_max_ = 3251, 1733 (CO), 1657, 1596 (NO_2_), 1551, 1354 (NO_2_), 1225, 1144, 1041, 820, 729, 661 cm^−1^. ^1^H-NMR (400 MHz, DMSO-*d_6_*): *δ* = 10.40 (br. s, 1H, NH), 8.54 (br. s, 1H, NH), 8.37 (s, 1H, HC=N), 8.18 (d, *J* = 9.0 Hz, 1CH), 7.62 (d, *J* = 2.5 Hz, 1CH), 7.43 (dd, *J* = 9.0, 2.5 Hz, 1CH), 4.91 (s, 2H, NCH_2_), 3.72 (s, 3H, OCH_3_), 2.17 (s, 3H, Me) ppm. ^13^C-NMR (101 MHz, DMSO-*d_6_*): *δ* = 169.9 (C=O), 168.6 (C=O), 148.7 (Cq), 143.3 (Cq), 136.8 (Cq), 130.1 (CH), 128.9 (CH), 128.1 (CH), 127.0 (Cq), 124.8 (C-Cl), 121.8 (C-Cl), 119.2 (CH), 107.3 (Cq), 52.7 (NCH_2_), 52.6 (OCH_3_), 22.9 (Me) ppm. MS: *m*/*z* (%) = 424 (100) [M^+^], 388 (18) [M—HCl]^+^, 381 (15) [M—Ac]^+^, 365 (8) [M—CO_2_Me]^+^_,_ 346 (80) [M—HCl—Ac]^+^.

#### 3.1.3. Crystal Data

X-Ray structure analysis for ethyl 2-(2-morpholino-3-nitro-7-oxopyrazolo[1,5-*d*][1,2,4]triazin-6(7*H*)-yl) acetate C_13_H_16_N_6_O_6_ (**7b**), M = 352.32 g mol^−1^**:** A suitable single crystal of the title compound was recrystallized from a mixture of acetone:methanol=1:1, selected under a polarization microscope, and mounted in a glass capillary (*d* = 0.3 mm). The crystal structure was determined by X-Ray diffraction analysis using graphite monochromated Mo-*K*_α_ radiation (0.71073 Å) [T = 223(2) K], whereas the scattering intensities were collected with a single-crystal diffractometer (STOE IPDS II). The crystal structure was solved by Direct Methods using SHELXS-97 and refined using alternating cycles of least-squares refinement against *F^2^* (SHELXL-97) [18]. All non-H atoms were located in Difference Fourier maps and were refined with anisotropic displacement parameters. The H positions were determined by a final Difference Fourier Synthesis.

C_13_H_16_N_6_O_6_ crystallized in the monoclinic space group *P*2_1_/*n* (no. 14), lattice parameters *a* = 11.184(2) Å, *b* = 5.2197(6) Å, *c* = 27.765(6) Å, *β* = 97.84(2) °, *V* = 1605.6(5) Å^3^, *Z* = 4, *d_calc._* = 1.457 g cm^−3^, F(000) = 736 using 2730 independent reflections and 272 parameters. *R*1 = 0.0753, *wR*2 = 0.1595 [*I* > 2*σ*(*I*)], goodness of fit on *F^2^* = 0.942, residual electron density = 0.378 and −0.389 e Å^−3^.

Further details of the crystal structure investigations have been deposited with the Cambridge Crystallographic Data Center, CCDC 2432103. Copies of this information may be obtained free of charge from The Director, CCDC, 12 Union Road, Cambridge, CB2 1EZ, UK (Fax: +44(1223)-336 033; e-mail: fileserv@ccdc.ac.uk or http://www.ccdc.cam.ac.uk).

### 3.2. Microbiological Investigations

Test organisms were the Gram-negative bacteria *Escherichia coli* ATCC 25922, the respective delta TolC mutant and *Klebsiella pneumniae* DSM681, the Gram-positive bacteria *Staphylococcus aureus* USA300, and the murine fibroblast cell line L929.

Bacteria were cultivated in suitable media: For *E. coli* strains, the LB medium was used, while for *K. pneumonia* and *S. aureus,* the TSB medium was used. Overnight cultures were inoculated from agar plates and incubated with shaking at 37 °C and 150 rpm (Thermo Scientific™ MaxQ™ 4000) (Fisher Scientific GmbH, Schwerte, Germany). The next day, the overnight culture was diluted to approximately 1:20 to result in an OD600 of 0.1. This preculture was allowed to grow until OD600 = 0.5 and was then diluted again with fresh medium to an OD600 = 0.02 (working culture).

For primary screening, 60 µL of the working culture was placed in each well of the 96-well half-area plate (Costar 3697). Then, 1.5 µL of 2 mM solutions (PBS + 20%DMSO) of the test compounds were added, resulting in approximately 50 µM final compound concentrations.

For dose–response studies, different volumes of the 10 mM DMSO stock solutions were added to the assay plate with the acoustic dispenser Echo 550 (Beckman Coulter), and 60 µL of the bacterial working culture was added directly into each well of the compound plates.

The plates were incubated at 37 °C for 22–24 h. The optical density at 600 nm (OD600) was determined using a microtiter plate spectrophotometer (Agilent BioTek Epoch 2) (Agilent Technologies GmbH, Waldbronn, Germany). The values were normalized with respect to growth in control wells without compounds.

For cytotoxicity studies, L929 cells were cultivated in DMEM supplemented with 10% serum (FBS). Two thousand cells were seeded per well of a 96-well half-area microtiter plate (Costar 3697) in 60 µL medium and allowed to adhere overnight. For primary screening, 1.5 µL of the 2 mM compound solutions (PBS + 20% serum) was added. For dose–response studies, the cells were seeded directly into the compound plates, prepared as described above with the Echo550. Cells were incubated with the compounds at 37 °C and 5% CO_2_ in a cell culture incubator (Thermo Scientific™ Heracell™ VIOS 160i “CO2” incubator) (Fisher Scientific GmbH, Schwerte, Germany) for 72 h. For the determination of the viability of the cells, 5 µL of the resazurin stock solution (500 µM) was added and incubated for 3 h. Fluorescence was determined using an excitation wavelength of 540 nm and an emission wavelength of 600 nm of a fluorescence microtiter plate reader (Tecan Spark) (Tecan Deutschland GmbH, Crailsheim, Germany).

## 4. Conclusions

In summary, we have developed a new three-step route for the formation of a 3-nitro-substituted pyrazolo[1,5-*d*][1,2,4]triazin-7(6*H*)-one cycle **4** starting from 2-nitroperchlorobutadiene (**1**) and synthesized a series of corresponding amino, acetamido, and 6-*N*-alkyl derivatives. We investigated the antibacterial and cytotoxic activities of 61 synthesized compounds. Hereby, we found that most of them were not active against any of the organisms used. However, it cannot be completely excluded that these data were influenced by the limited solubility of the compounds in the mostly aqueous solutions, which are needed for biological assays.

## Figures and Tables

**Table 1:** Yields of compounds **2–4**.

Amines HNRR^1^	Yields, %		
	Compds. 2a–j	Compds. 3a–j	Compds. 4a–j
Methylamine	55 (**2a**)	67 (**3a**, Alk = Me)	94 (**4a**)
Morpholine	86 (**2b**)	84 (**3b**, Alk = Me)	90 (**4b**)
2-(4-Chlorophenoxy)-ethan-1-amine	88 (**2c**)	65 (**3c**, Alk = Me)	67 (**4c**)
(2,6-Difluorophenyl)-methanamine	94 (**2d**)	85 (**3d**, Alk = Me)	86 (**4d**)
Aniline	88 (**2e**)	74 (**3e**, Alk = Me) 60 (**3ee**, Alk = Et)	93 (**4e** from **3e**) 88 (**4e** from **3ee**)
4-Fluoroaniline	86 (**2f**)	52 (**3f**, Alk = Me) 55 (**3ff**, Alk = Et)	95 (**4f** from **3f**) 88 (**4f** from **3ff**)
4-Chloroaniline	95 (**2g**)	90 (**3g**, Alk = Me) 80 (**3gg**, Alk = Et)	85 (**4g** from **3g**) 83 (**4g** from **3gg**)
2-Chloroaniline	95 (**2h**)	88 (**3h**, Alk = Me)	74 (**4h**)
2,4-Dichloroaniline	86 (**2i**)	93 (**3i**, Alk = Me)	85 (**4i**)
2-Methyl-3-(trifluoromethyl)aniline	85 (**2j**)	78 (**3j**, Alk = Me)	42 (**4j**)

**Table 2:** ^1^H and ^13^C-NMR shifts and ^1^J^13^_C-H_-couplings constants of the CH=N group.

	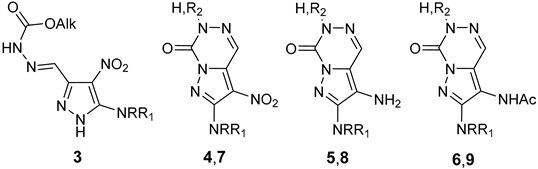			
Compounds CH=N	Pyrazoles 3	Nitro-Triazinones 4/7	Amino-Triazinones 5/8	Amides 6/9
^1^H shifts, ppm	8.48 ± 0.03	**4**: 8.65 ± 0.05 **7**: 8.81 ± 0.03	**5**: 8.19 ± 0.12 **8**: 8.31 ± 0.05	**6**: 8.23 ± 0.15 **9**: 8.30 ± 0.15
^13^C-shifts, ppm	131.1 ± 0.5	**4**: 128.4 ± 0.3 **7**: 128.7 ± 0.2	**5**: 130.2 ± 0.3 **8**: 130.7 ± 0.2	**6**: 130.5 ± 0.2 **9**: 130.4 ± 0.3
^1^*J*^13^_C-H_-coupl. constants, Hz	175.7 ± 1.2	**4**: 203.4 ± 0.8 **7**: 204.0 ± 0.8	**5**: 191.6 ± 0.8 **8**: 191.8 ± 0.5	**6**: 197.1 ± 0.8 **9**: 197.3 ± 0.5

## Data Availability

The original contributions presented in this study are included in the article/Supplementary Material. Further inquiries can be directed to the corresponding author.

## Supplementary Materials

The following supporting information can be downloaded at https://www.mdpi.com/article/10.3390/molecules30183792/s1; Figures S1–S121: ^1^H–NMR and ^13^C–NMR; Figures S122–S130: HRMS; Table S1: list of primary screening data and IC_50_ values for all tested organisms.

## References

[1] Kaberdin R.V., Potkin V.I., Zapol’skii V.A. (1997). Nitrobutadienes and their halogen derivatives: Synthesis and reactions. Russ. Chem. Rev..

[2] Zapol’skii V.A., Bilitewski U., Kupiec S.R., Ramming I., Kaufmann D.E. (2020). Polyhalonitrobutadienes as Versatile Building Blocks for the Biotargeted Synthesis of Substituted N-Heterocyclic Compounds. Molecules.

[3] SciFinder®—A Database of Chemical and Bibliographic Information, a Product of CAS—Columbus, Ohio, United States. https://scifinder.cas.org.

[4] Baraldi P.G., Cacciari B., Romagnoli R., Spalluto G. (1999). 1*H*-pyrazolo [2,3-*d*][1,2,4]triazine-3,7-diones as a new class of human leukocyte elastase inhibitors. Arzneim. Forsch..

[5] Walensky L.D. (2013). Pyrazol-3-Ones that Activate Pro-Apoptotic. BAX. Patent.

[6] Oltvai Z.N., Milliman C.L., Korsmeyer S.J. (1993). Bcl-2 heterodimerizes in vivo with a conserved homolog, Bax, that accelerates programed cell death. Cell.

[7] Padwa A., Woolhouse A.D., Blount J.J. (1983). 1,3-Dipolar cycloaddition reactions of diazopyrazolinones with electron-deficient dipolarophiles. J. Org. Chem..

[8] Zhang Z., Wang X., Tao S., Zhu S. (2012). 1,3-Dipolar cycloaddition reaction of 3-trifluoromethyl-4-diazopyrazolinones with acetylenedicarboxylates. Tetrahedron.

[9] Zapol’skii V.A., Namyslo J.C., Gjikaj M., Kaufmann D.E. (2010). Chemistry of Polyhalogenated Nitrobutadienes, Part 9: Acyclic and Heterocyclic Nitroenamines and Nitroimines from 2-Nitroperchlorobuta-1,3-diene. Z. Fur Naturforschung Sect. B A J. Chem. Sci..

[10] Zapol’skii V.A., Namyslo J.C., Gjikaj M., Kaufmann D.E. (2014). Efficient synthesis of functionalized (*Z*)-2-allylidenethiazolidin-4-ones. Beilstein J. Org. Chem..

[11] Bürgi M., Zapol’skii V.A., Hinkelmann B., Köster M., Kaufmann D.E., Sasse F., Hauser H., Etcheverrigaray M., Kratje R., Bollati-Fogolín M. (2016). Screening and characterization of molecules that modulate the biological activity of IFNs-I. J. Biotechnol..

[12] https://www.periodensystem-online.de/index.php?sel=wertdesc&prop=pKb-Werte&show=list&el=92&id=-acid.

[13] Spitale R.C., Volpini R., Heller M.G., Krucinska J., Cristalli G., Wedekind J.E. (2009). Identification of an imino group indispensable for cleavage by a small ribozyme. J. Am. Chem. Soc..

[14] Potkin V.I., Zapol’skii V.A., Kaberdin R.V. (1996). Nitration of 2-*H*-pentachloro-1,3-butadiene. Dokl. Natl. Acad. Sci. Belarus.

[15] Ol’dekop Y.A., Kaberdin R.V., Potkin V.I. (1979). Reaction of 2-nitropentachloro-1,3-butadiene with secondary amines. J. Org. Chem. USSR Engl. Transl..

[16] Ol’dekop Y.A., Kaberdin R.V., Potkin V.I., Shingel I.A. (1979). Reaction of 2-nitropentachloro-1,3-butadiene with primary amines. J. Org. Chem. USSR Engl. Transl..

[17] Zapol’skii V.A., Bürgi M., Oggero M., Bollati-Fogolín M., Kaufmann D.E. (2023). Synthesis of new types of compounds modulating the biological activity of Type I Interferons (IFN-I). Arkivoc.

[18] Sheldrick G.M. (2015). Crystal structure refinement with SHELXL. Acta Crystallogr..

