# Supplementary material for: Synthesis and Microbiological Activities of 3-Nitropyrazolo-[1,5-d][1,2,4]triazin-7(6H)-ones and Derivatives
Source: Molecules. 2025 Sep 18;30(18):3792. doi: 10.3390/molecules30183792 (PMC12473042; doi:10.3390/molecules30183792)
Supplement: Supplementary file 1 [file molecules-30-03792-s001.zip › molecules-3826305-supplementary.pdf]

Supplementary Material

Contents

1. Copies of ¹H-NMR and ¹³C-NMR spectra for newly synthesized compounds (Figures S1 – S121)……………..……………………………………………………………………S1
2. Copies of HR-MS spectra for newly synthesized compounds (Figures S122 – S130)………………………………………...……………………….......S123
3. Primary screening data of all tested compounds and IC_{50}-values of those compounds, which showed a growth inhibitory effect > 50% in the primary screening (Table S1)…………..S132

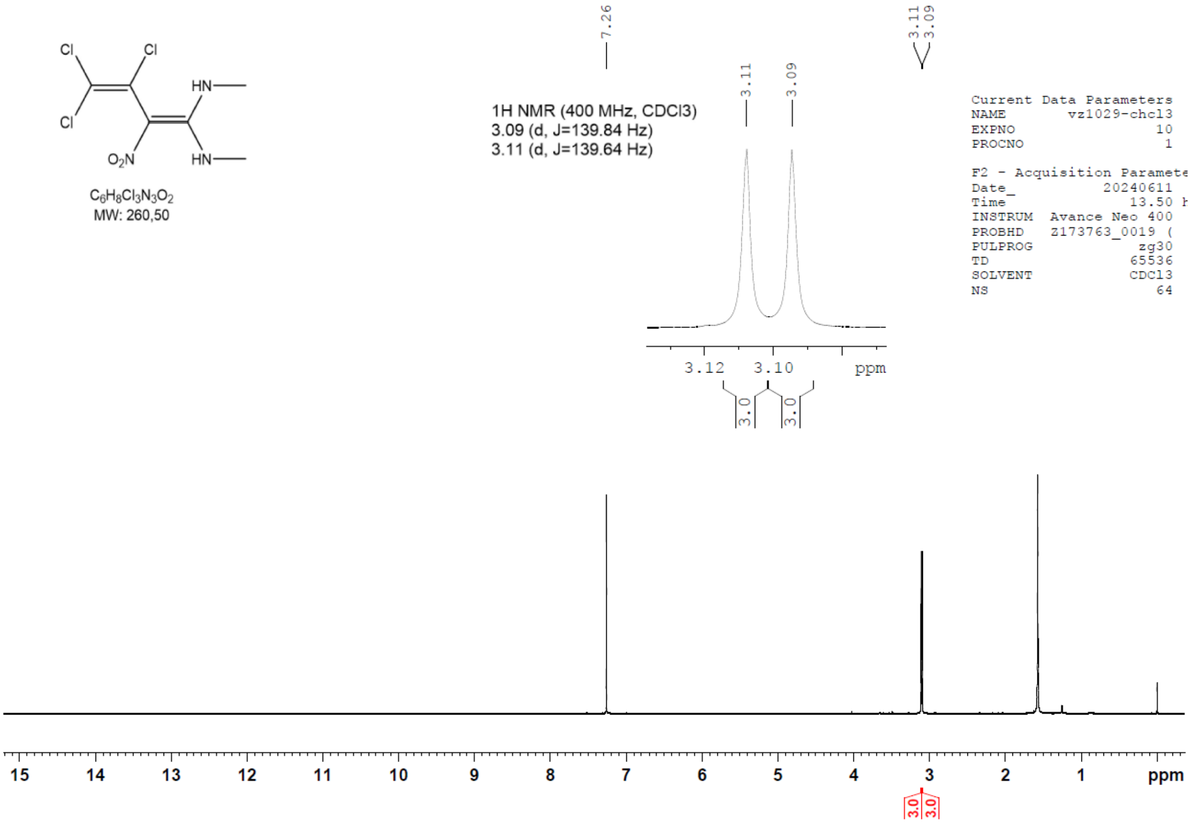

Figure S1. ¹H NMR spectrum of compound **2a** in $CDCl_3$

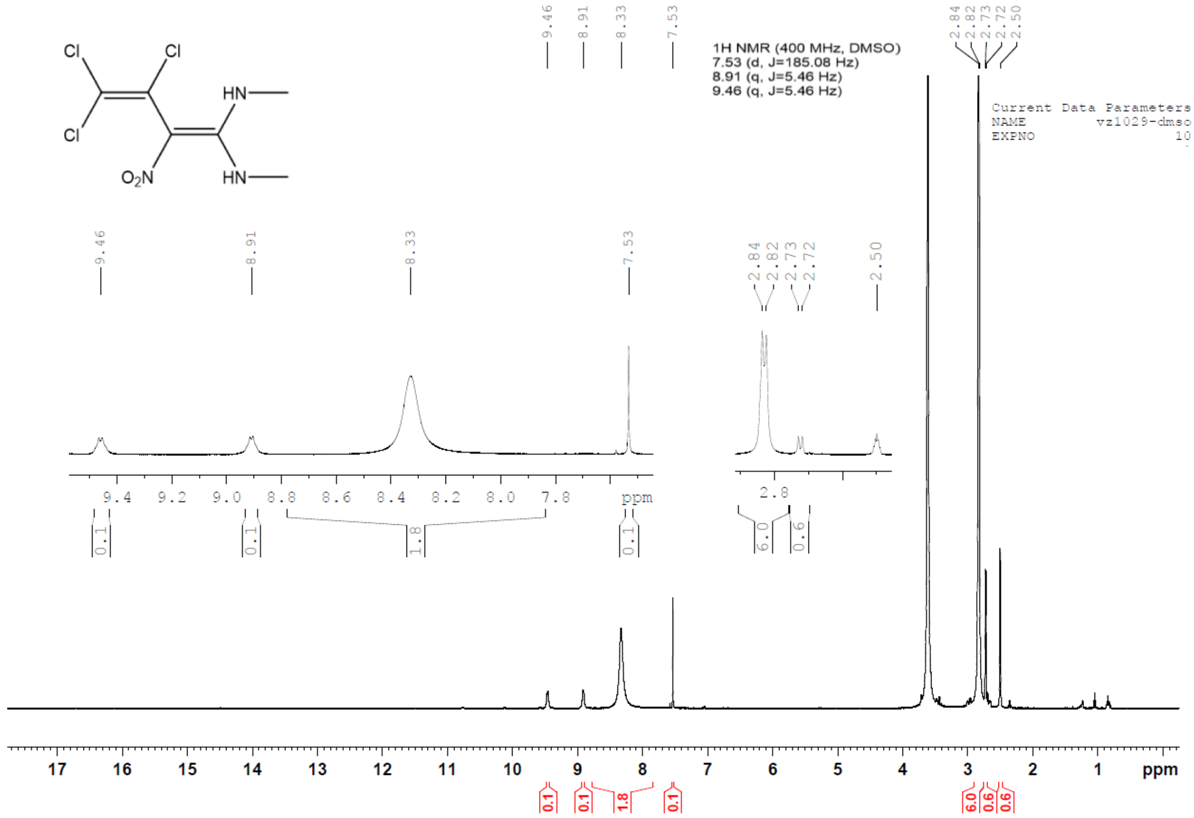

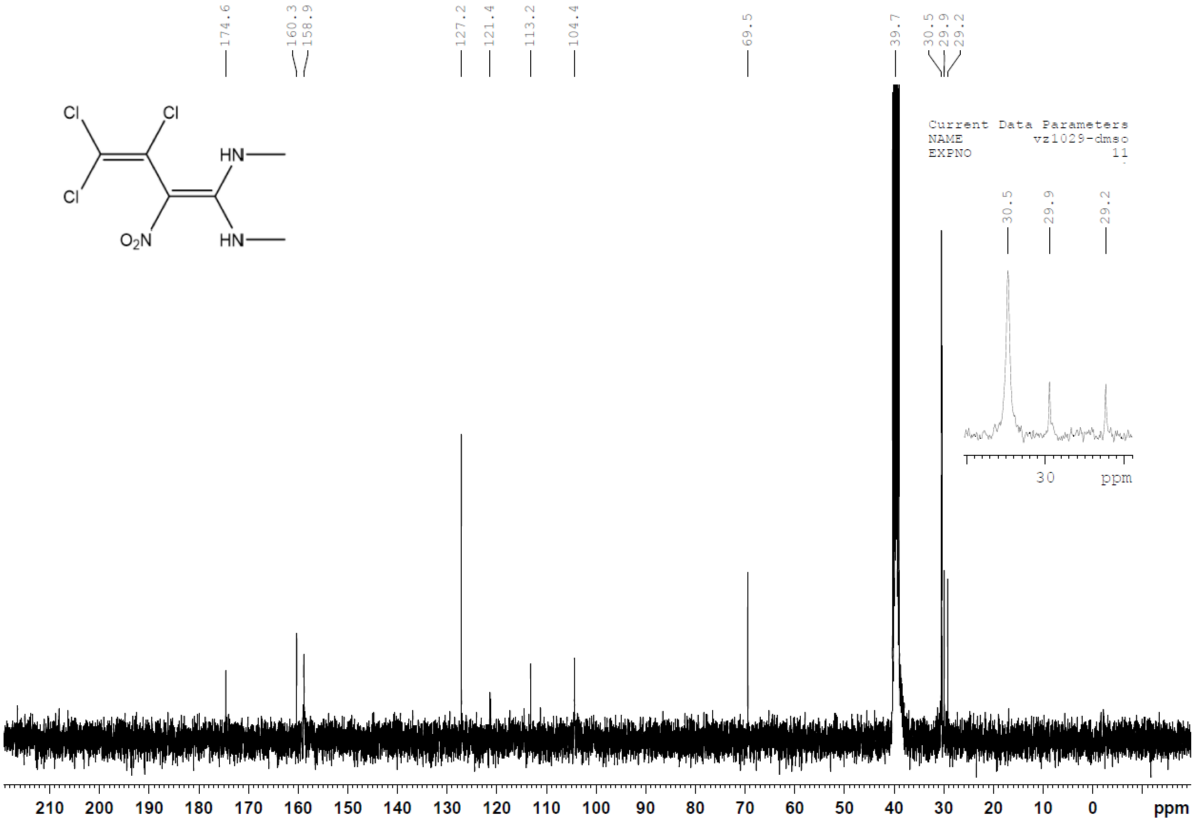

Figure S2-S3. ¹H and 13C NMR spectra of compound **2a** in DMSO-d₆

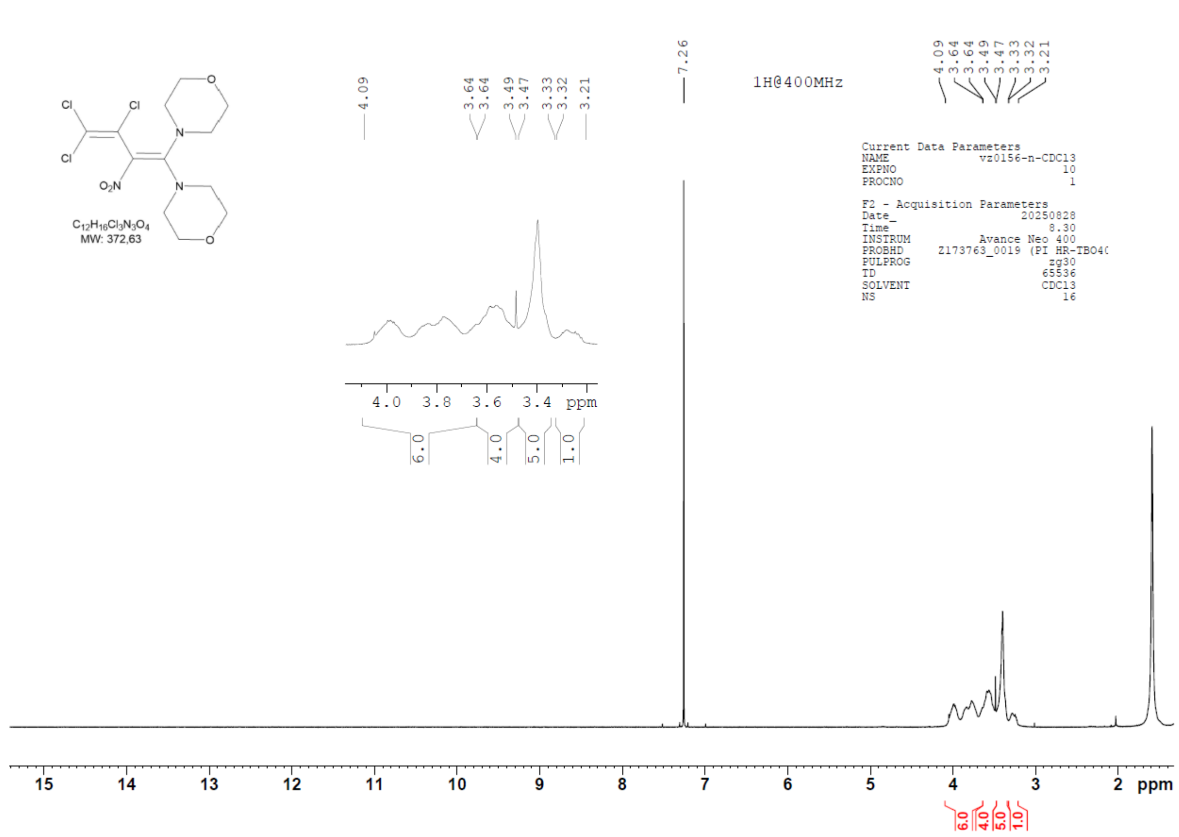

Figure S4. ^{1}H NMR spectrum of compound **2b** in $CDCl_3$

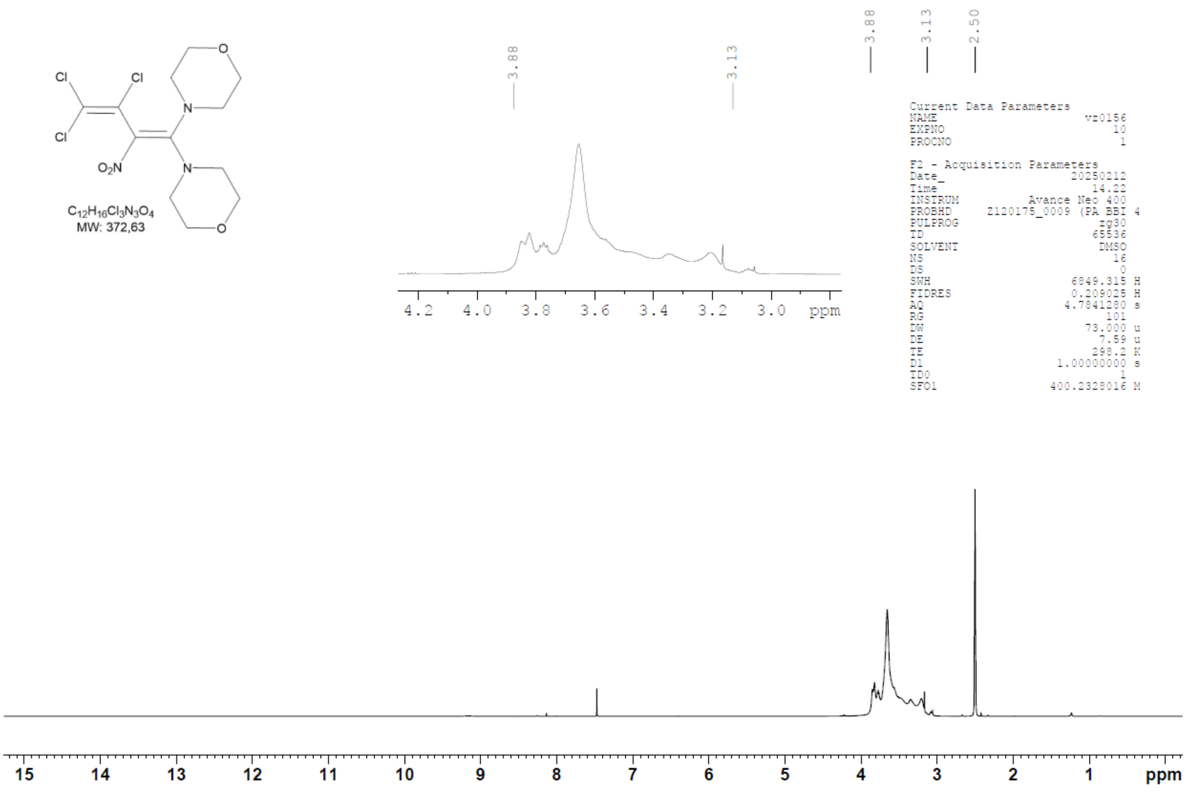

Figure S5. ^{1}H NMR spectrum of compound **2b** in DMSO-d_6

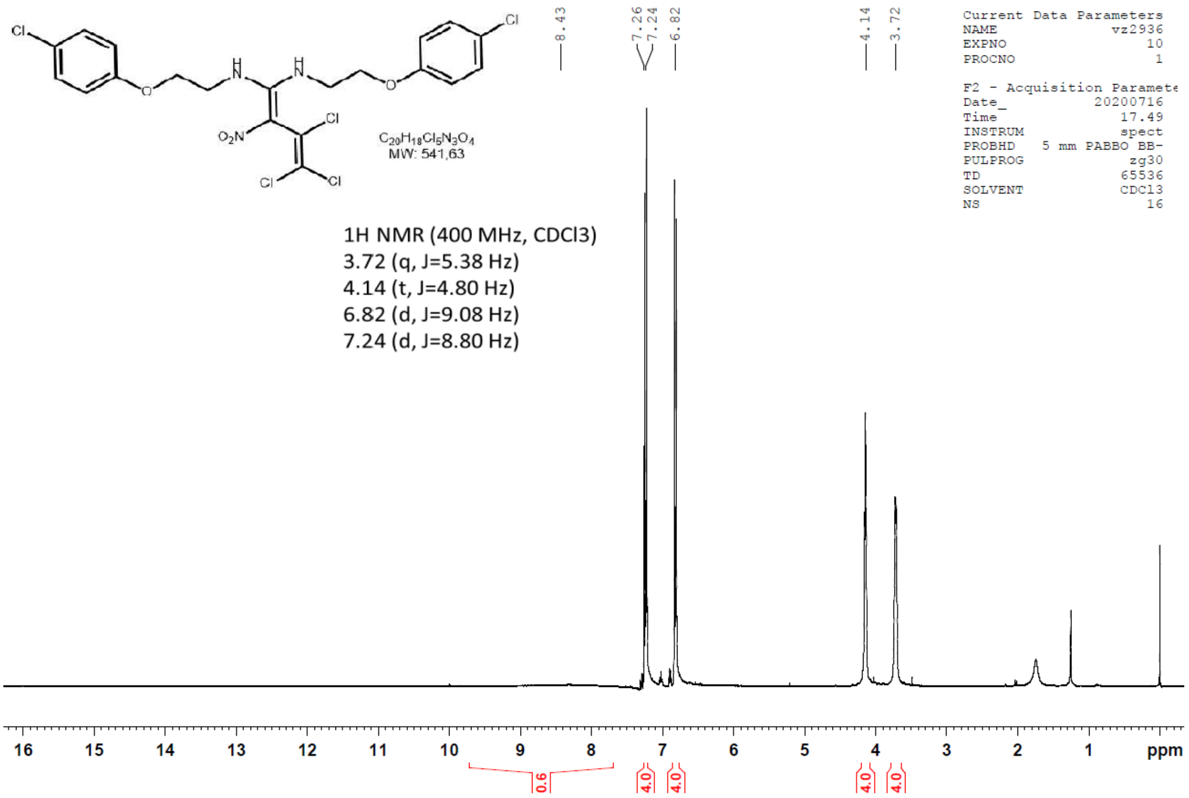

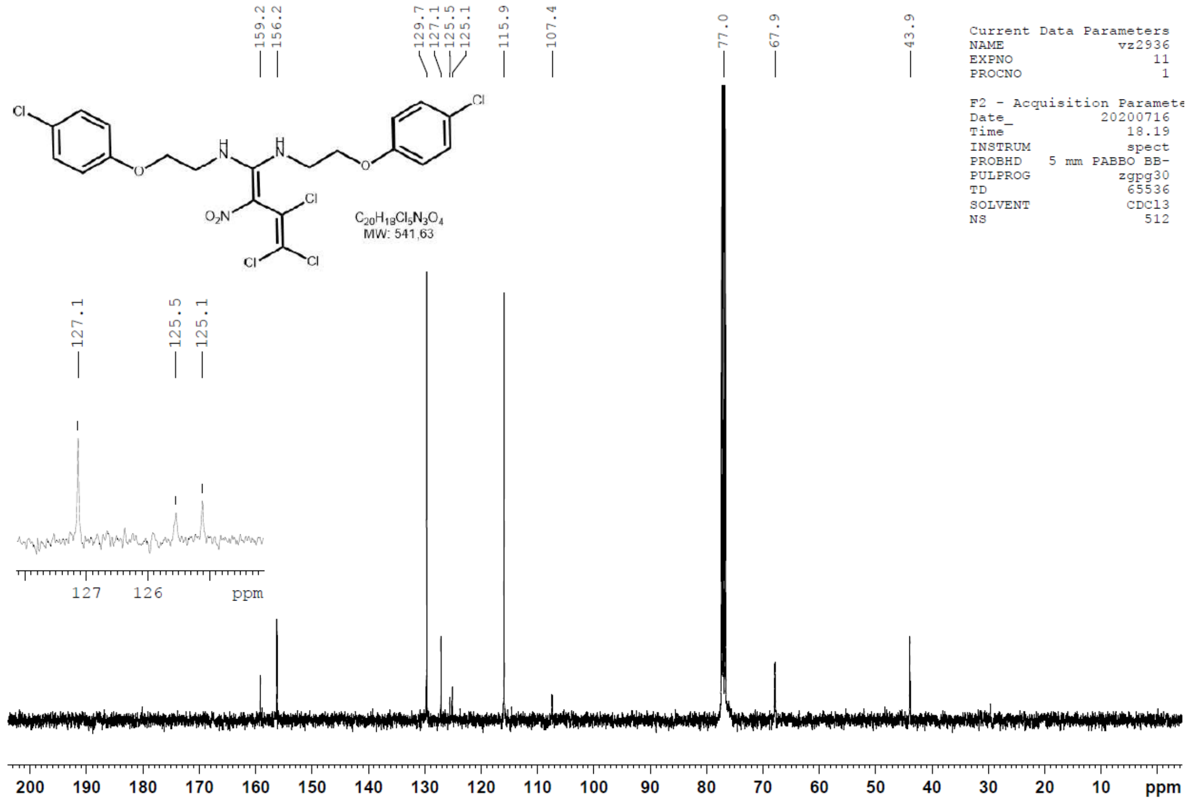

Figure S6-S7. ^{1}H and ^{13}C NMR spectra of compound **2c**

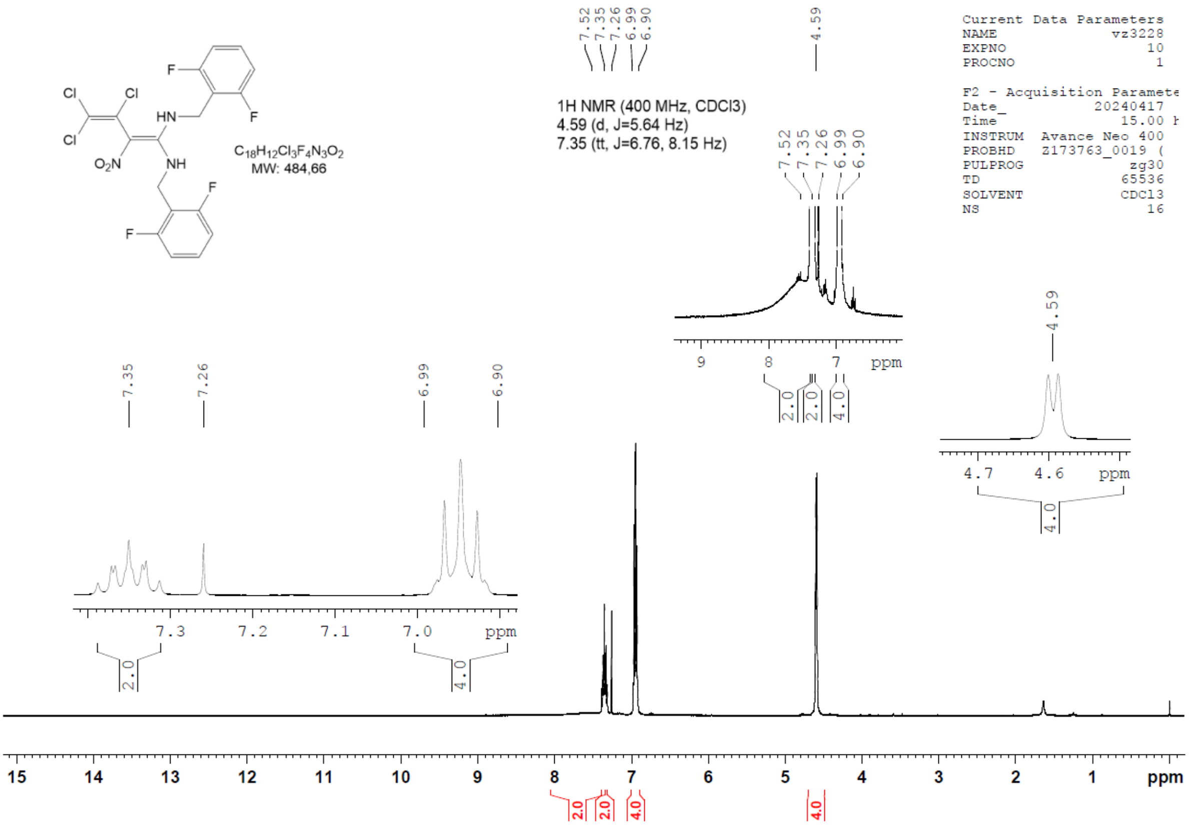

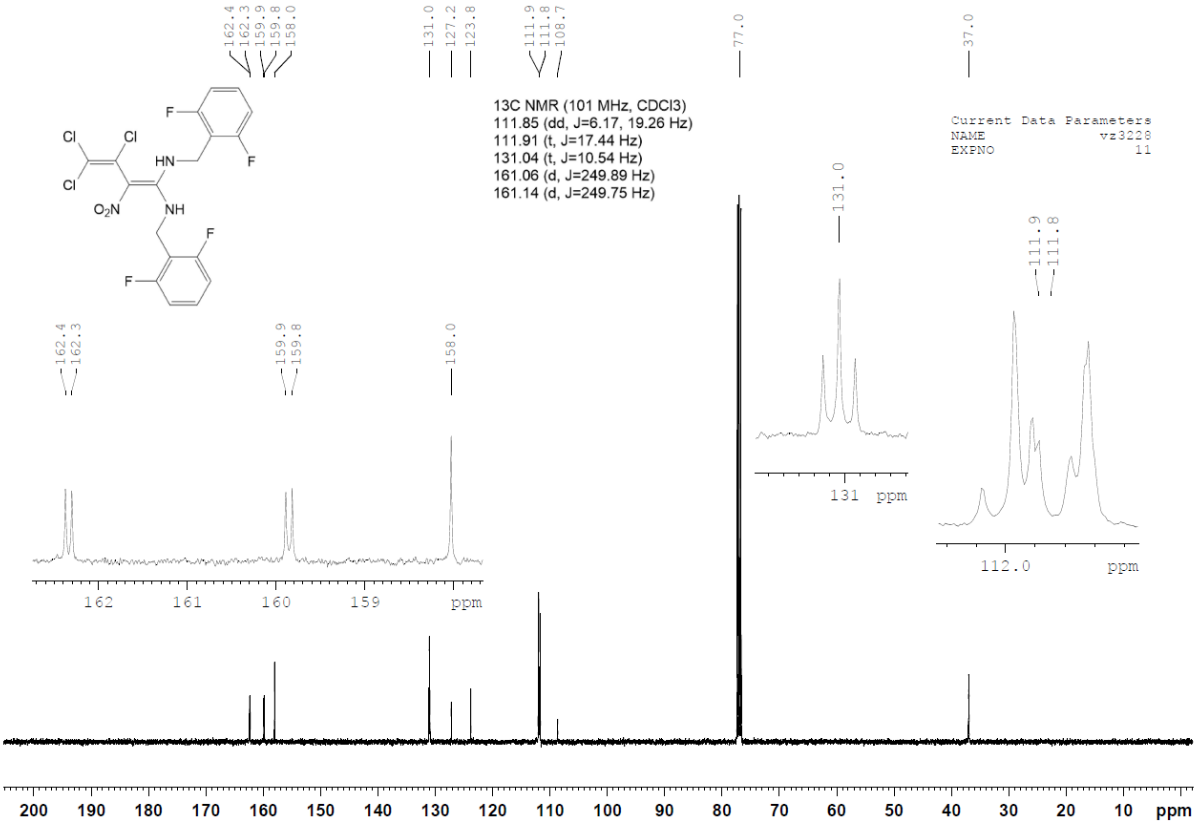

Figure S8-S9. ^{1}H and ^{13}C NMR spectra of compound **2d**

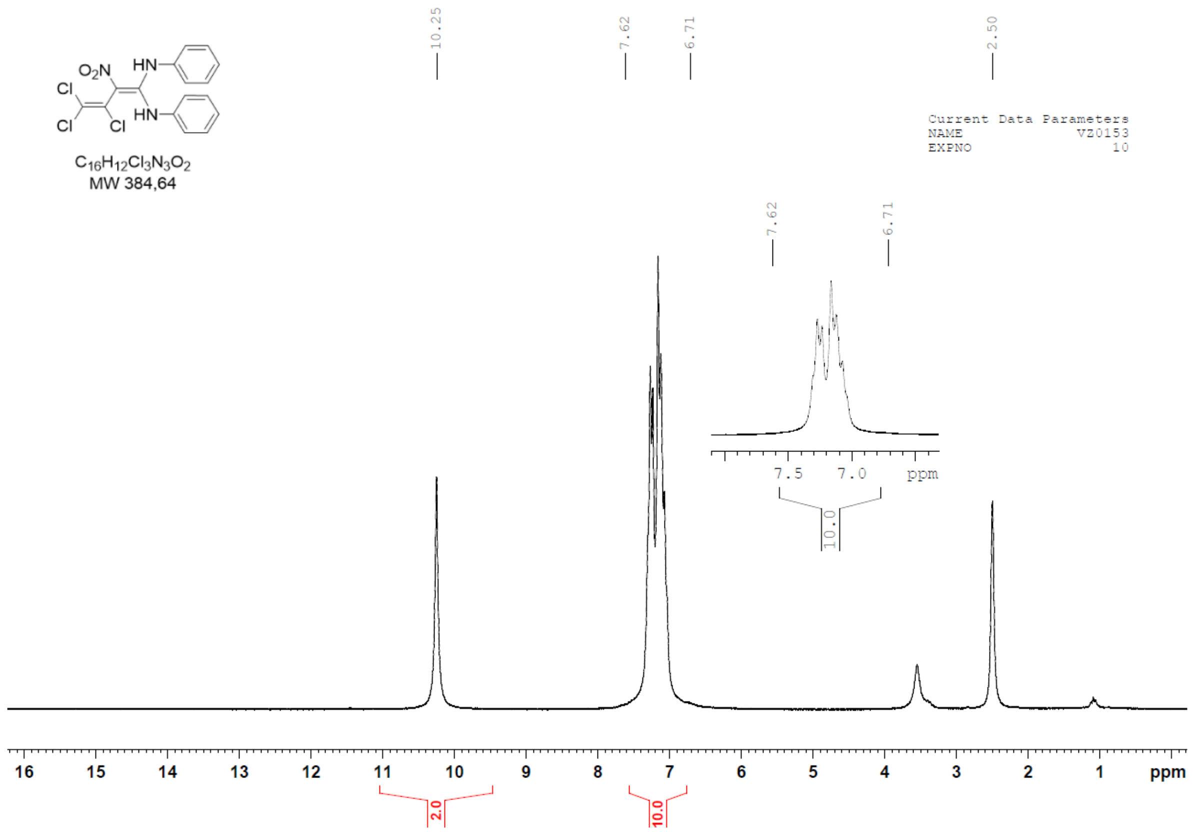

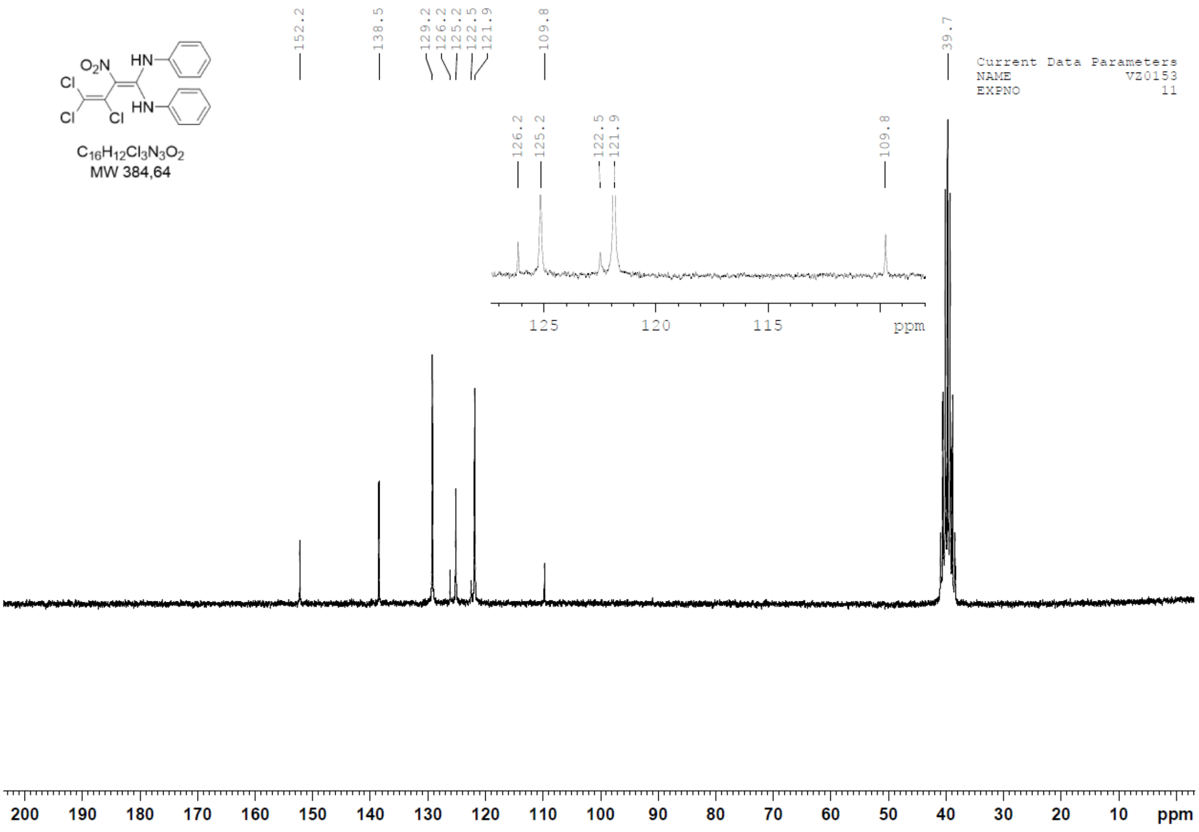

Figure S9-S10. ^{1}H and ^{13}C NMR spectra of compound **2e**

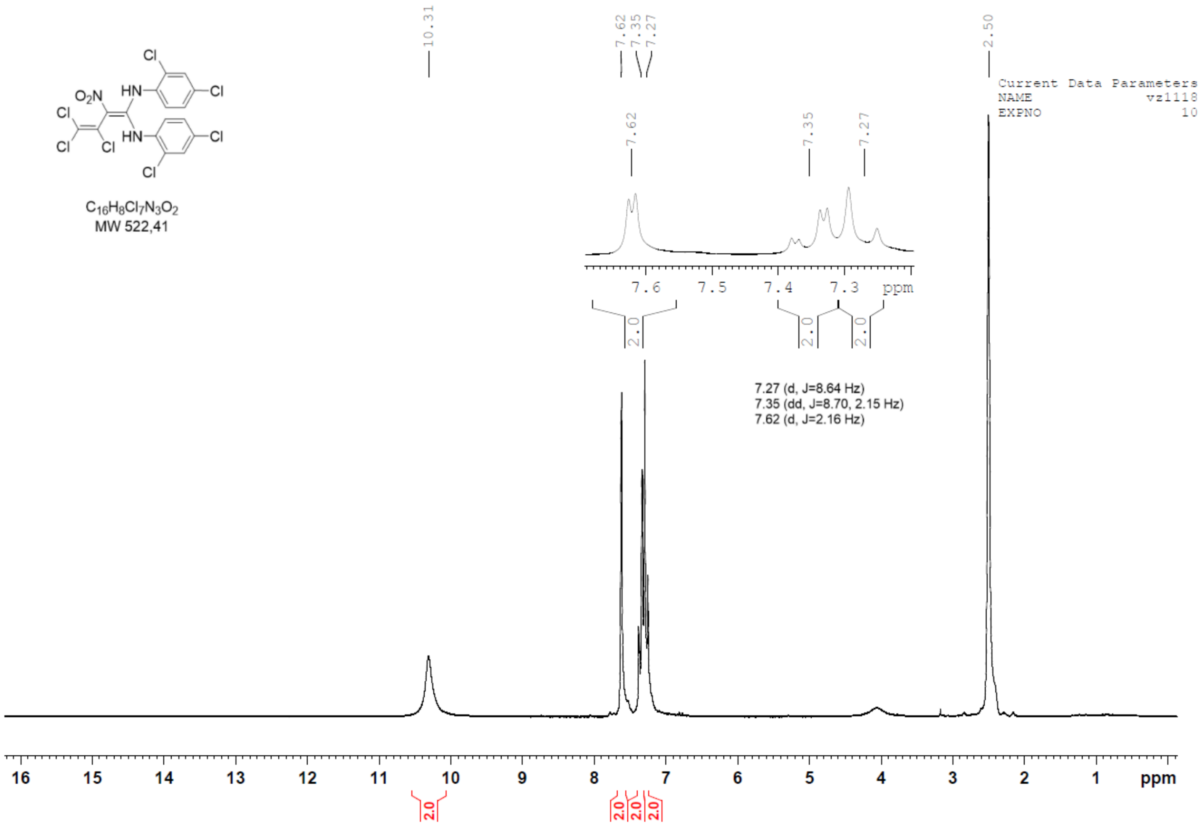

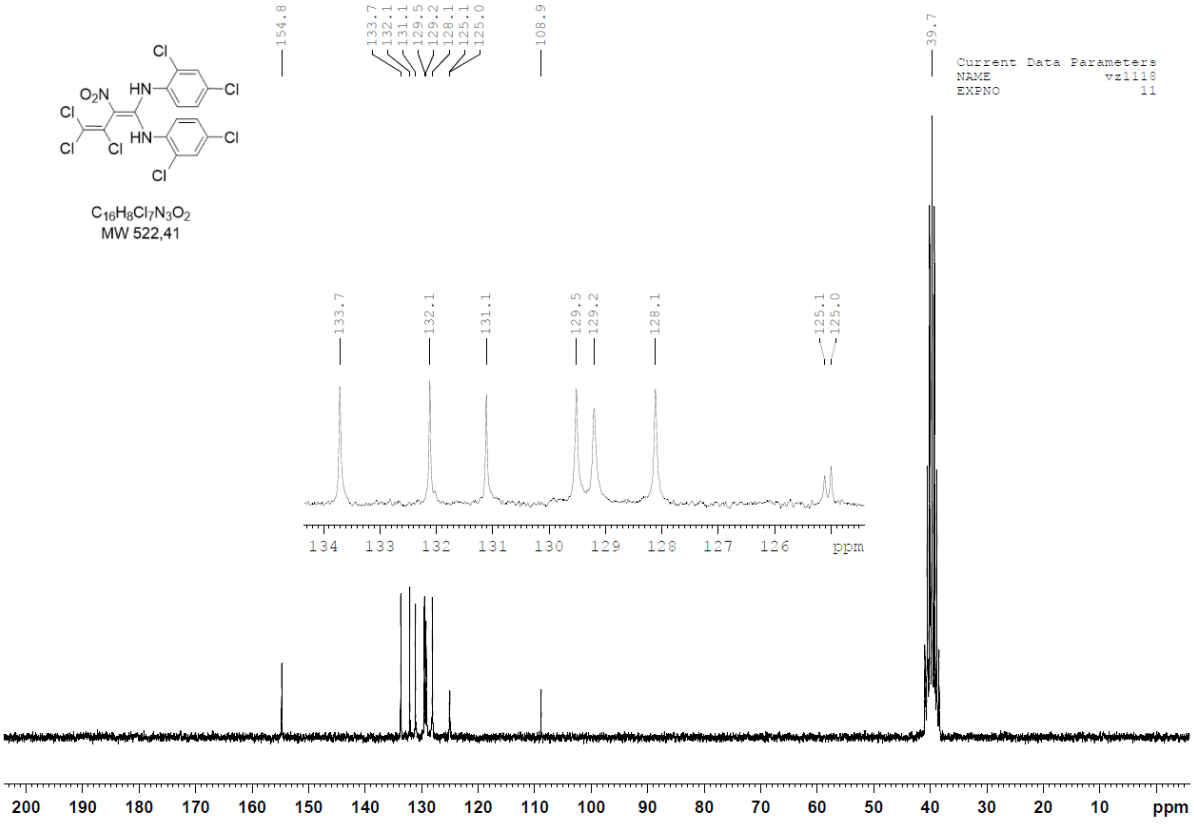

Figure S11-S12. ^{1}H and ^{13}C NMR spectra of compound **2i**

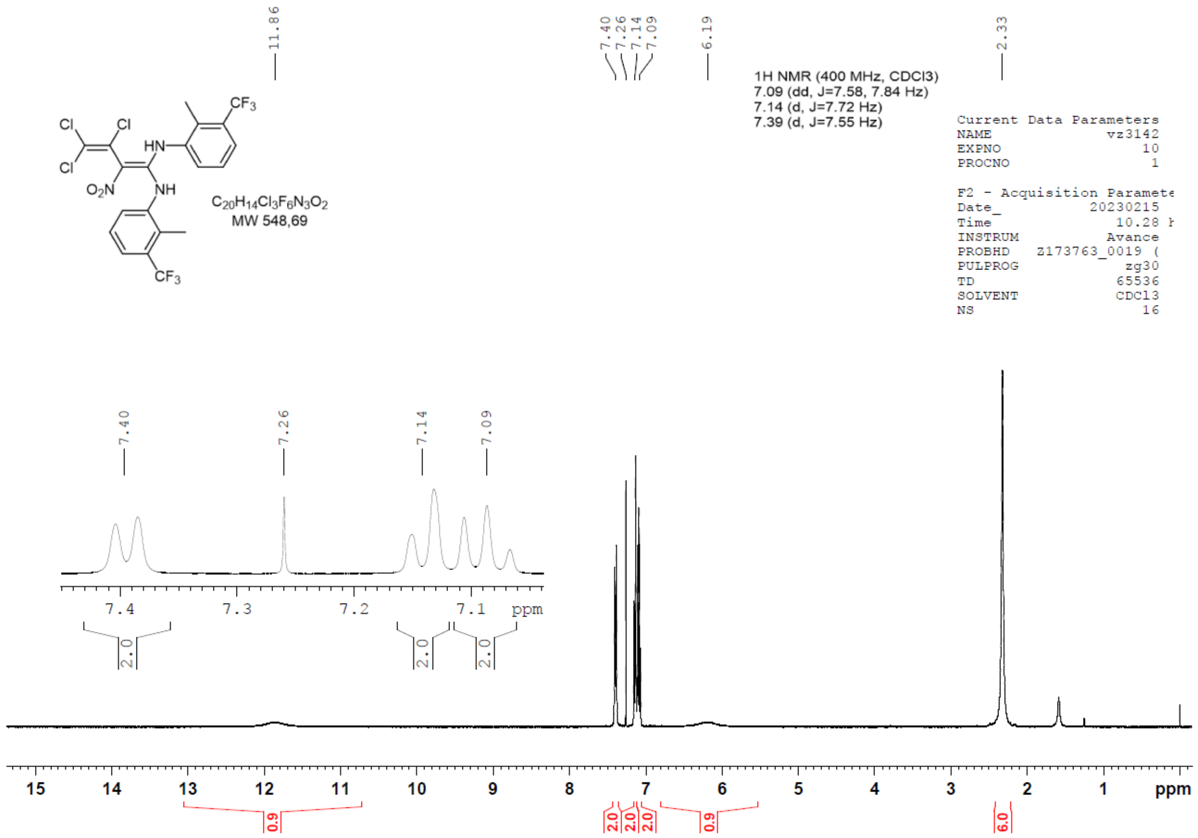

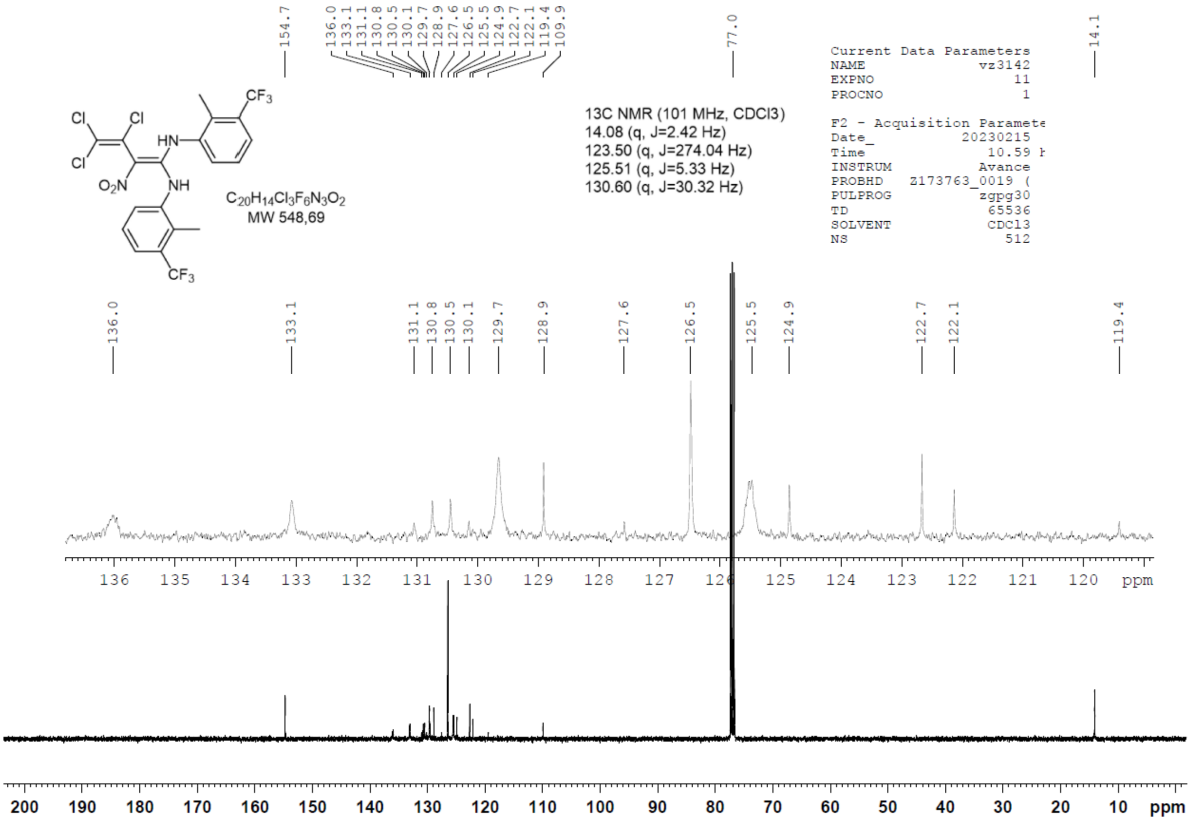

Figure S13-S14. ^{1}H and ^{13}C NMR spectra of compound **2j**

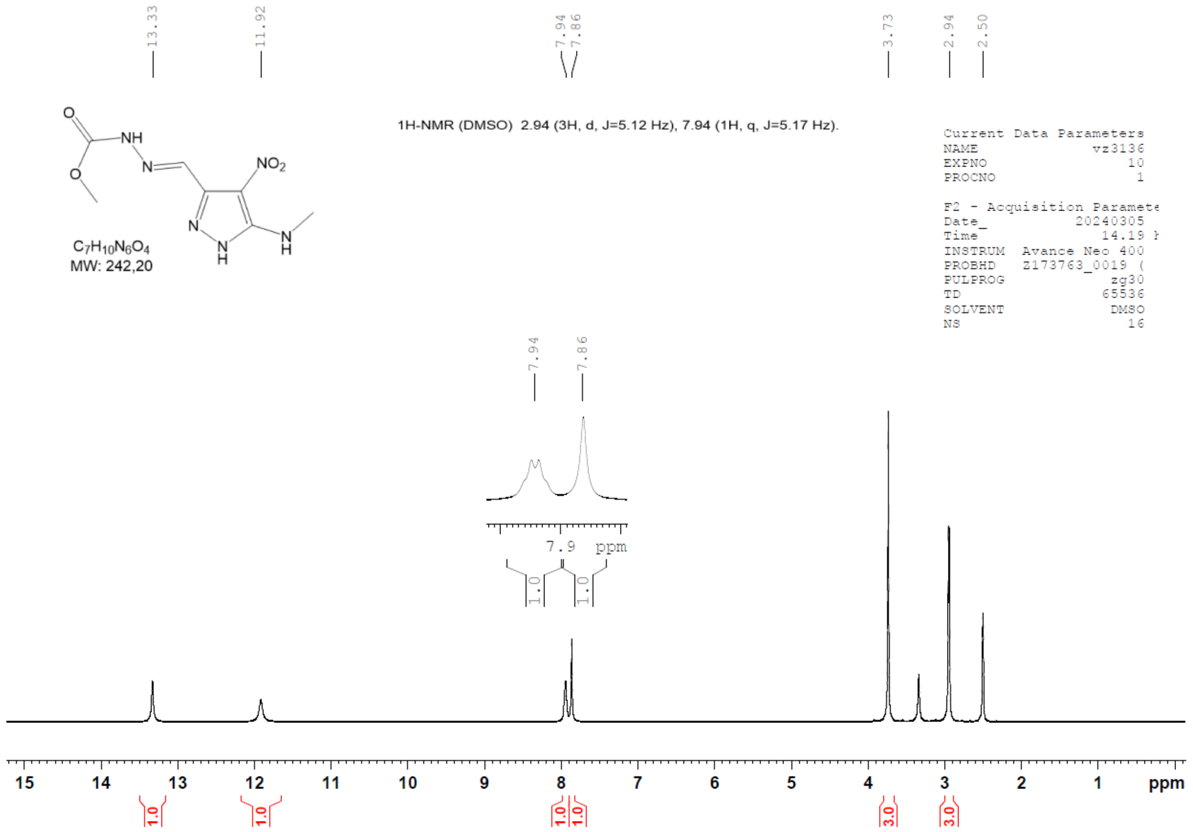

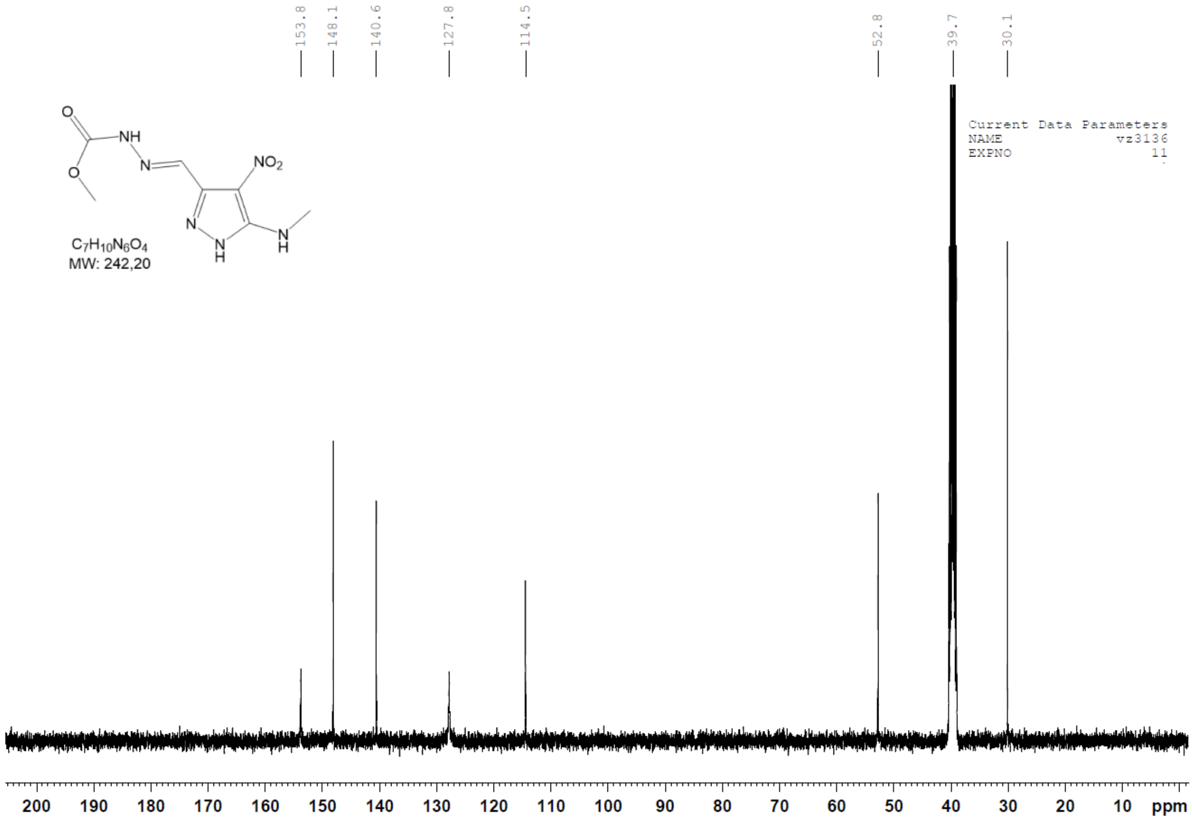

Figure S15-S16. ^{1}H and ^{13}C NMR spectra of compound **3a**

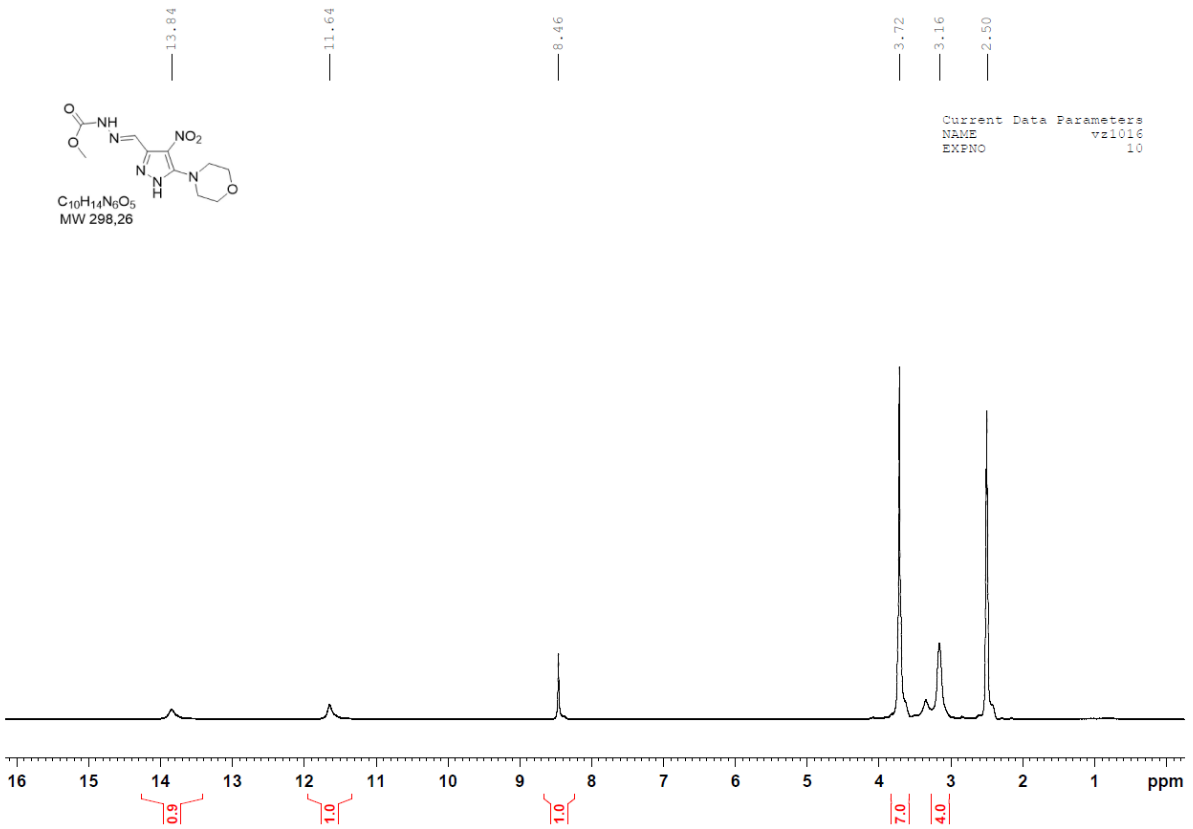

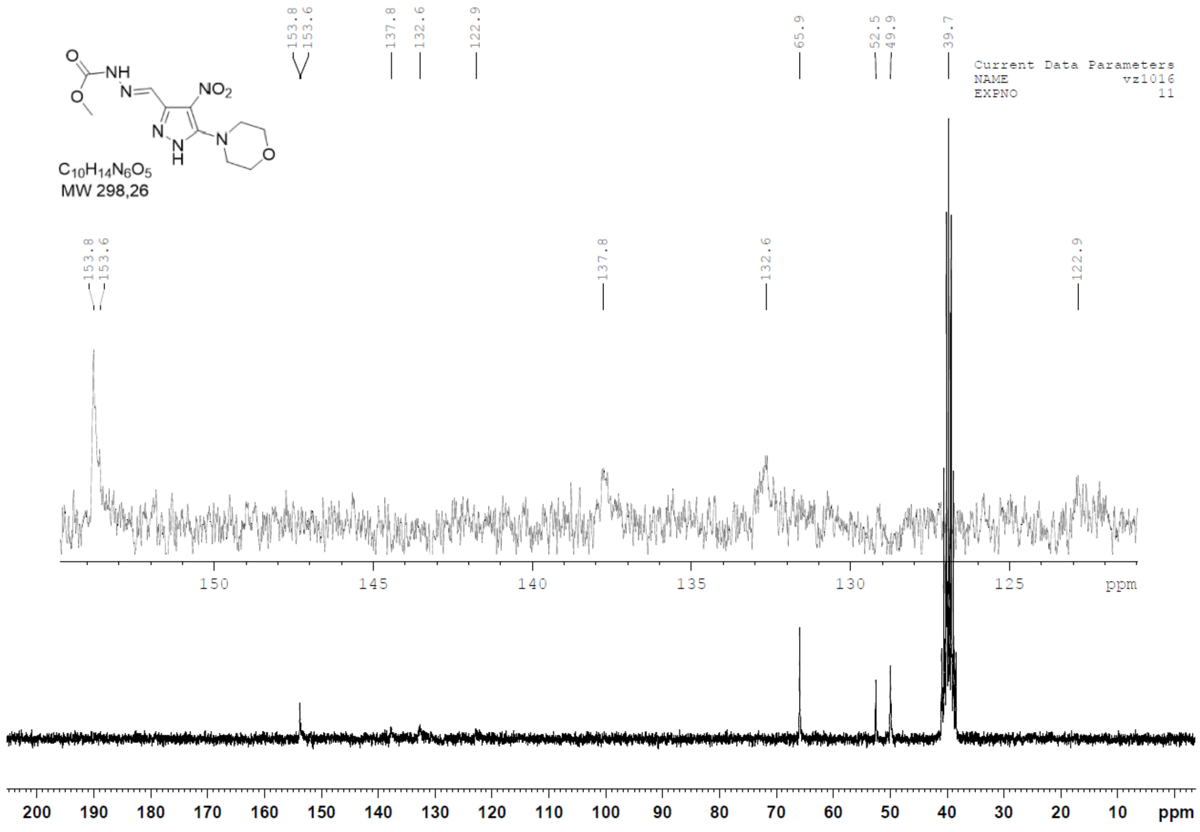

Figure S17-S18. ^{1}H and ^{13}C NMR spectra of compound **3b**

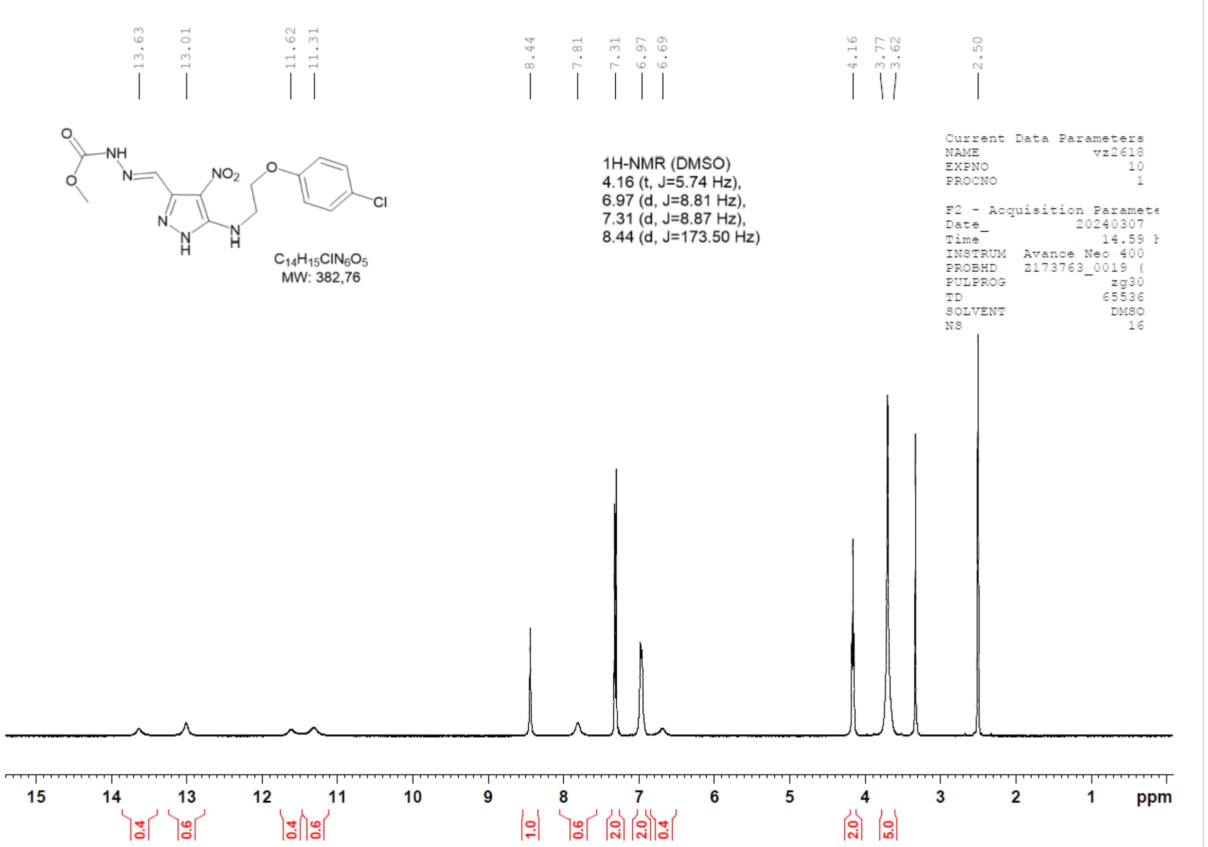

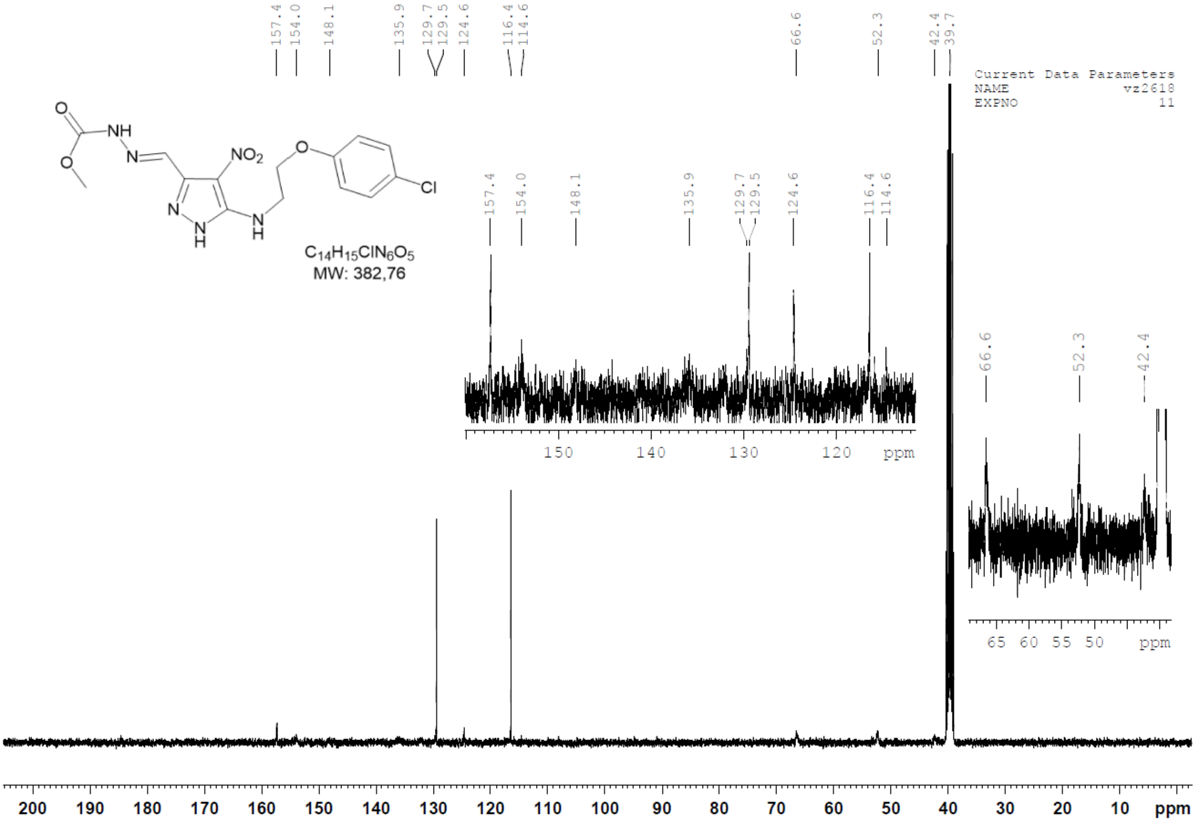

Figure S19-S20. ^{1}H and ^{13}C NMR spectra of compound **3c**

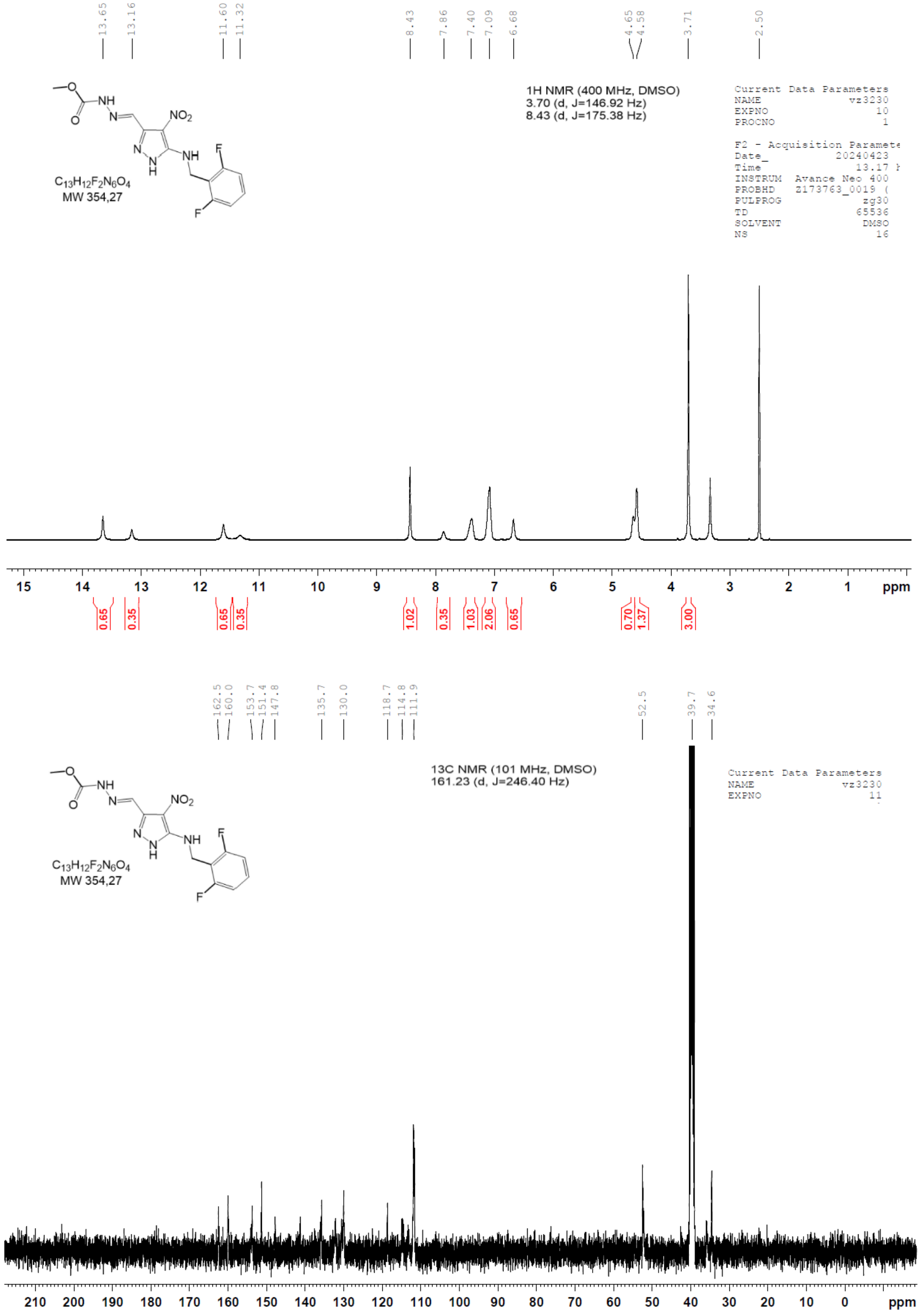

Figure S21-S22. ^{1}H and ^{13}C NMR spectra of compound **3d**

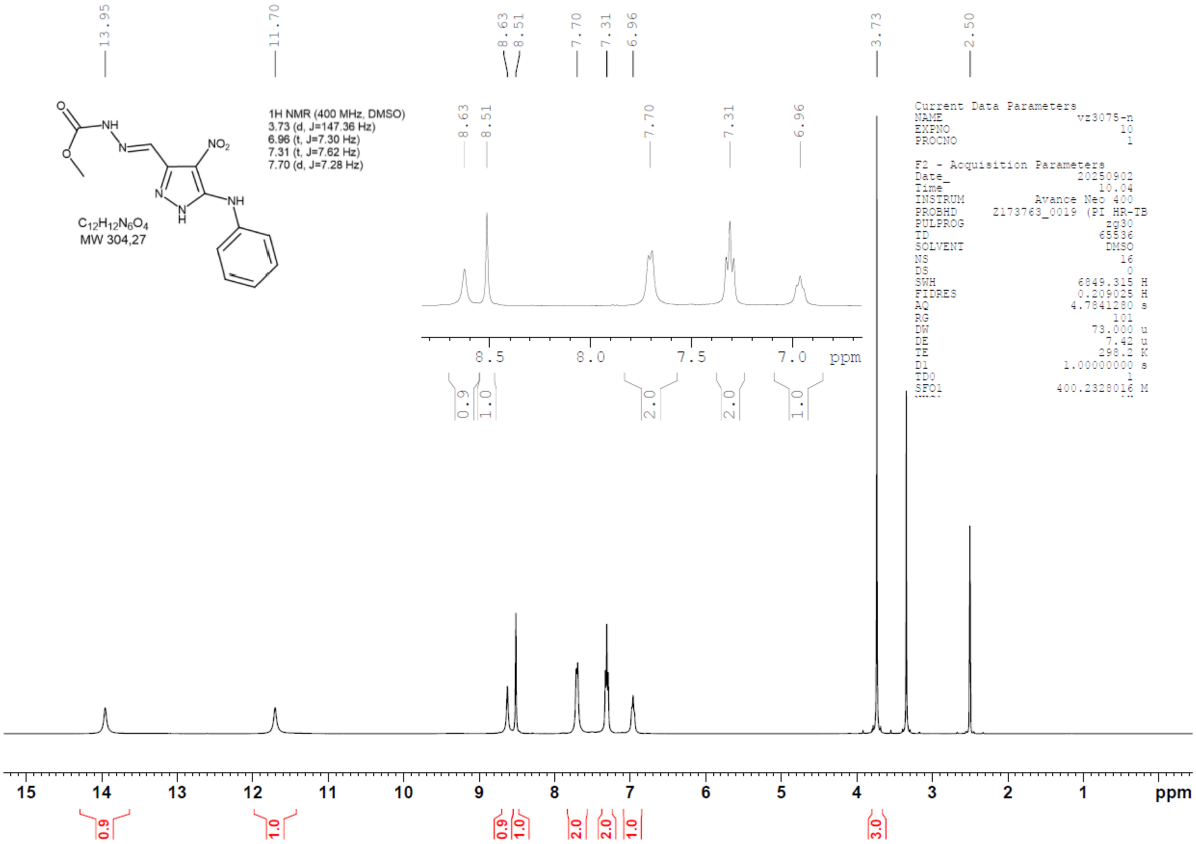

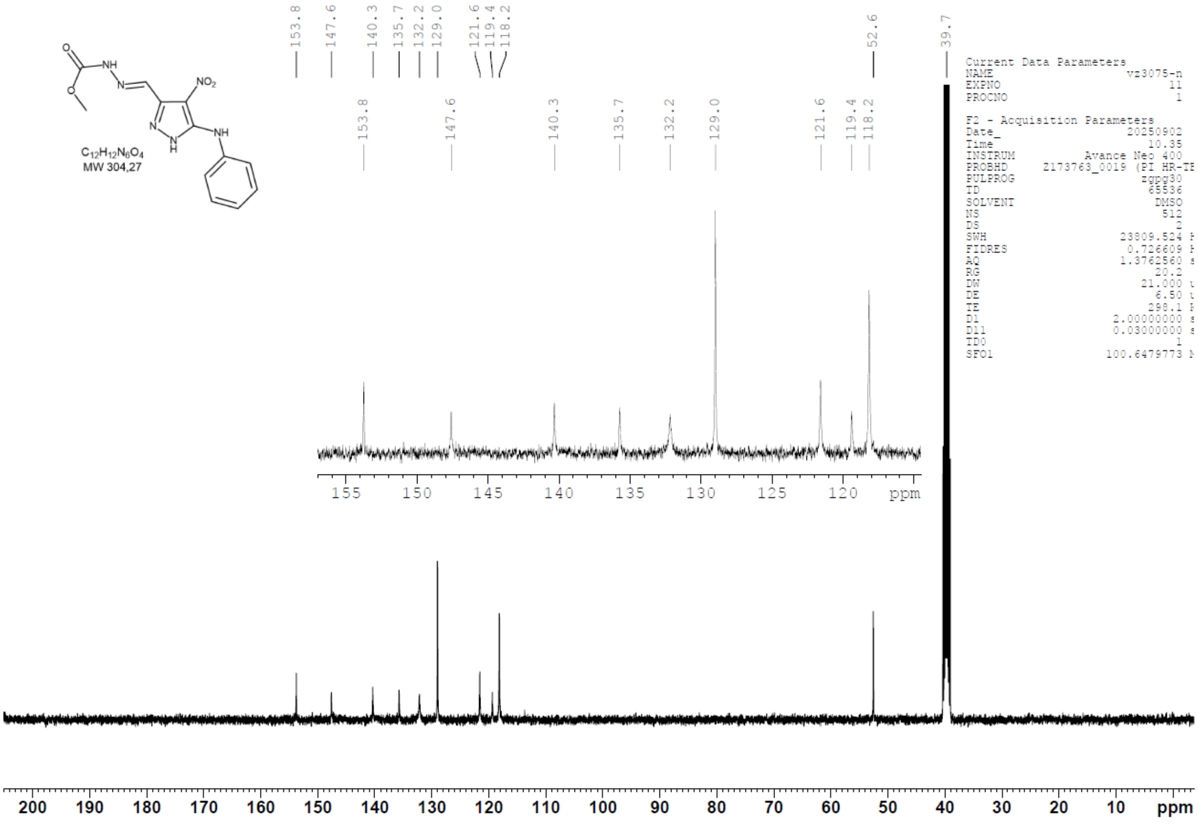

Figure S23-S24. ^{1}H and ^{13}C NMR spectra of compound **3e**

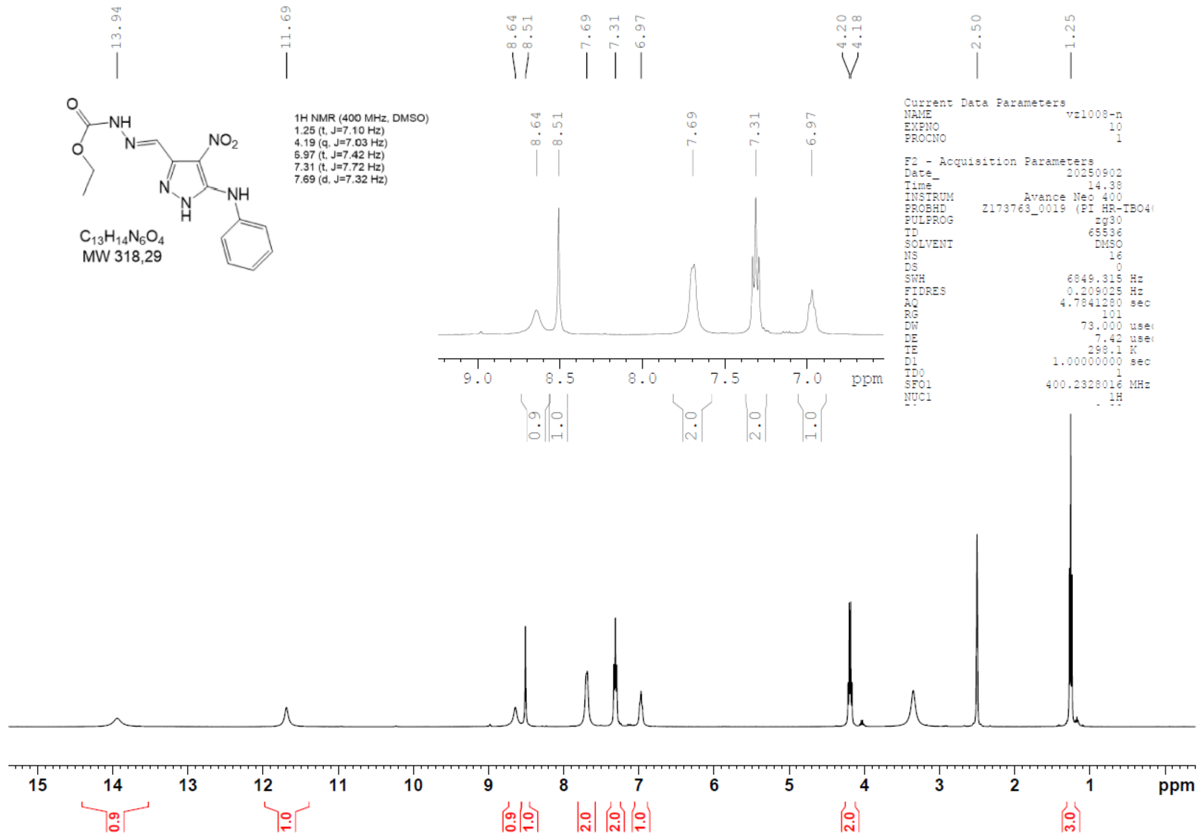

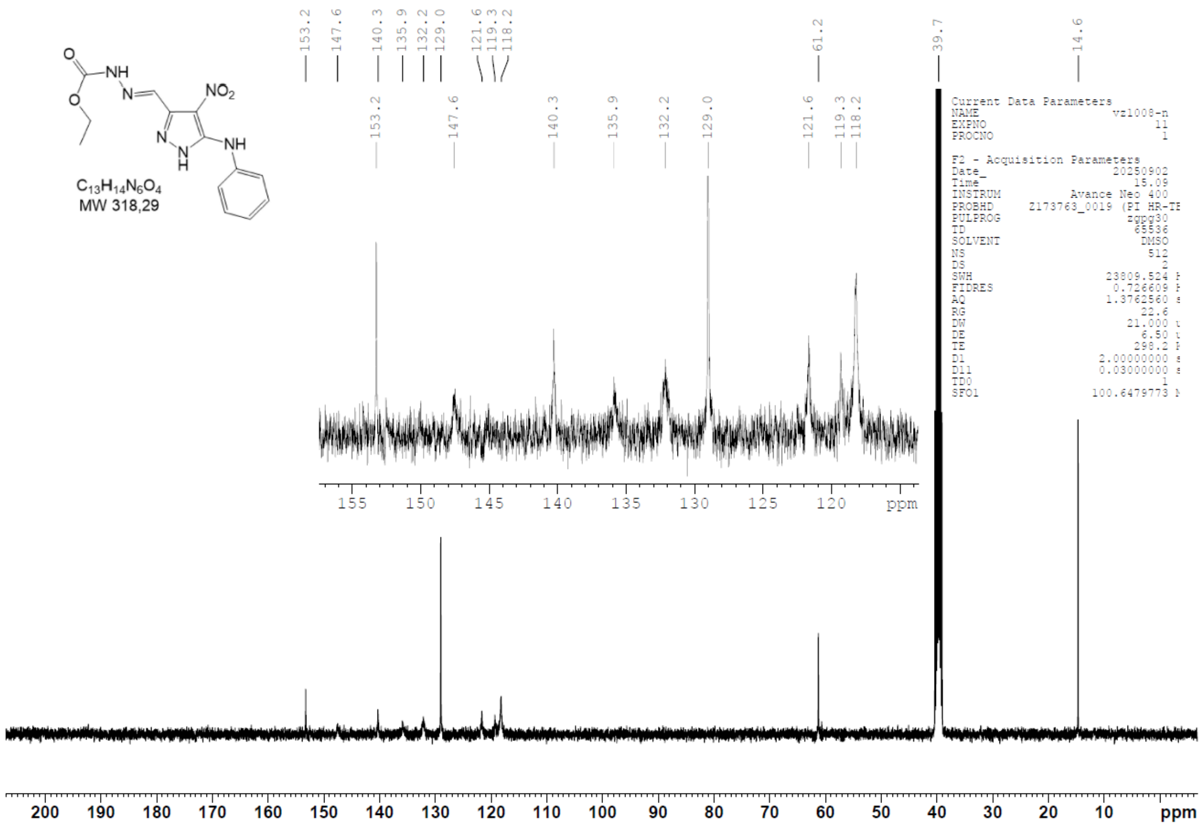

Figure S25-S26. ^{1}H and ^{13}C NMR spectra of compound **3ee**

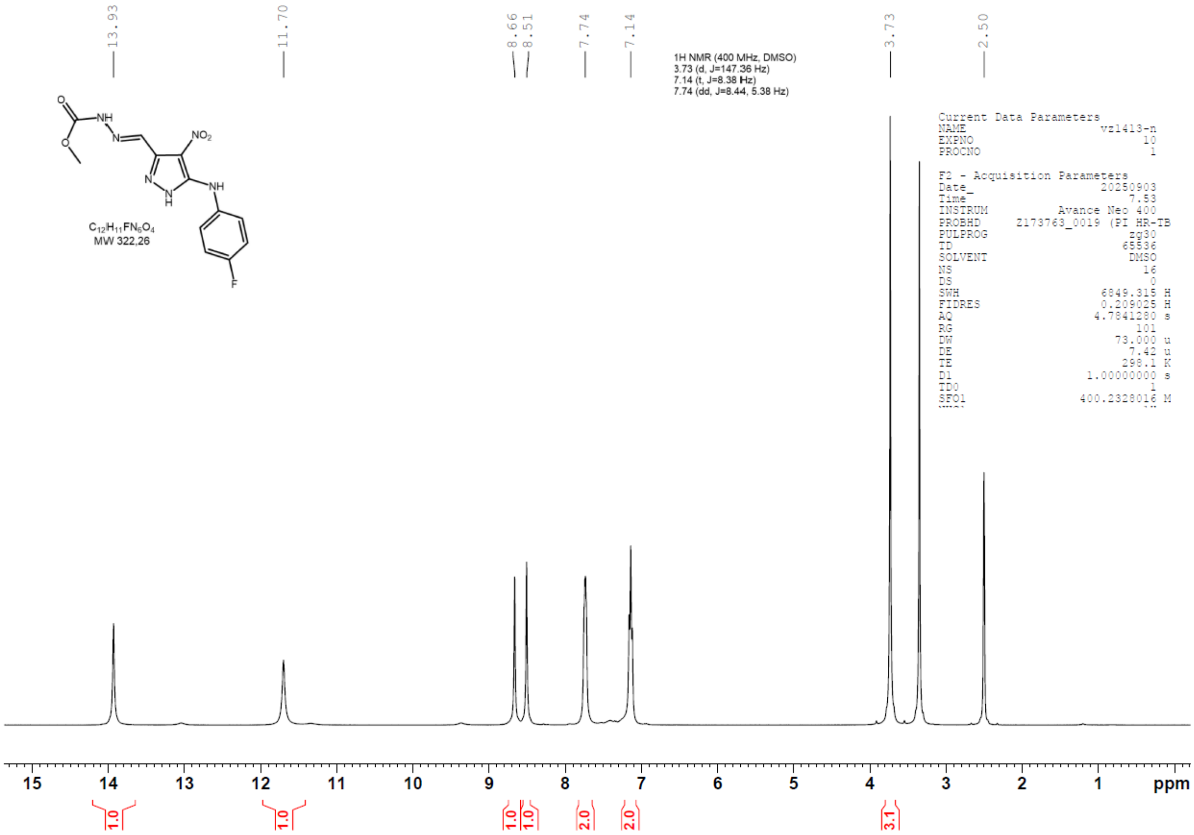

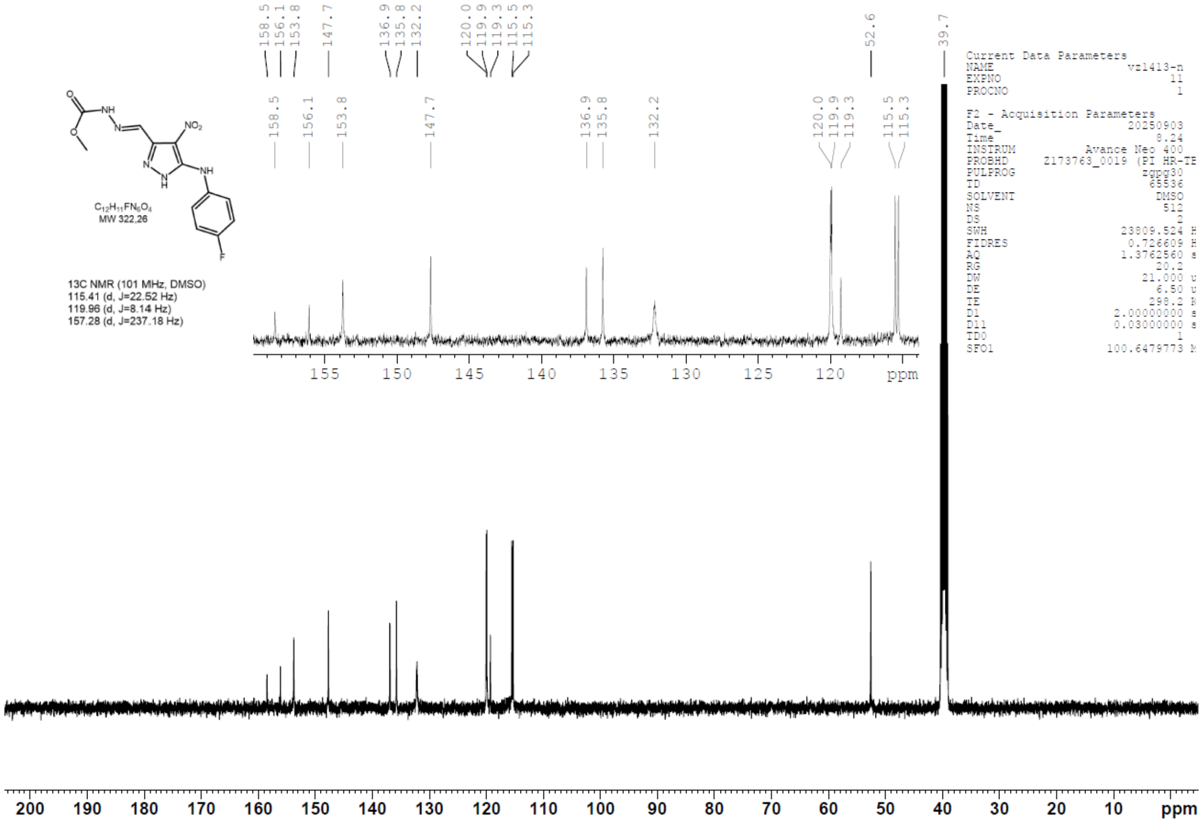

Figure S27-S28. ^{1}H and ^{13}C NMR spectra of compound **3f**

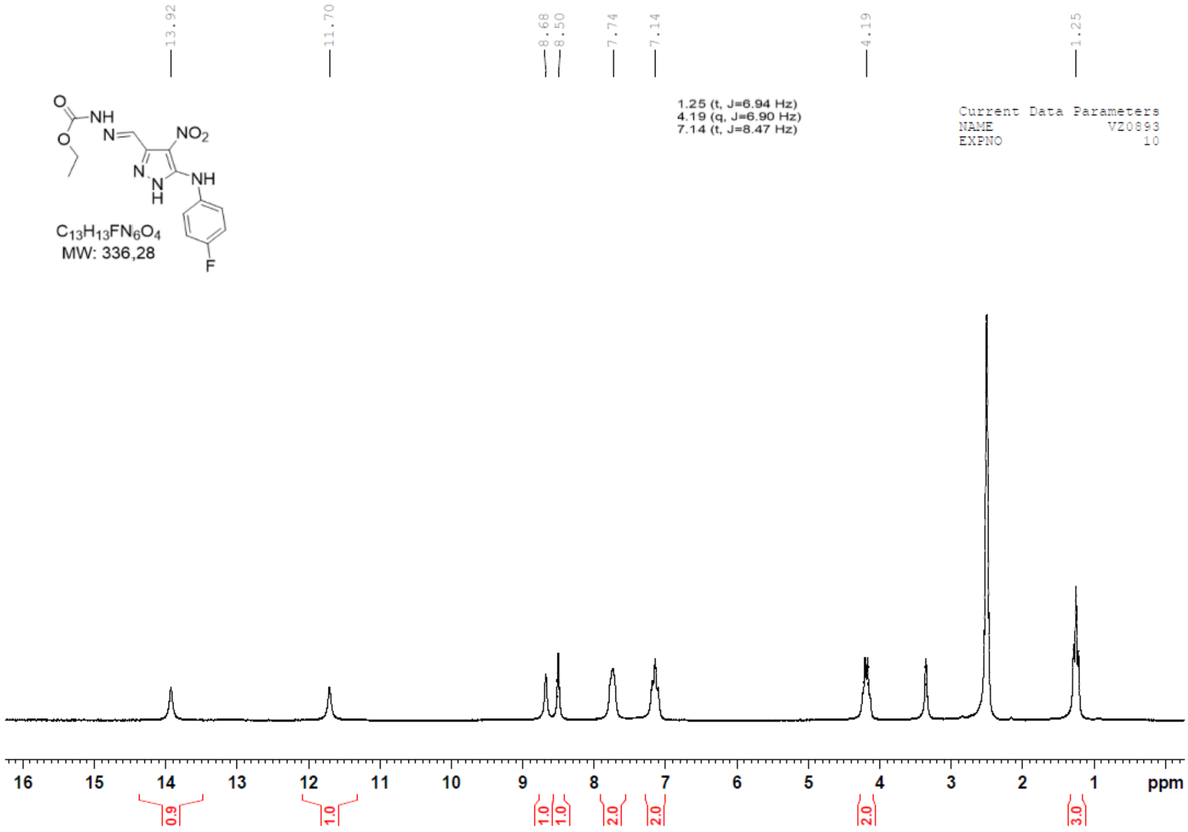

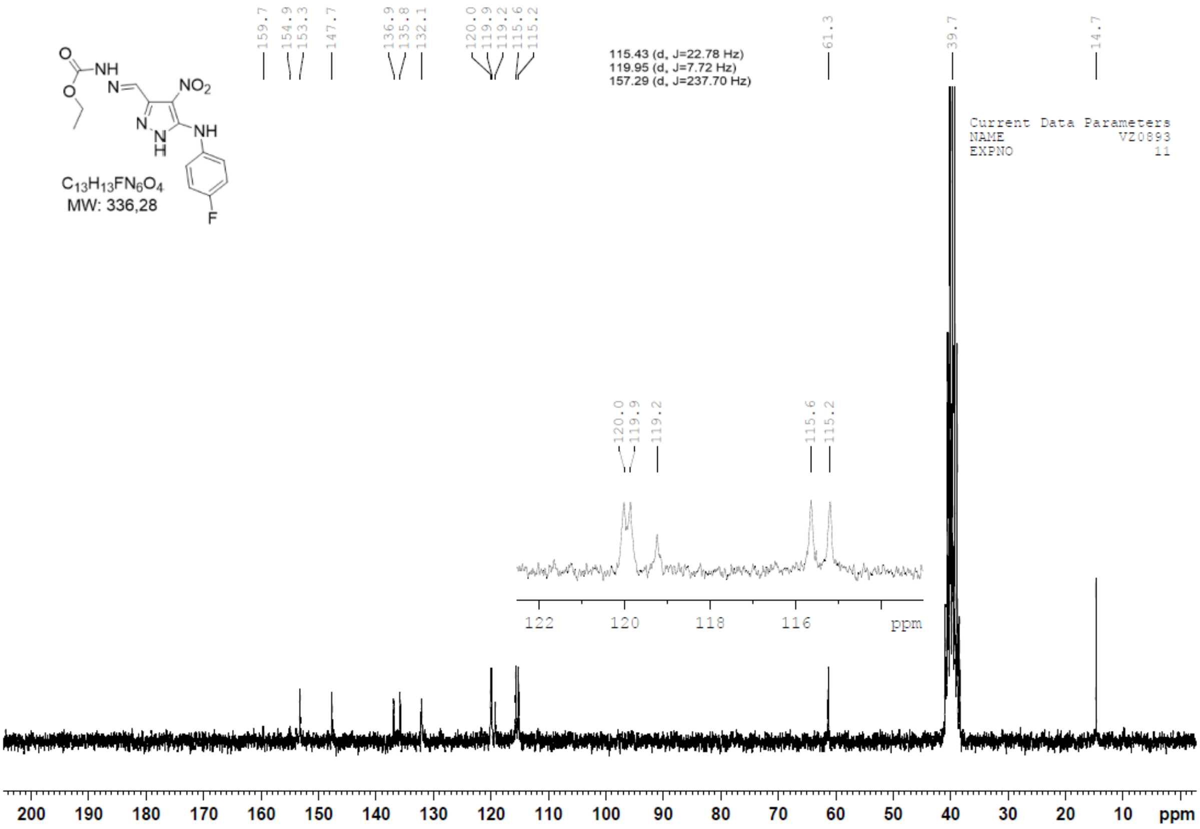

Figure S29-S30. ^{1}H and ^{13}C NMR spectra of compound **3ff**

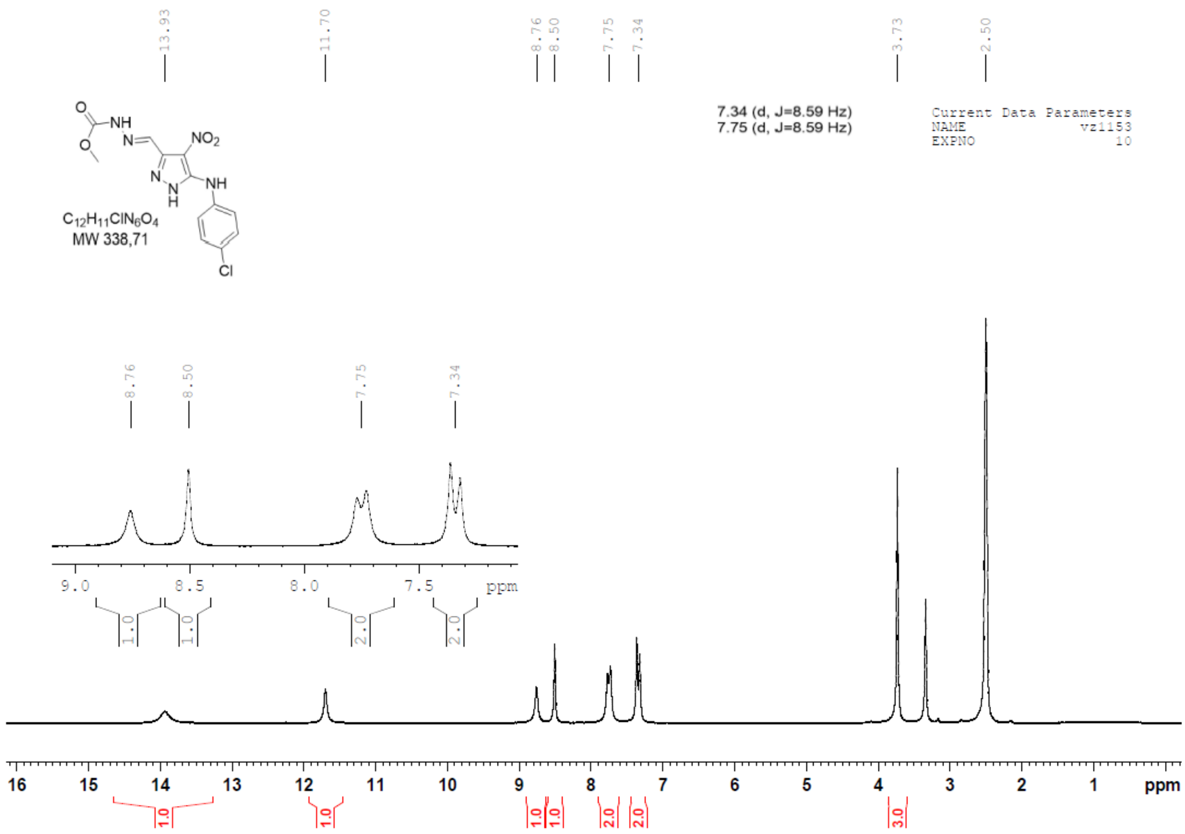

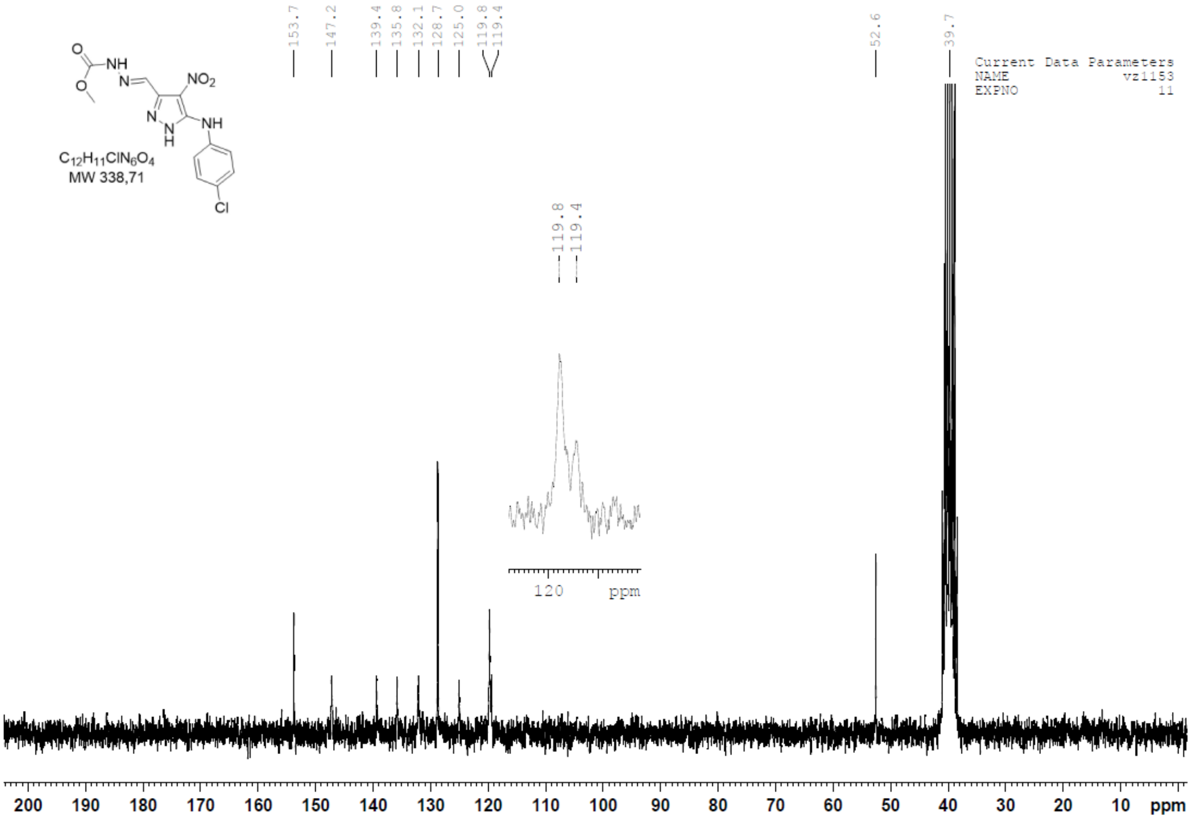

Figure S31-S32. ^{1}H and ^{13}C NMR spectra of compound **3g**

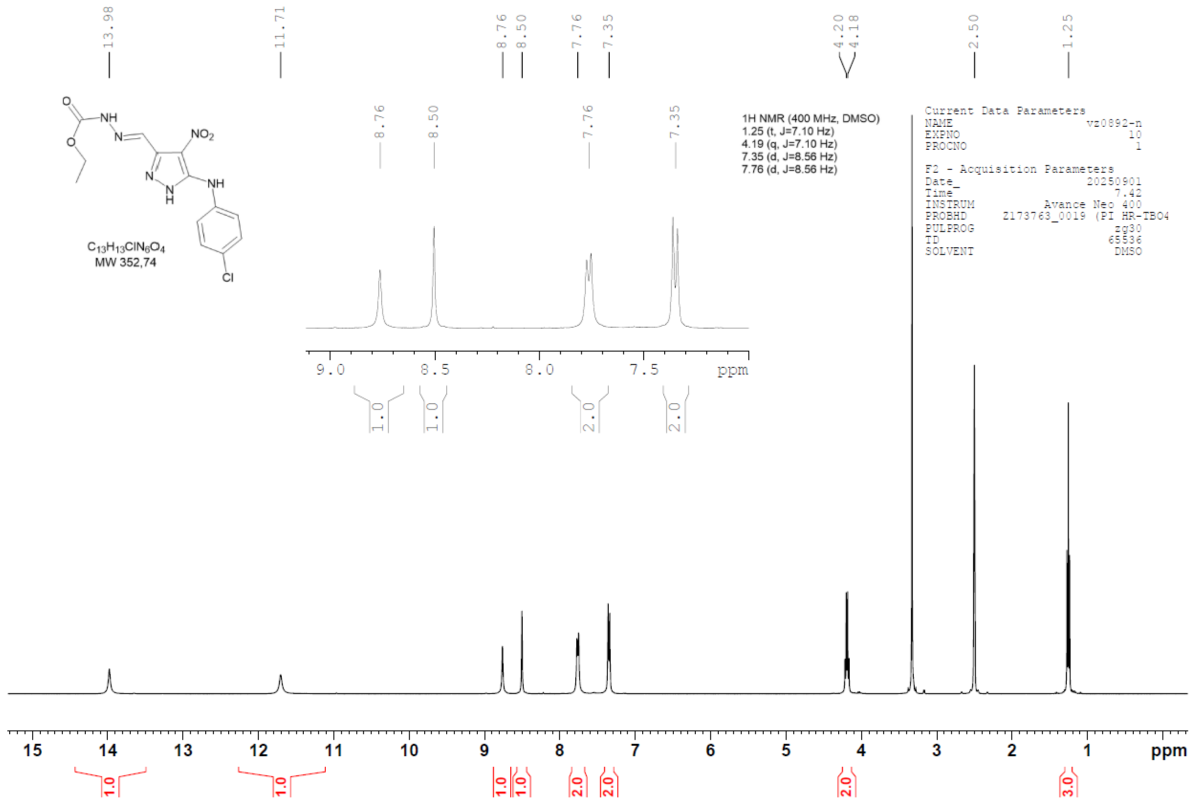

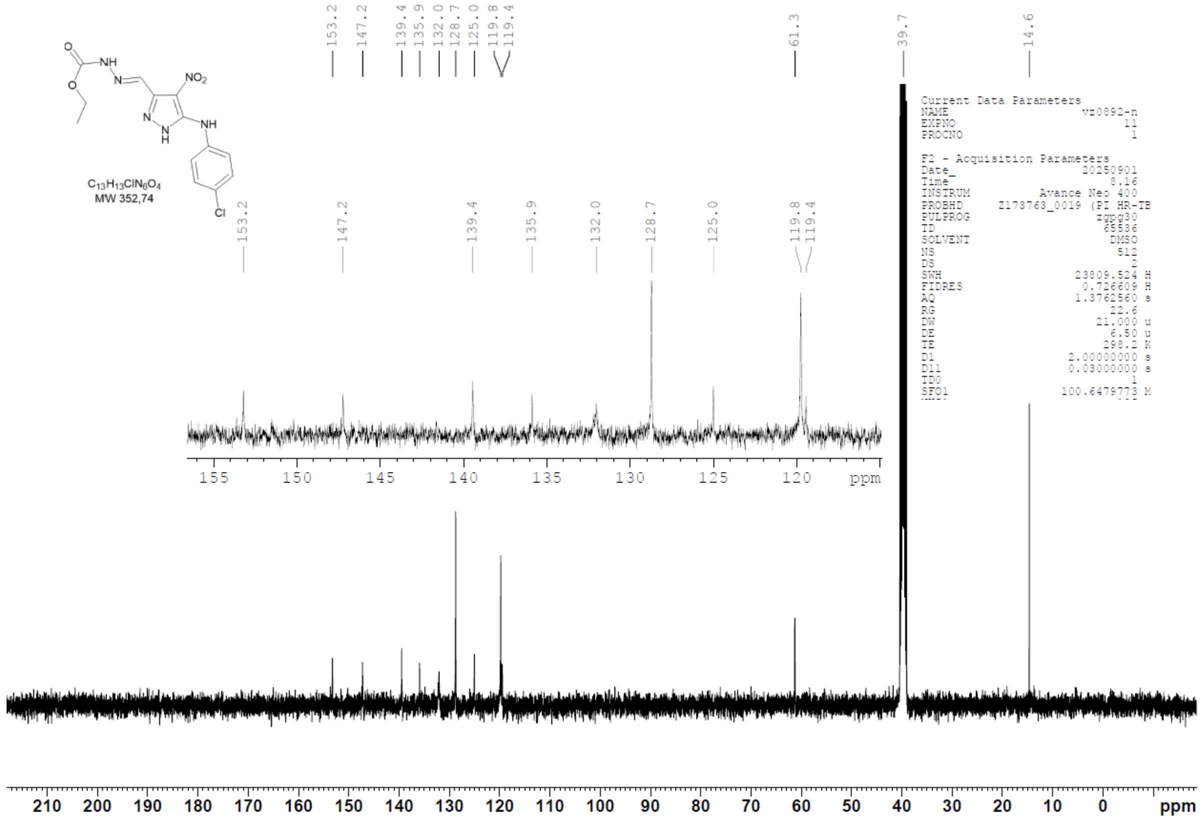

Figure S33-S34. ^{1}H and ^{13}C NMR spectra of compound **3gg**

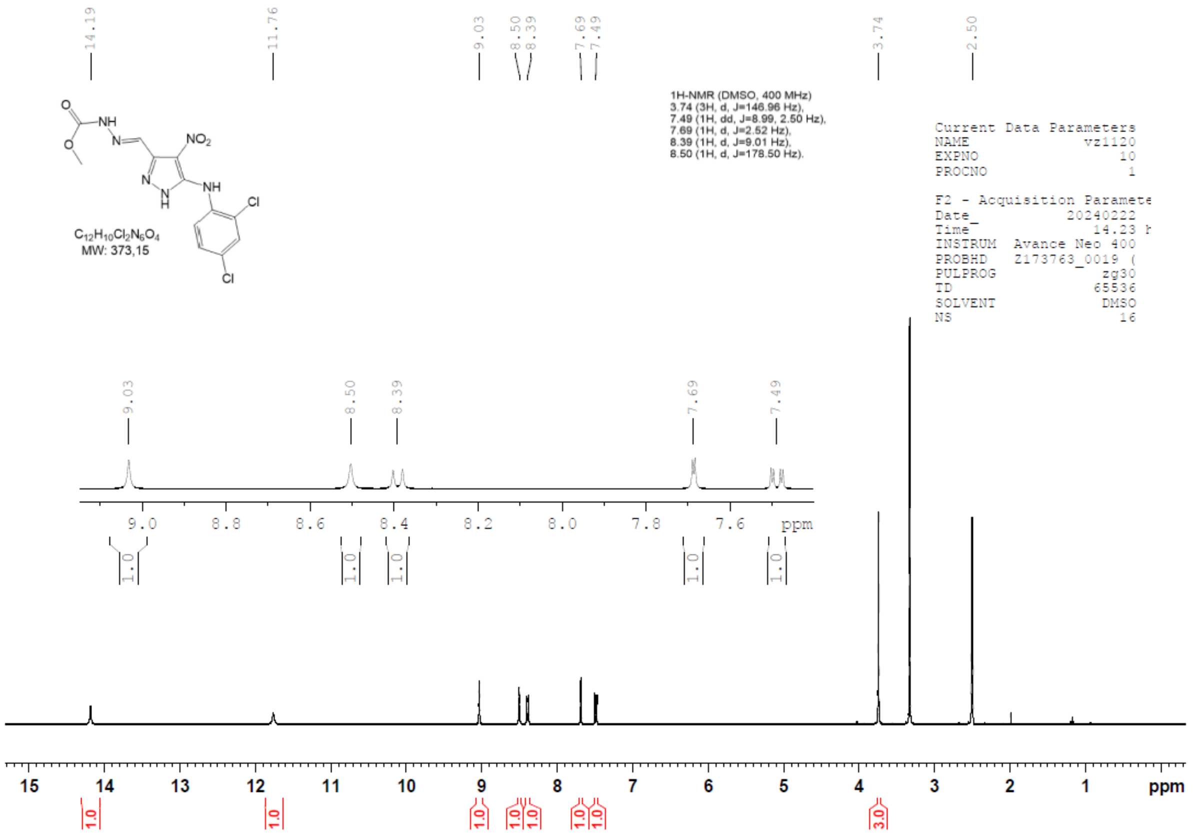

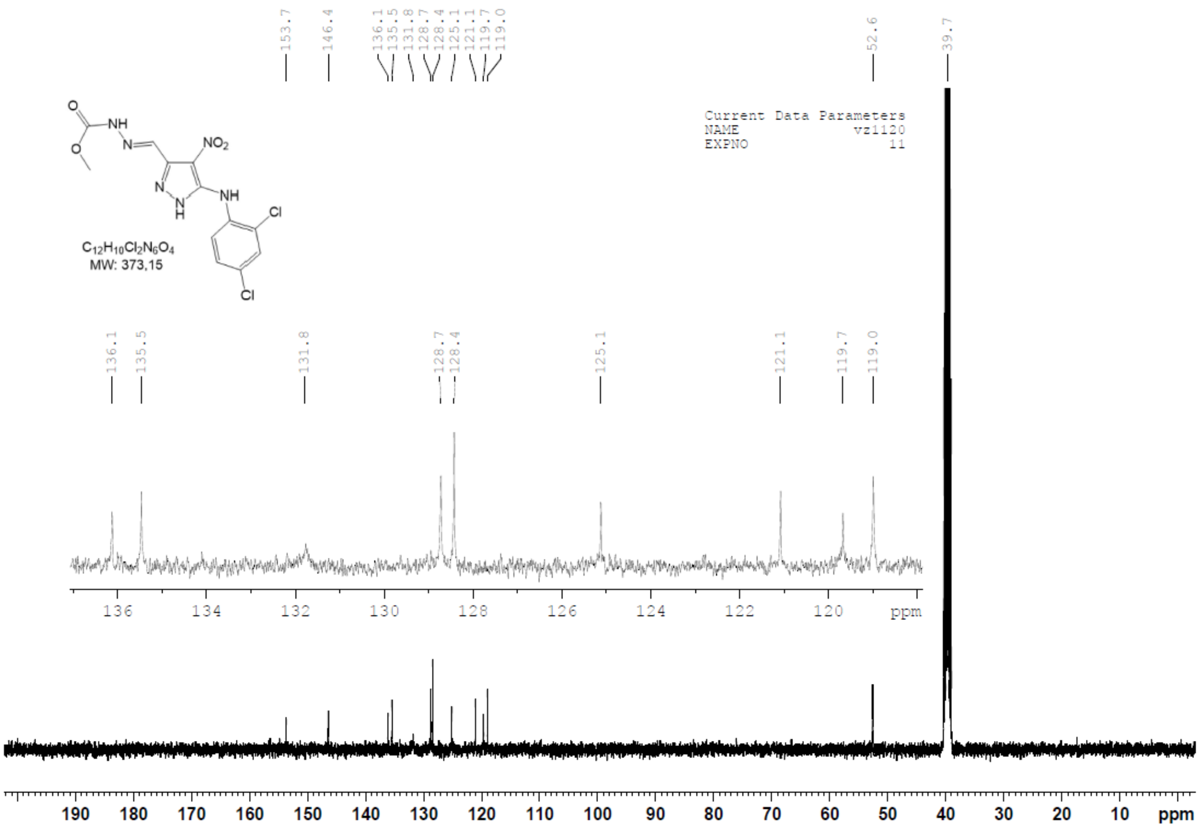

Figure S35-S36. ^{1}H and ^{13}C NMR spectra of compound **3i**

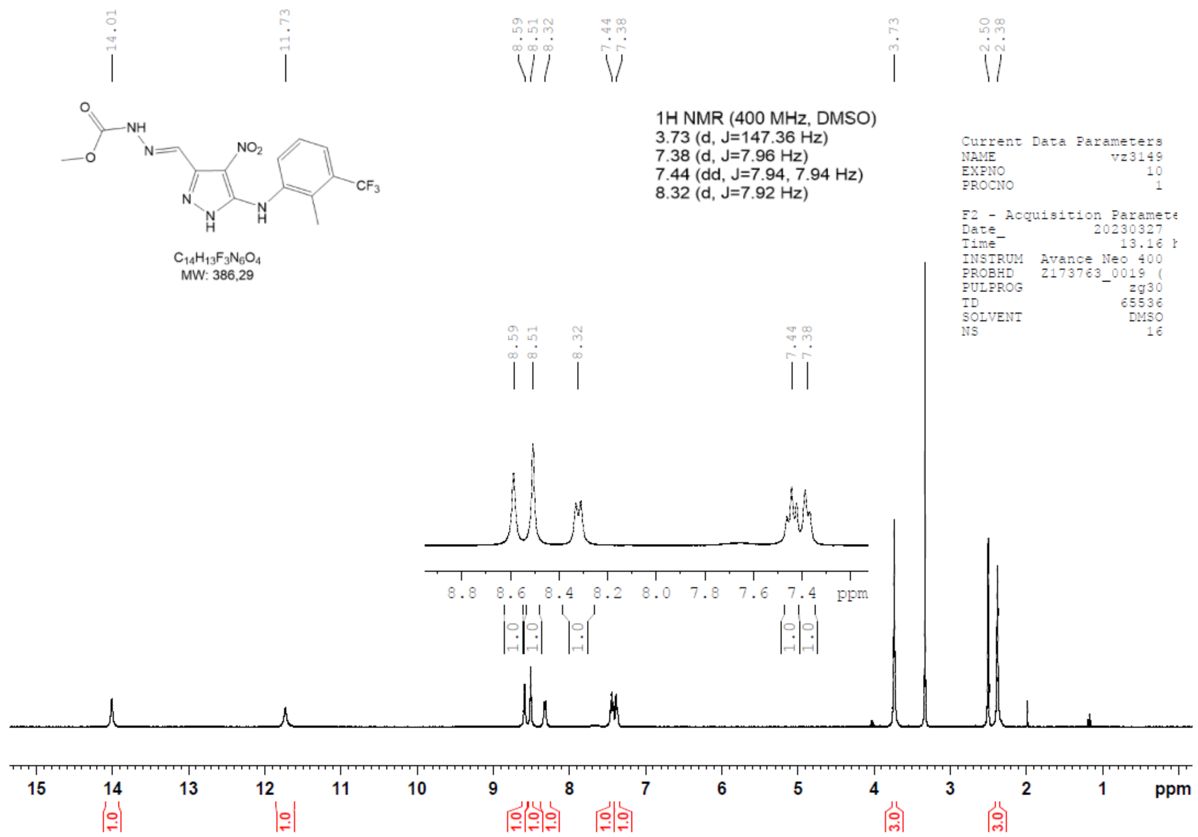

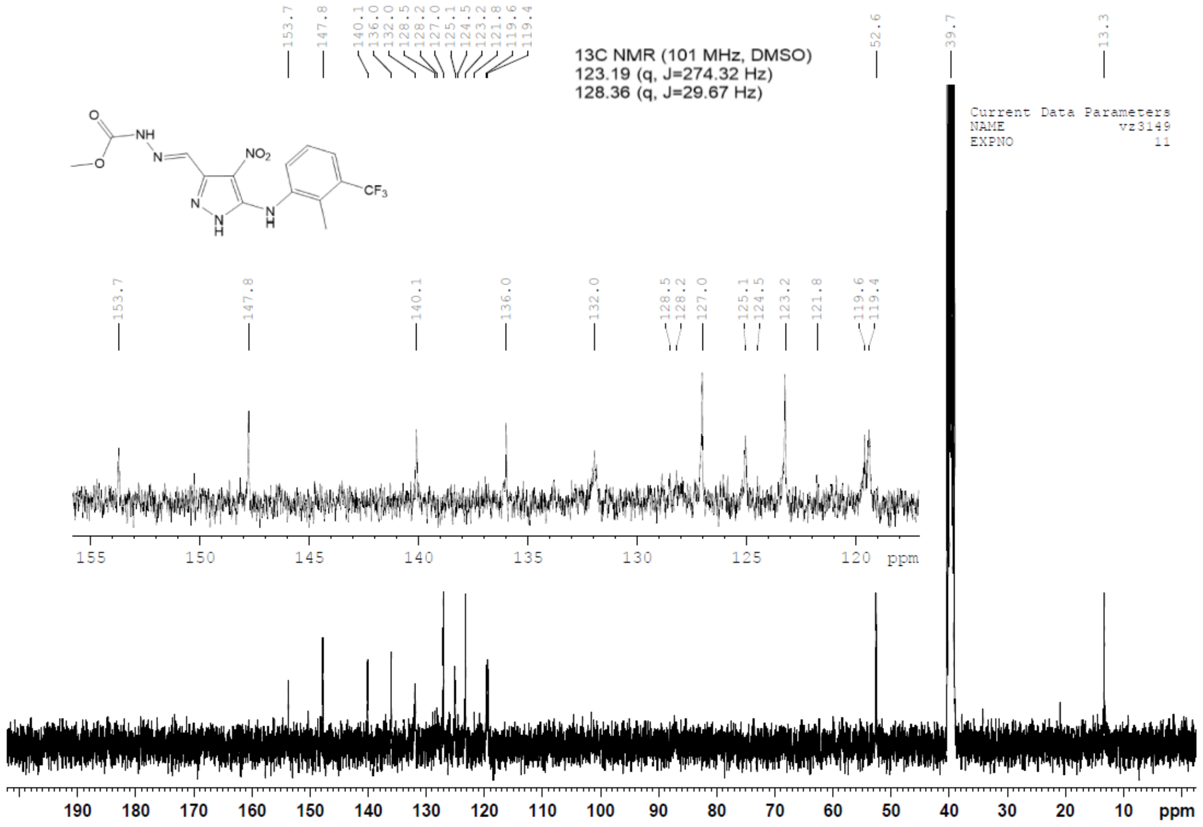

Figure S37-S38. ^{1}H and ^{13}C NMR spectra of compound **3j**

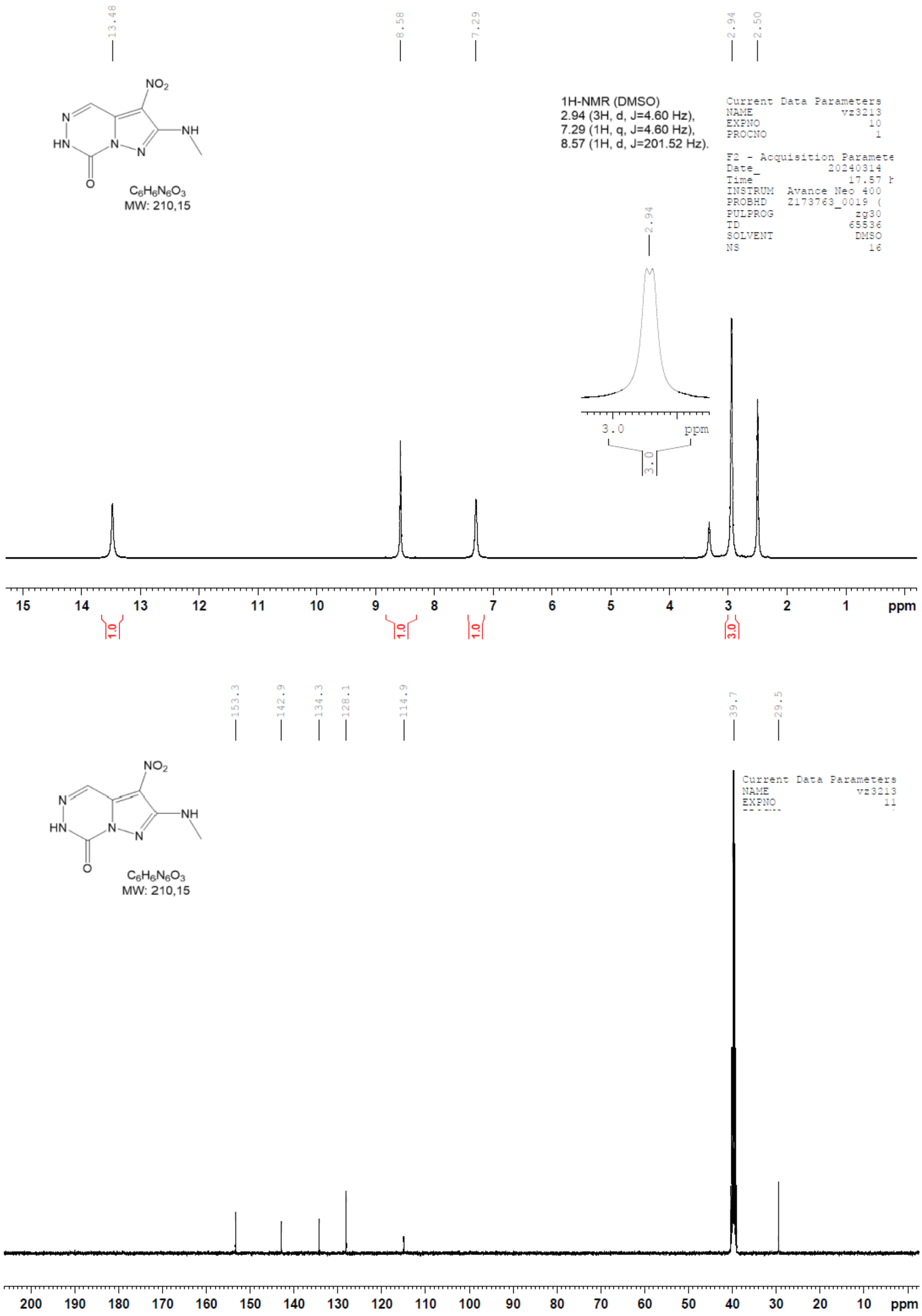

Figure S39-S40. ^{1}H and ^{13}C NMR spectra of compound **4a**

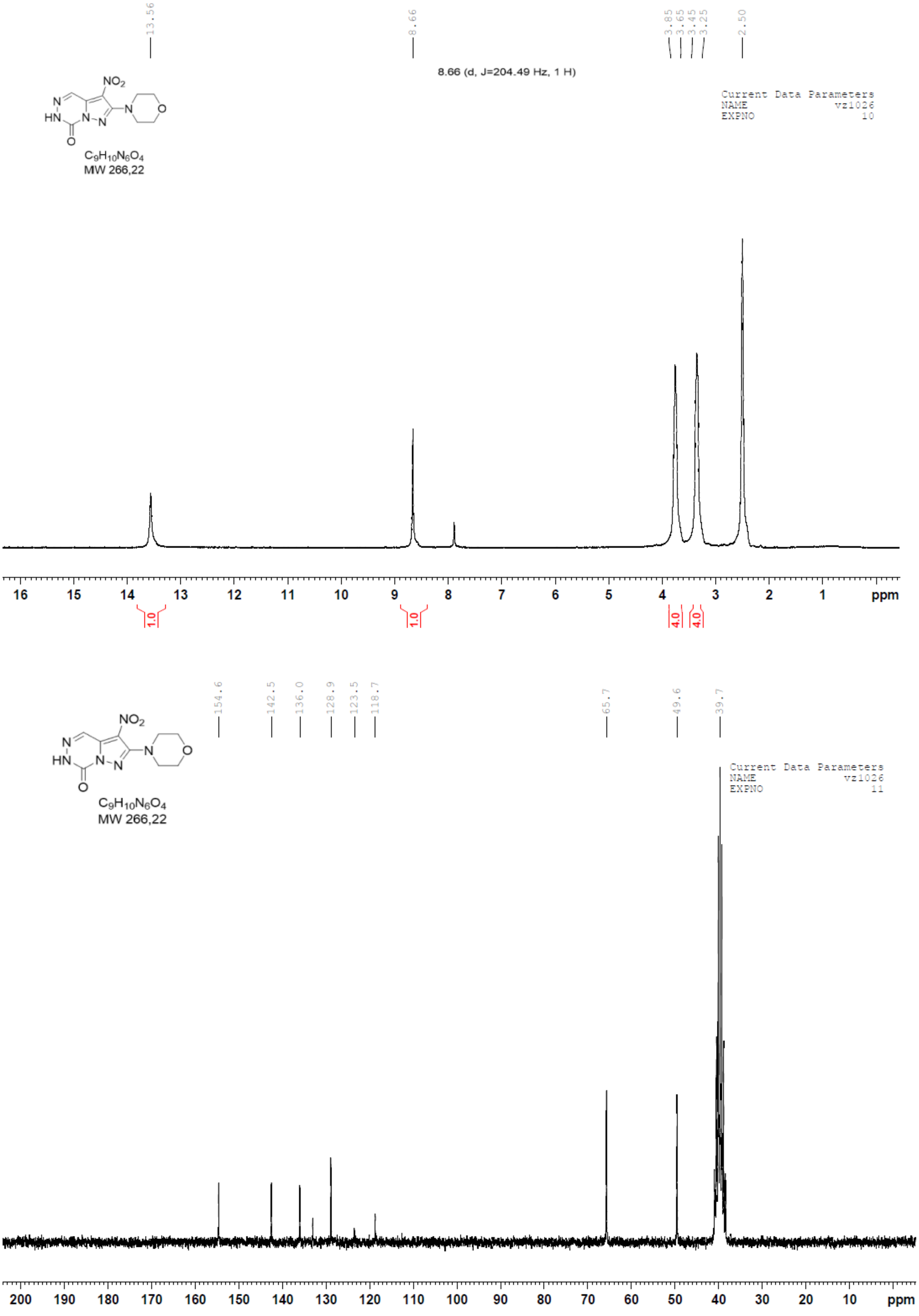

Figure S41-S42. ^{1}H and ^{13}C NMR spectra of compound **4b**

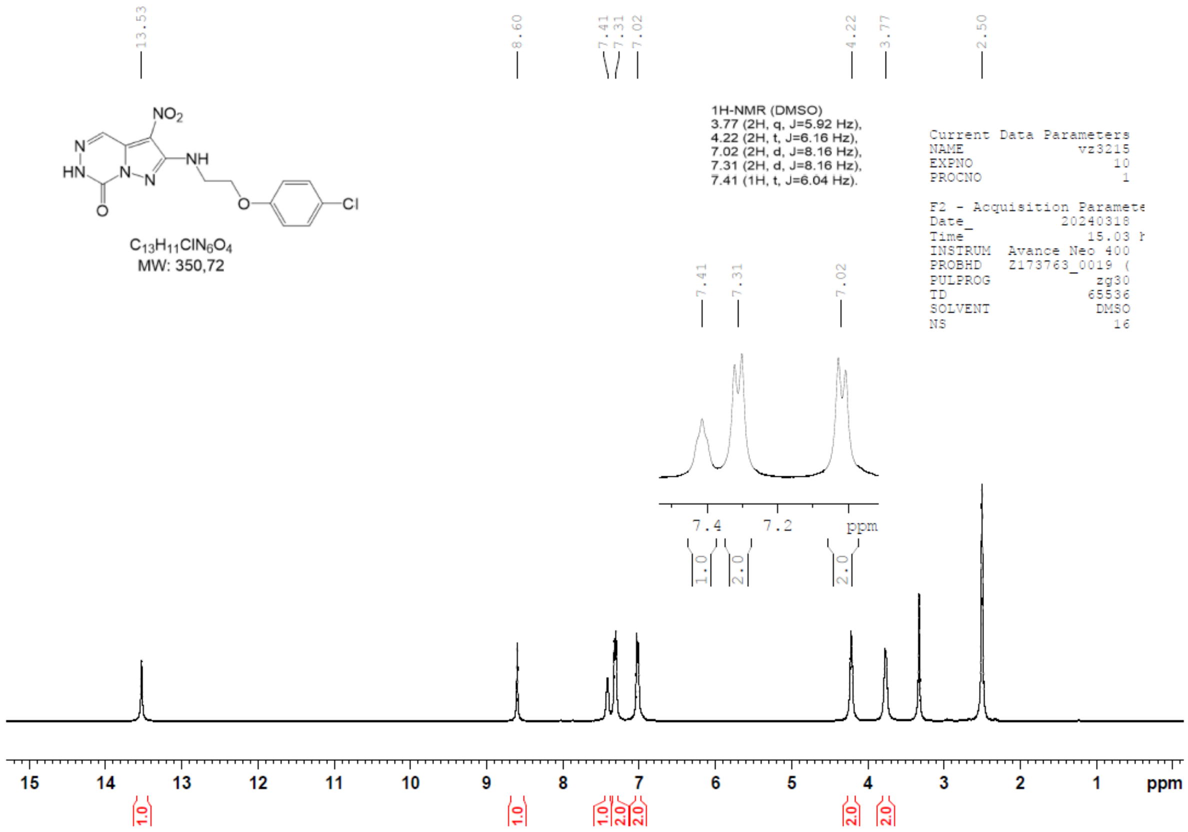

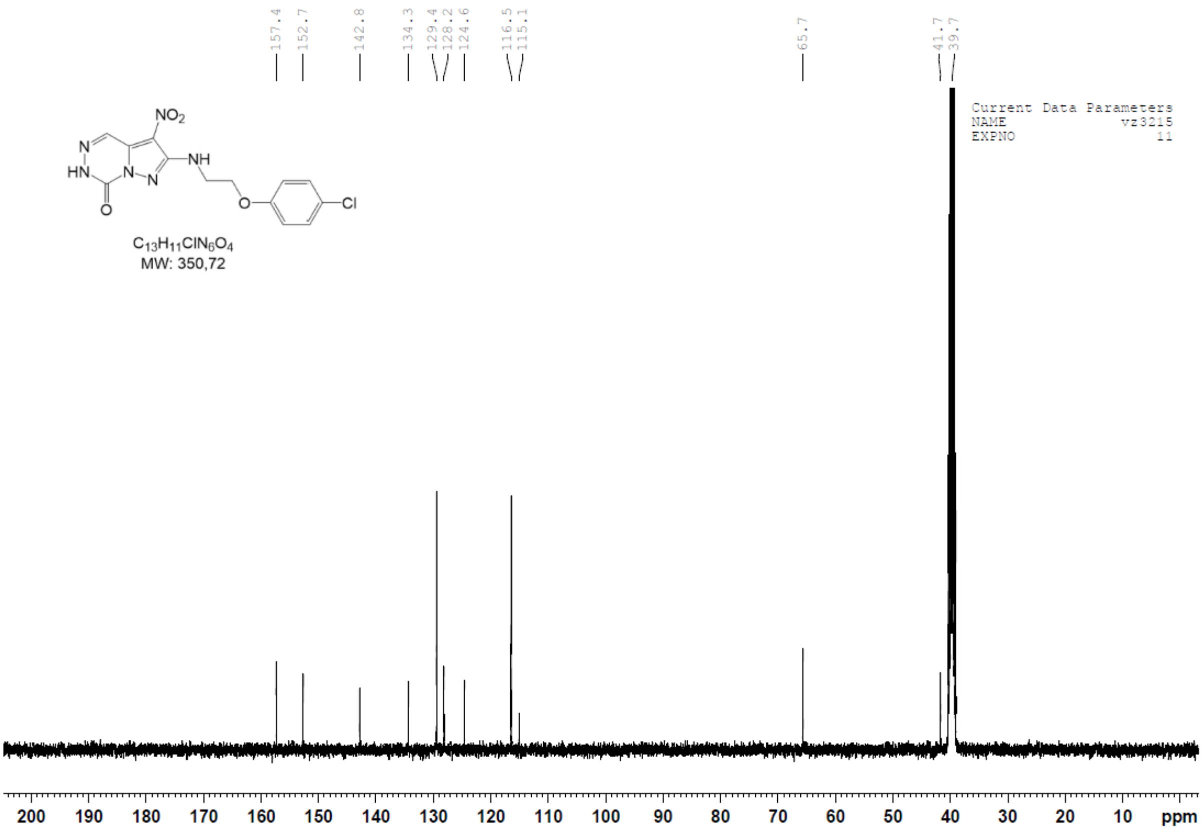

Figure S43-S44. ^{1}H and ^{13}C NMR spectra of compound **4c**

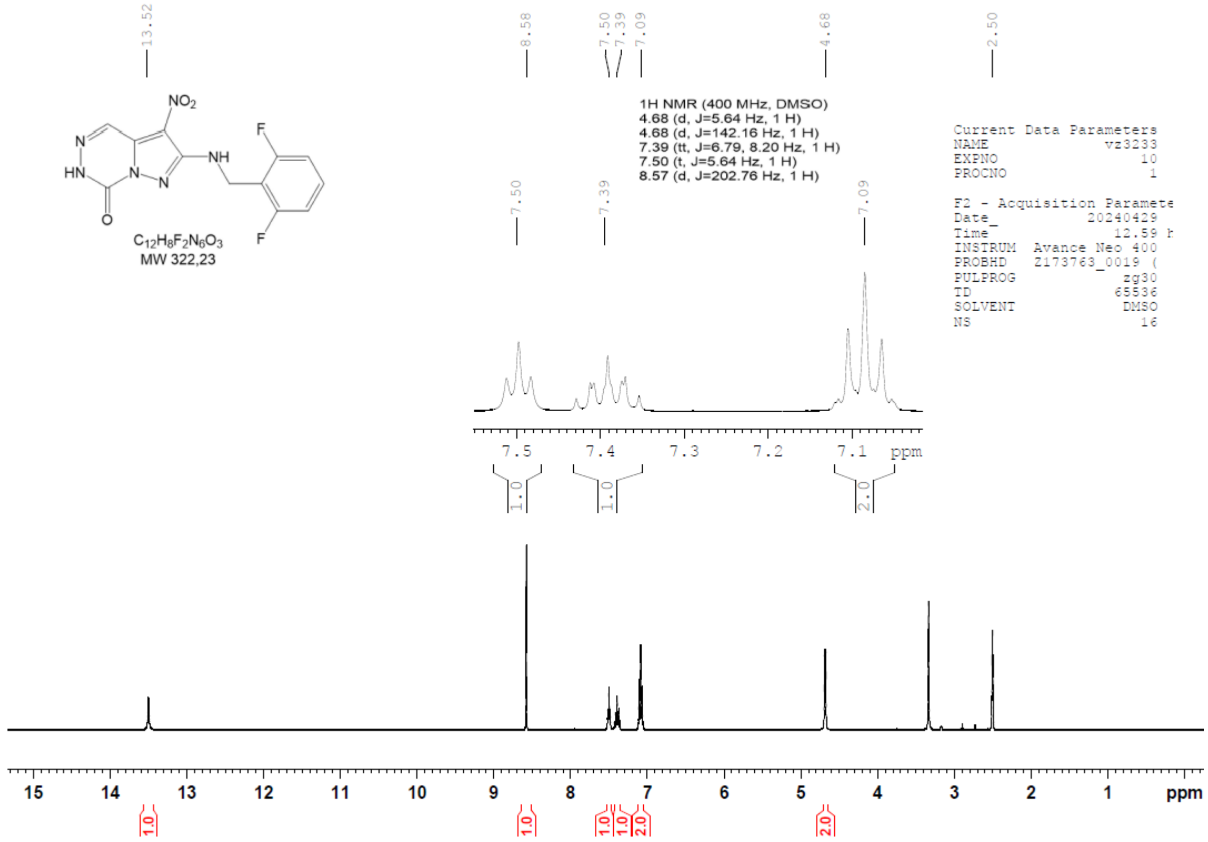

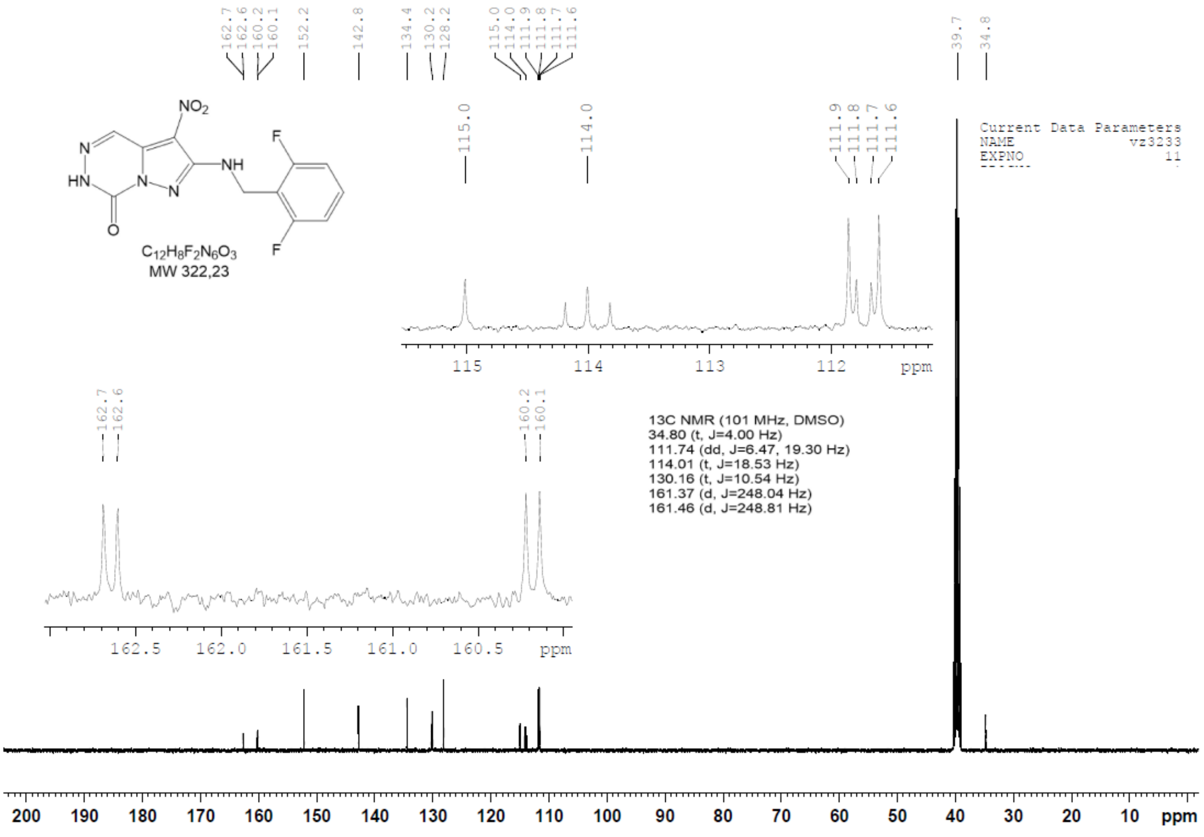

Figure S45-S46. ^{1}H and ^{13}C NMR spectra of compound **4d**

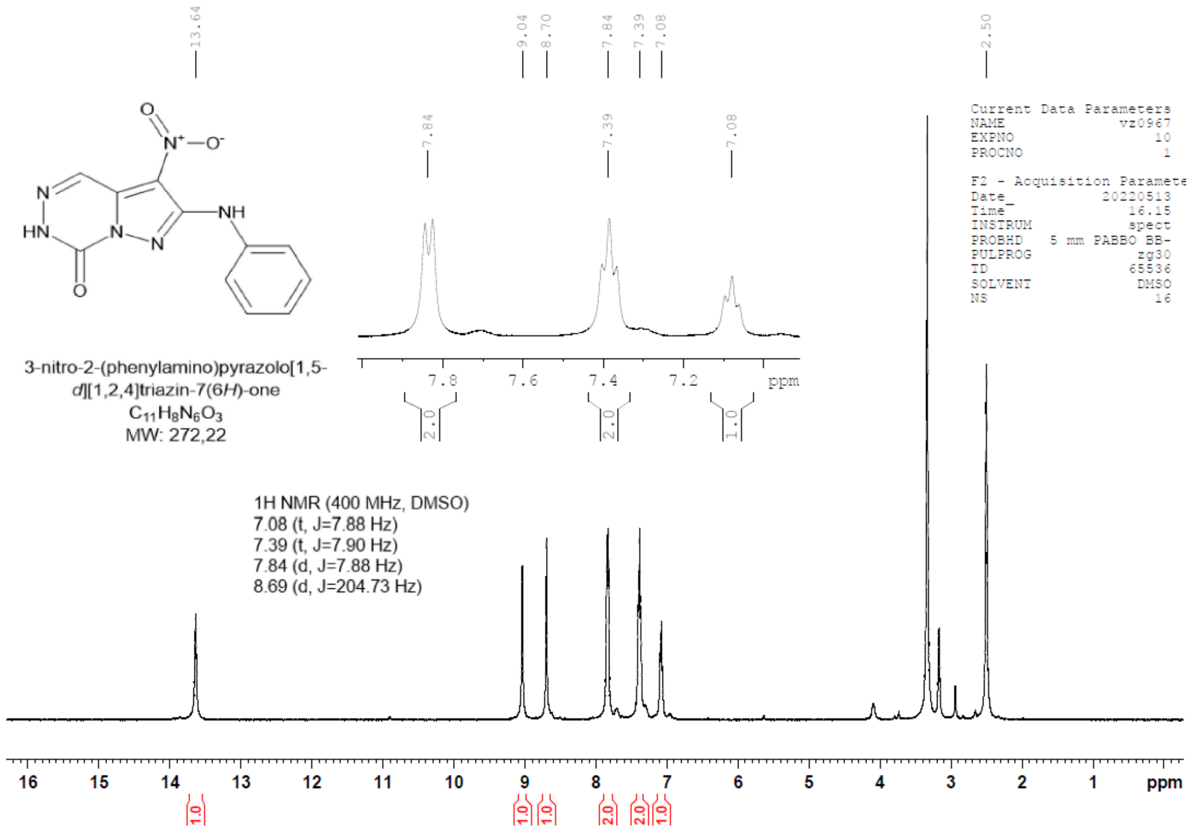

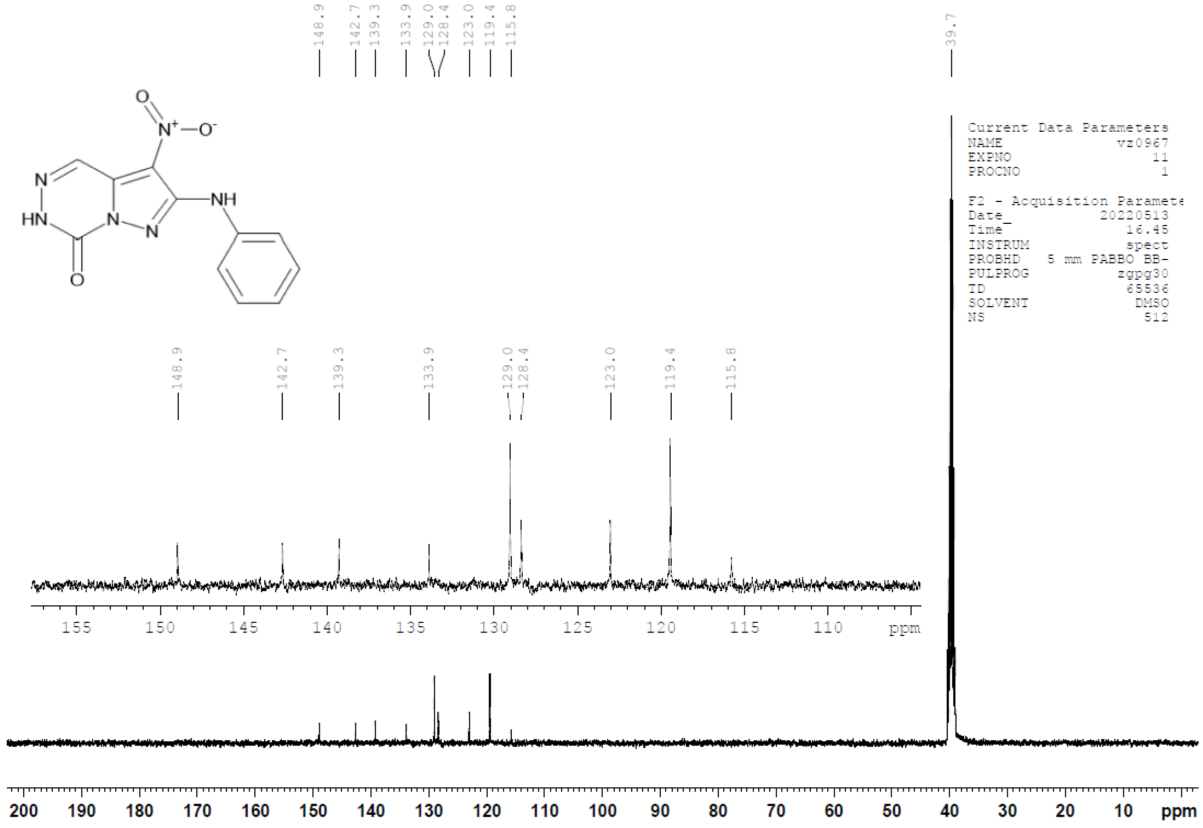

Figure S47-S48. ^{1}H and ^{13}C NMR spectra of compound **4e**

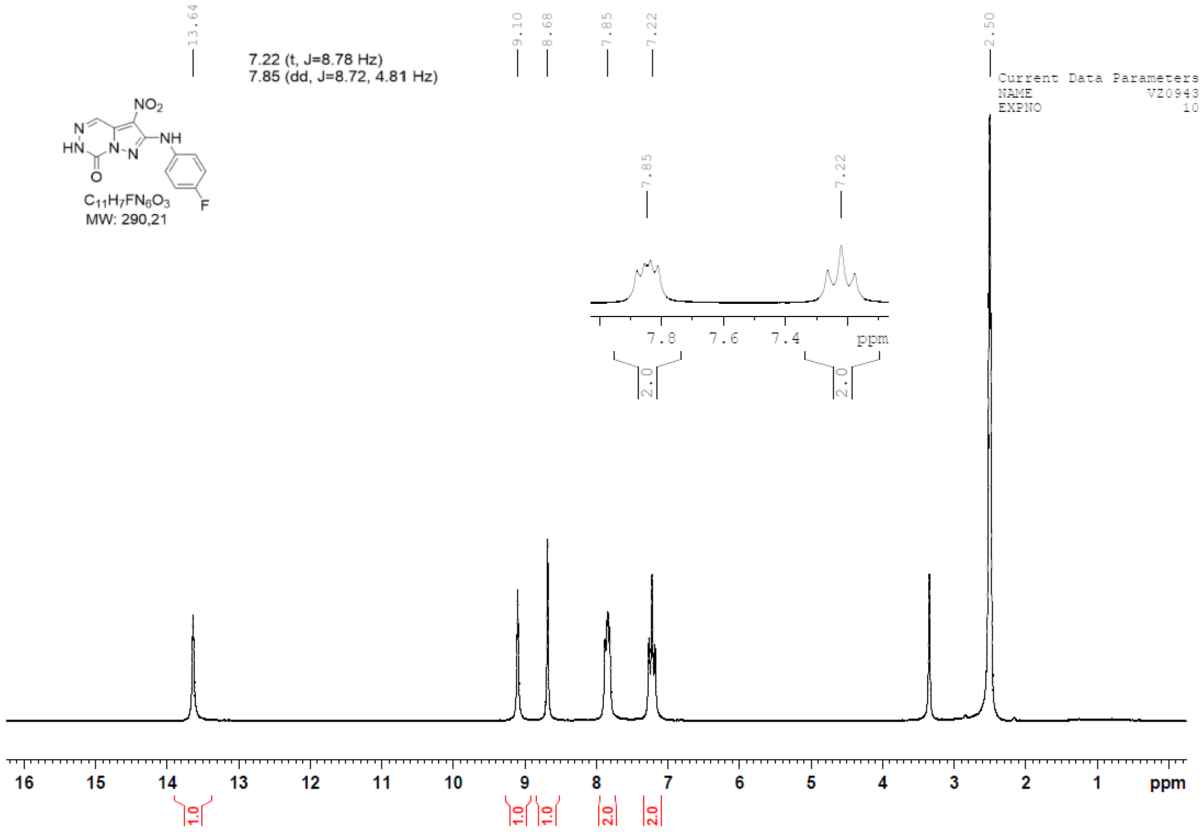

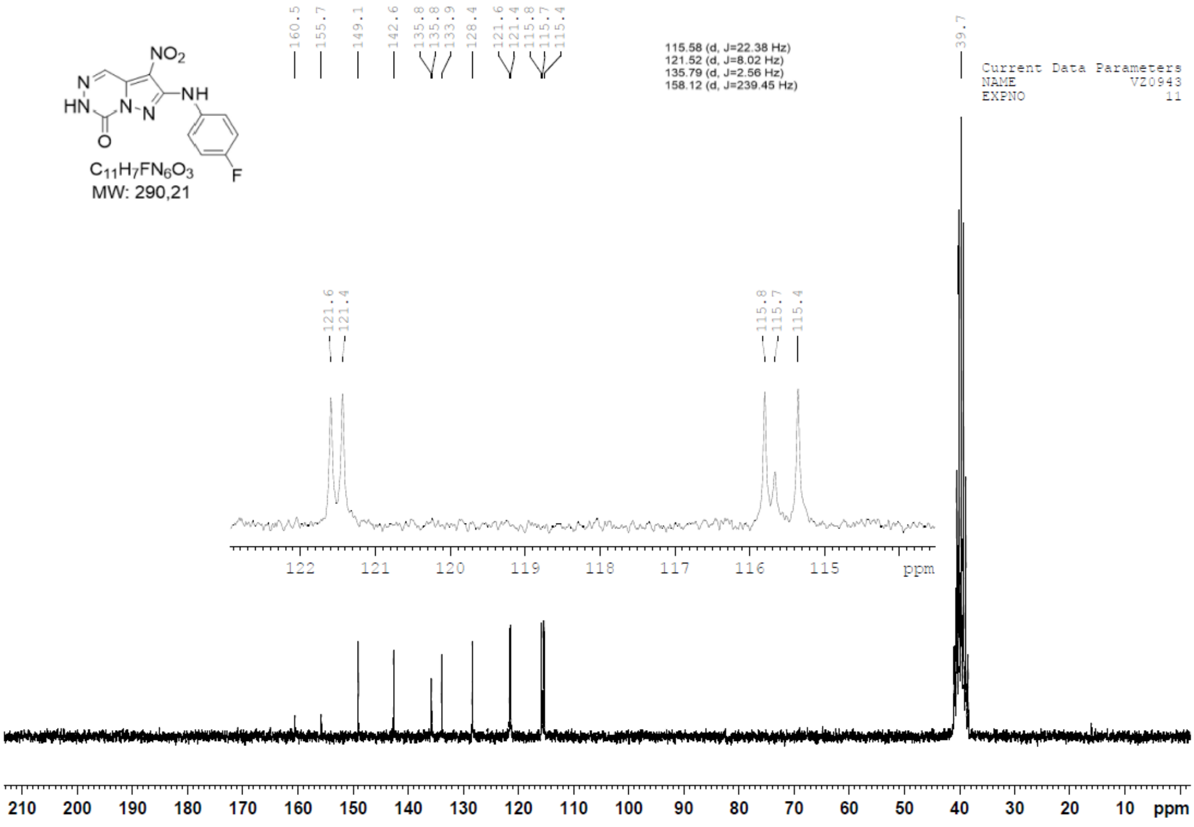

Figure S49-S50. ^{1}H and ^{13}C NMR spectra of compound **4f**

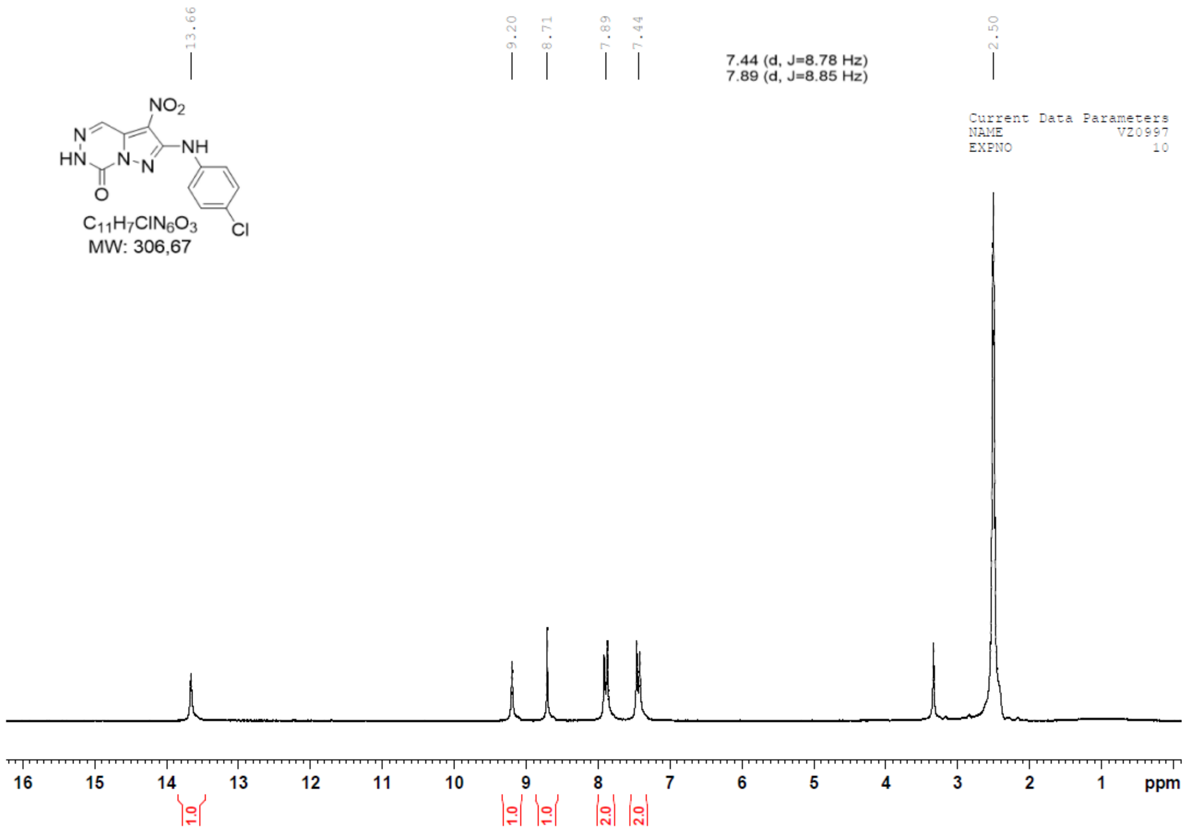

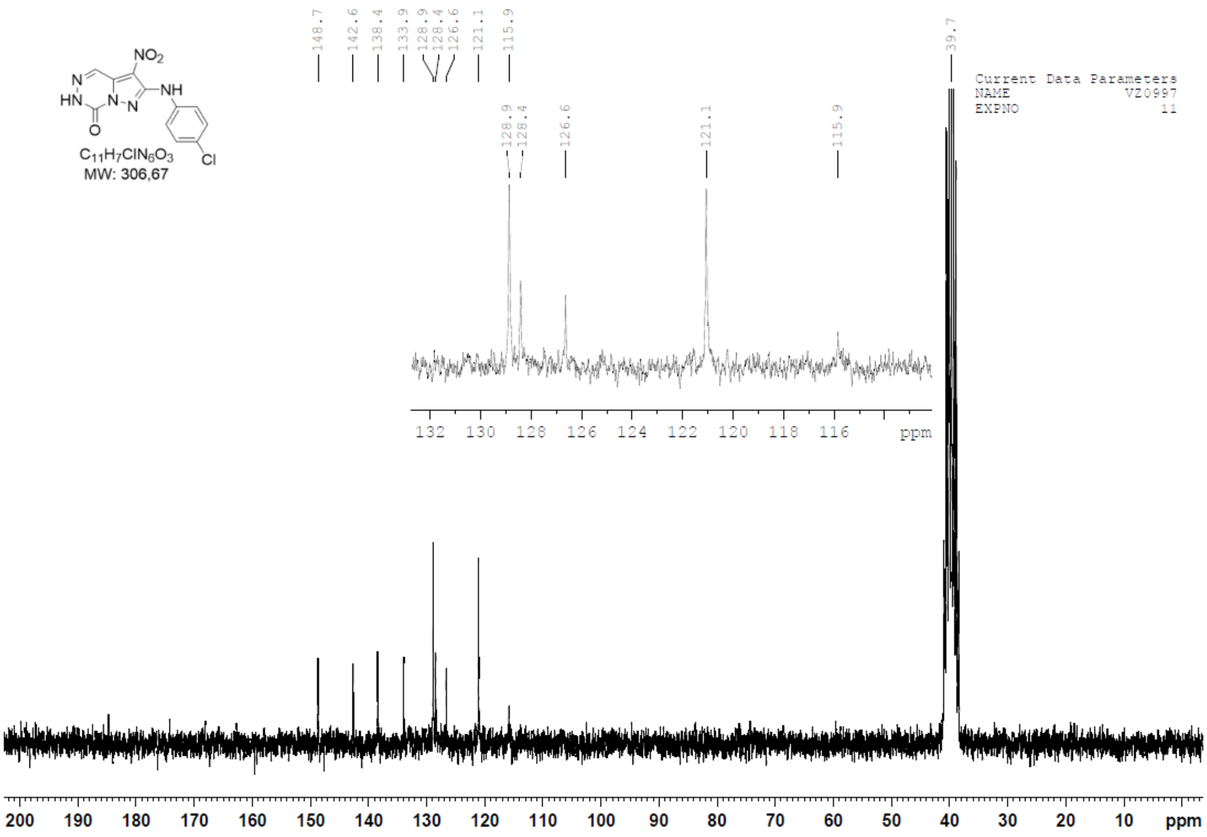

Figure S51-S52. ^{1}H and ^{13}C NMR spectra of compound **4g**

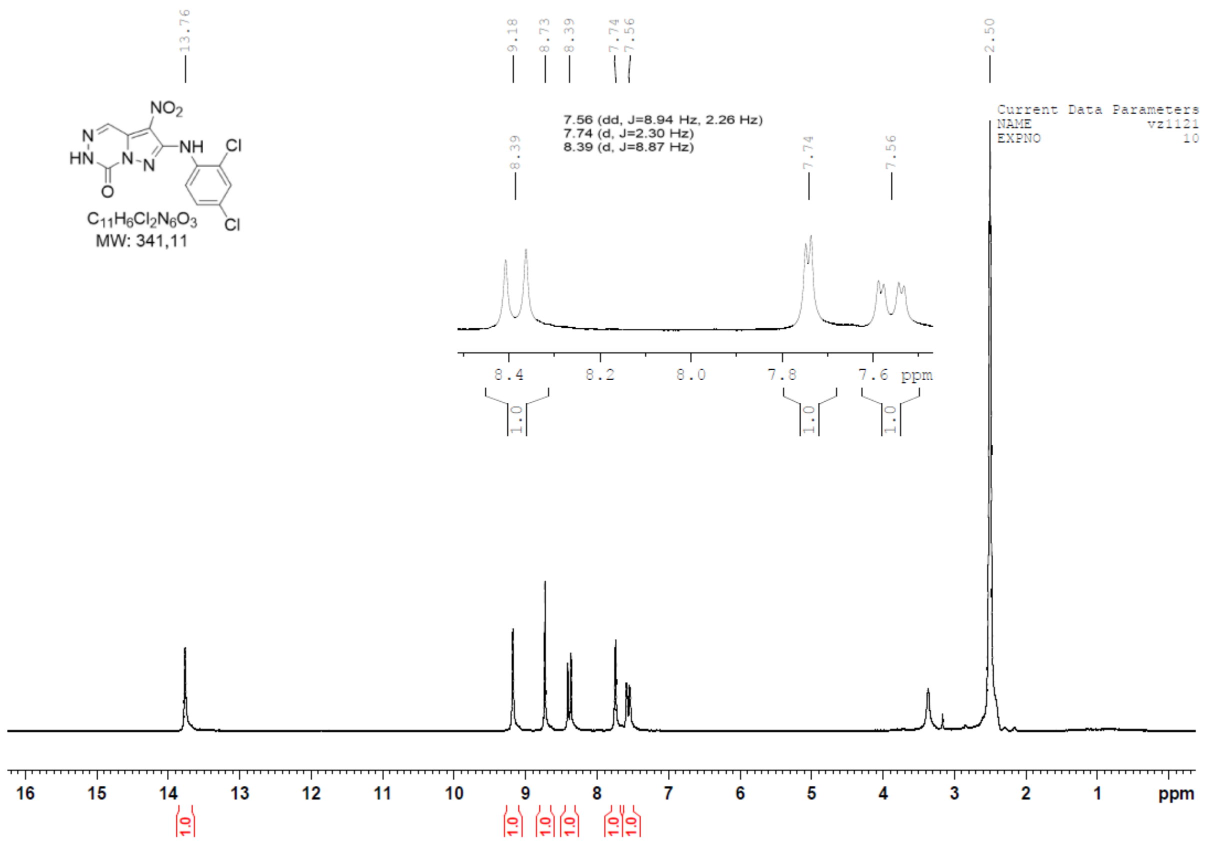

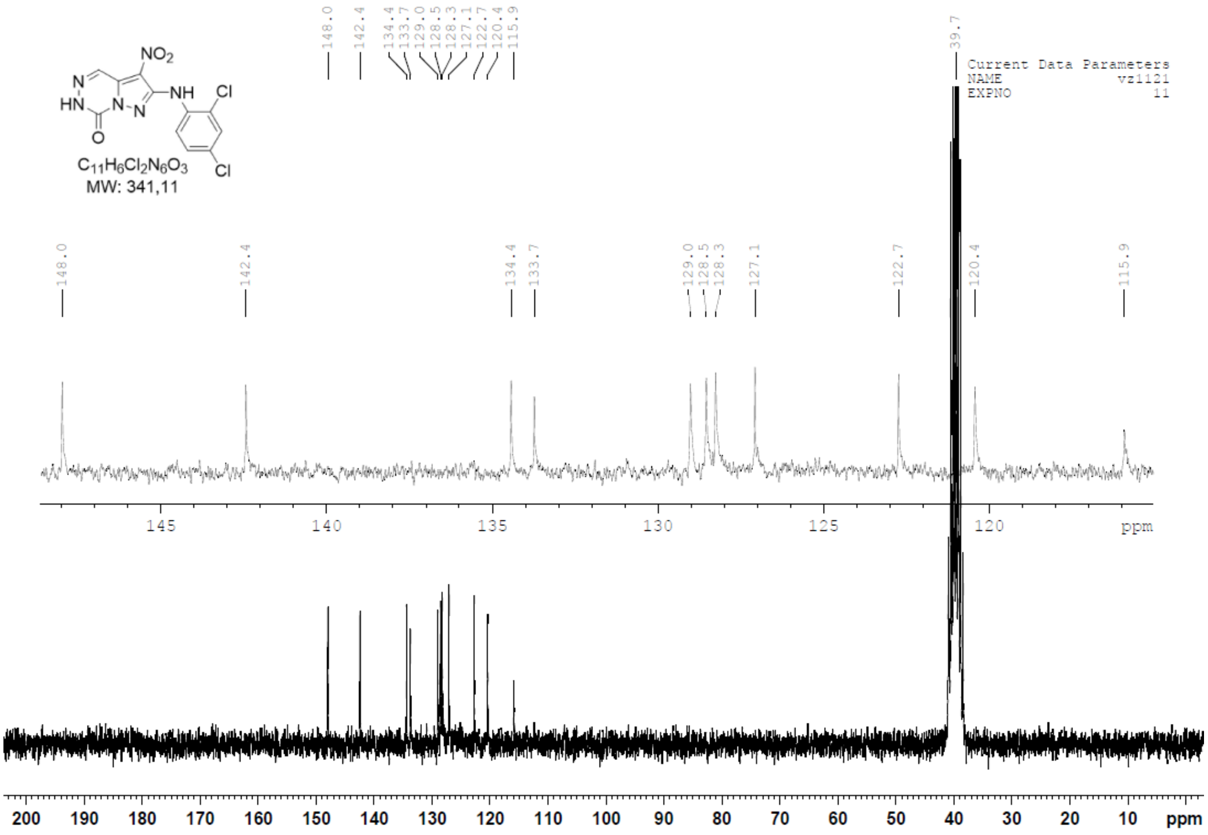

Figure S53-S54. ^{1}H and ^{13}C NMR spectra of compound **4i**

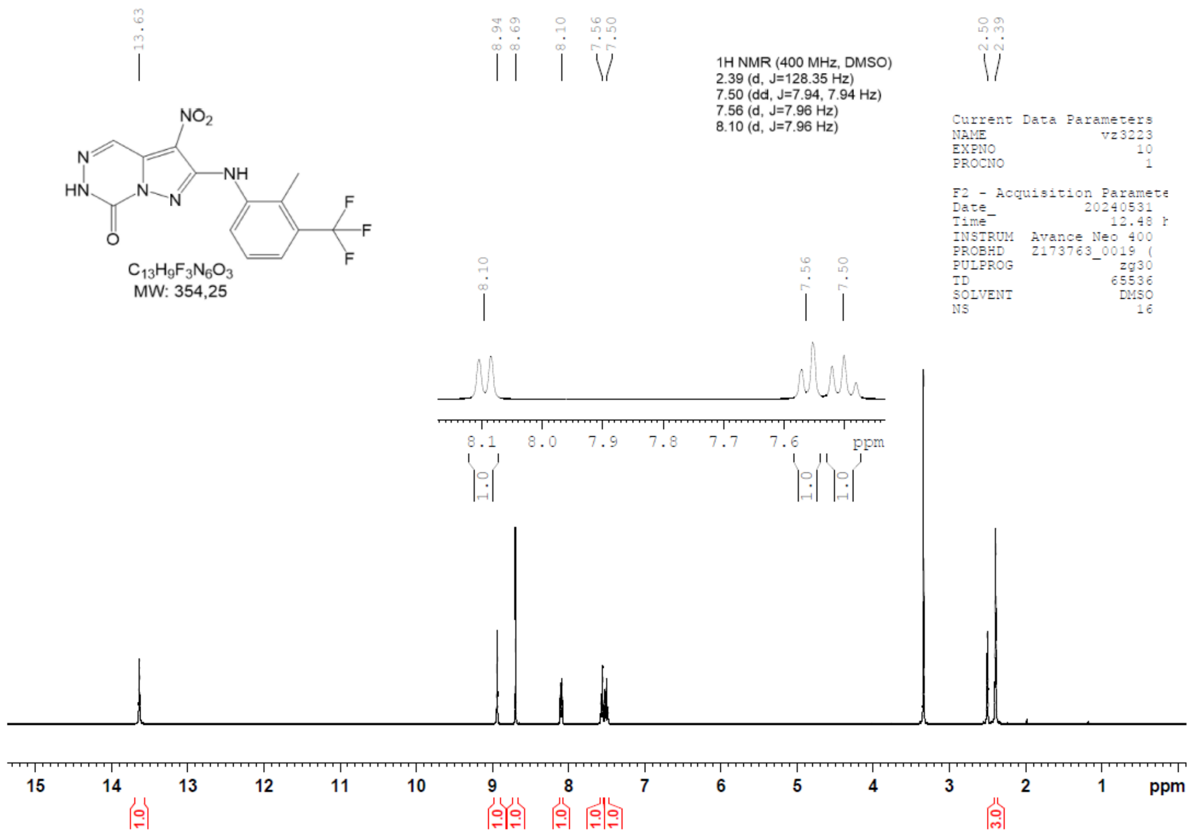

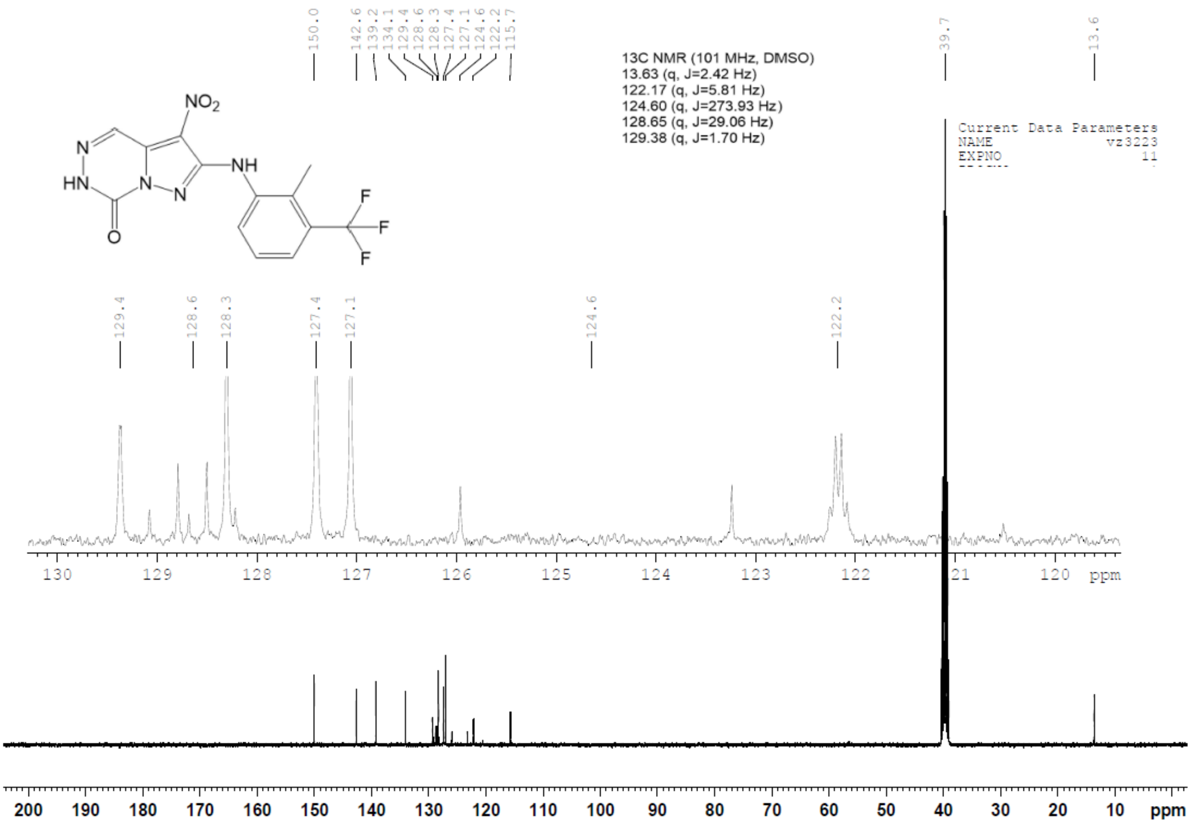

Figure S55-S56. ^{1}H and ^{13}C NMR spectra of compound **4j**

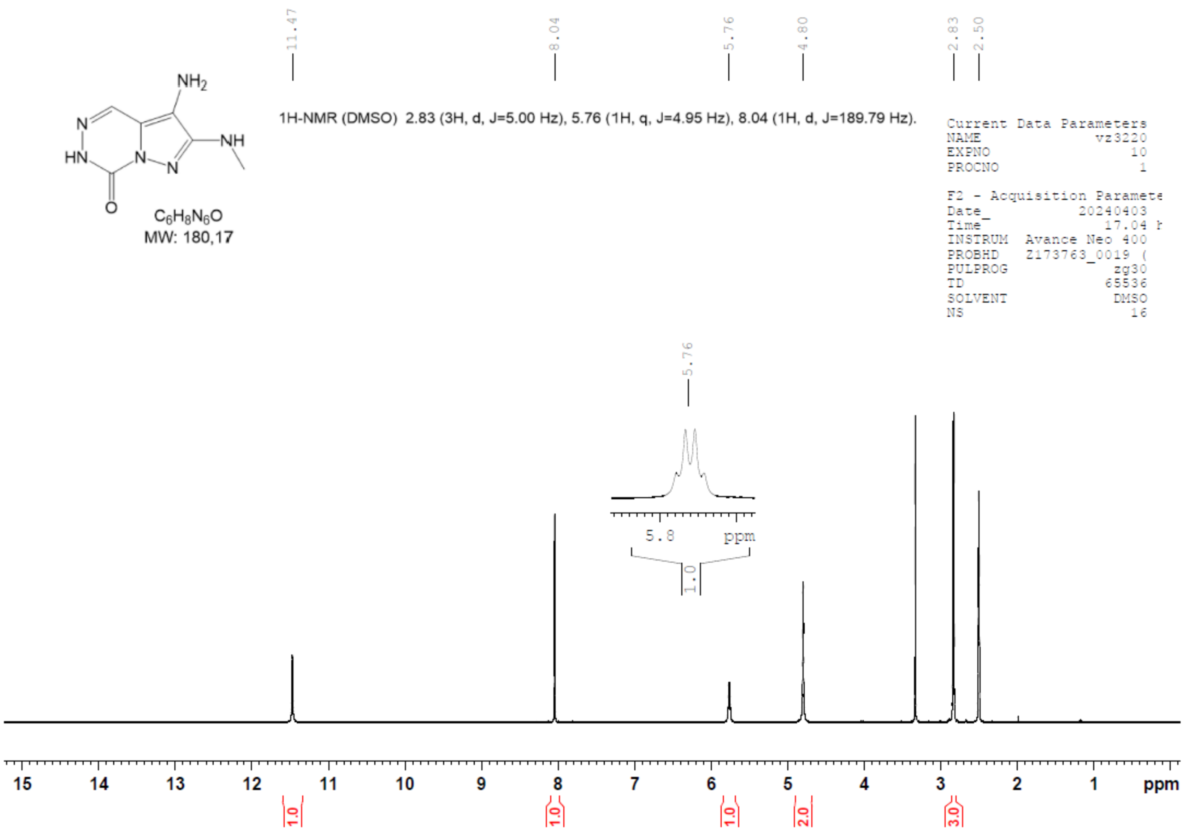

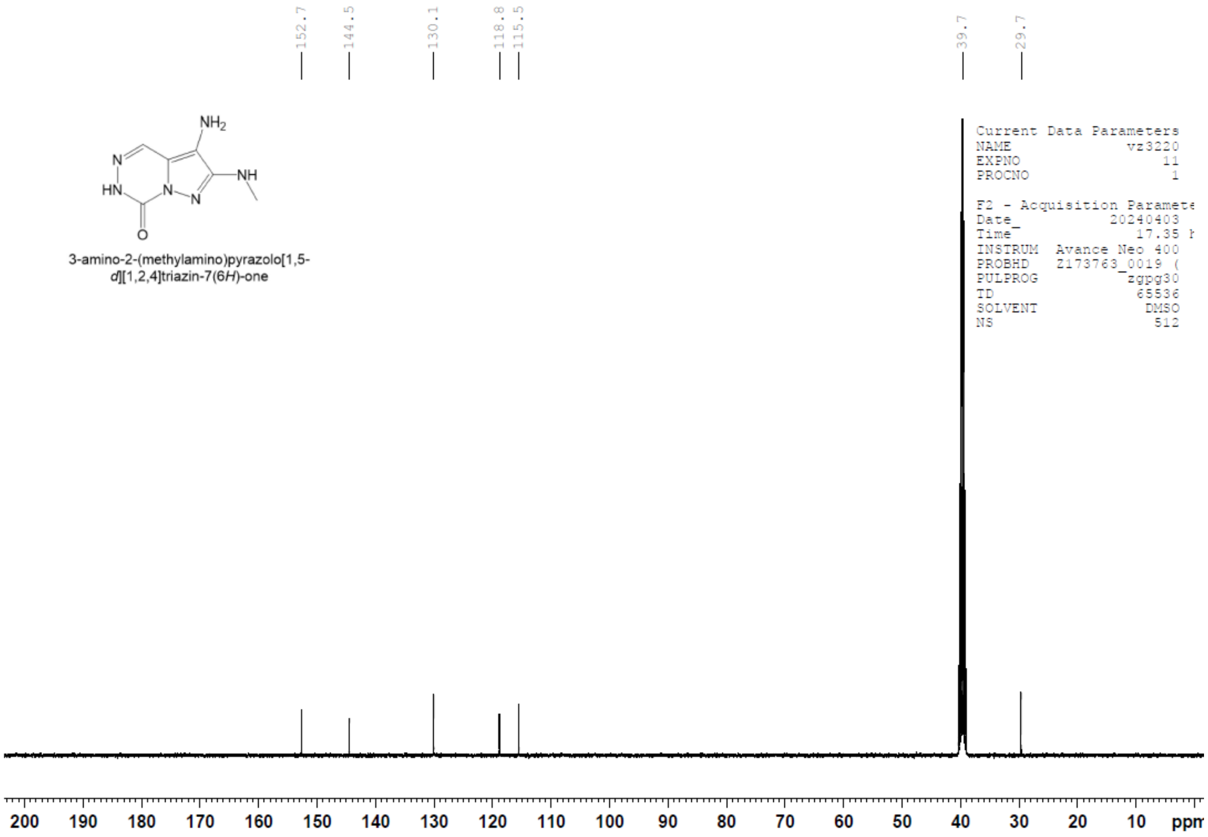

Figure S57-S58. ^{1}H and ^{13}C NMR spectra of compound **5a**

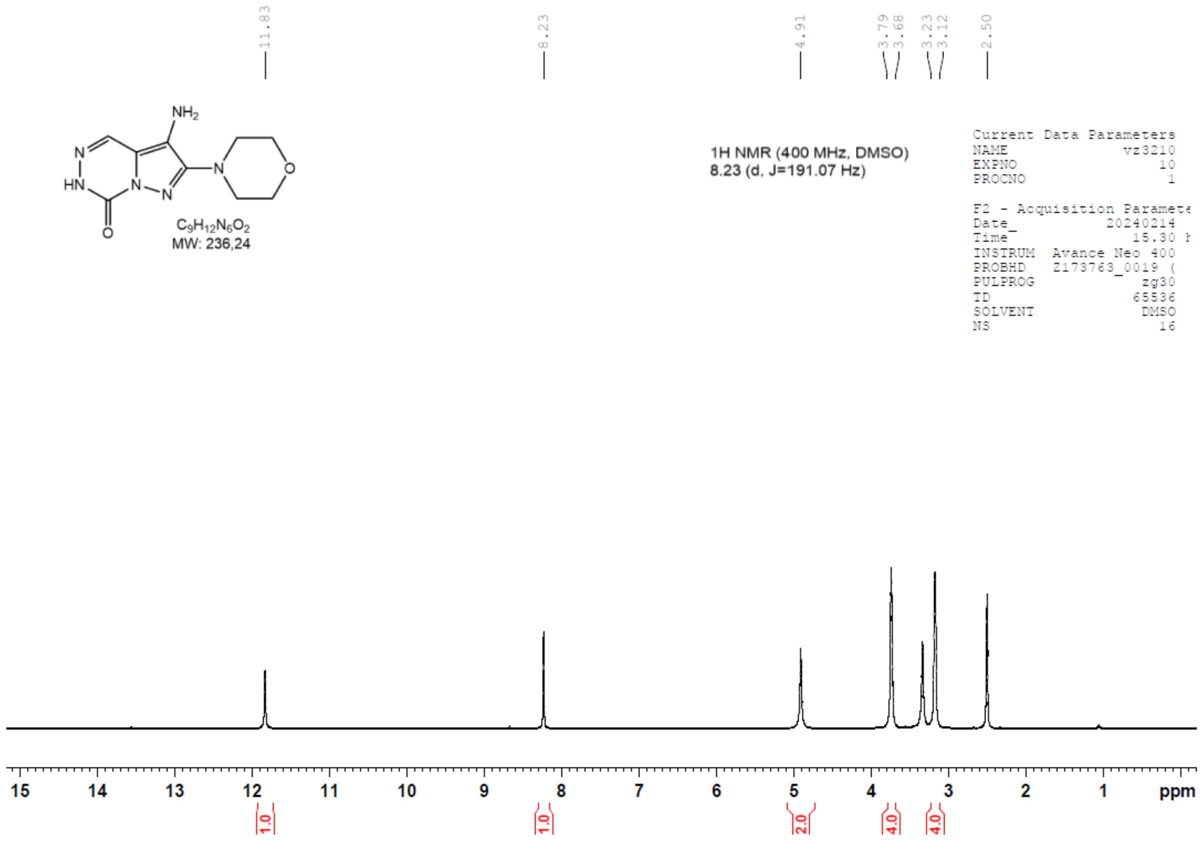

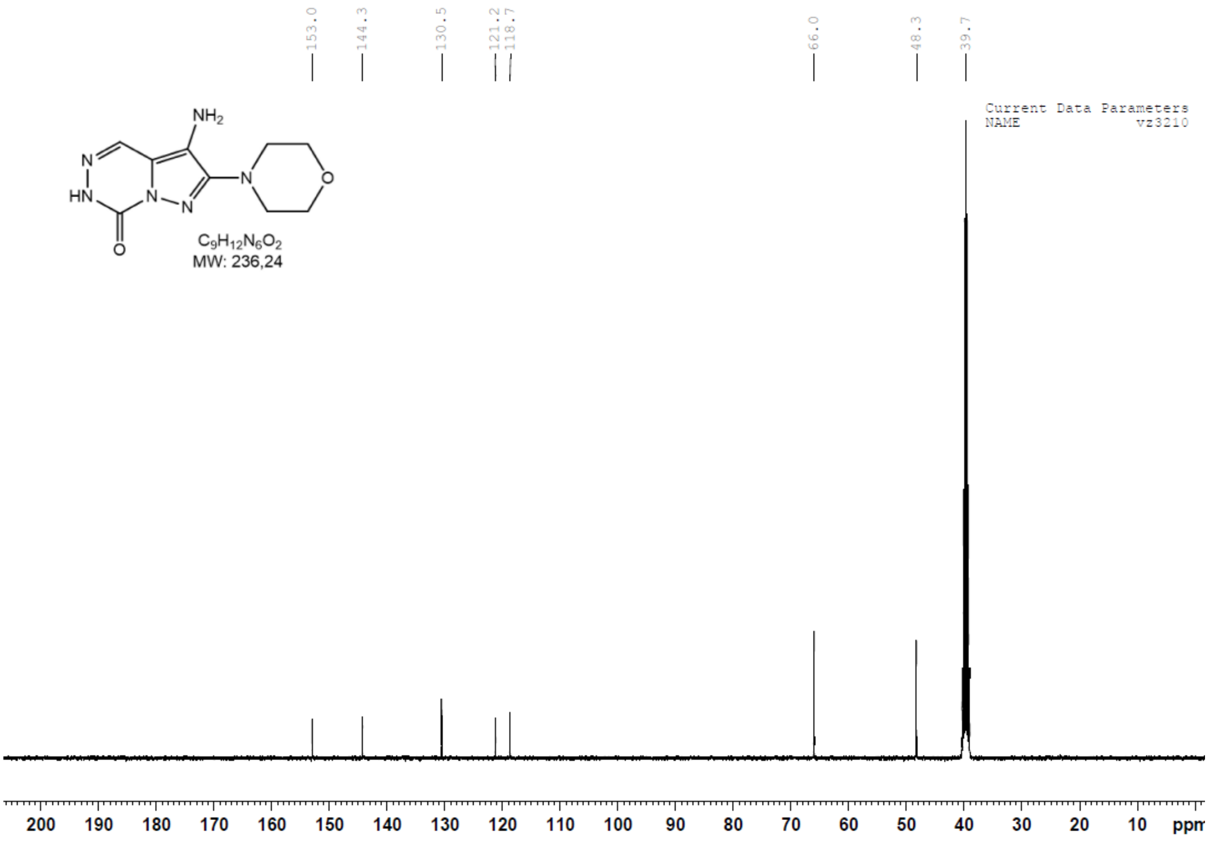

Figure S59-S60. ¹H and ¹³C NMR spectra of compound **5b**

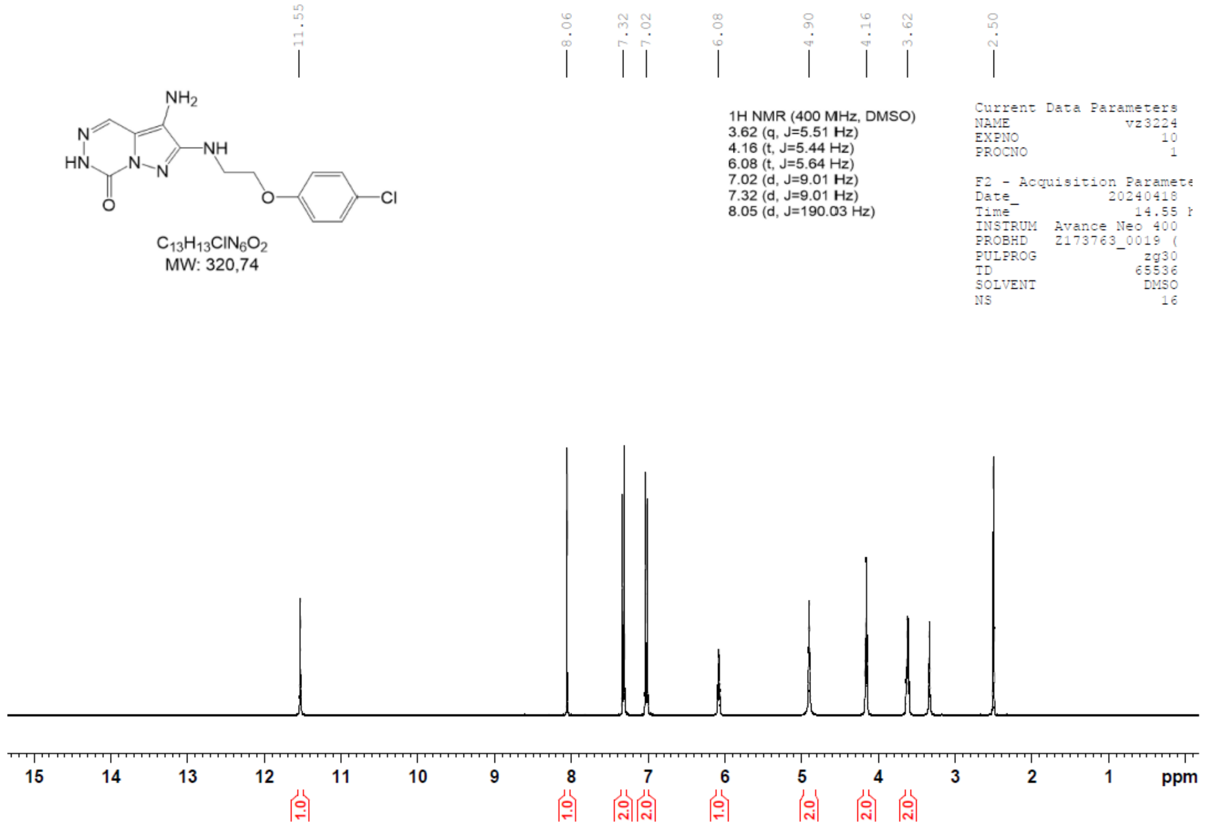

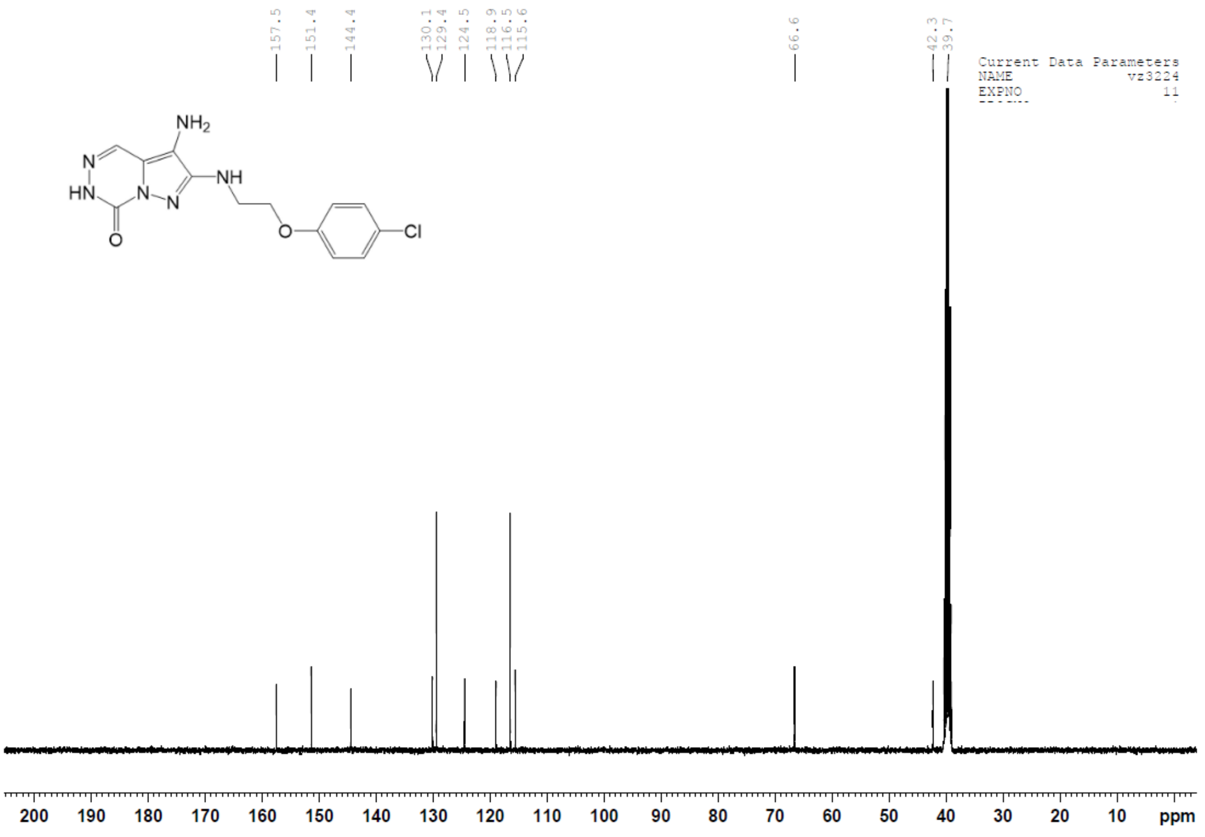

Figure S61-S62. ^{1}H and ^{13}C NMR spectra of compound **5c**

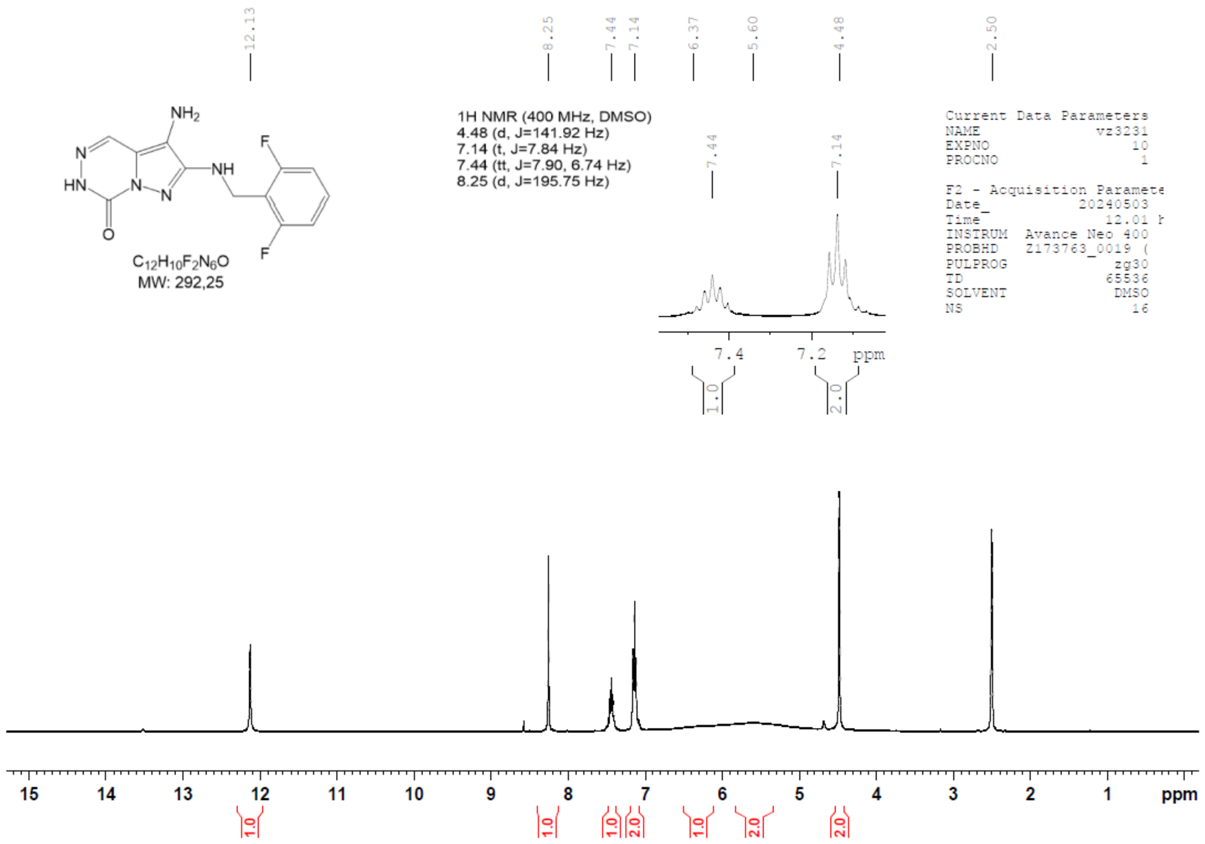

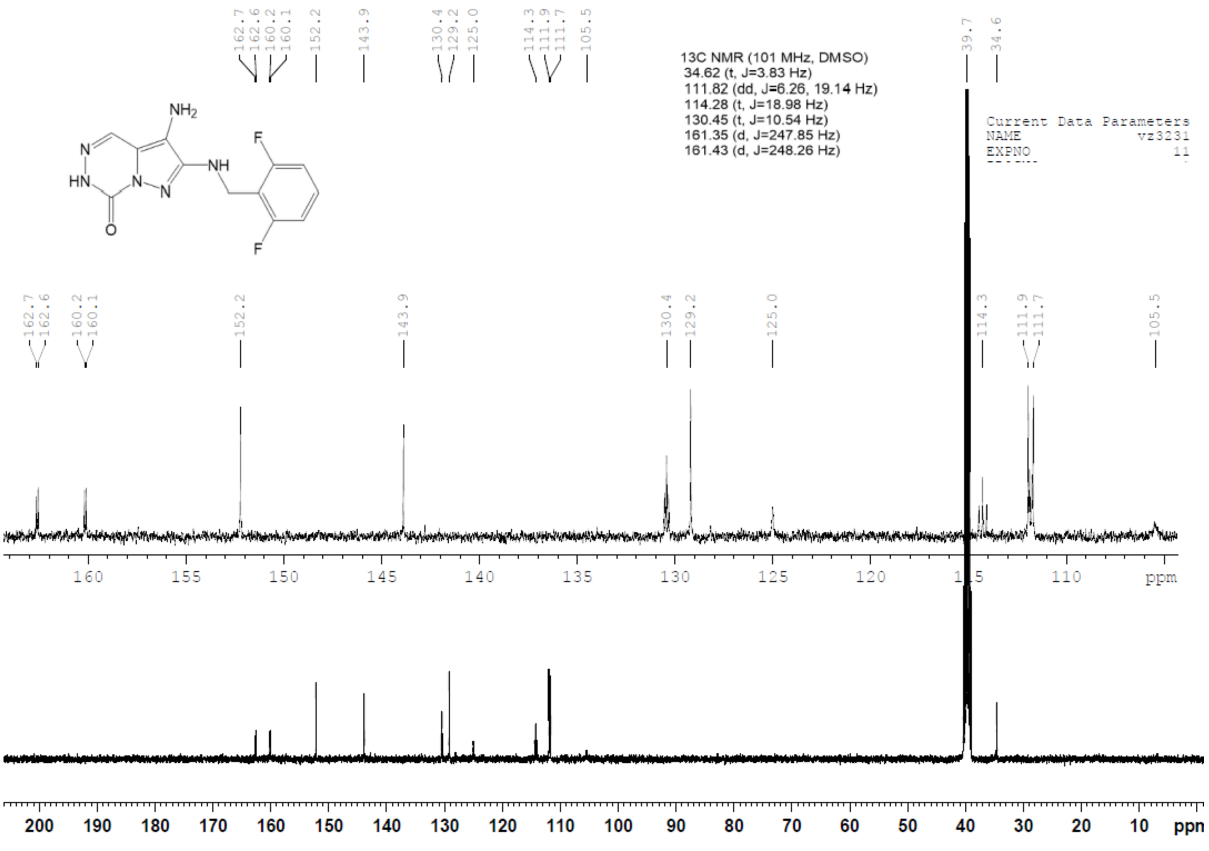

Figure S63-S64. ^{1}H and ^{13}C NMR spectra of compound **5d**

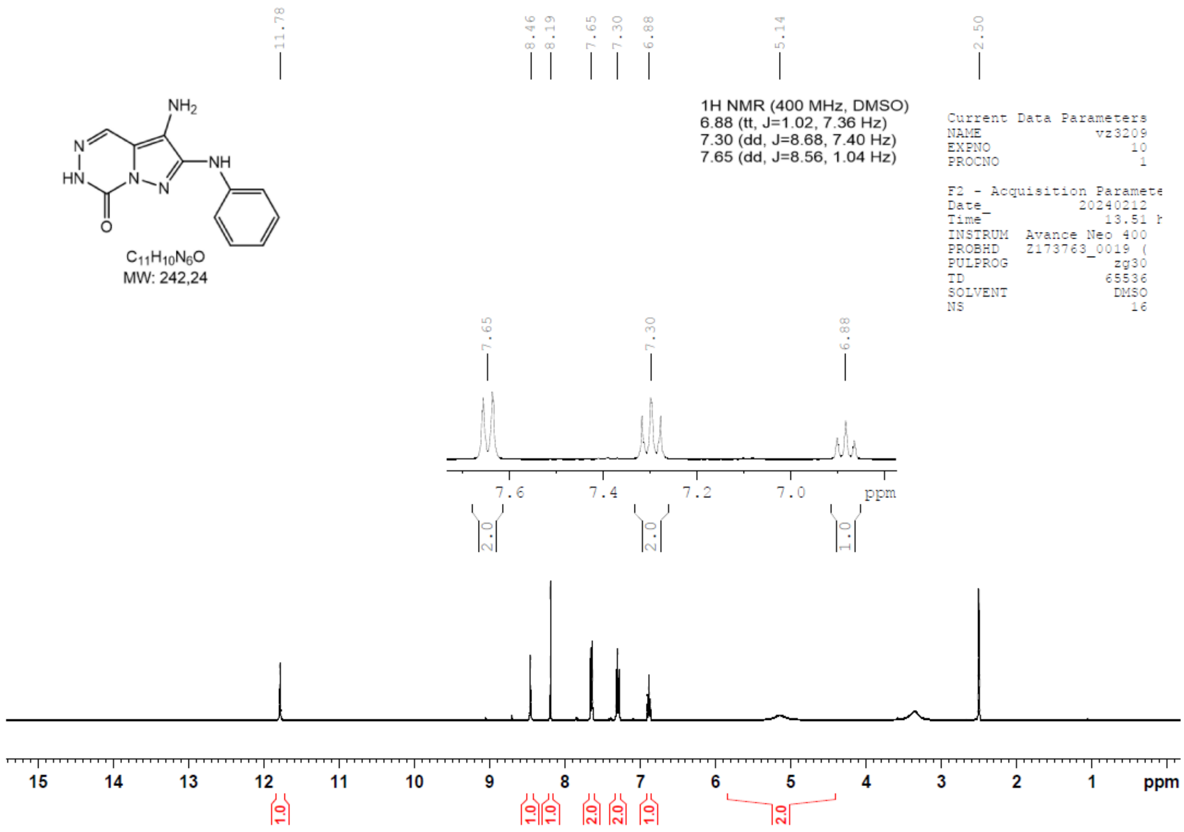

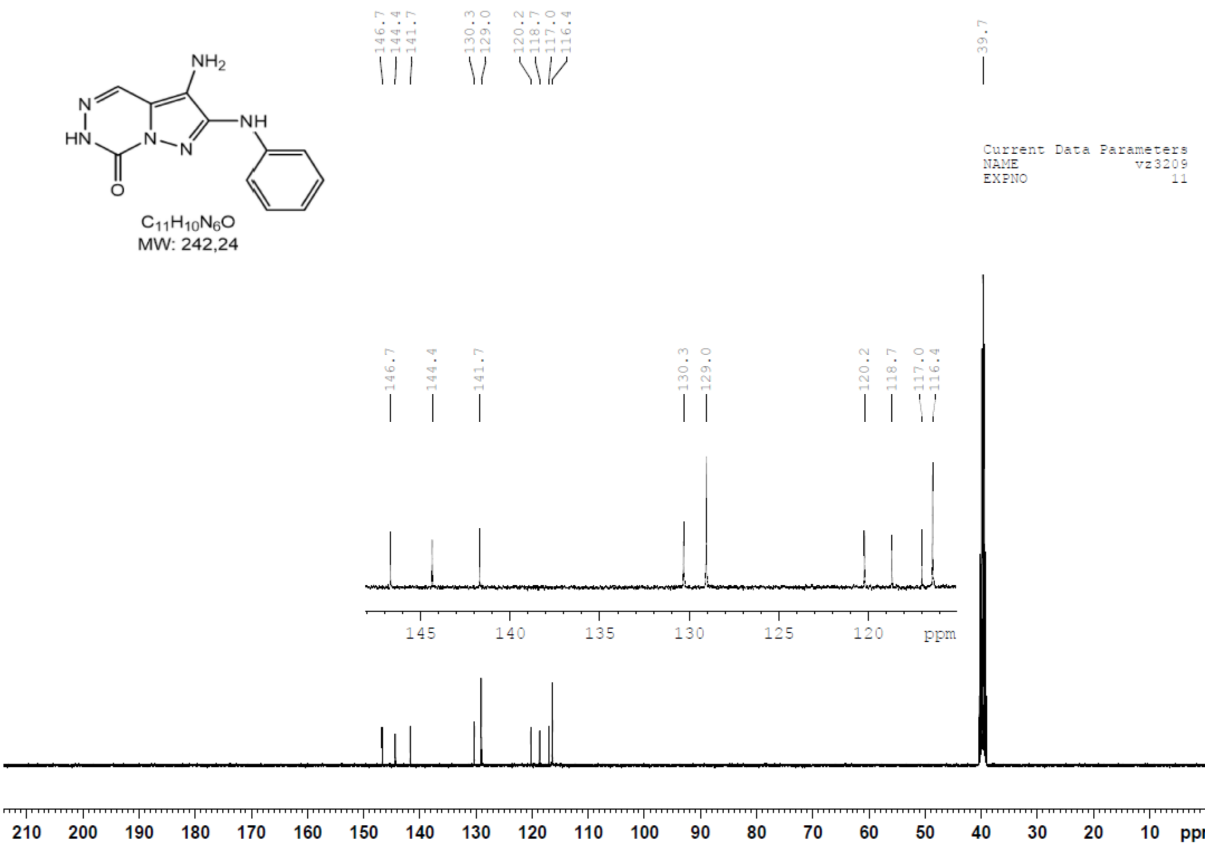

Figure S65-S66. ^{1}H and ^{13}C NMR spectra of compound **5e**

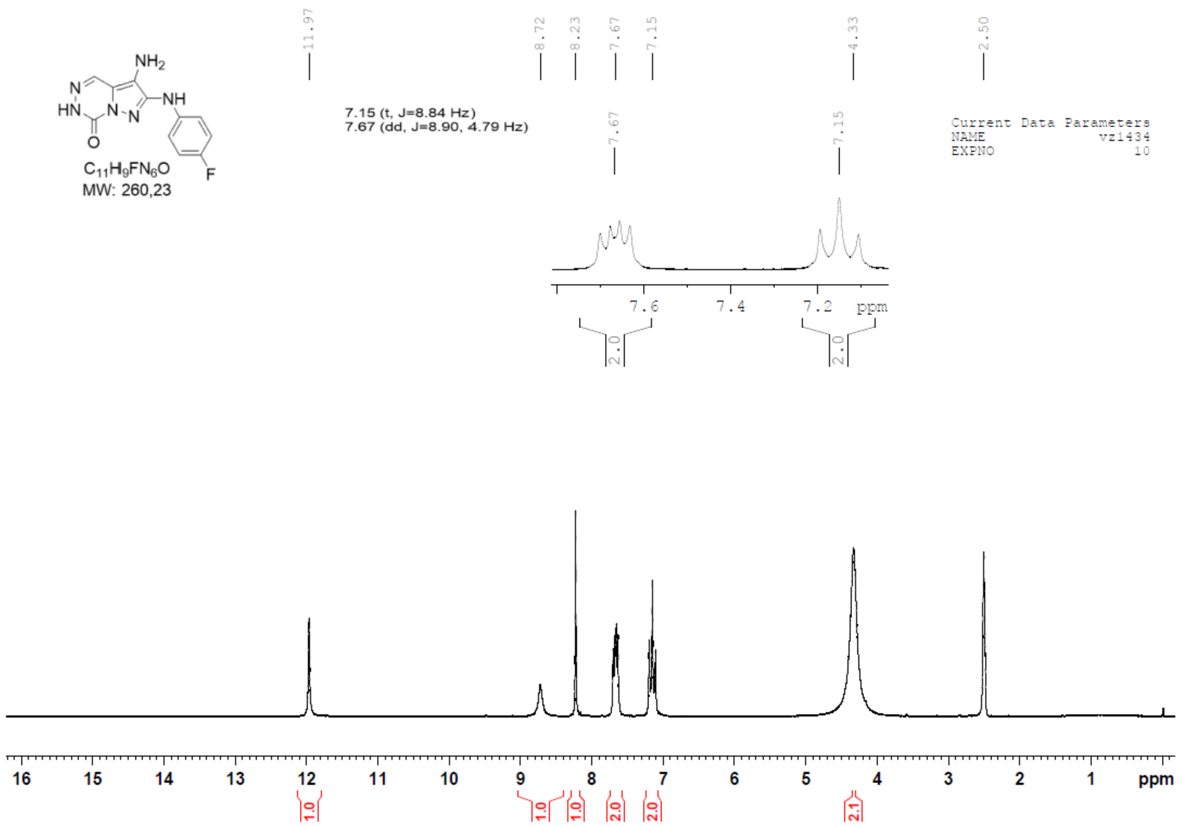

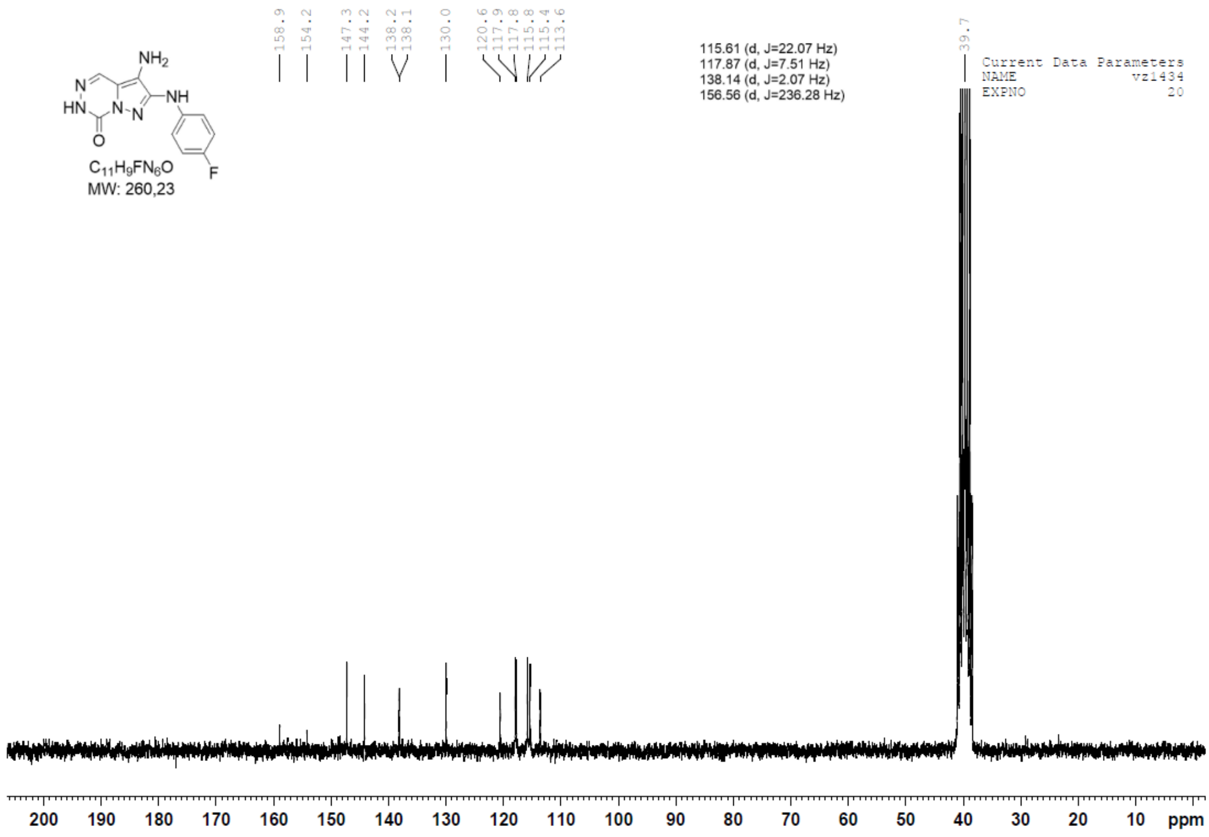

Figure S67-S68. ^{1}H and ^{13}C NMR spectra of compound **5f**

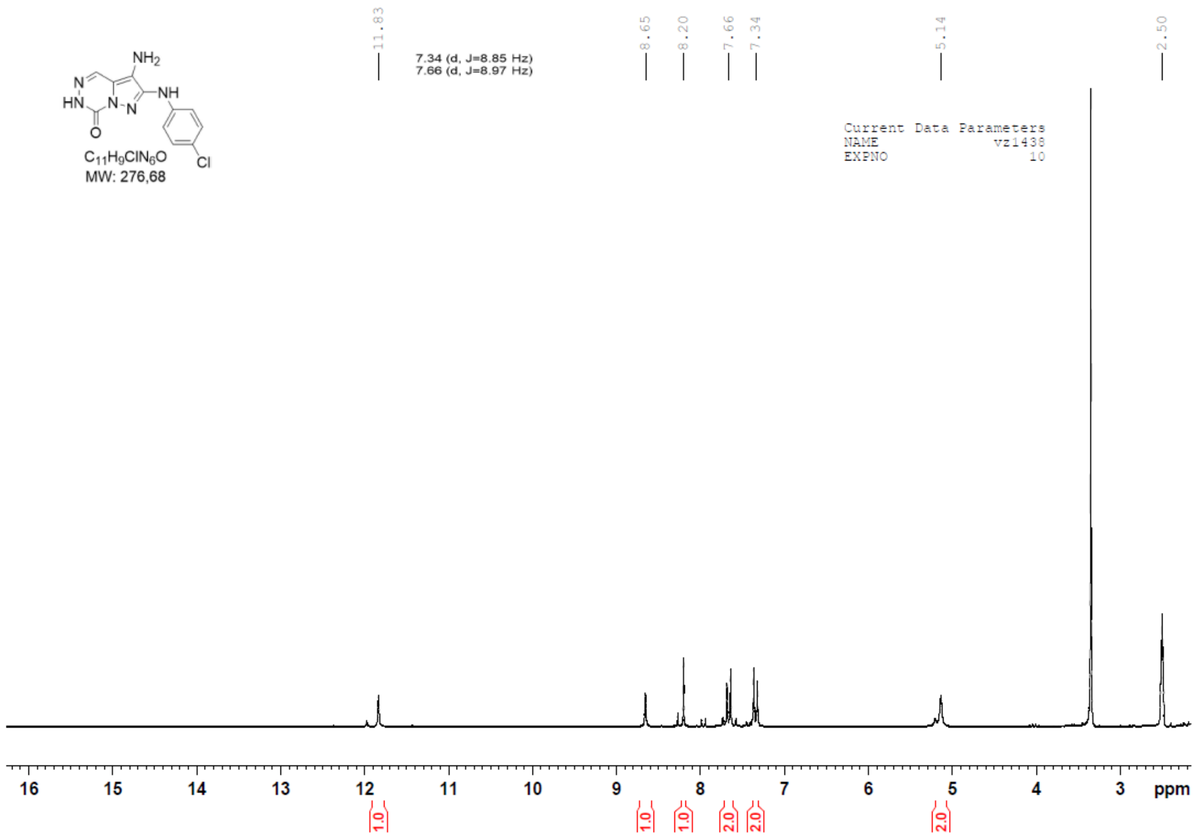

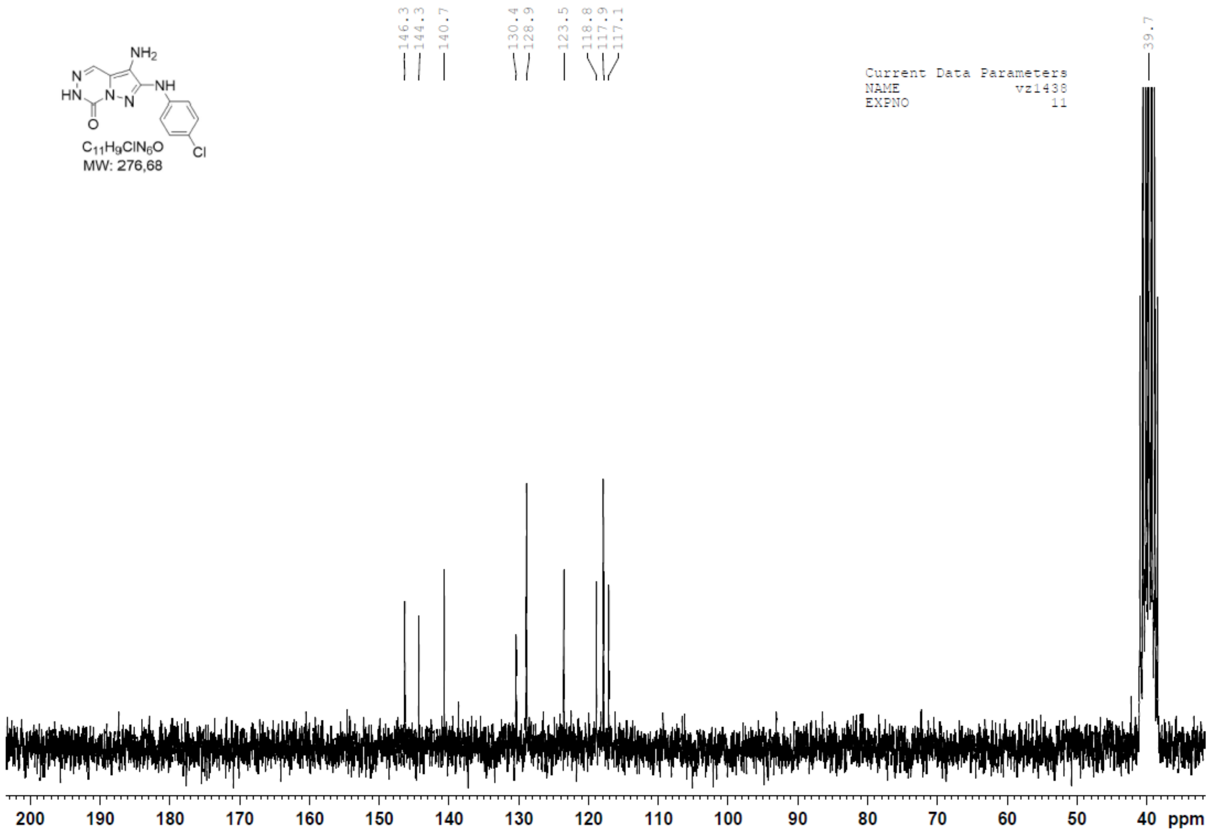

Figure S69-S70. ^{1}H and ^{13}C NMR spectra of compound **5g**

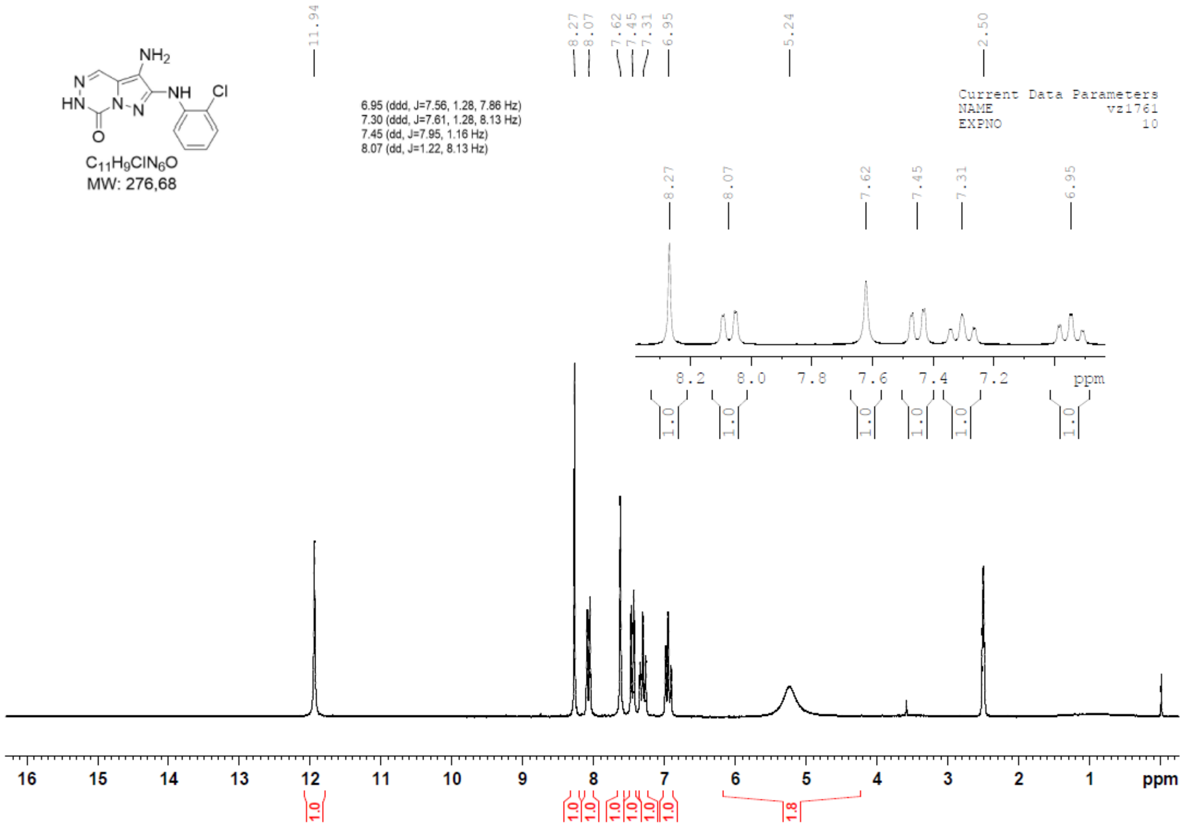

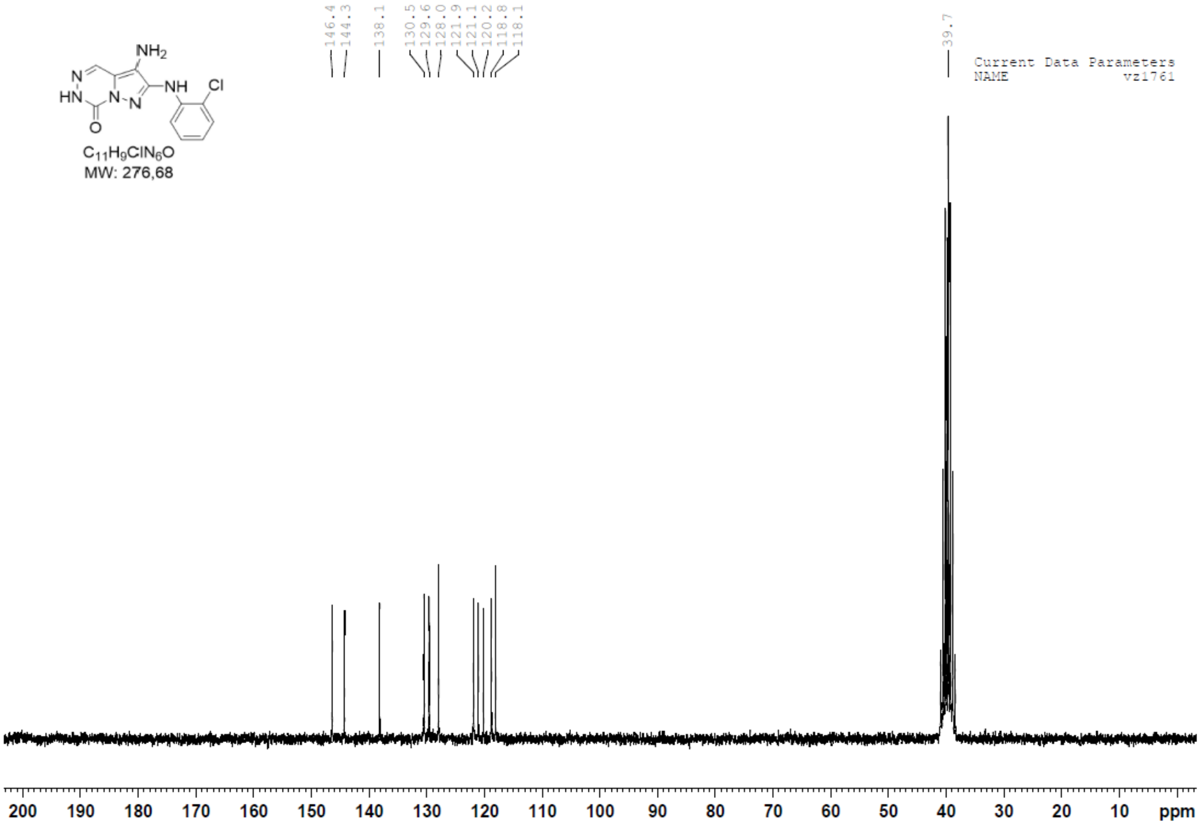

Figure S71-S72. ^{1}H and ^{13}C NMR spectra of compound **5h**

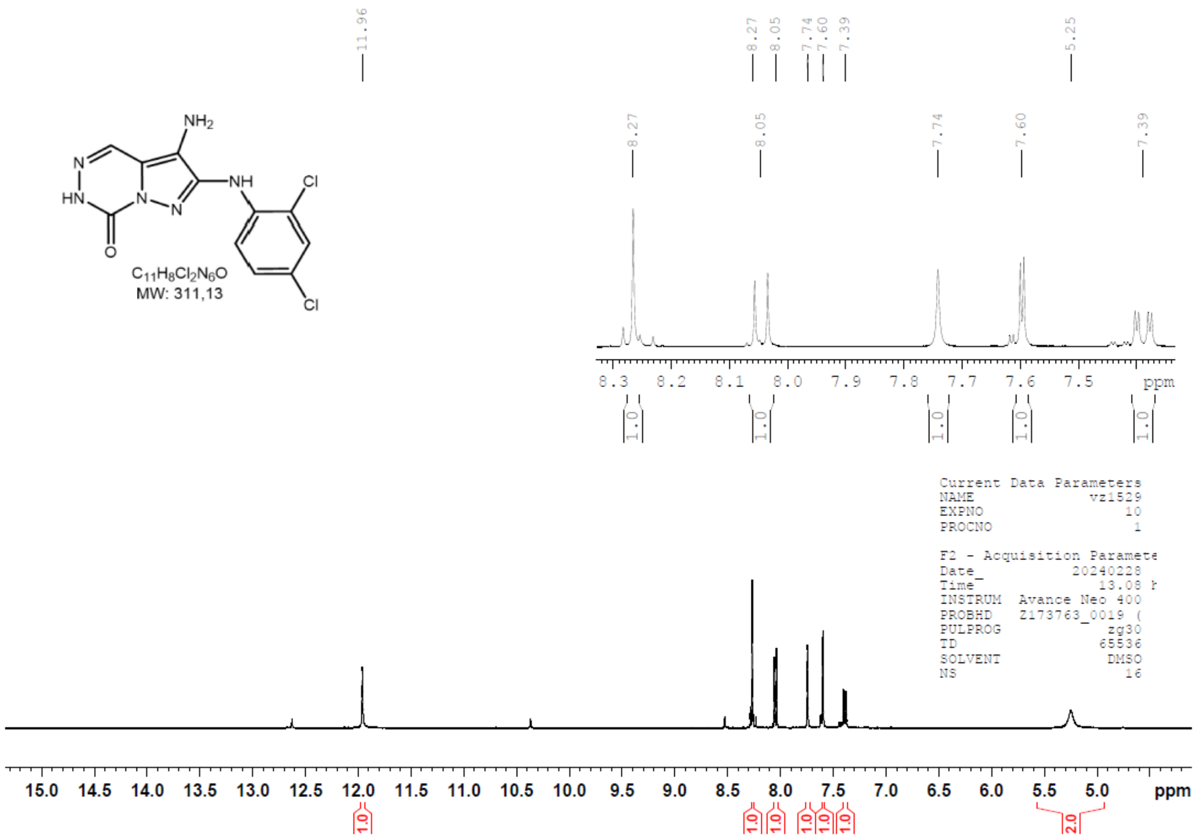

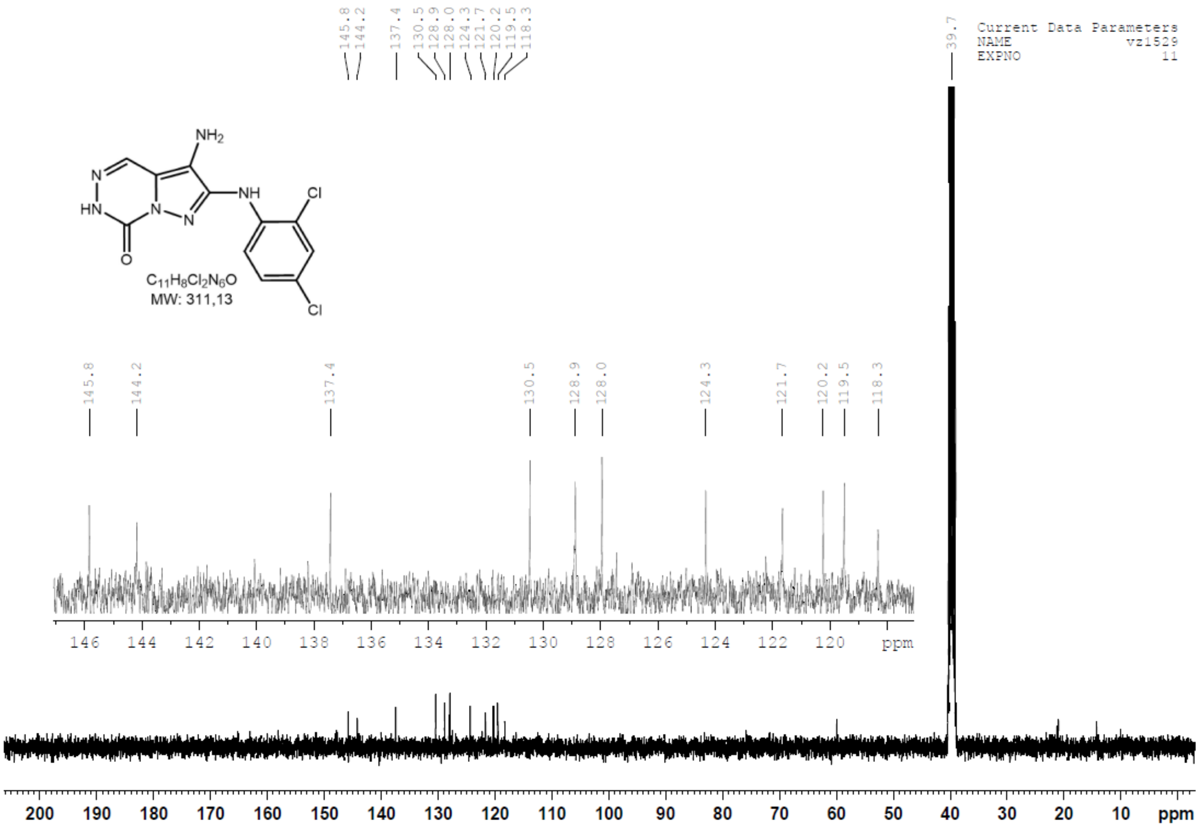

Figure S73-S74. ^{1}H and ^{13}C NMR spectra of compound **5i**

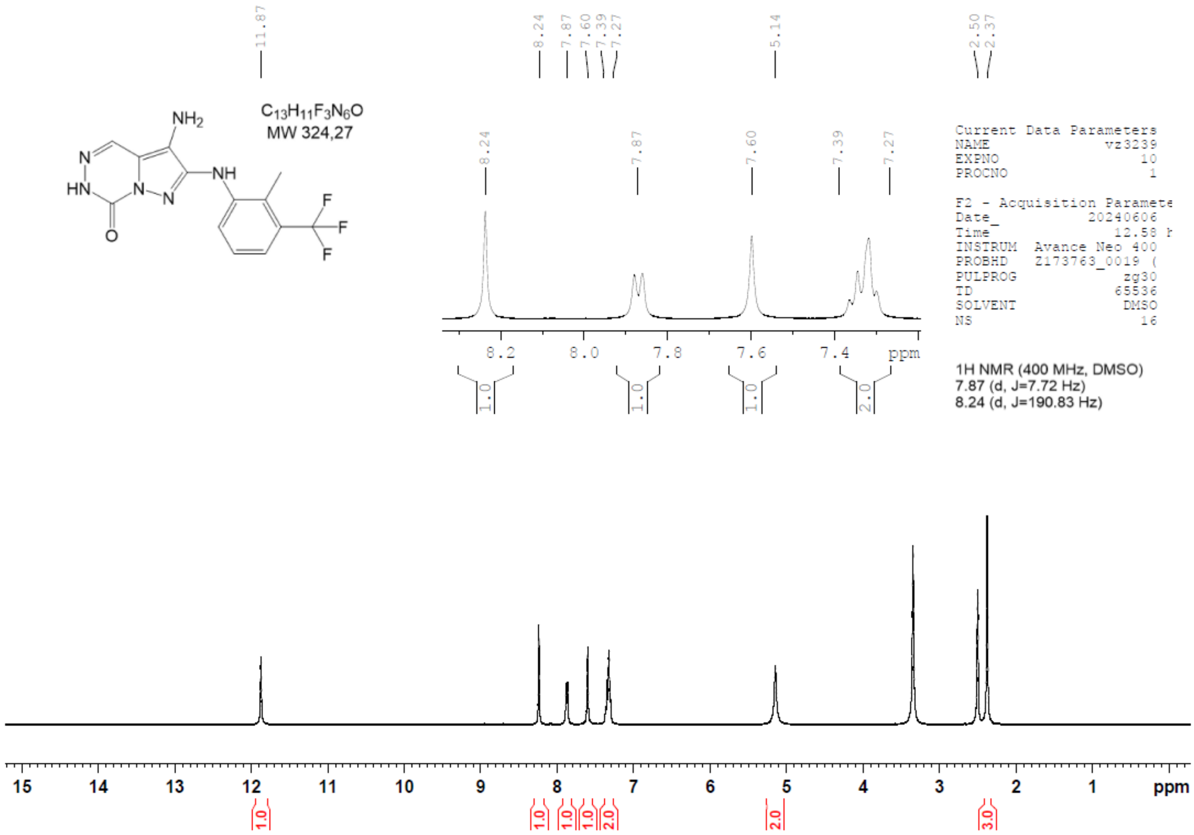

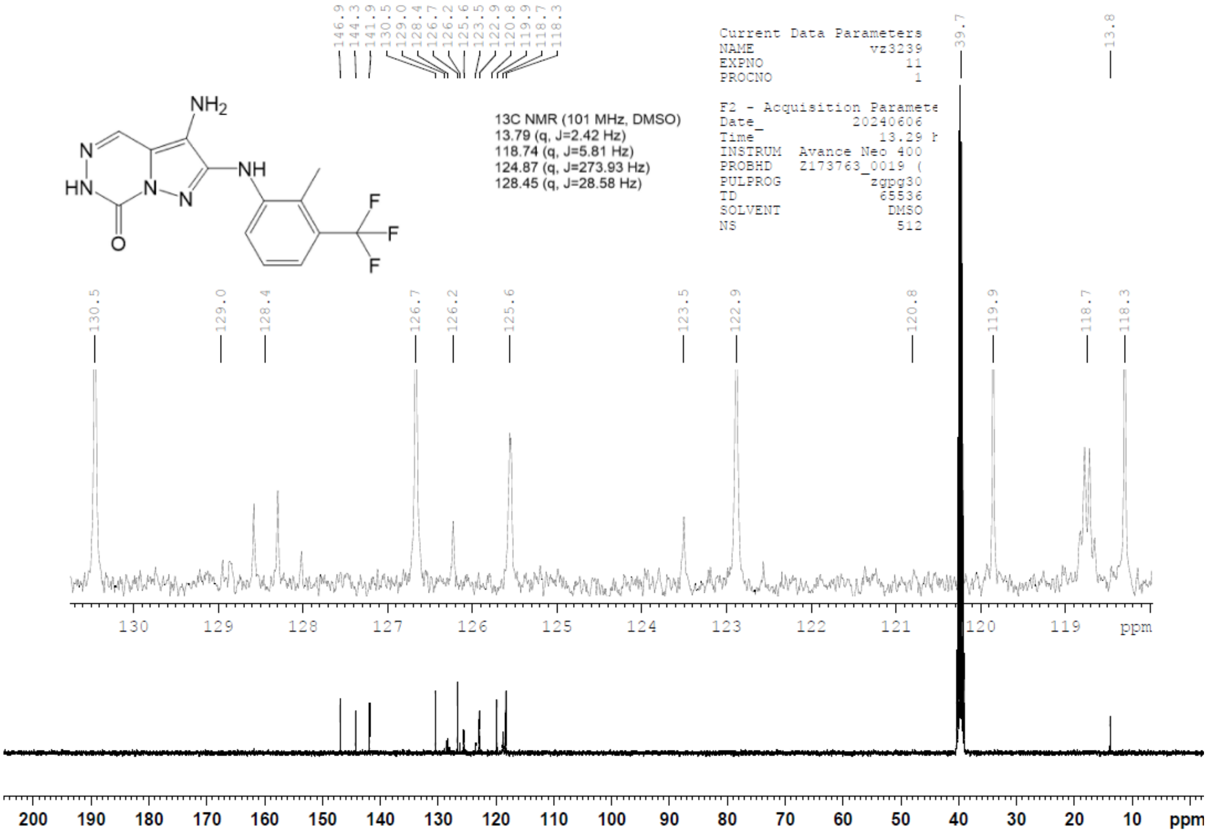

Figure S75-S76. ^{1}H and ^{13}C NMR spectra of compound **5j**

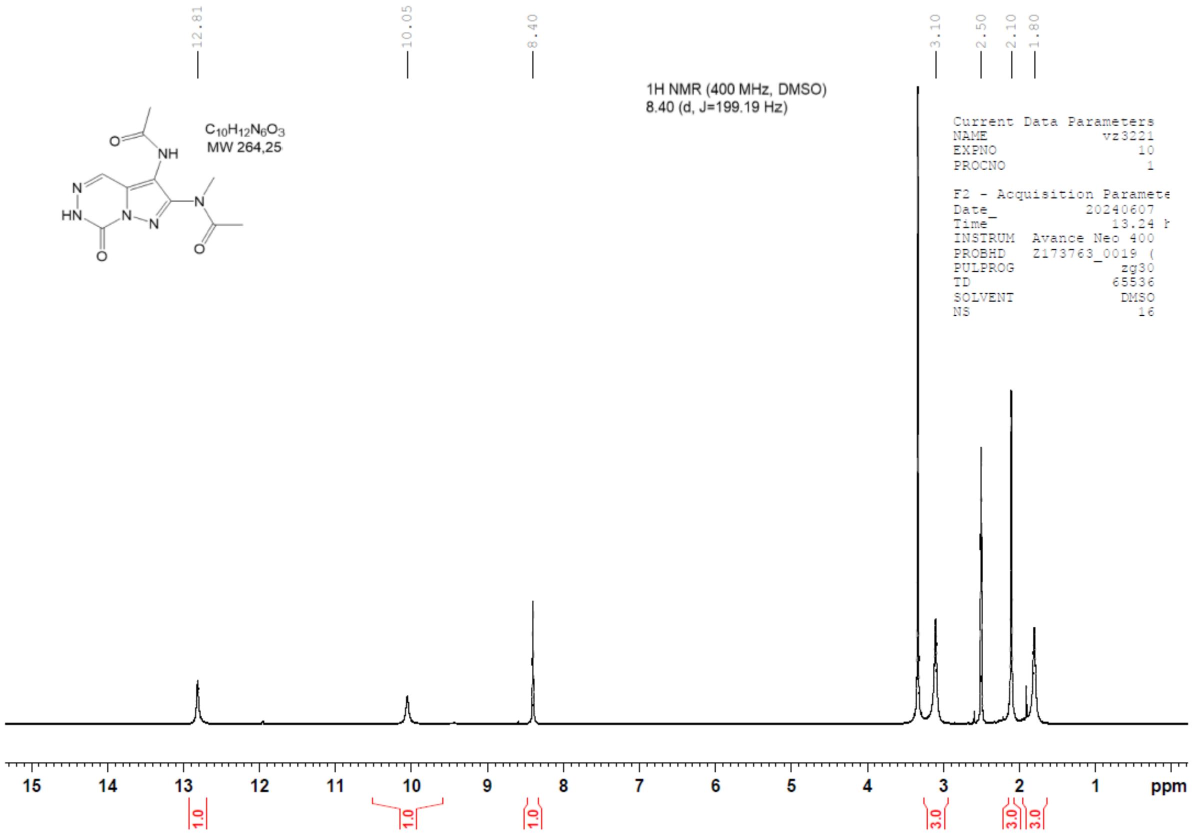

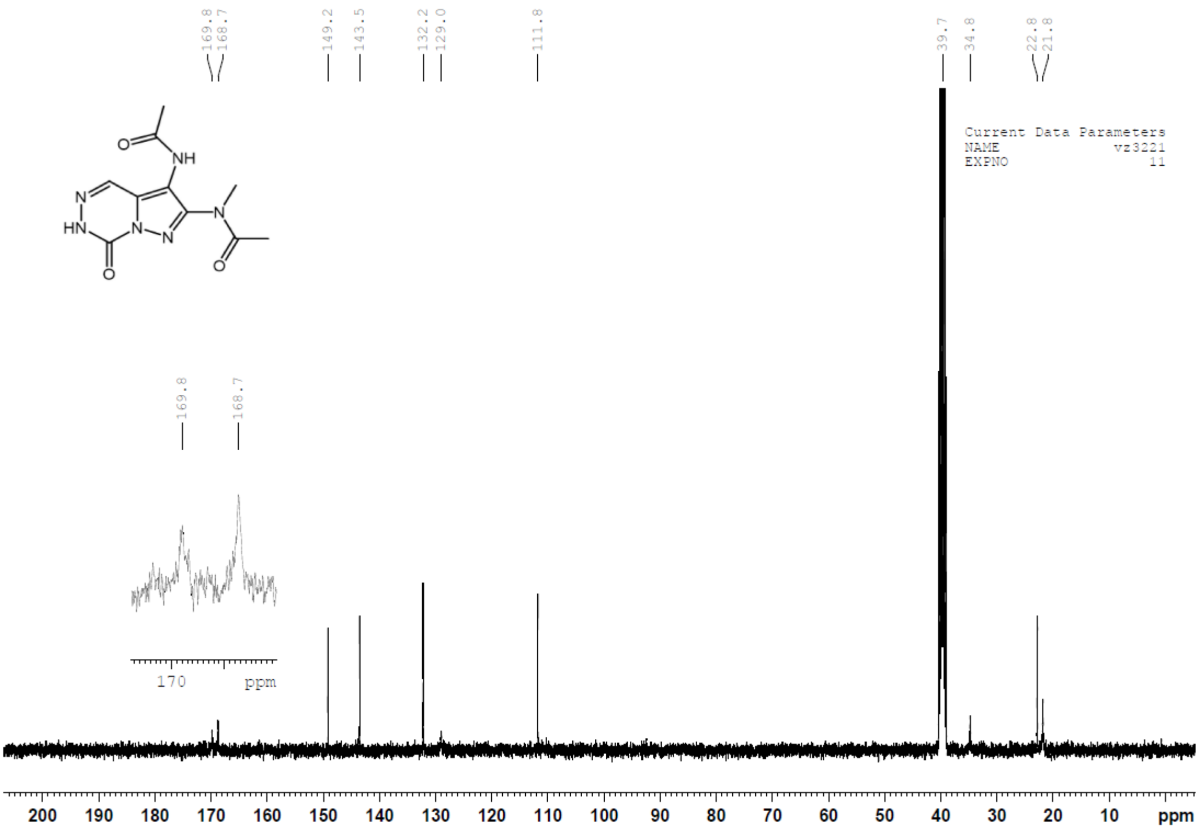

Figure S77-S78. ^{1}H and ^{13}C NMR spectra of compound **6a**

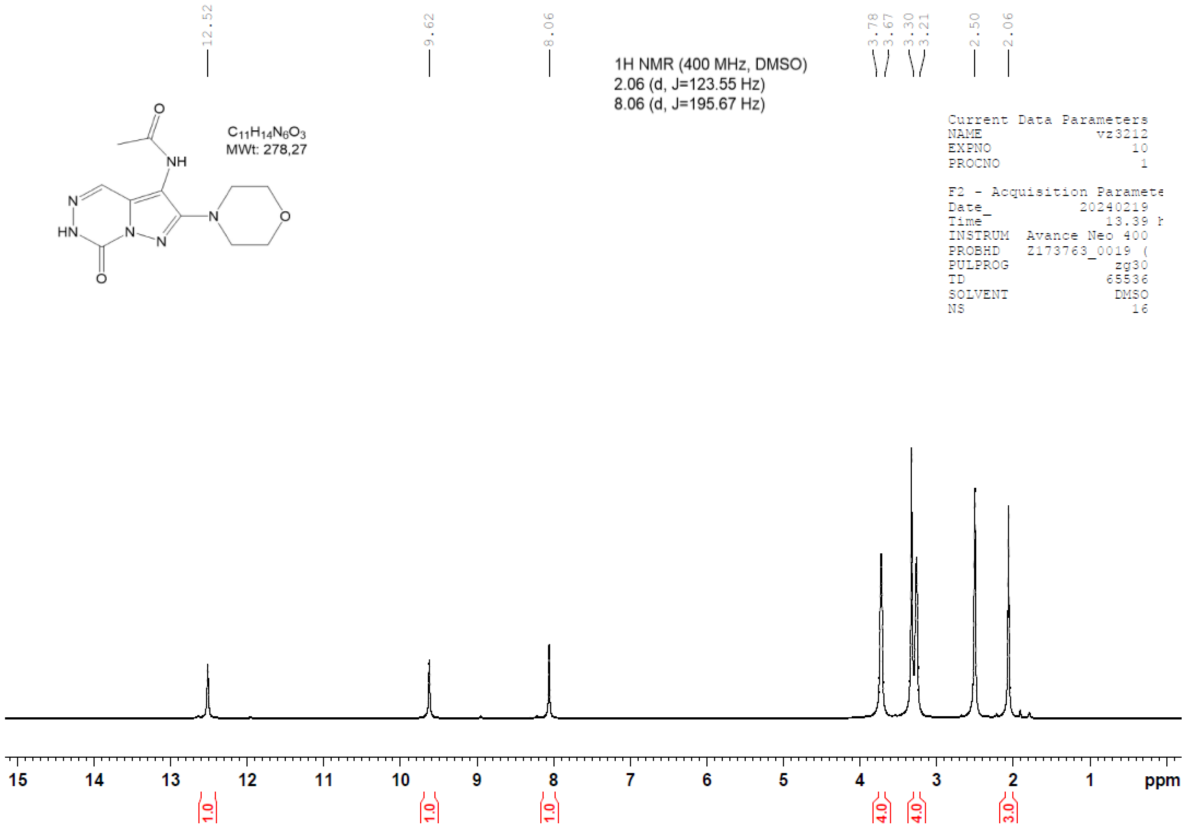

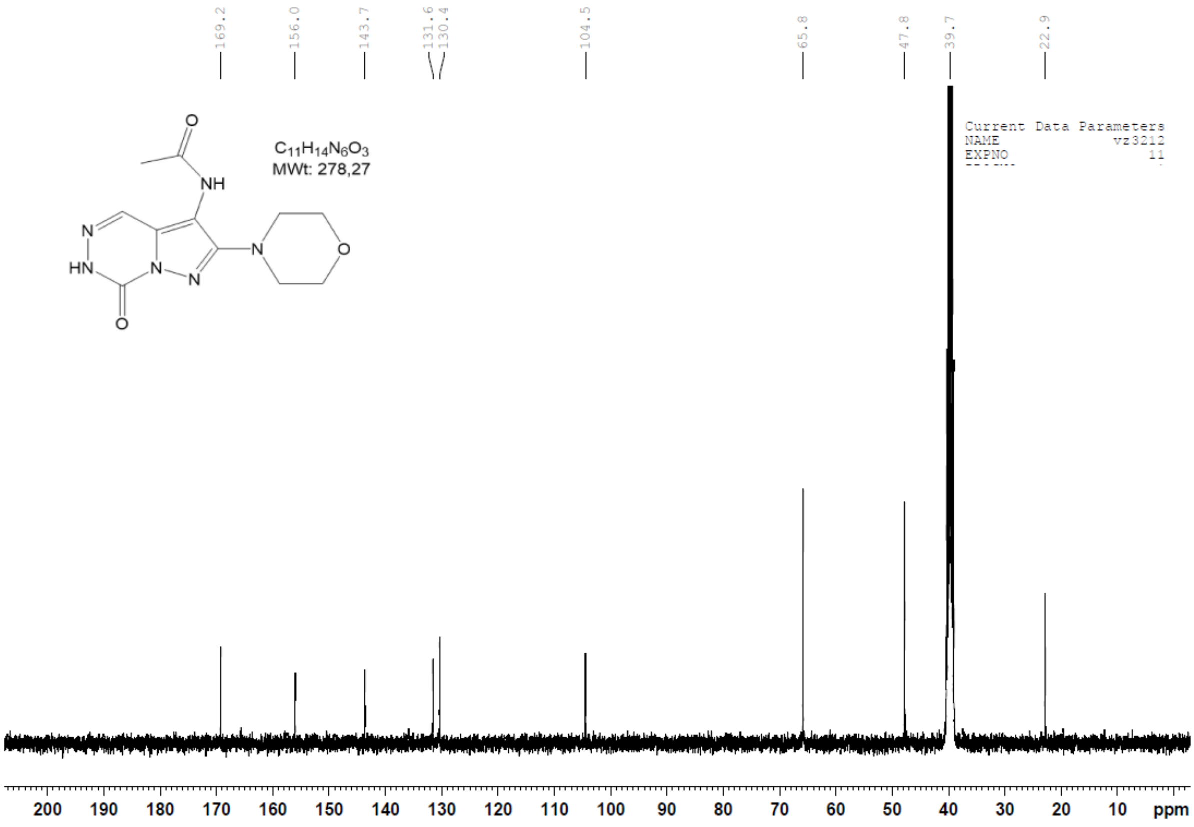

Figure S79-S80. ¹H and ¹³C NMR spectra of compound **6b**

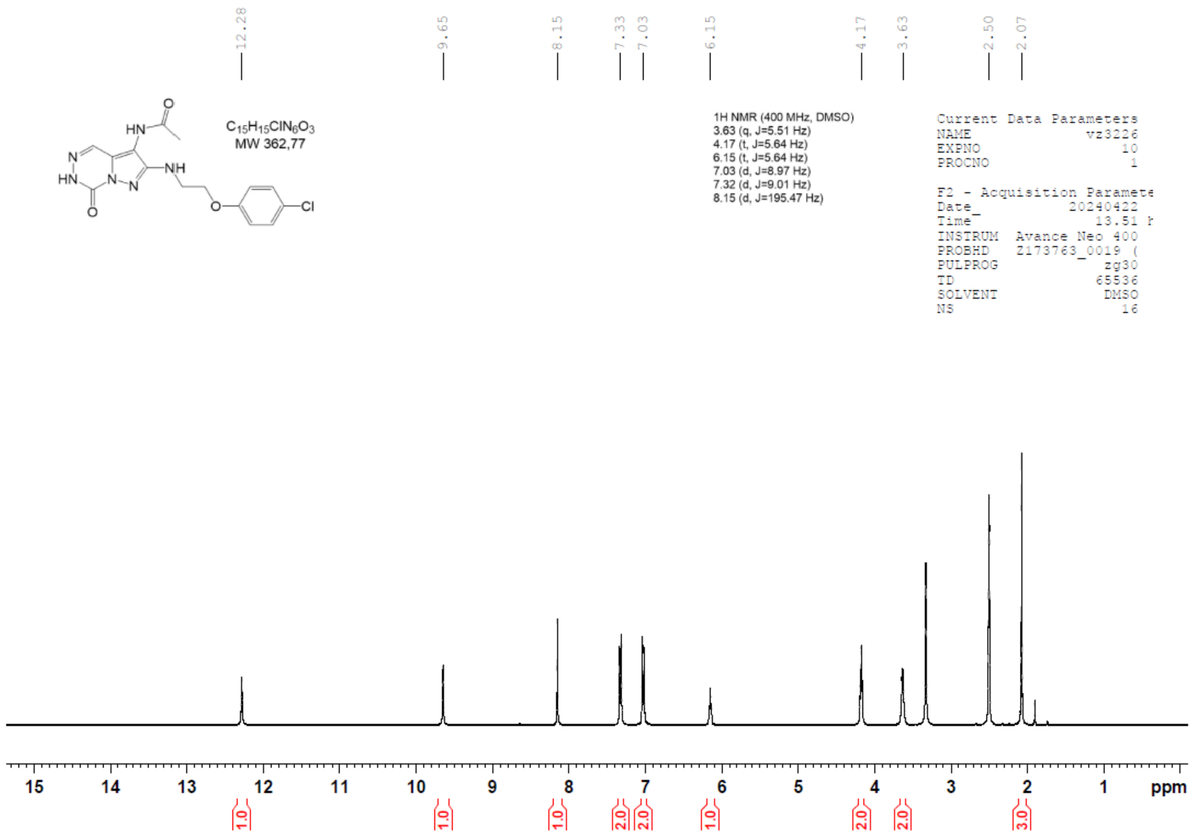

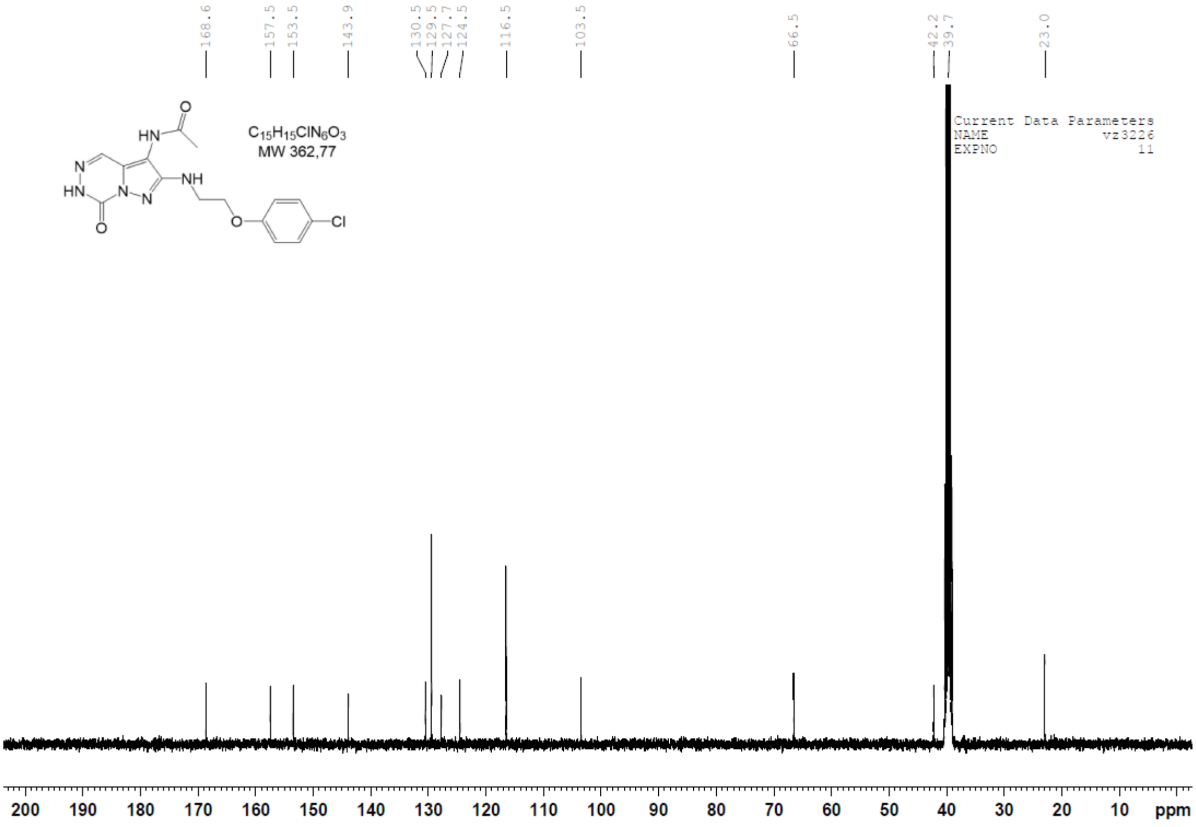

Figure S81-S82. ^{1}H and ^{13}C NMR spectra of compound **6c**

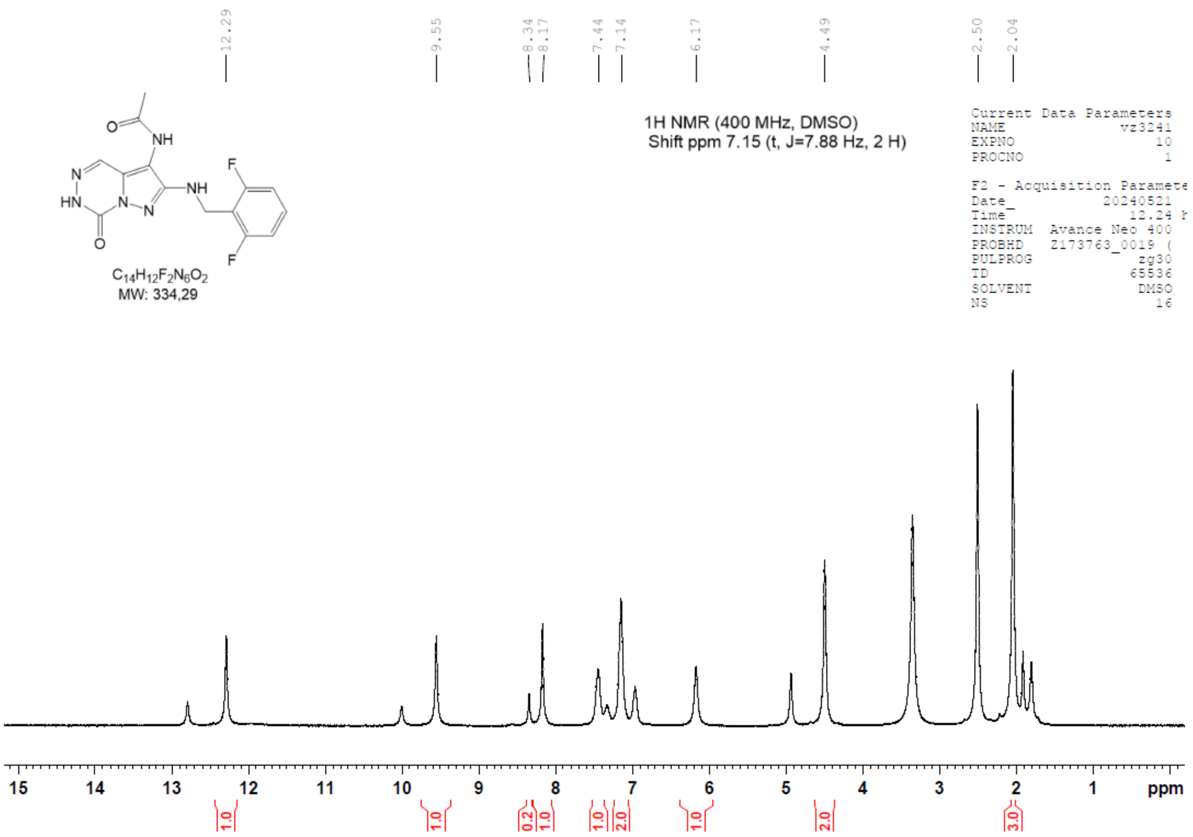

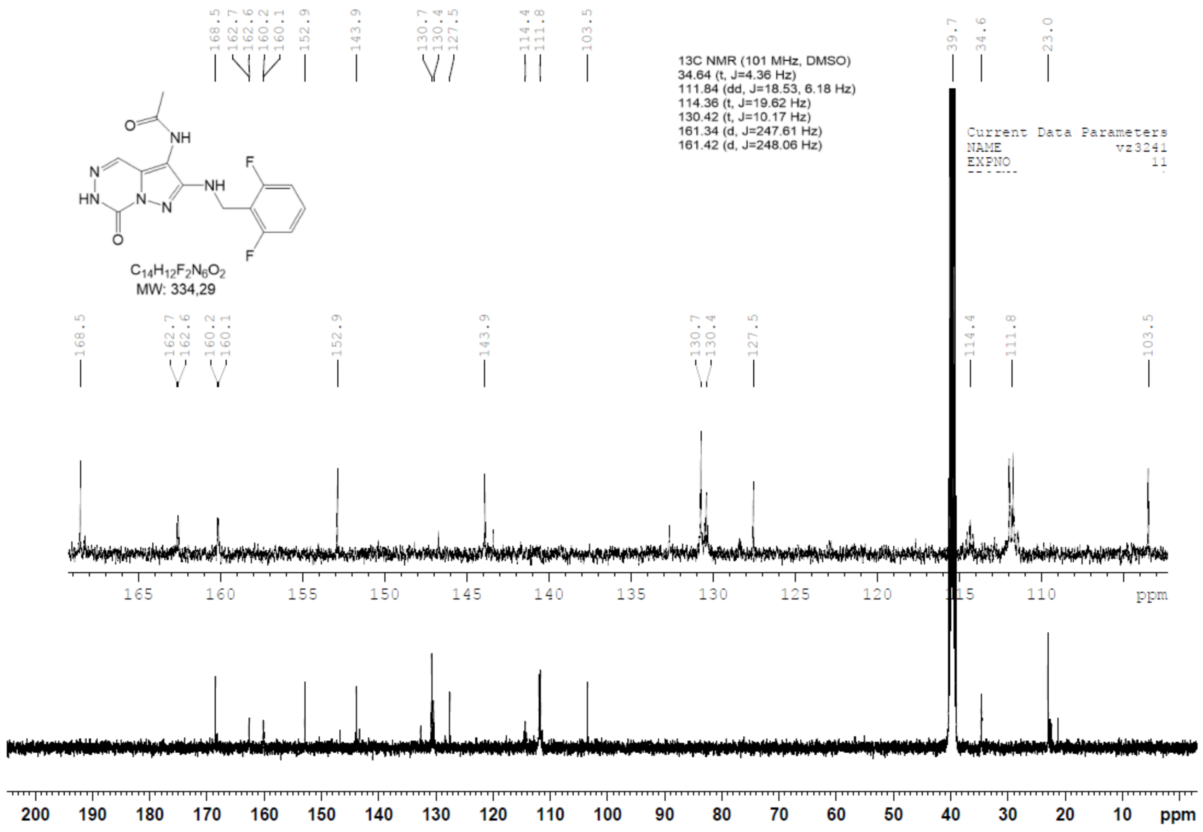

Figure S83-S84. ^{1}H and ^{13}C NMR spectra of compound **6d**

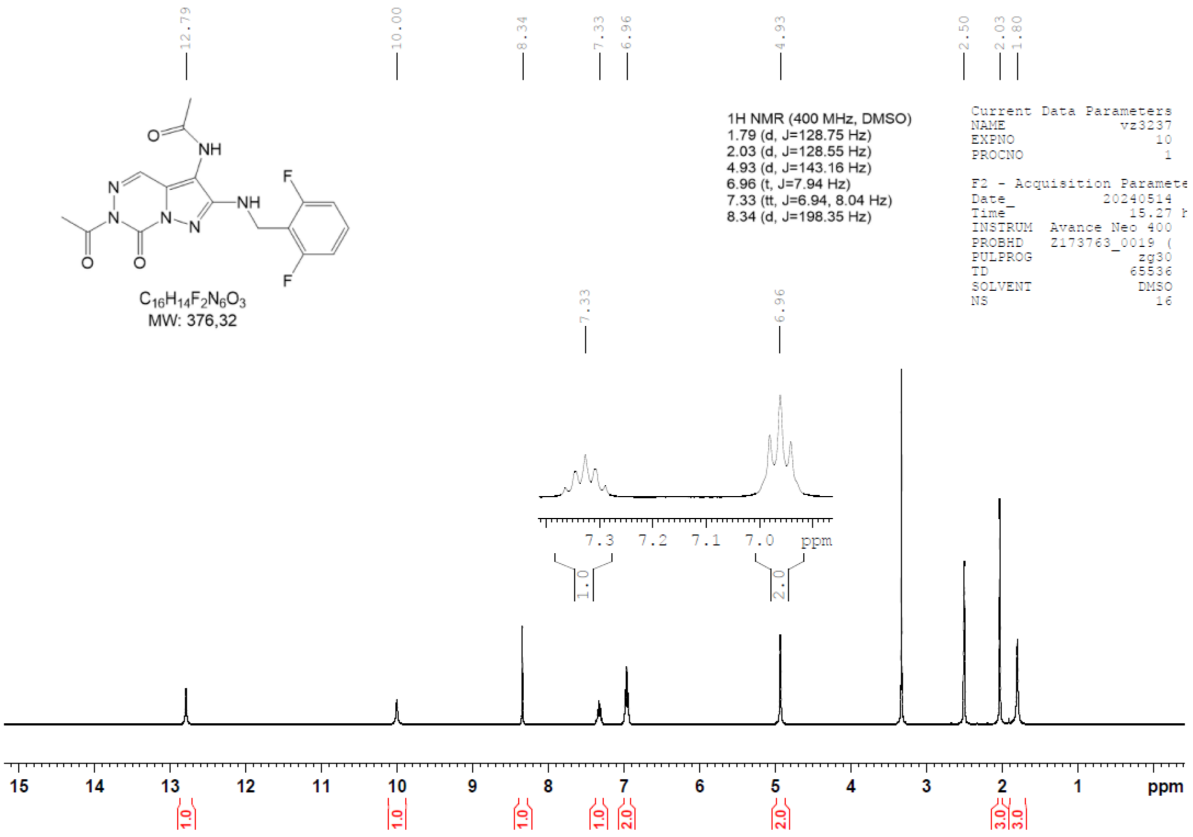

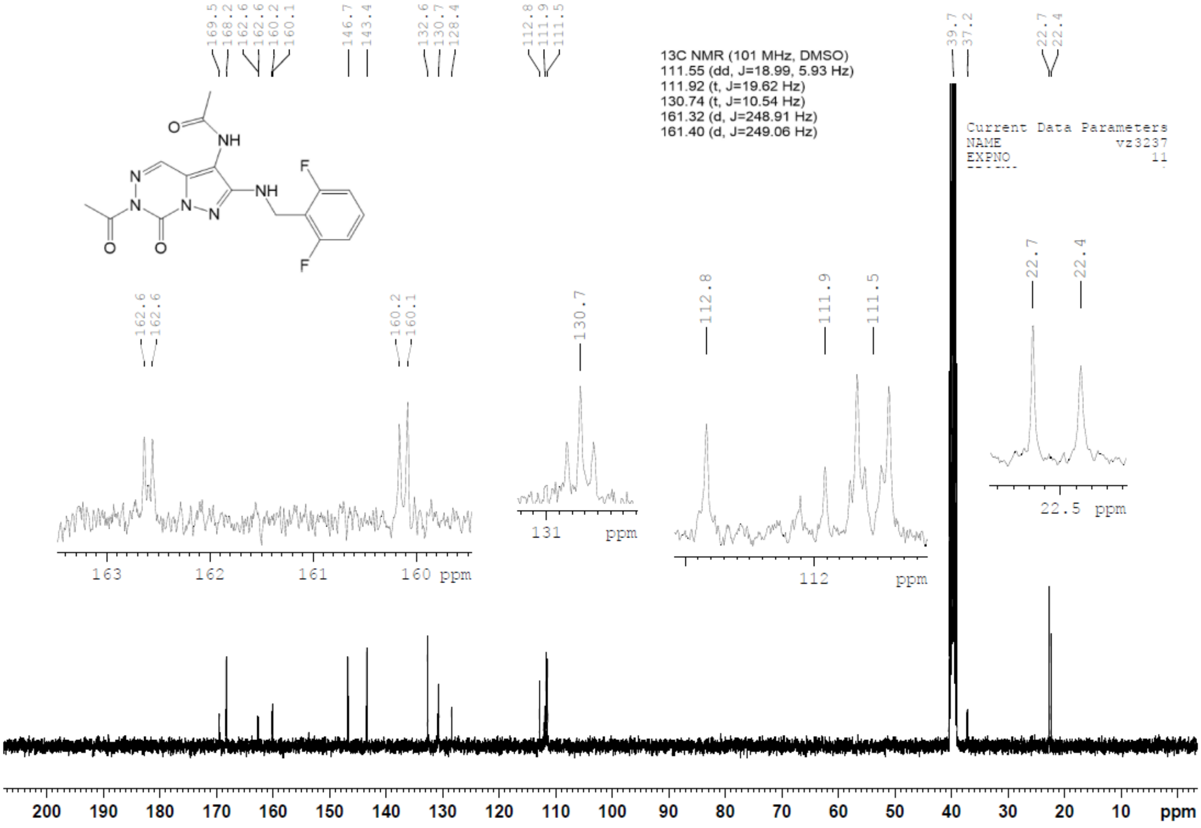

Figure S85-S86. ^{1}H and ^{13}C NMR spectra of compound **6dd**

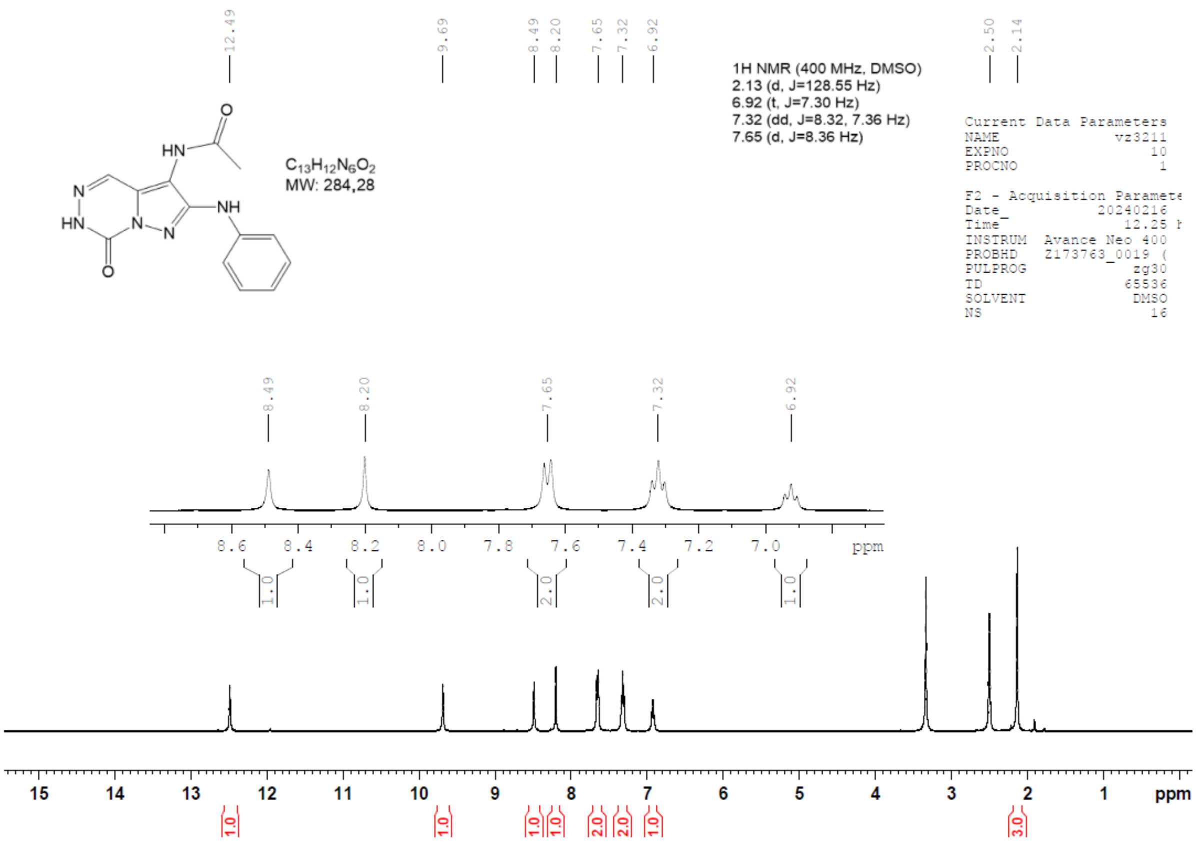

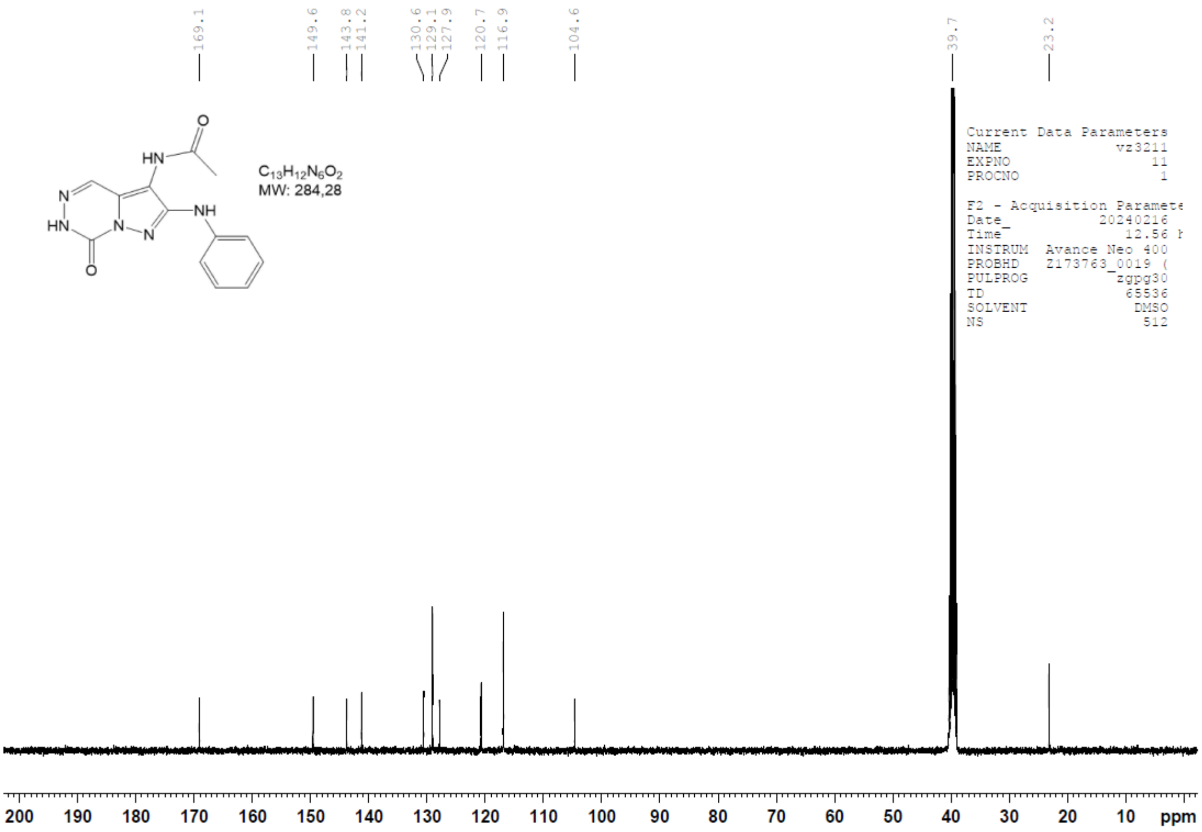

Figure S87-S88. ^{1}H and ^{13}C NMR spectra of compound **6e**

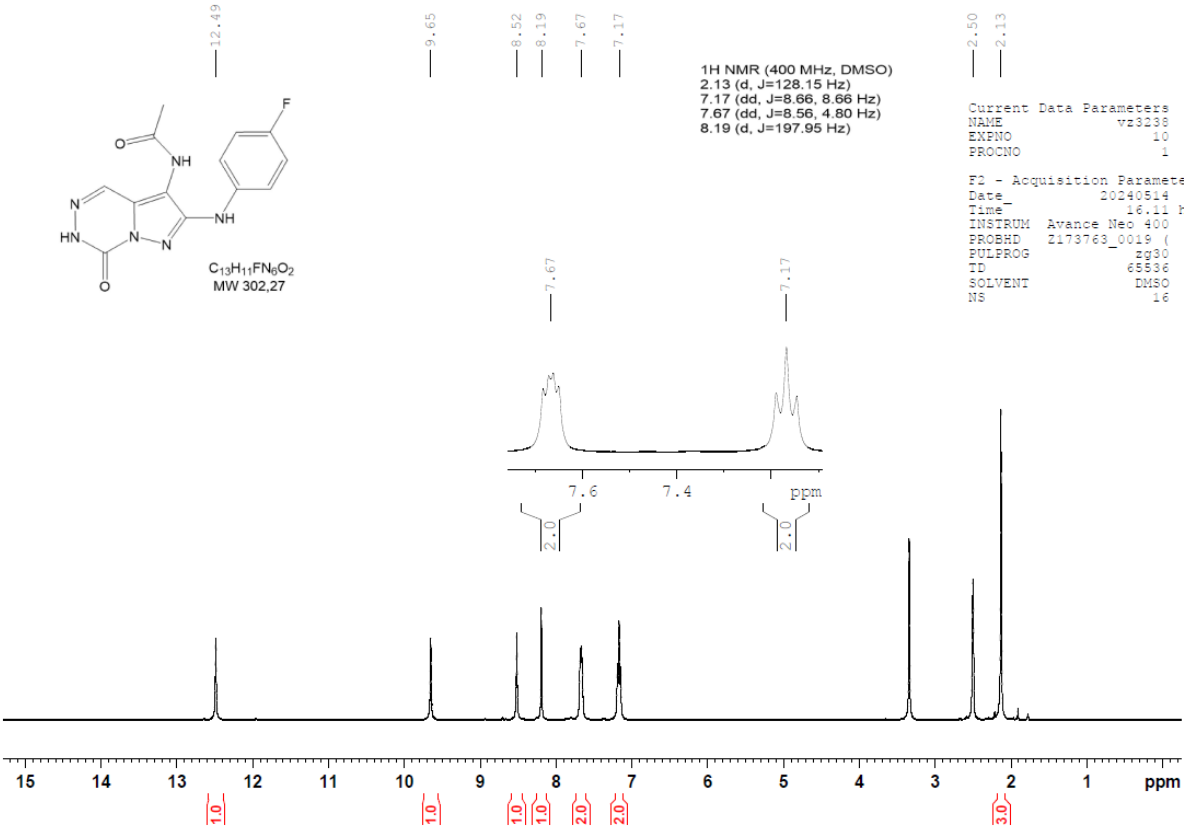

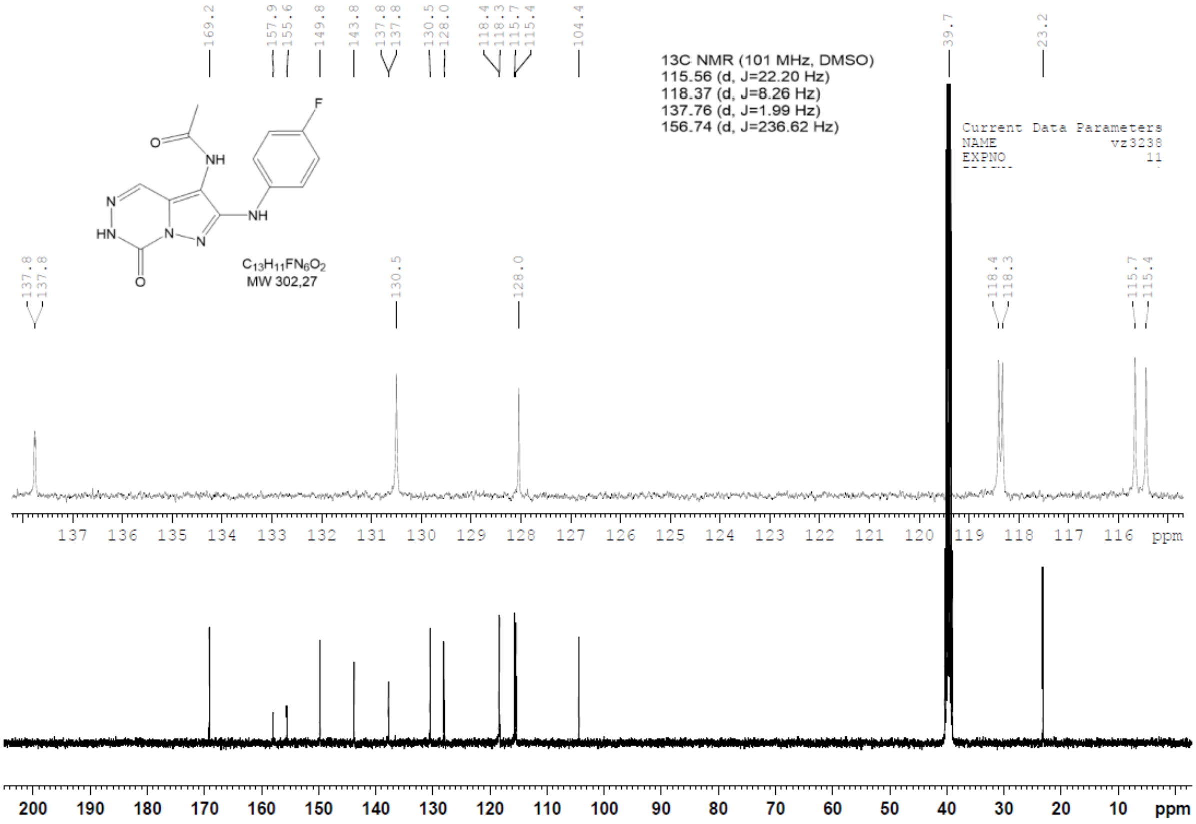

Figure S89-S90. ^{1}H and ^{13}C NMR spectra of compound **6f**

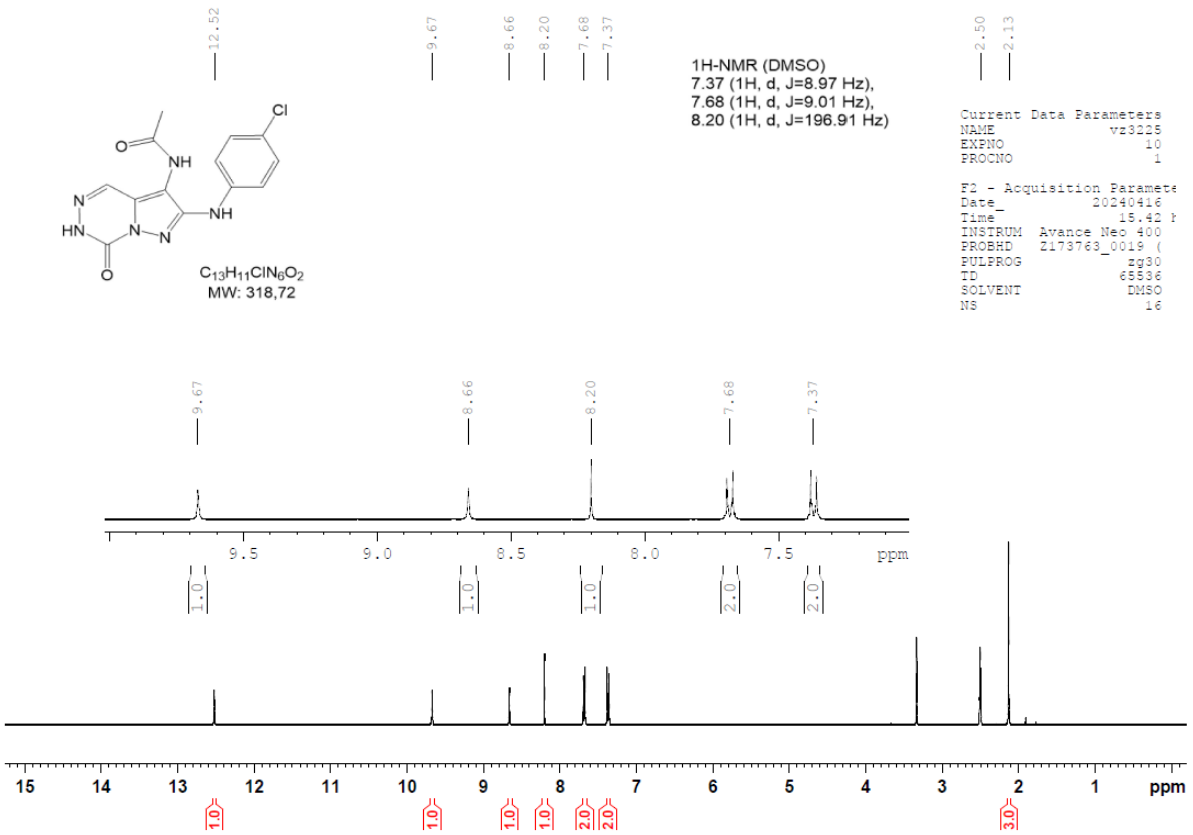

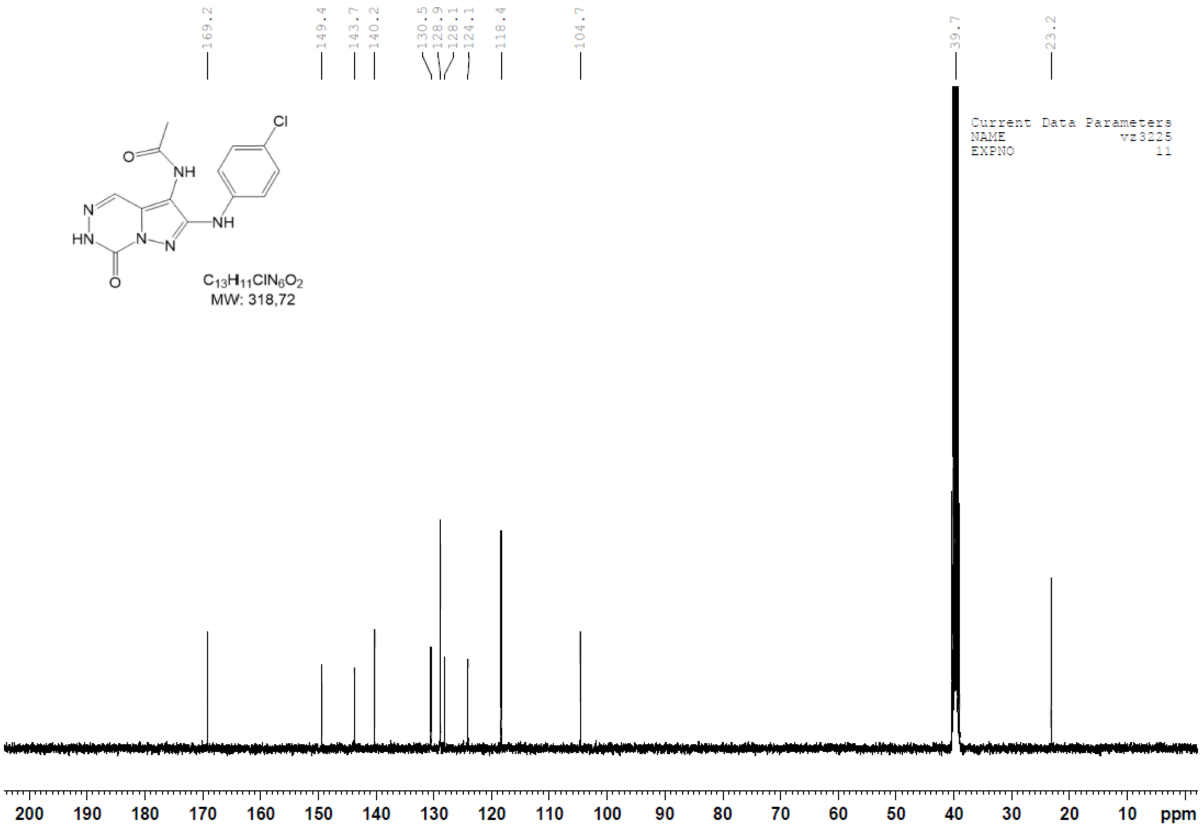

Figure S91-S92. ^{1}H and ^{13}C NMR spectra of compound **6g**

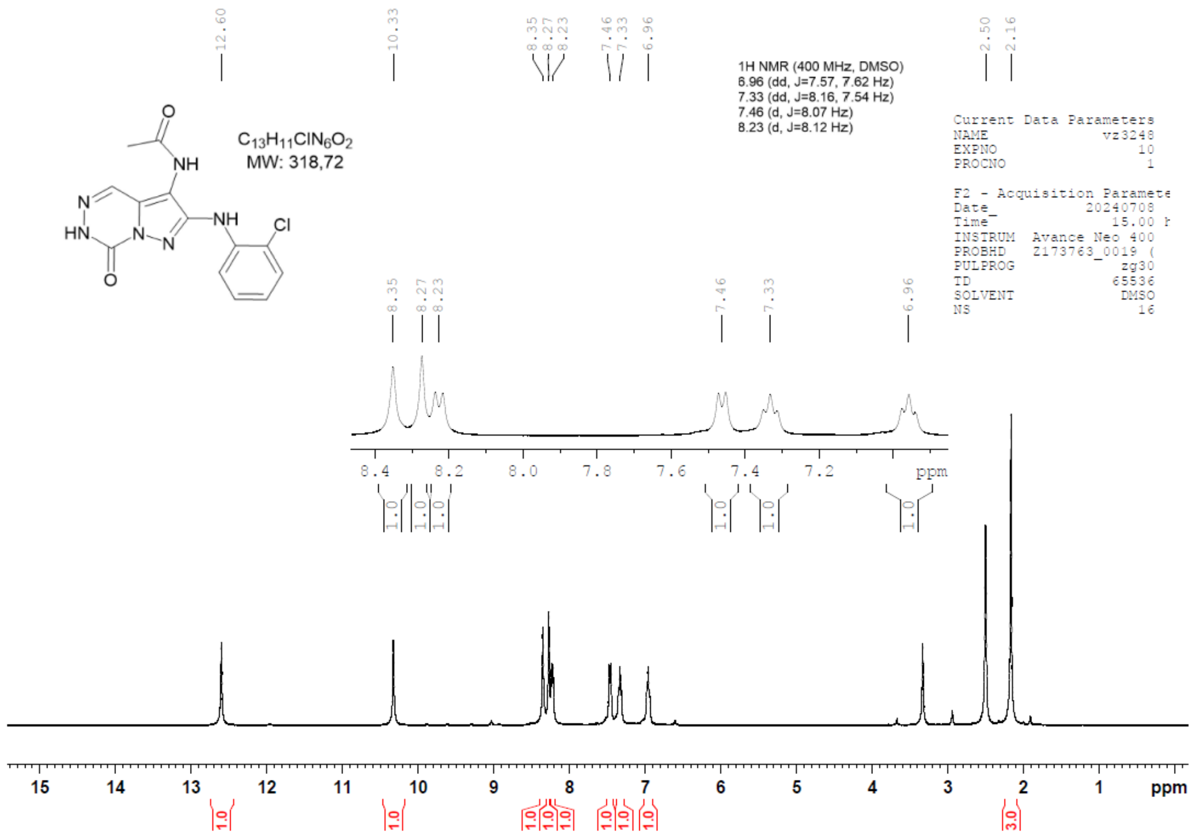

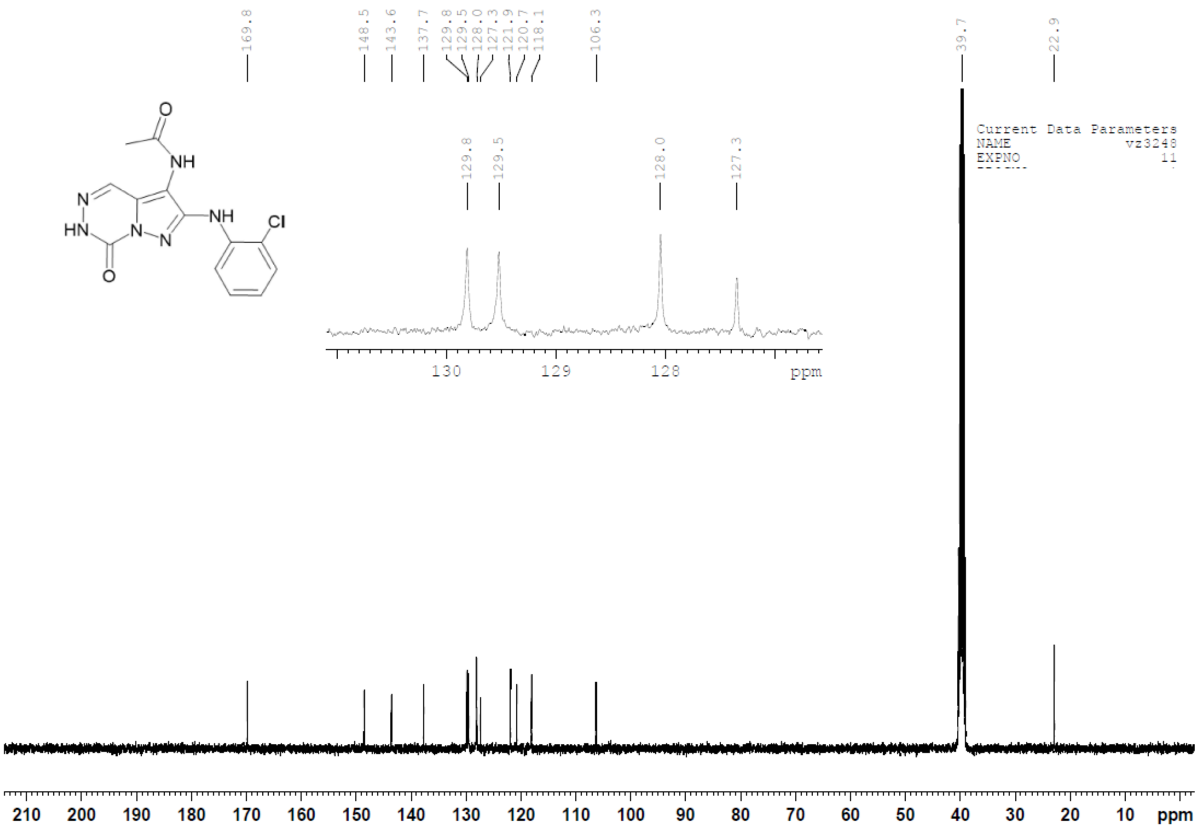

Figure S93-S94. ^{1}H and ^{13}C NMR spectra of compound **6h**

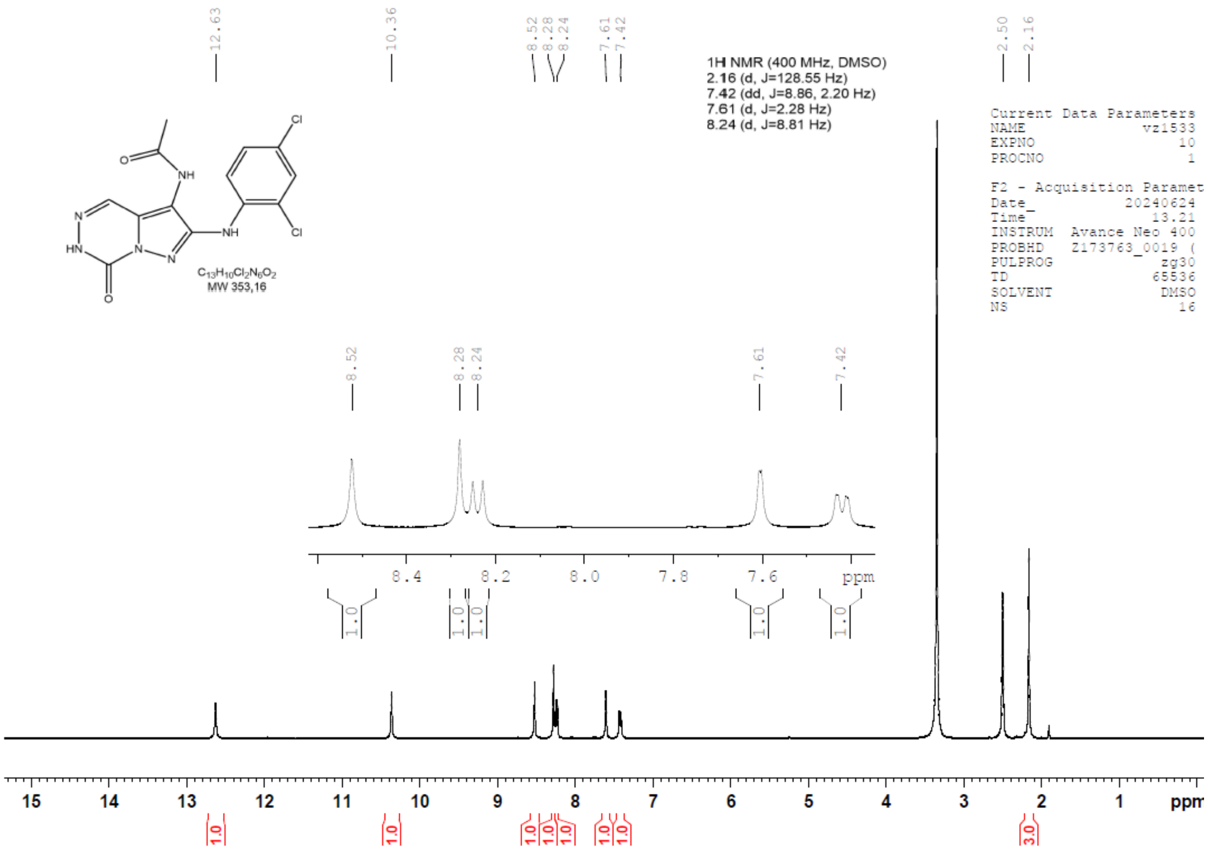

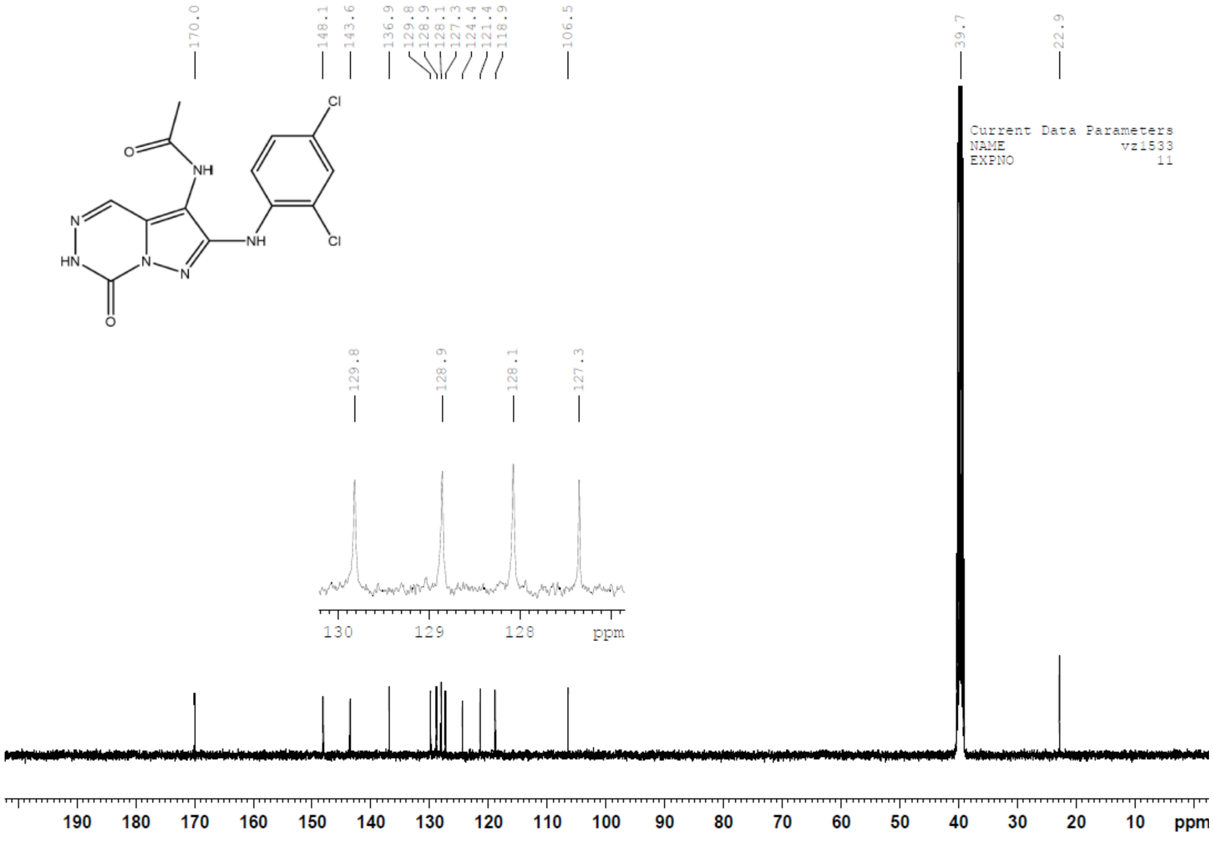

Figure S95-S96. ^{1}H and ^{13}C NMR spectra of compound **6i**

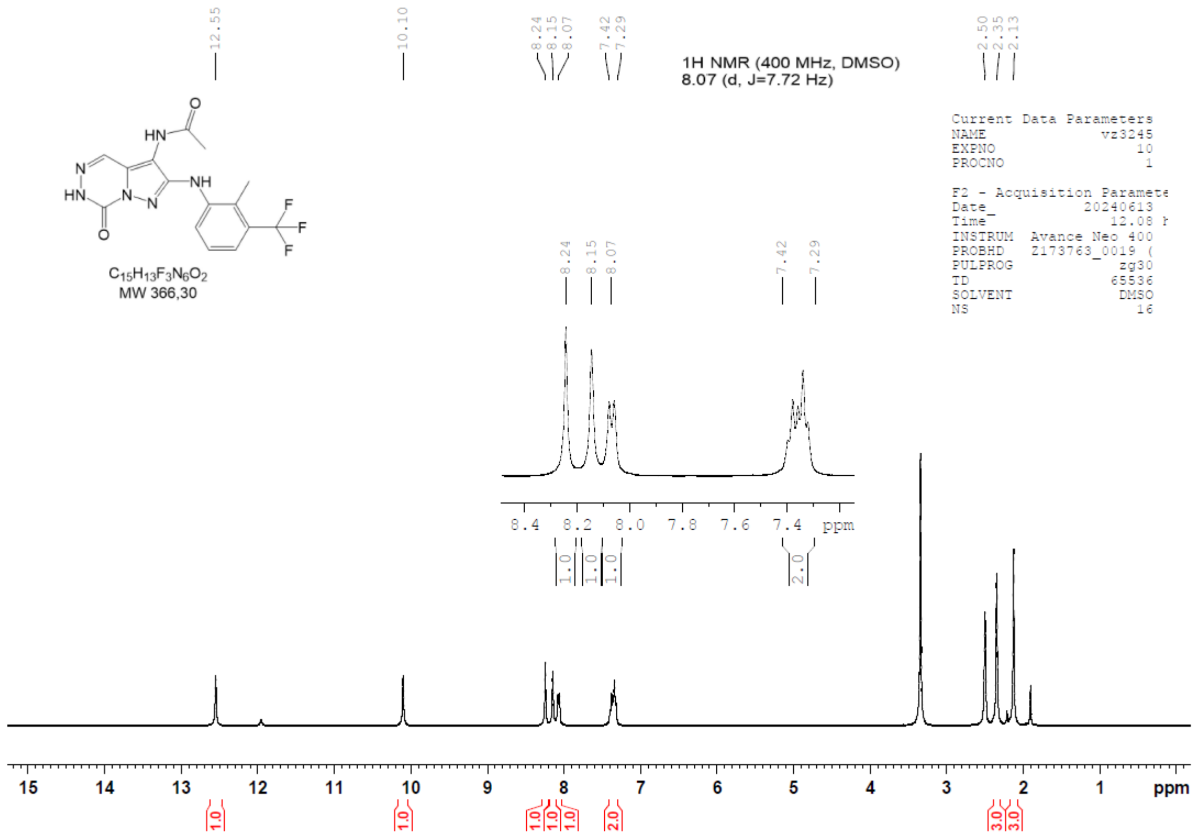

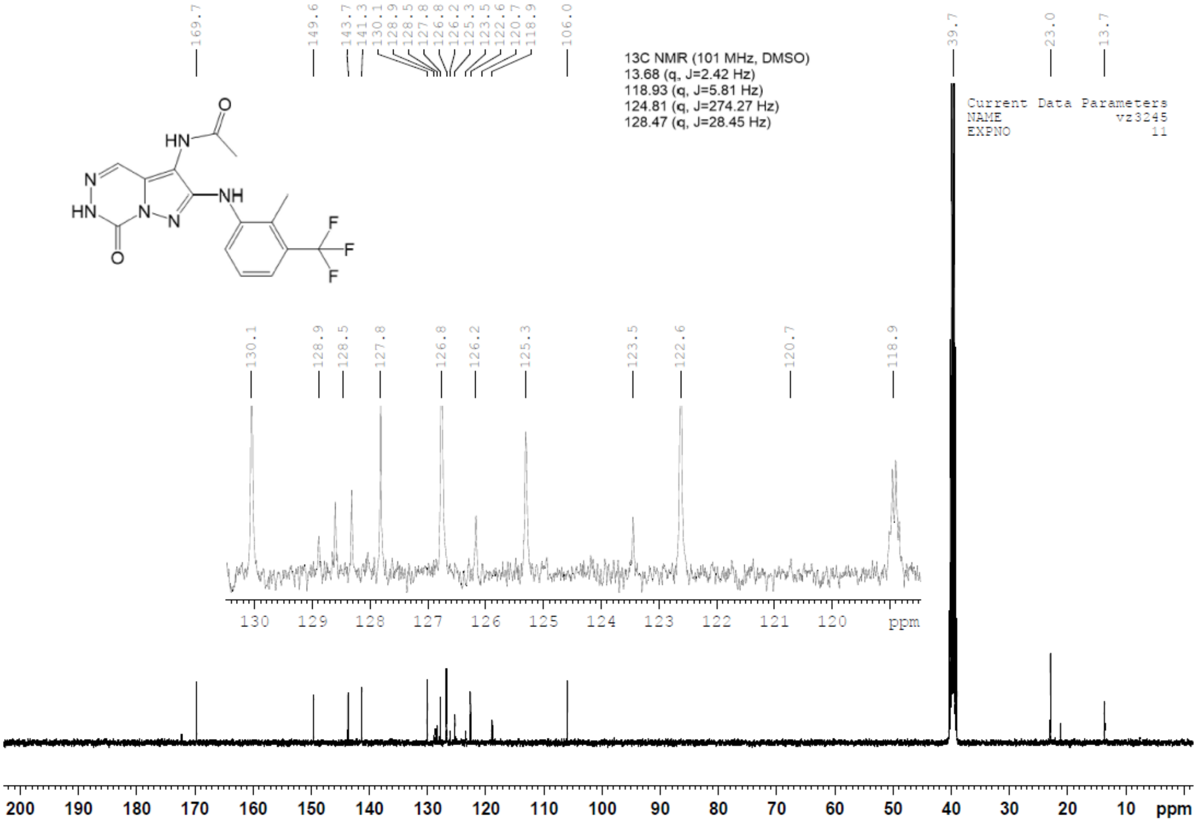

Figure S97-S98. ^{1}H and ^{13}C NMR spectra of compound **6j**

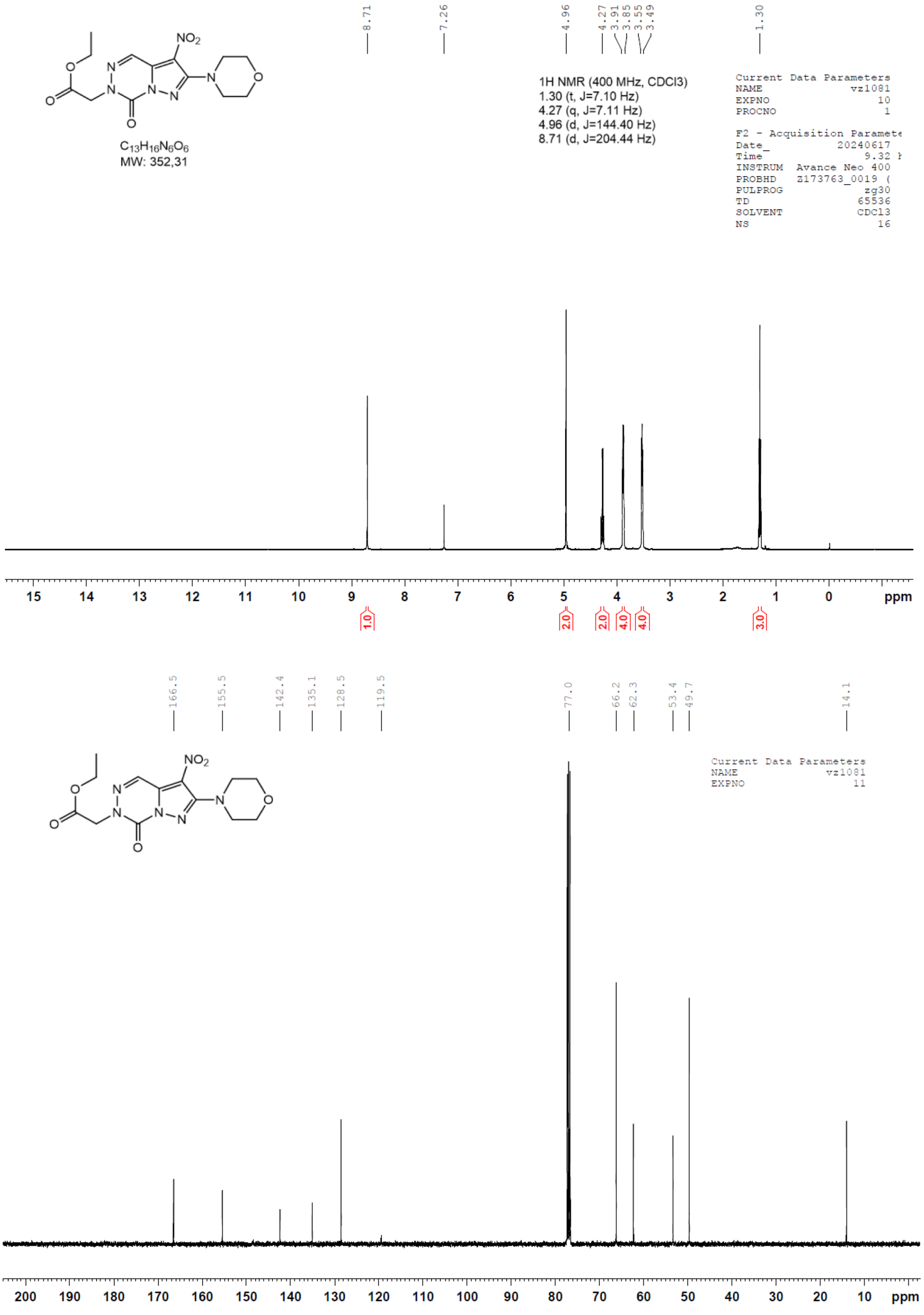

Figure S99-S100. ^{1}H and ^{13}C NMR spectra of compound **7b**

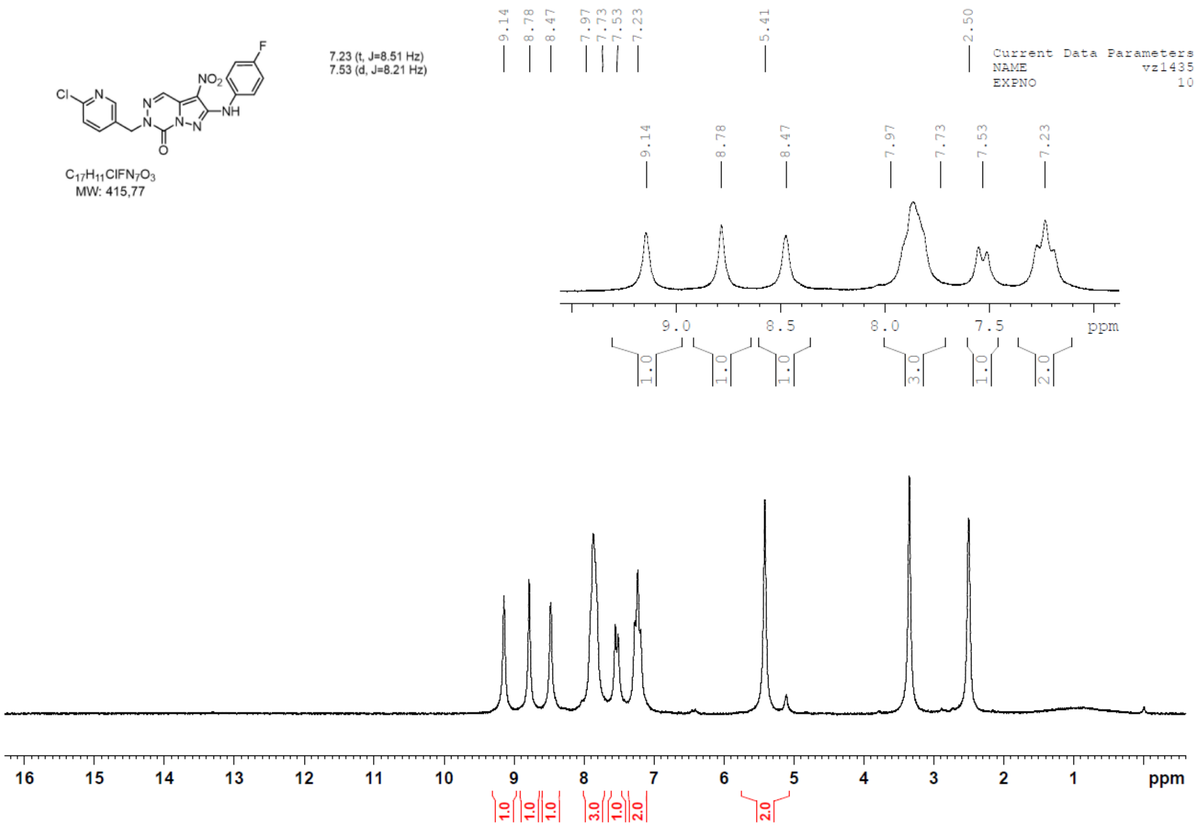

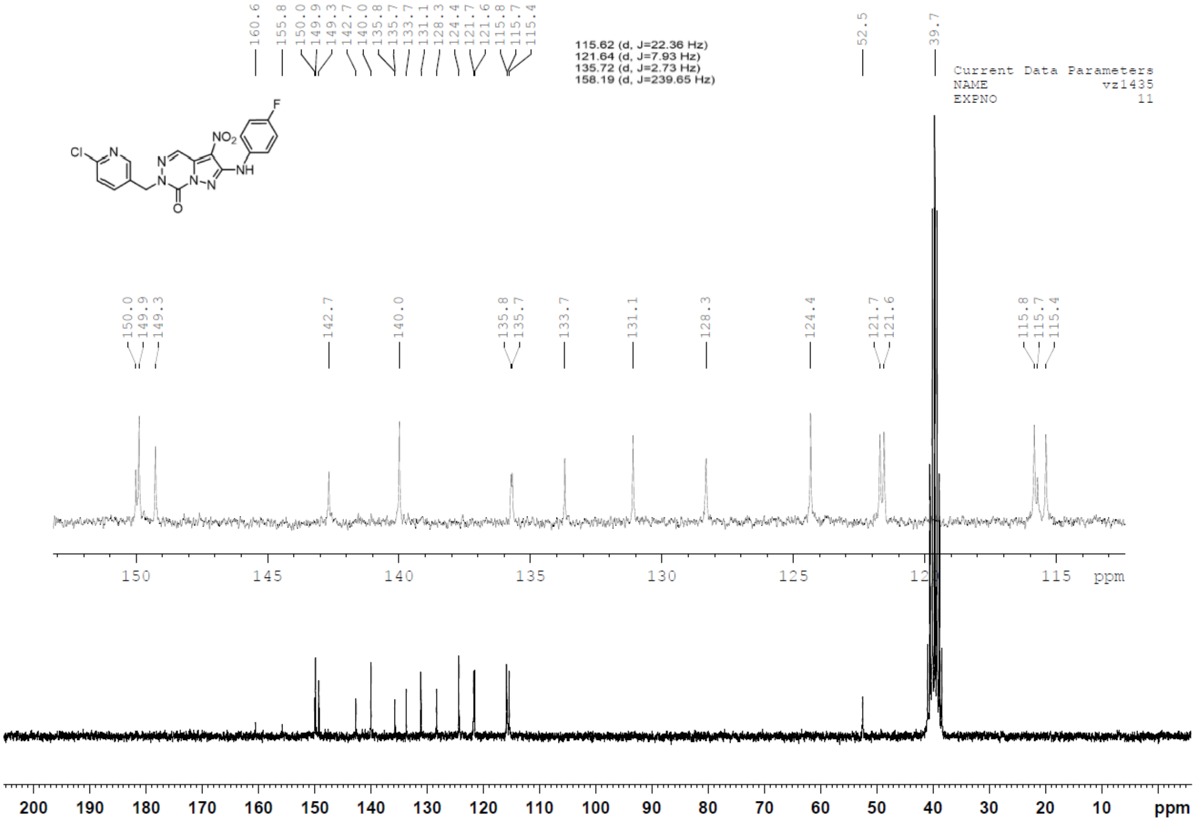

Figure S101-S102. ^{1}H and ^{13}C NMR spectra of compound **7f**

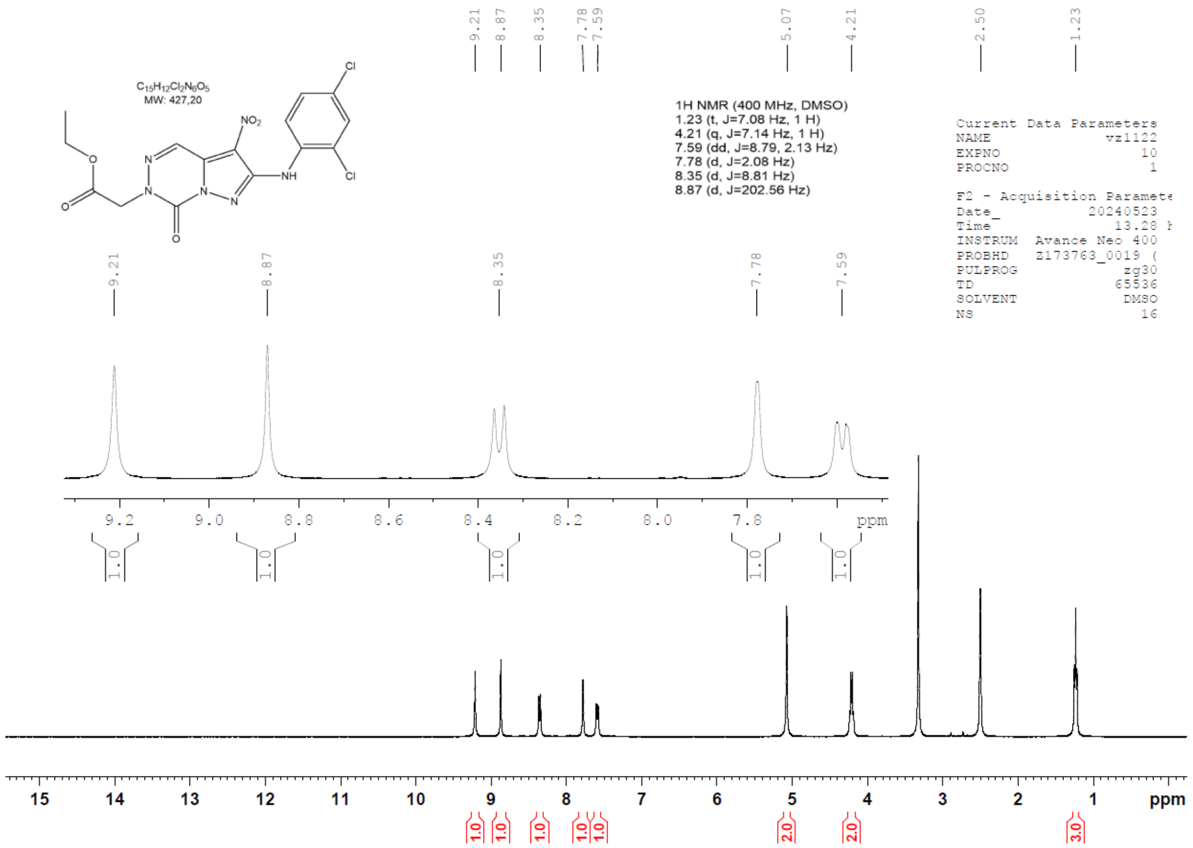

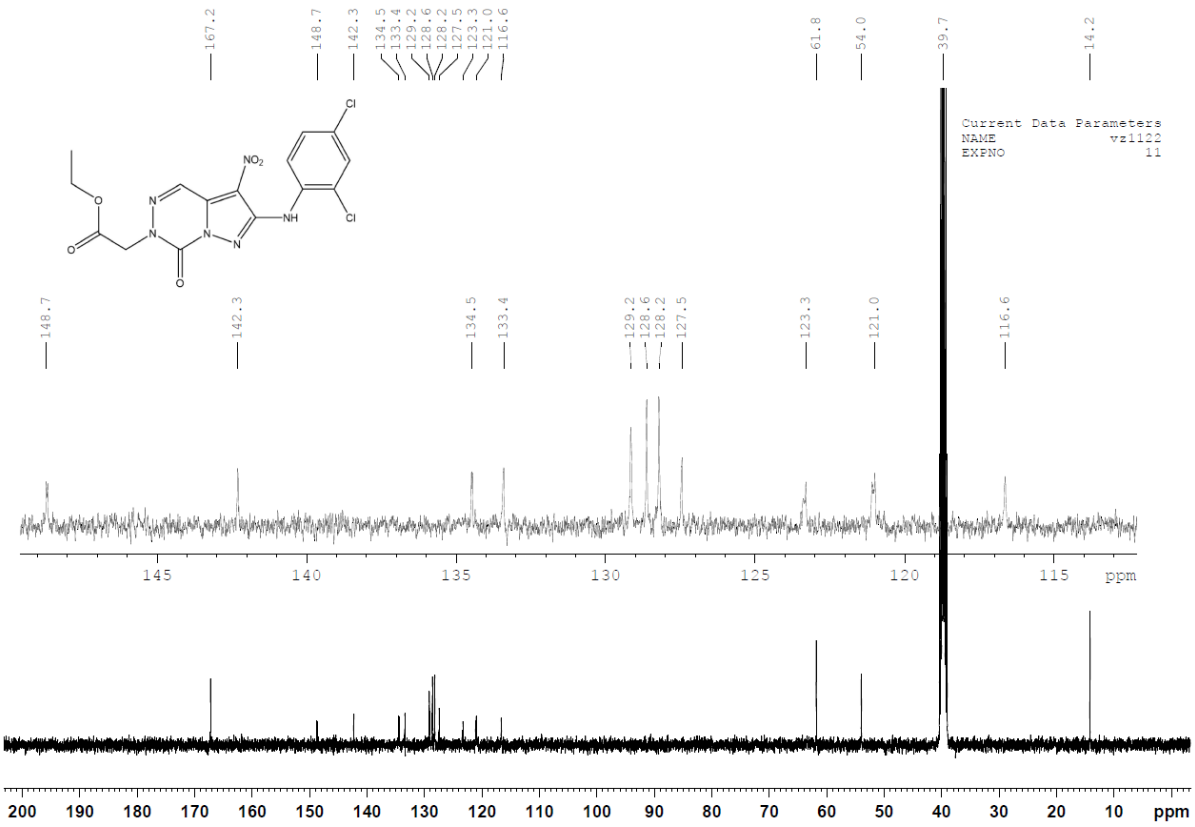

Figure S103-S104. ^{1}H and ^{13}C NMR spectra of compound **7i**

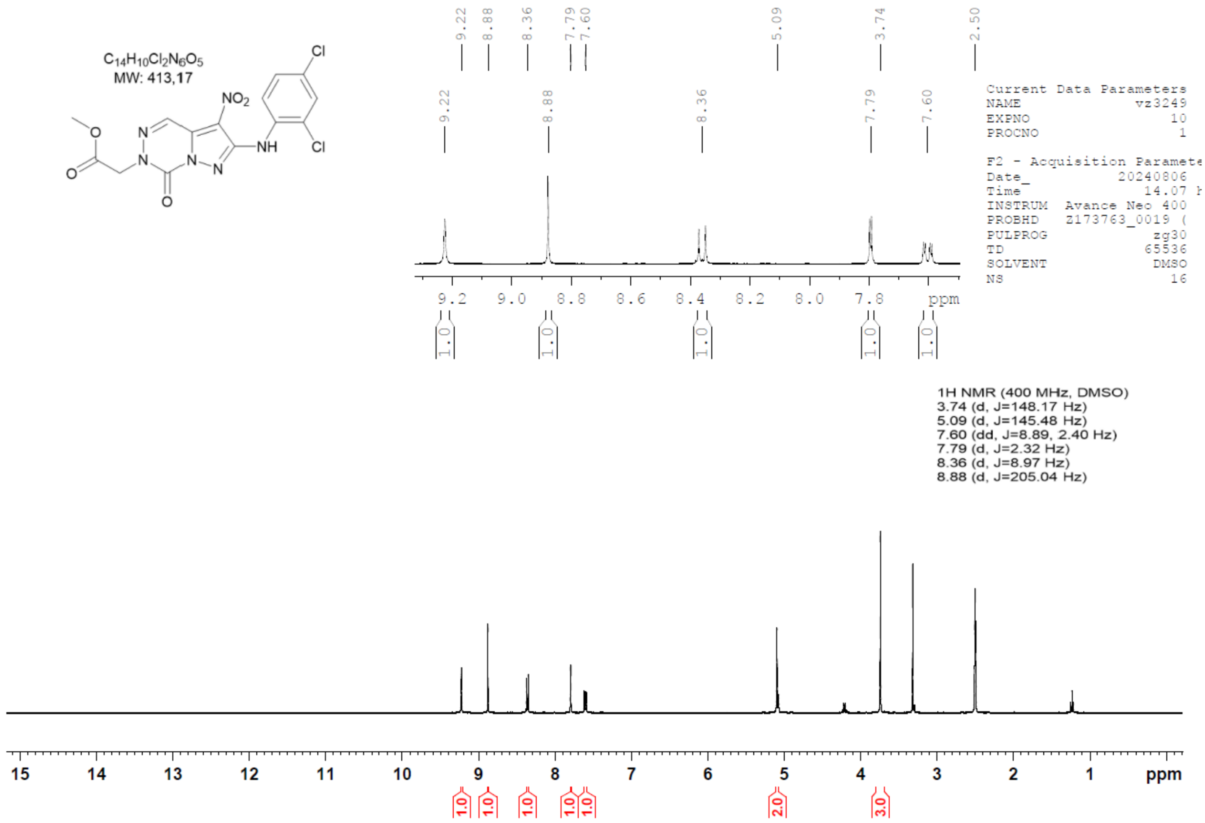

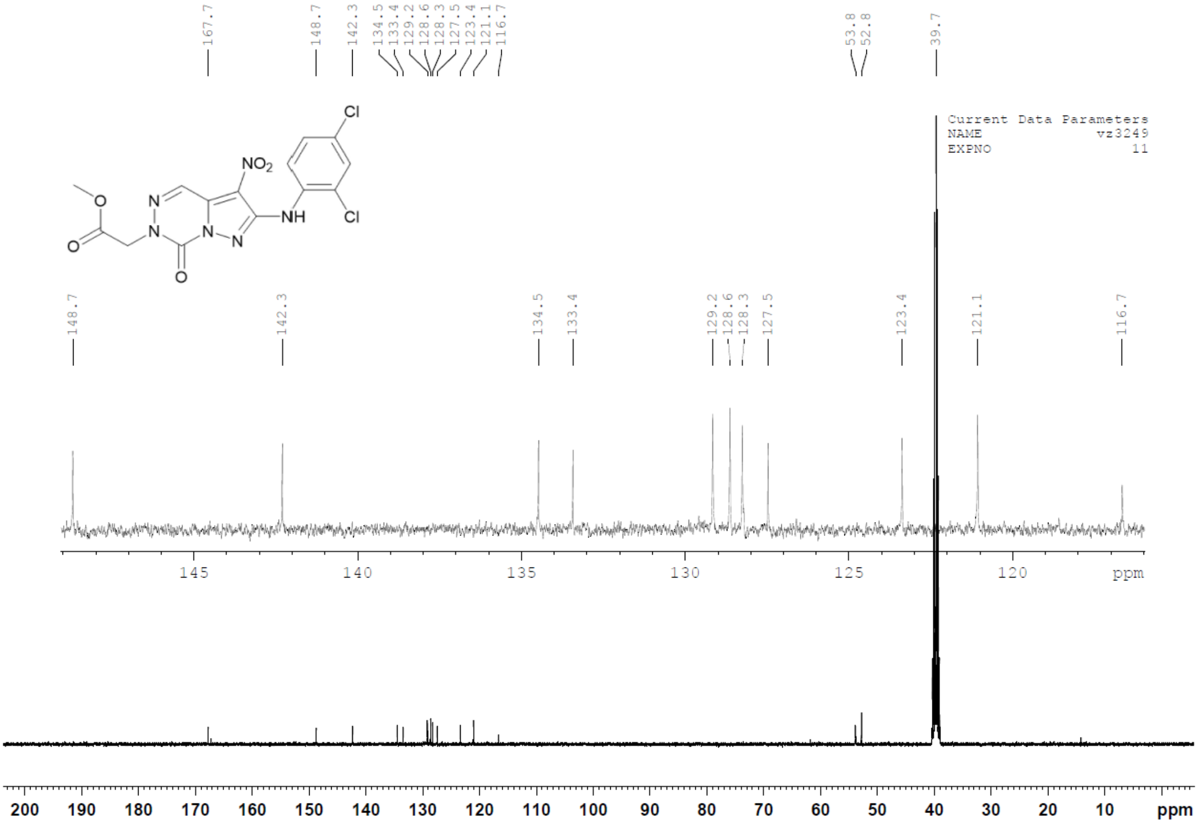

Figure S105-S106. ^{1}H and ^{13}C NMR spectra of compound **7ii**

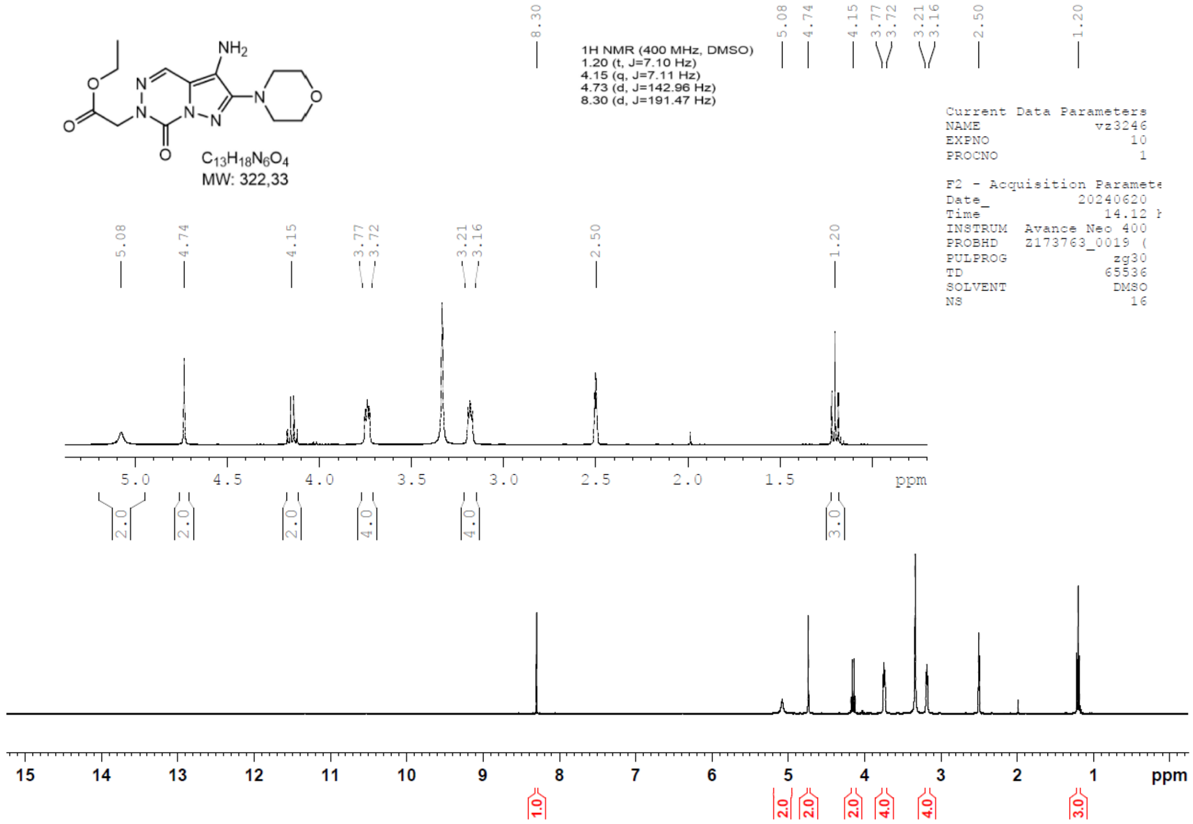

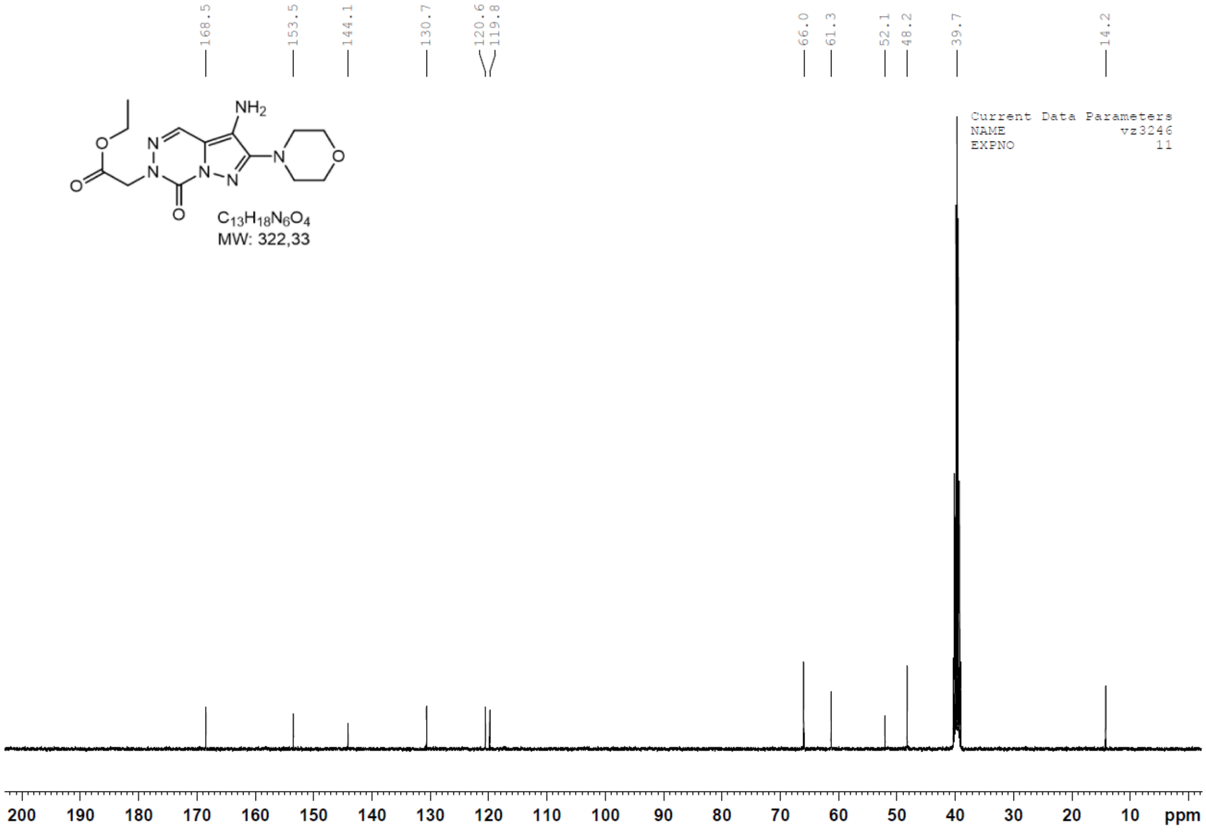

Figure S107-S108. ^{1}H and ^{13}C NMR spectra of compound **8b**

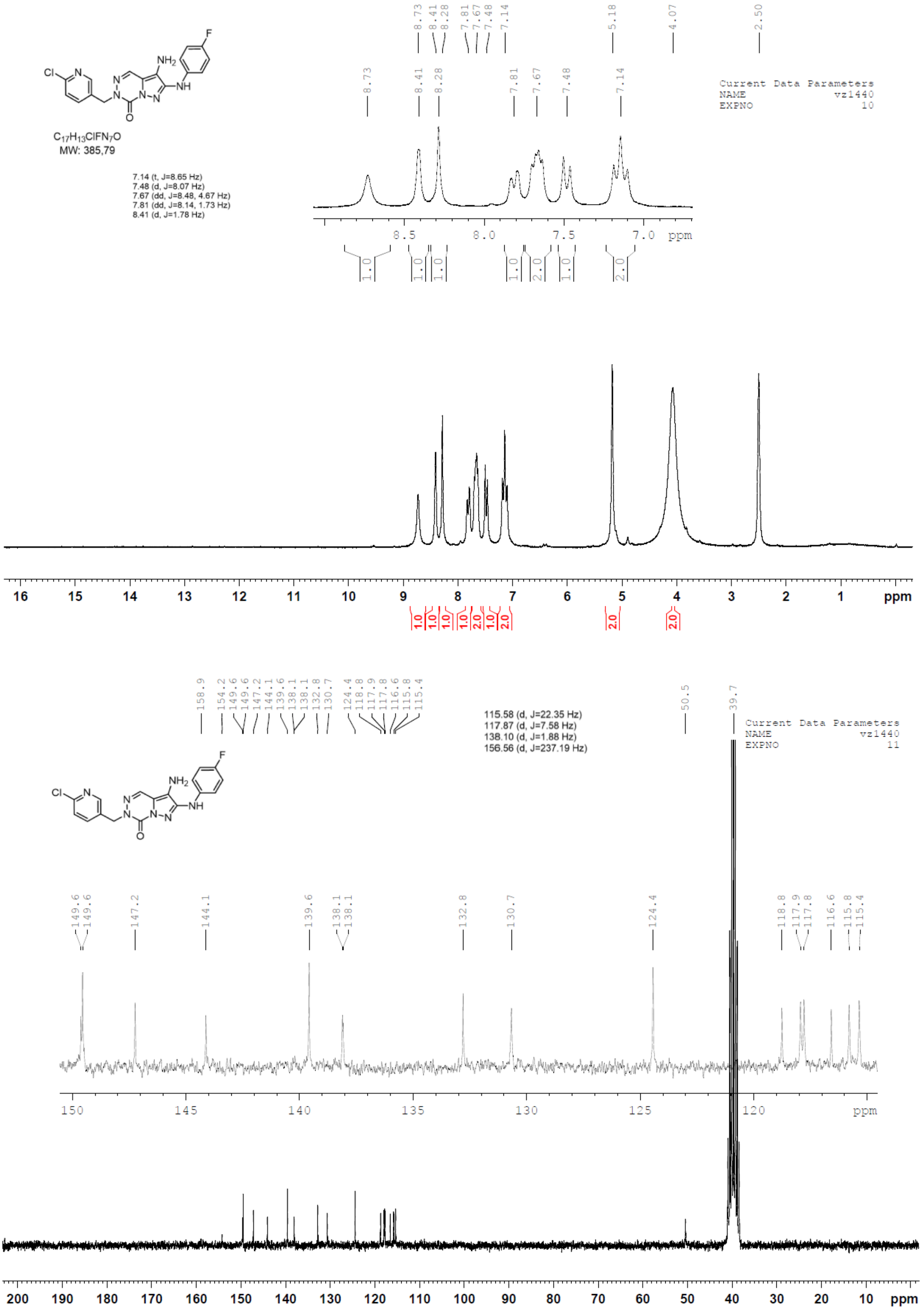

Figure S109-S110. ^{1}H and ^{13}C NMR spectra of compound **8f**

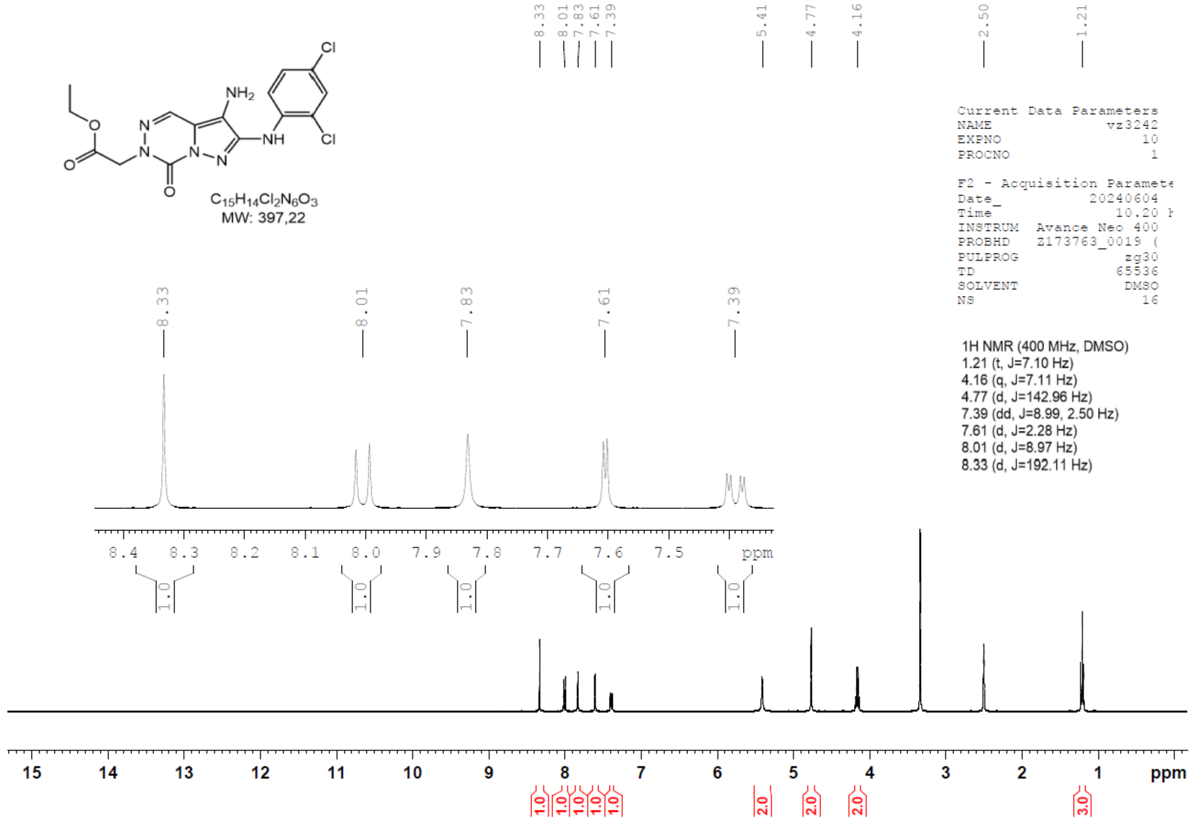

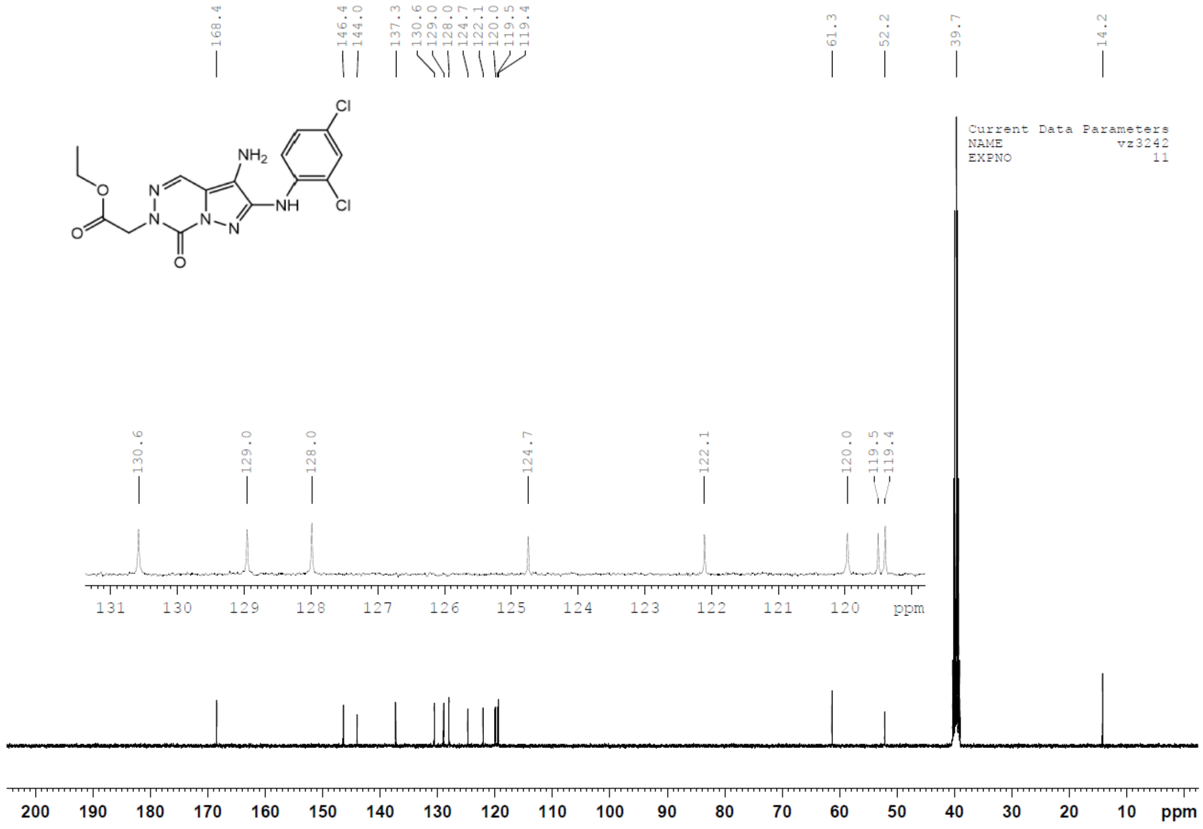

Figure S111-S112. ^{1}H and ^{13}C NMR spectra of compound **8i**

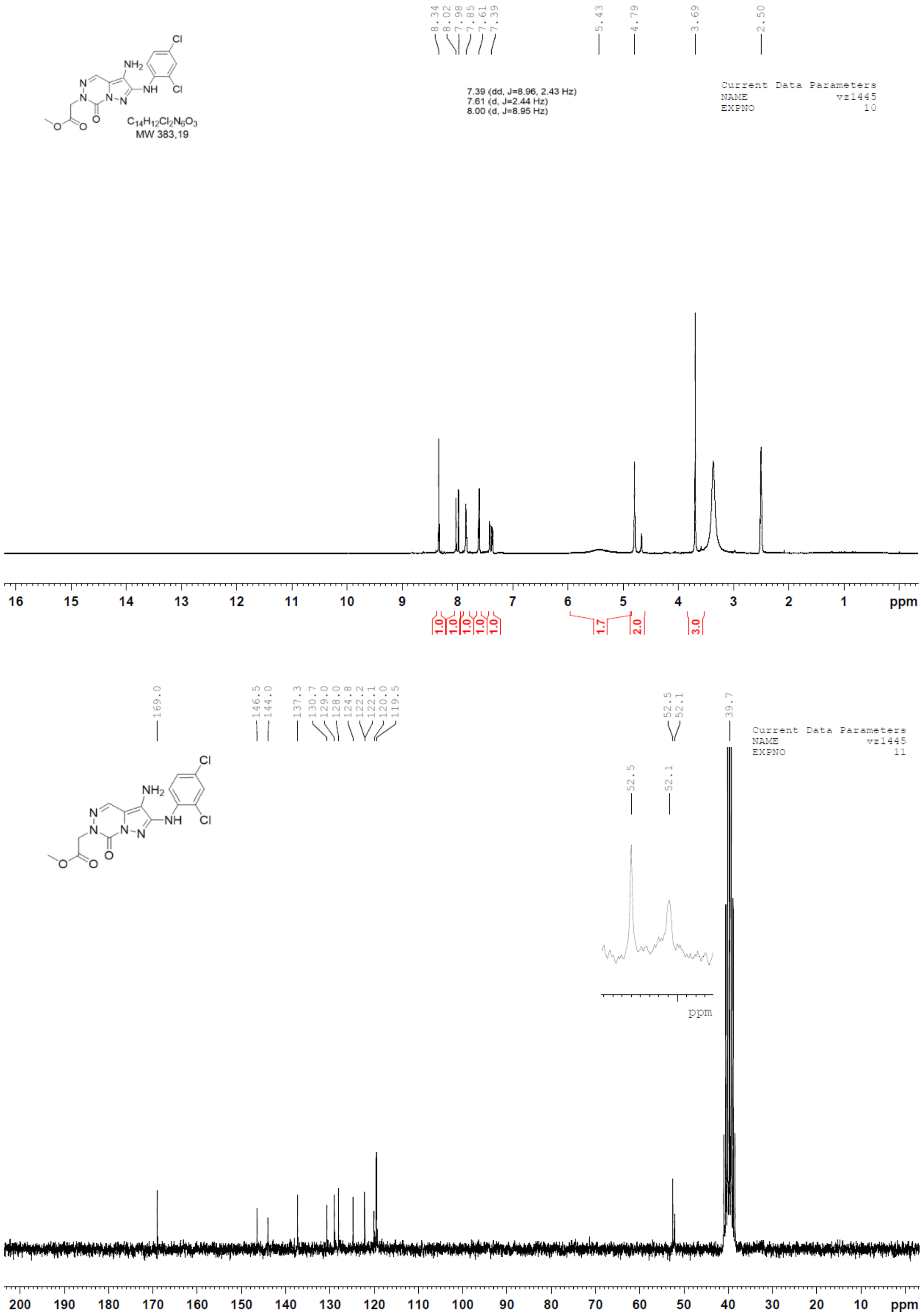

Figure S113-S114. ^{1}H and ^{13}C NMR spectra of compound **8ii**

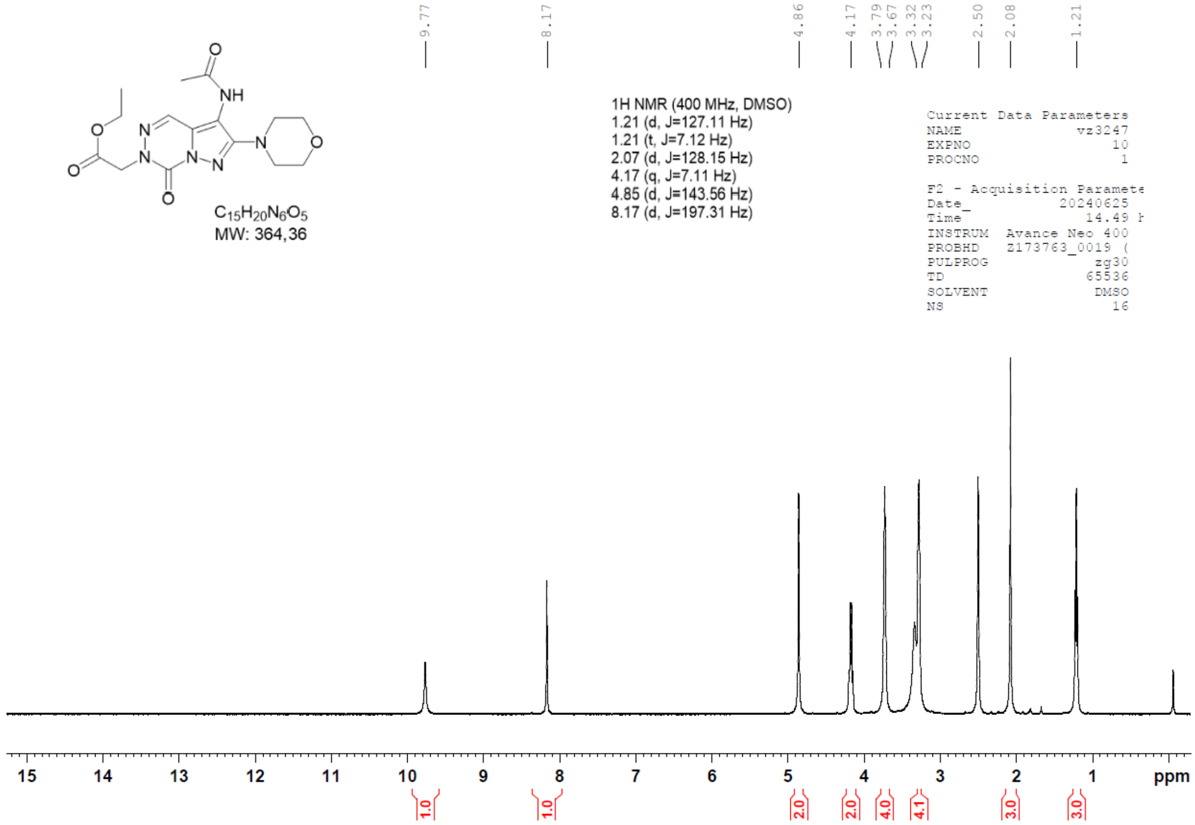

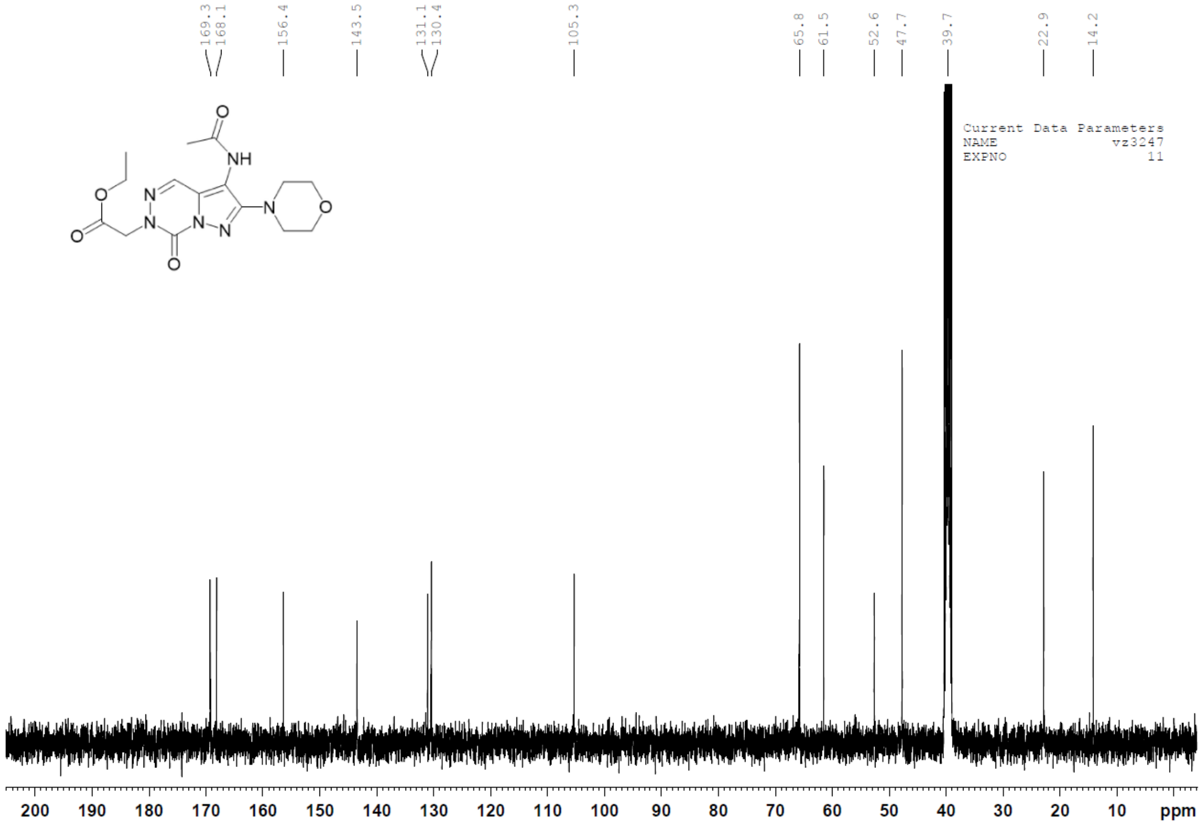

Figure S115-S116. ^{1}H and ^{13}C NMR spectra of compound **9b**

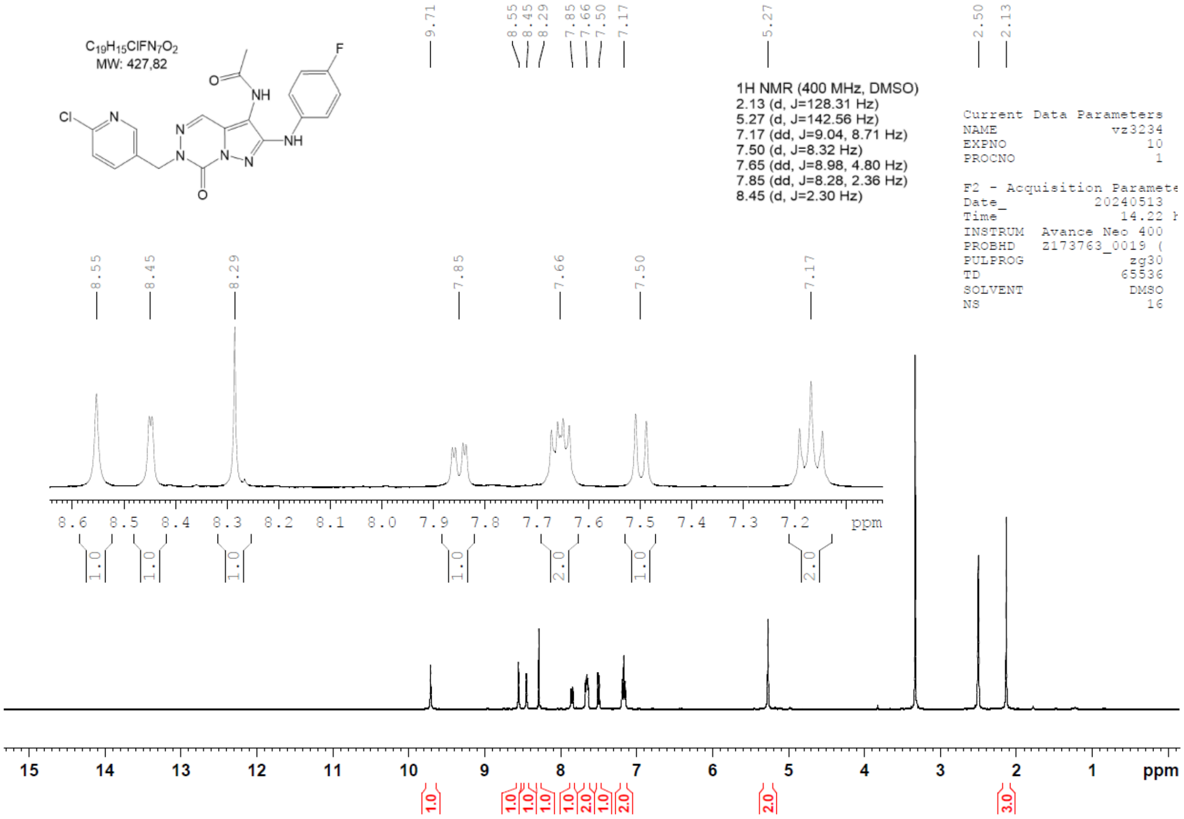

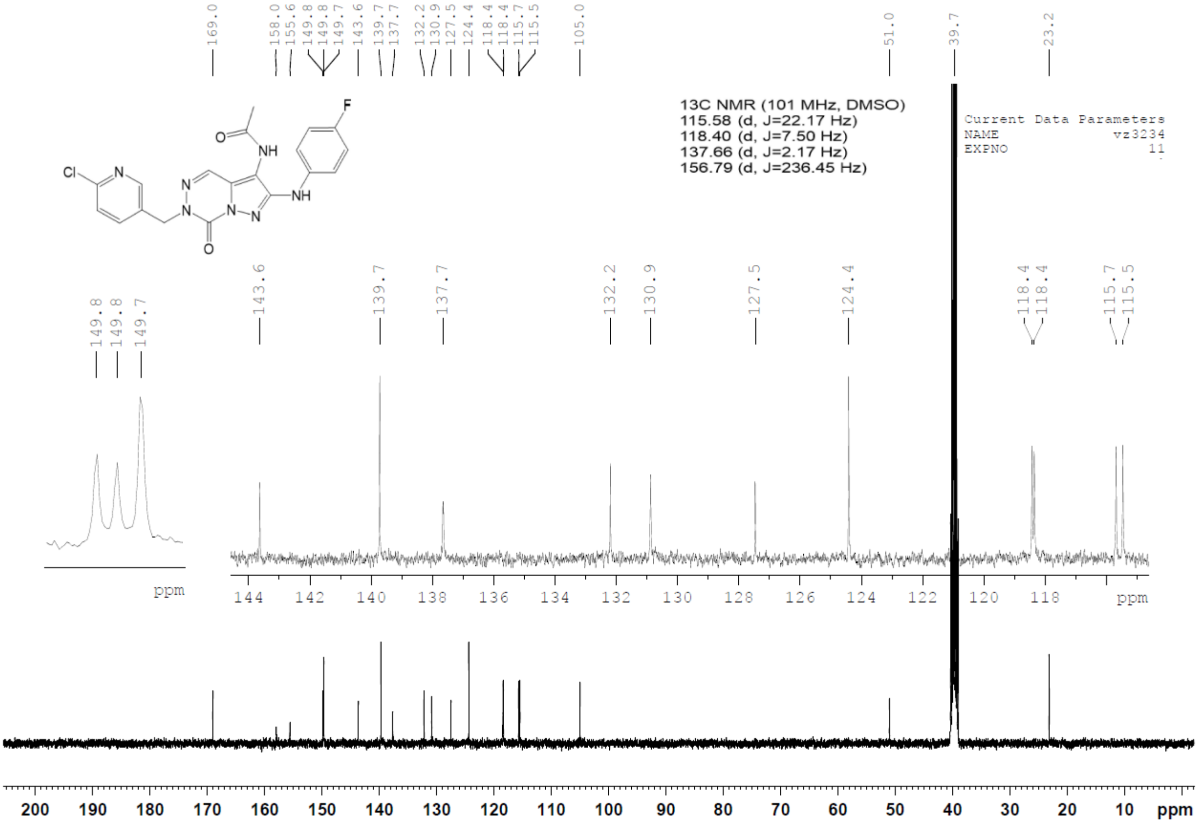

Figure S117-S118. ^{1}H and ^{13}C NMR spectra of compound **9f**

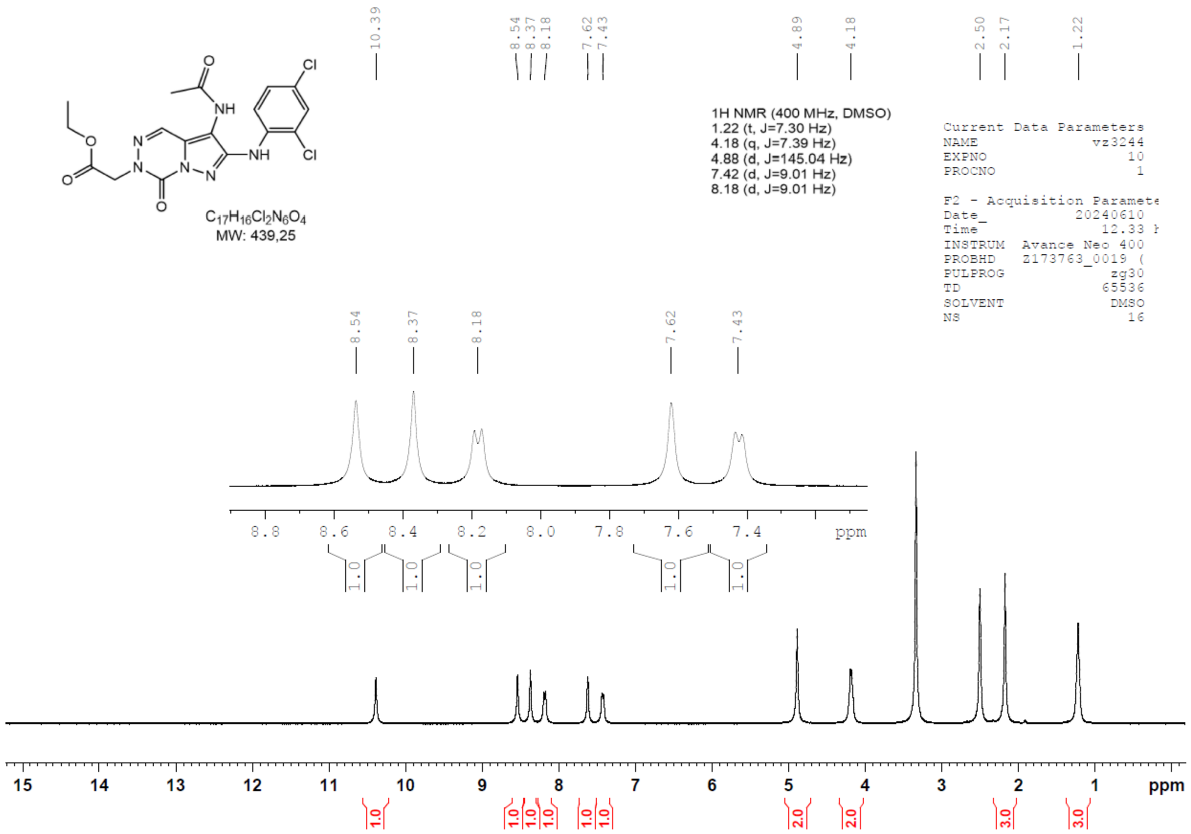

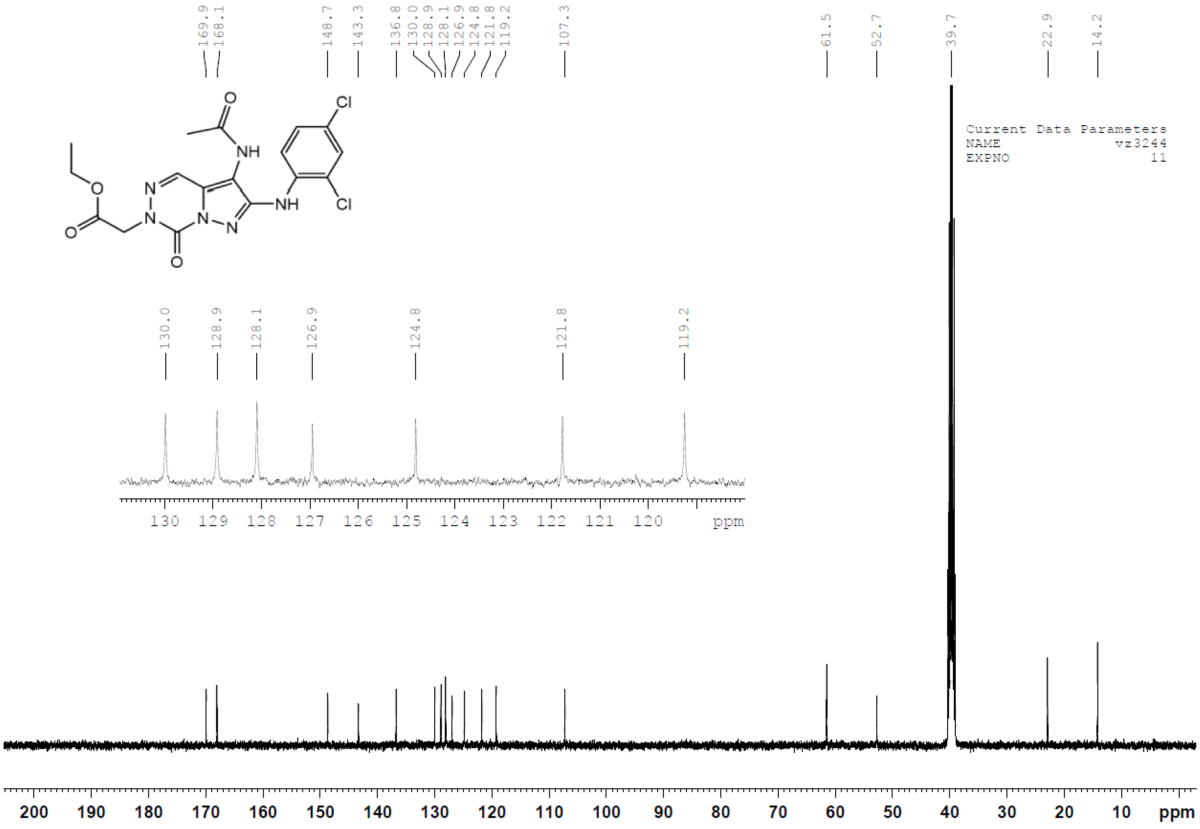

Figure S119-S120. ^{1}H and ^{13}C NMR spectra of compound **9i**

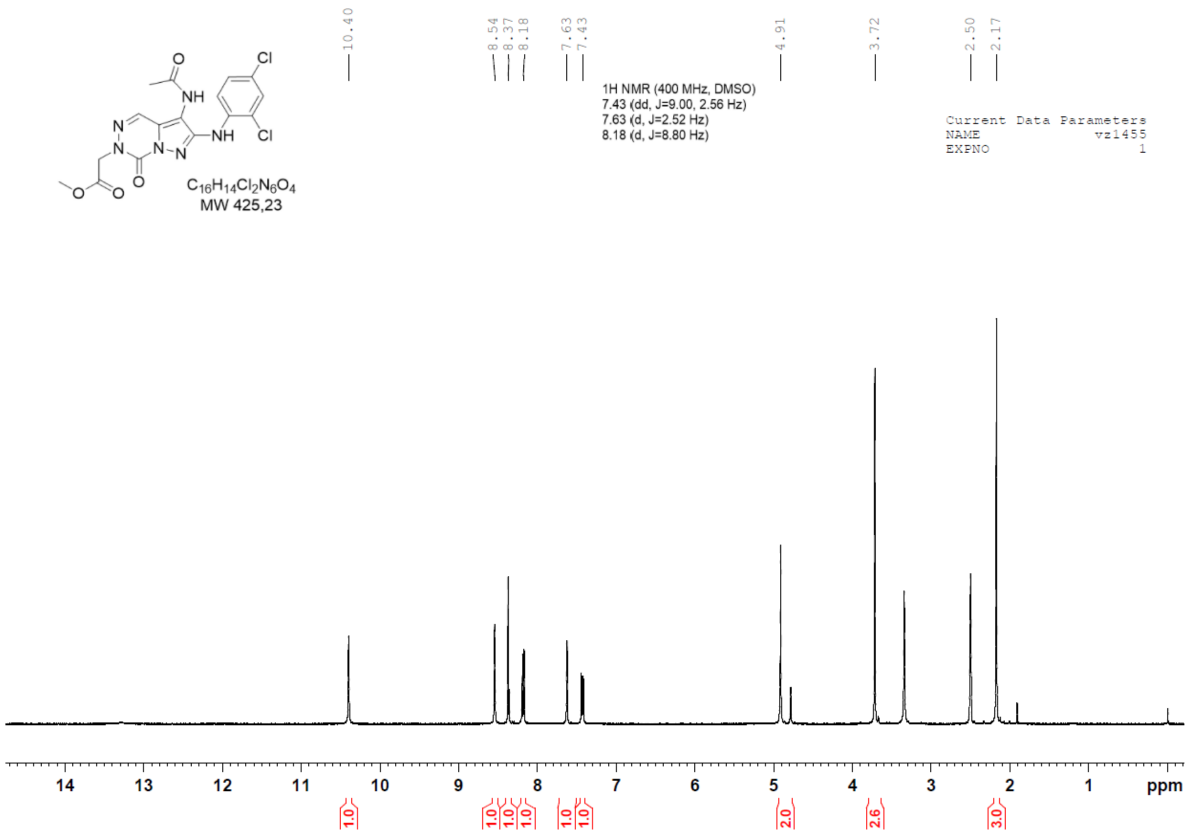

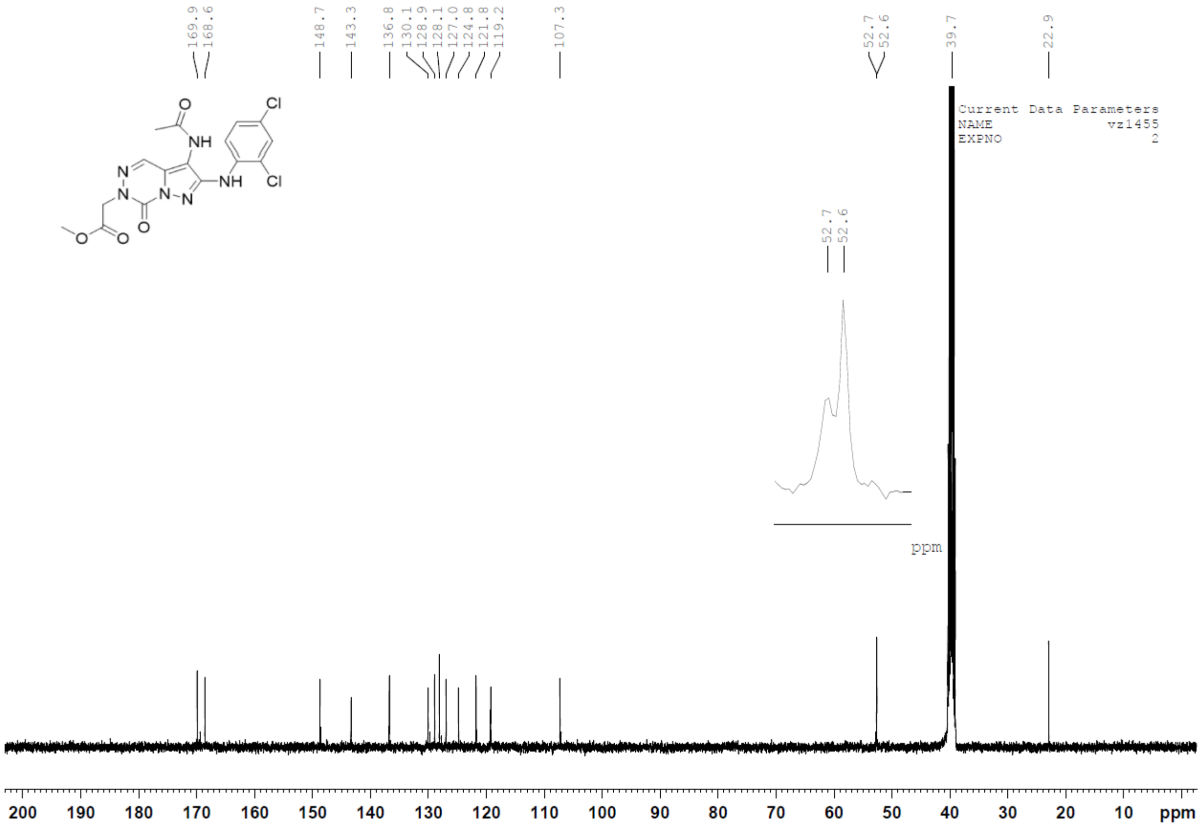

Figure S121-S122. ¹H and ¹³C NMR spectra of compound **9ii**

Soll=281,9574(M+Na+) **Generic Display Report**

Analysis Info

Analysis Name	D:\Data\Messungen 2025\Zapolski Viktor\VZ1029-A.d	Acquisition Date	5/9/2025 10:59:13 AM
Method	ESI-pos-klein_Naformiat.m	Operator	Demo User
Sample Name		Instrument	impact II
Comment			

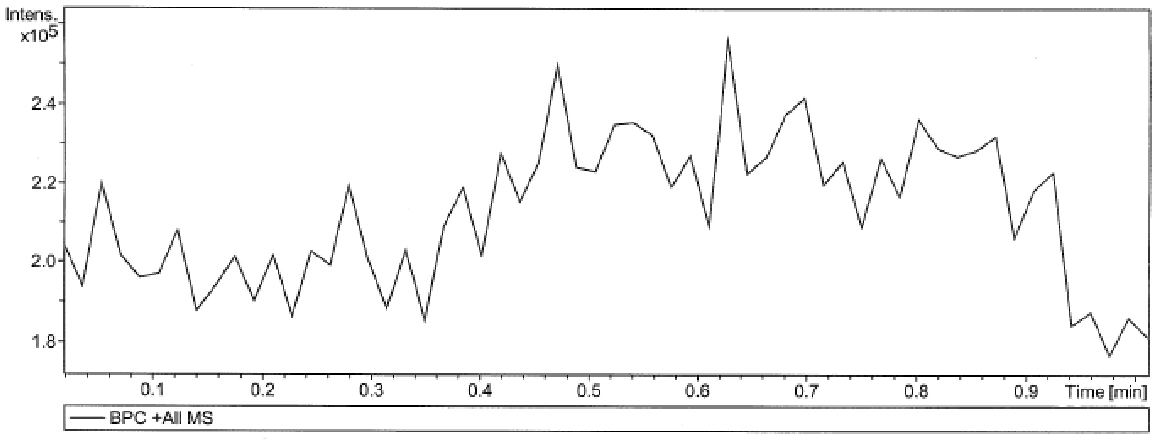

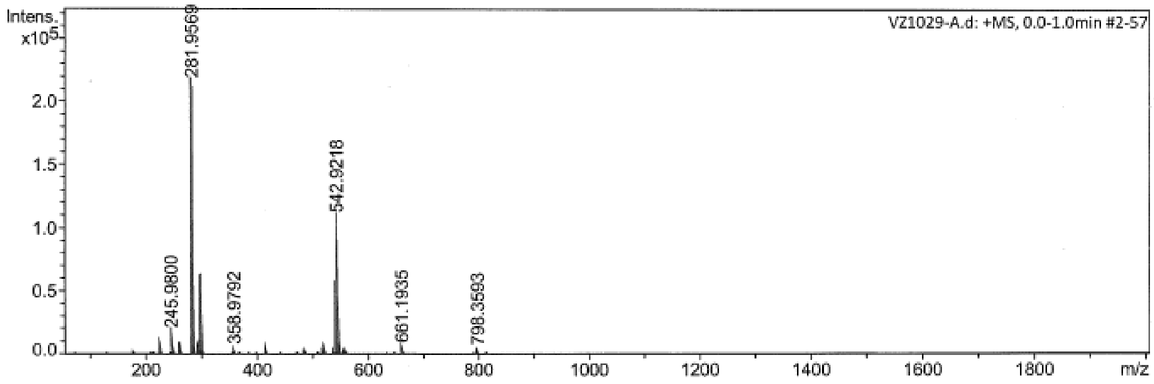

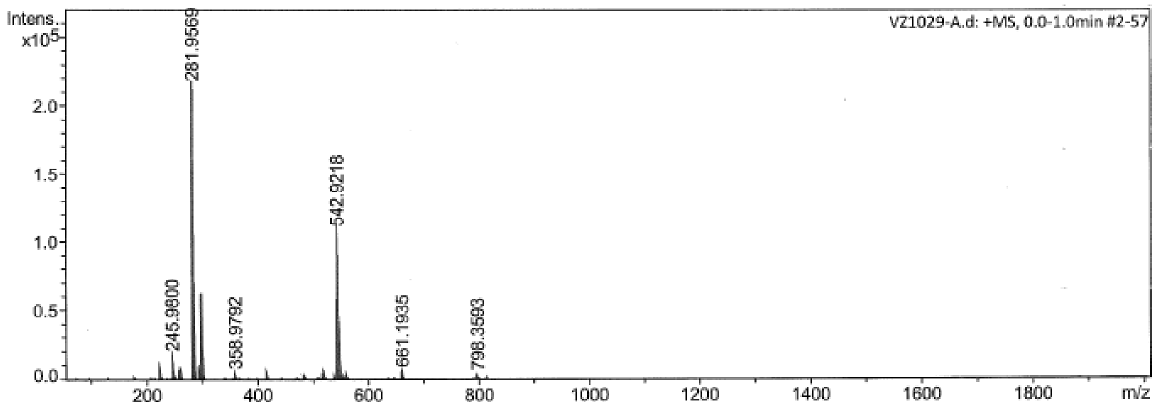

Bruker Compass DataAnalysis 4.4 printed: 5/9/2025 11:00:40 AM by: demo Page 1 of 1

Figure S123. HRMS spectrum of **2a**

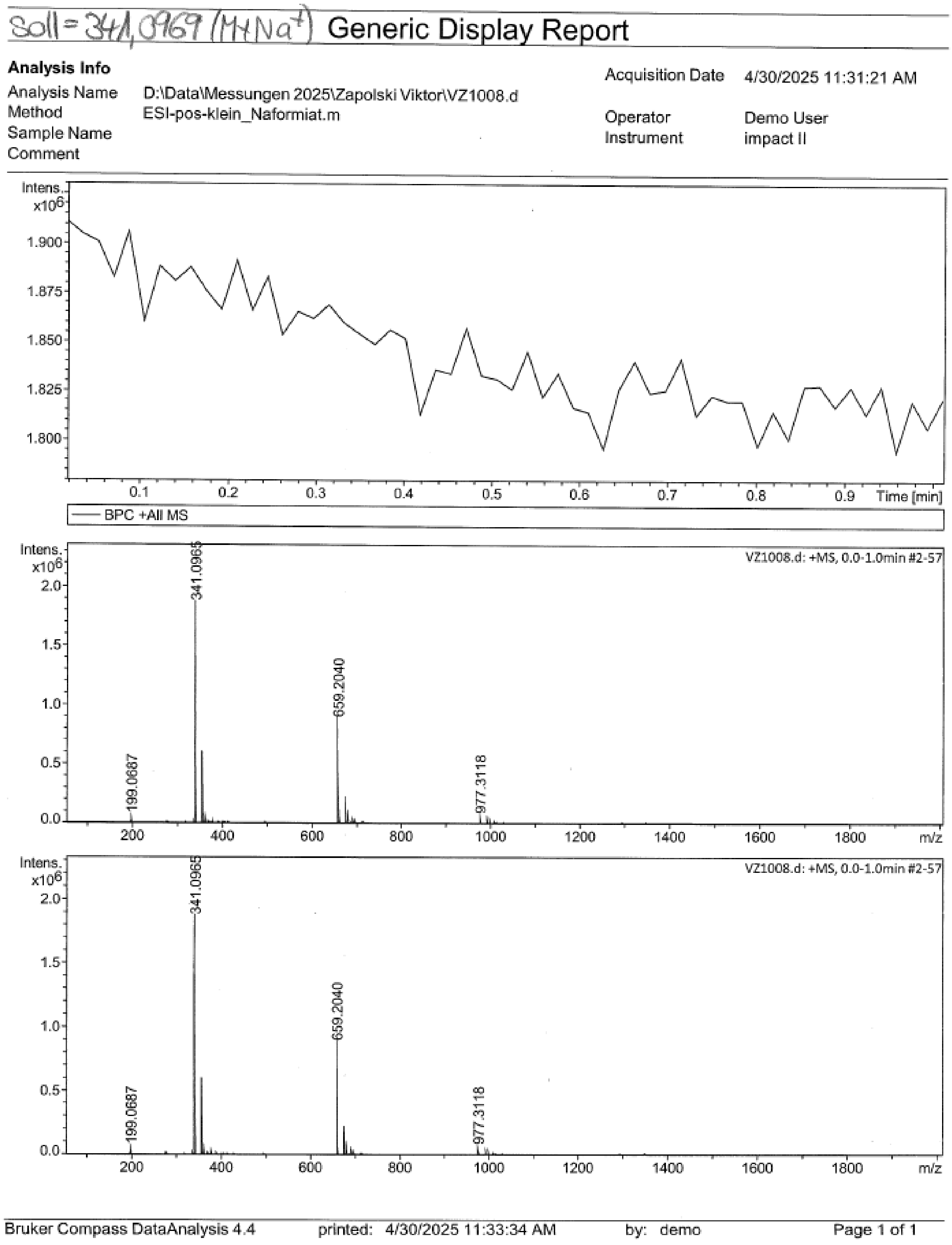

Figure S124. HRMS spectrum of **3ee**

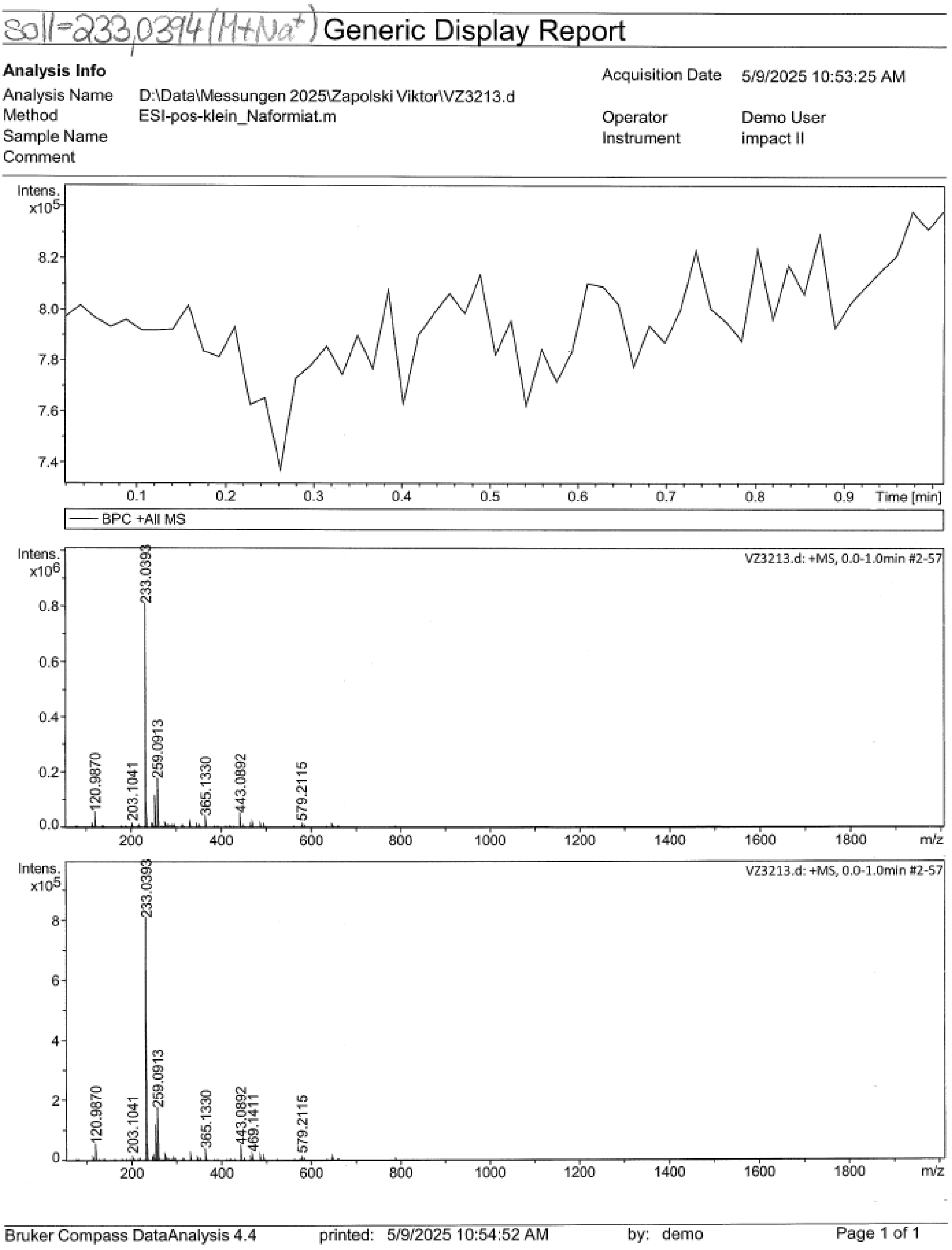

Figure S125. HRMS spectrum of **4a**

Soll = 259,0914 ($M+Na^+$) **Generic Display Report**

Analysis Info

Analysis Name	D:\Data\Messungen 2025\Zapolski Viktor\VZ3210.d	Acquisition Date	5/9/2025 10:47:29 AM
Method	ESI-pos-klein_Naformiat.m		
Sample Name		Operator	Demo User
Comment		Instrument	impact II

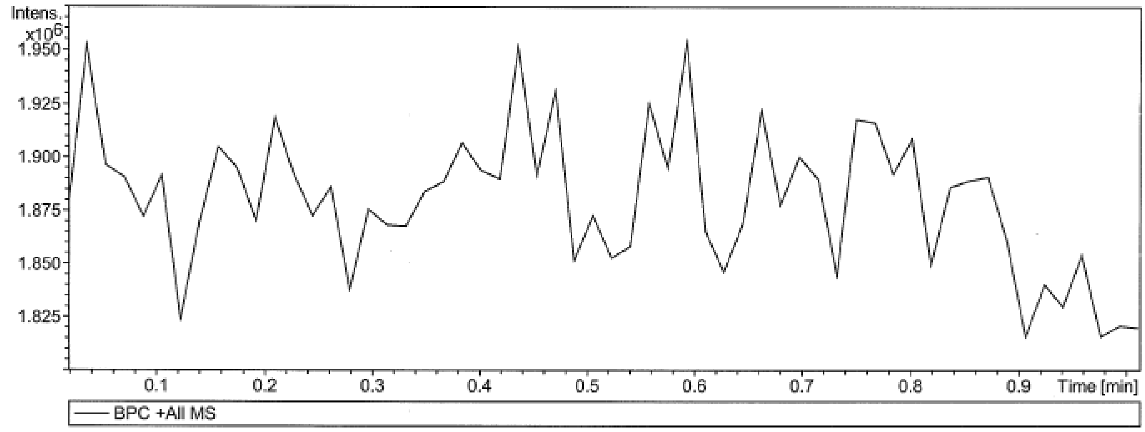

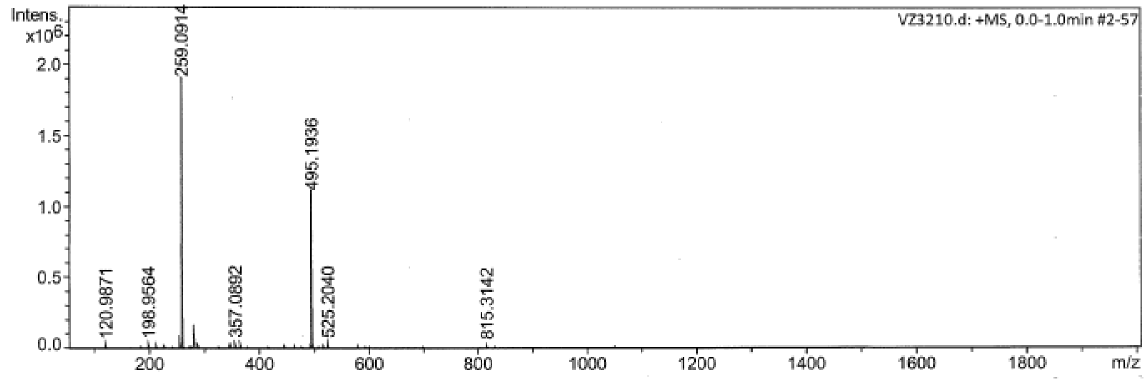

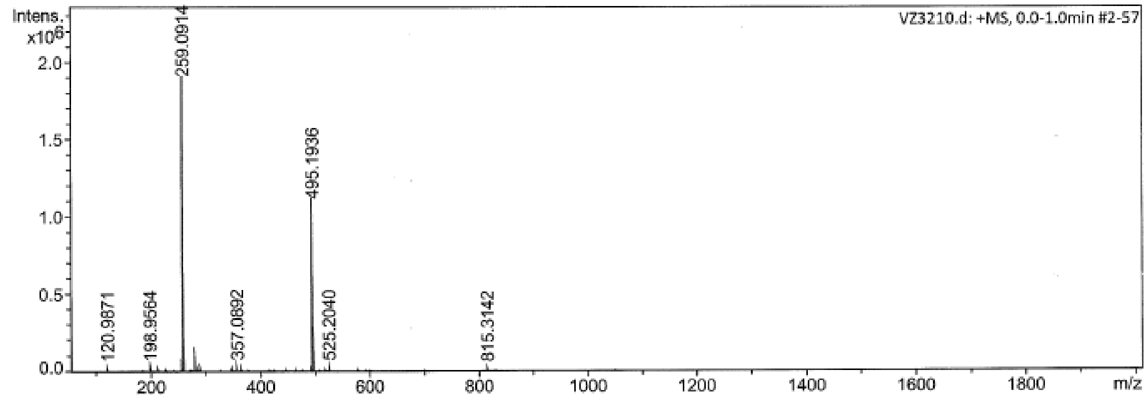

Bruker Compass DataAnalysis 4.4 printed: 5/9/2025 10:49:25 AM by: demo Page 1 of 1

Figure S126. HRMS spectrum of **5b**

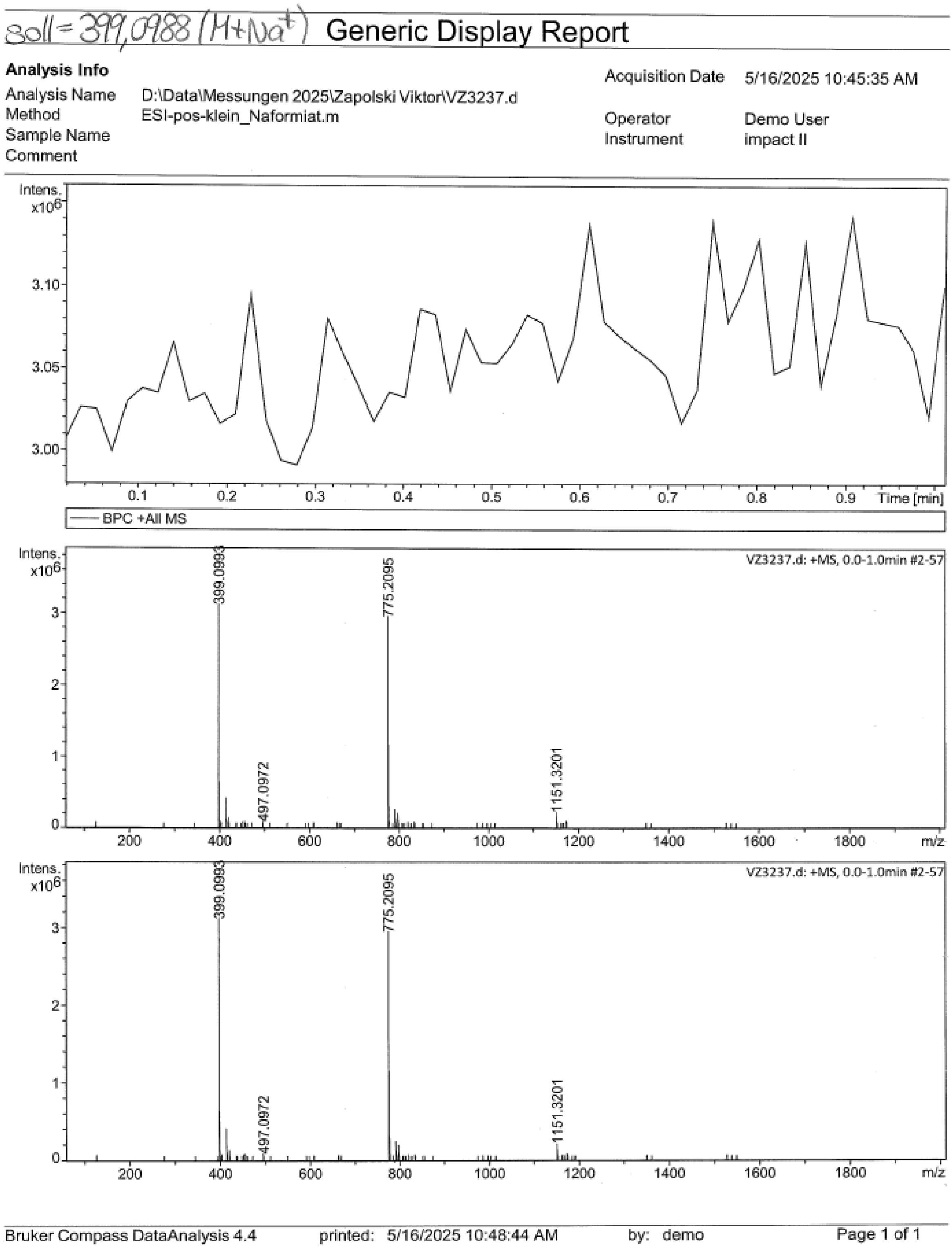

Figure S127. HRMS spectrum of **6dd**

Soll=307,0914 (M+Na+) Generic Display Report

Analysis Info

Analysis Name	D:\Data\Messungen 2025\Zapolski Viktor\VZ3211.d	Acquisition Date	5/16/2025 10:35:44 AM
Method	ESI-pos-klein_Naformiat.m	Operator	Demo User
Sample Name		Instrument	impact II
Comment			

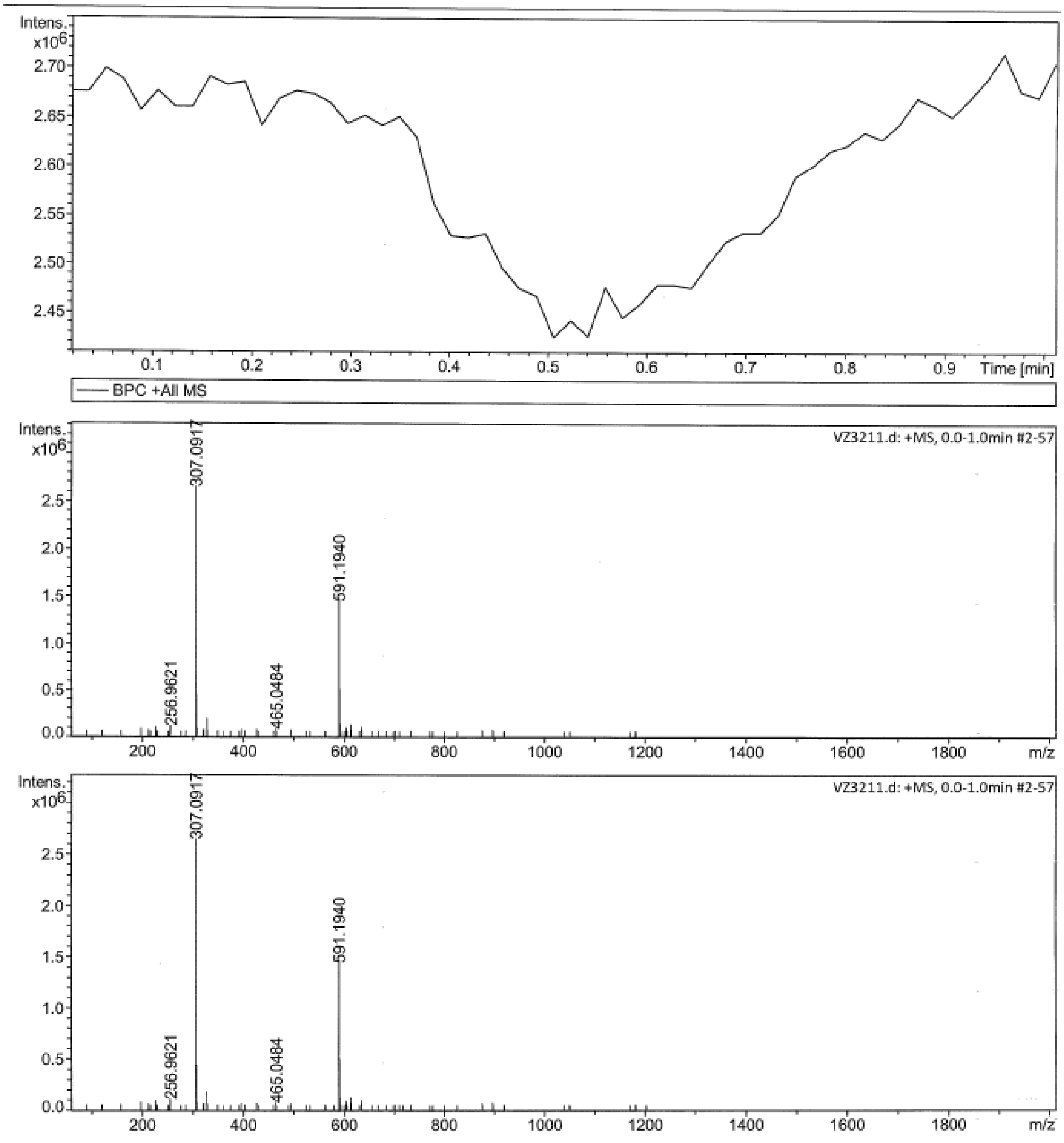

Figure S128. HRMS spectrum of **6e**

Sal(=438,0488(M+Na+) Generic Display Report

Analysis Info

Analysis Name	D:\Data\Messungen 2025\Zapolski Viktor\VZ1435.d	Acquisition Date	5/16/2025 10:40:50 AM
Method	ESI-pos-klein_Naformiat.m	Operator	Demo User
Sample Name		Instrument	impact II
Comment			

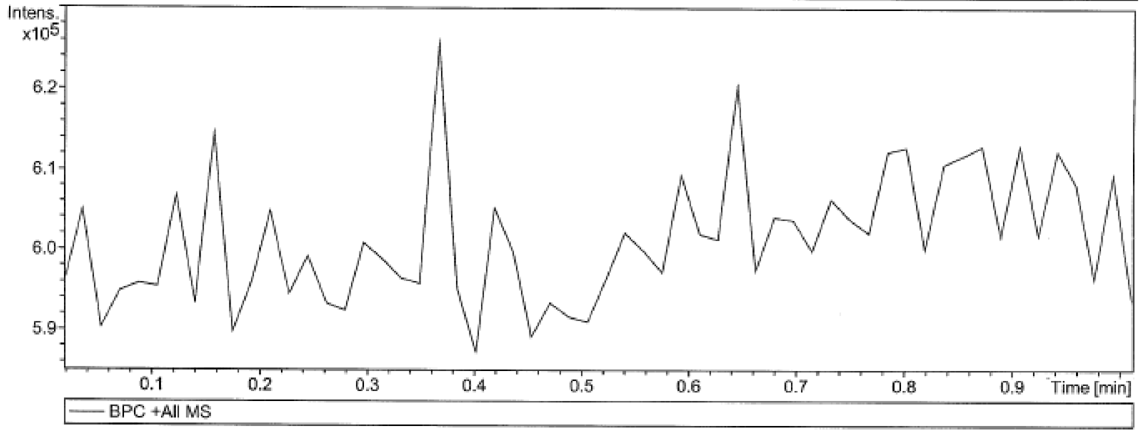

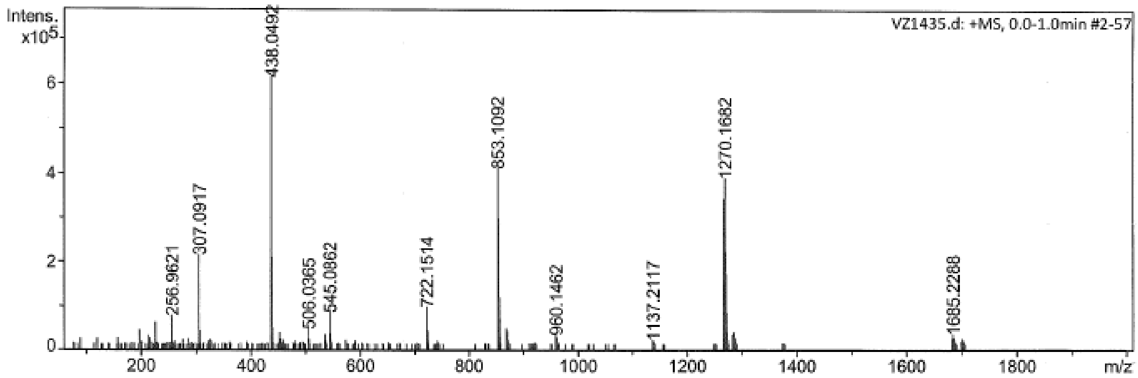

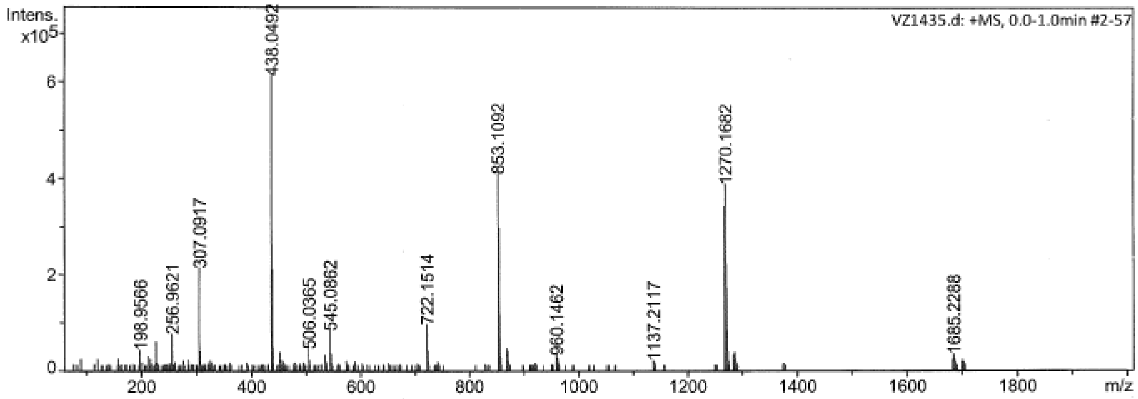

Bruker Compass DataAnalysis 4.4 printed: 5/16/2025 10:42:14 AM by: demo Page 1 of 1

Figure S129. HRMS spectrum of **7f**

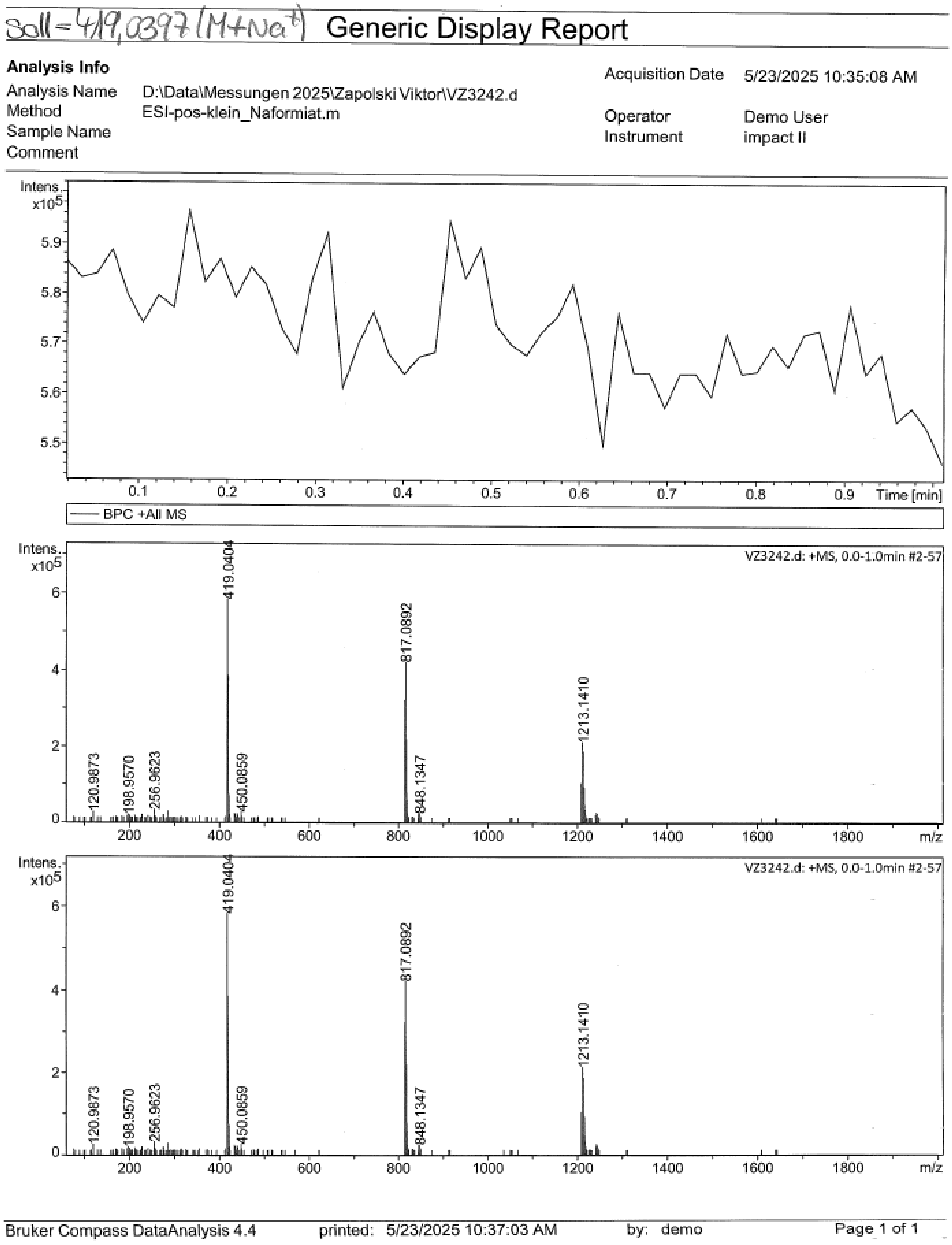

Figure S130. HRMS spectrum of **8i**

soll=450,0852 (M+Na+) Generic Display Report

Analysis Info

Analysis Name	D:\Data\Messungen 2025\Zapolski Viktor\VZ3234.d	Acquisition Date	5/23/2025 10:29:25 AM
Method	ESI-pos-klein_Naformiat.m	Operator	Demo User
Sample Name		Instrument	impact II
Comment			

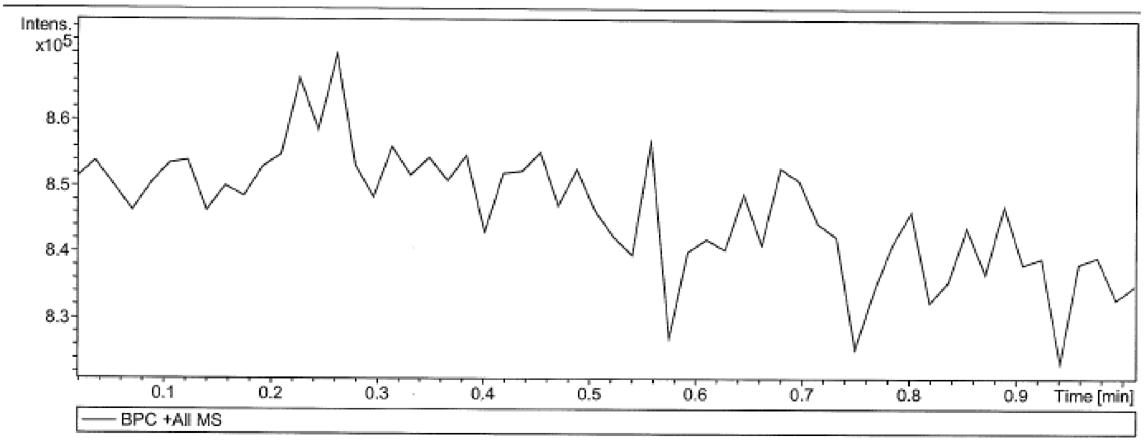

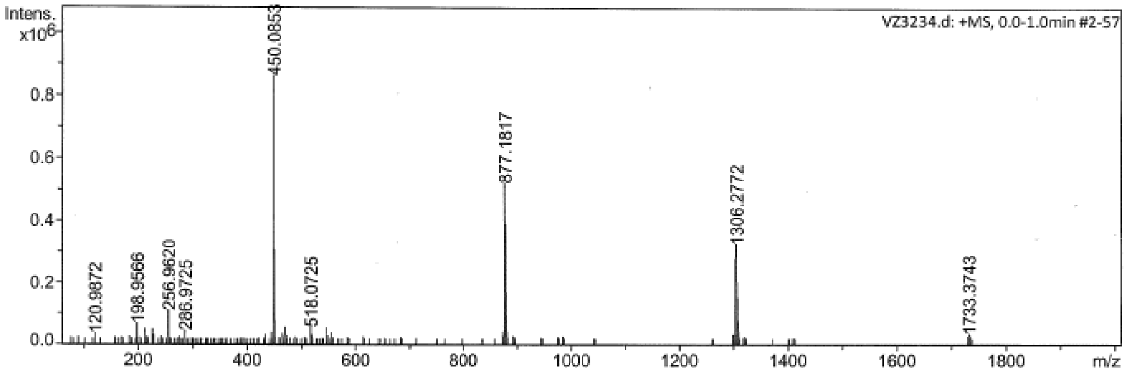

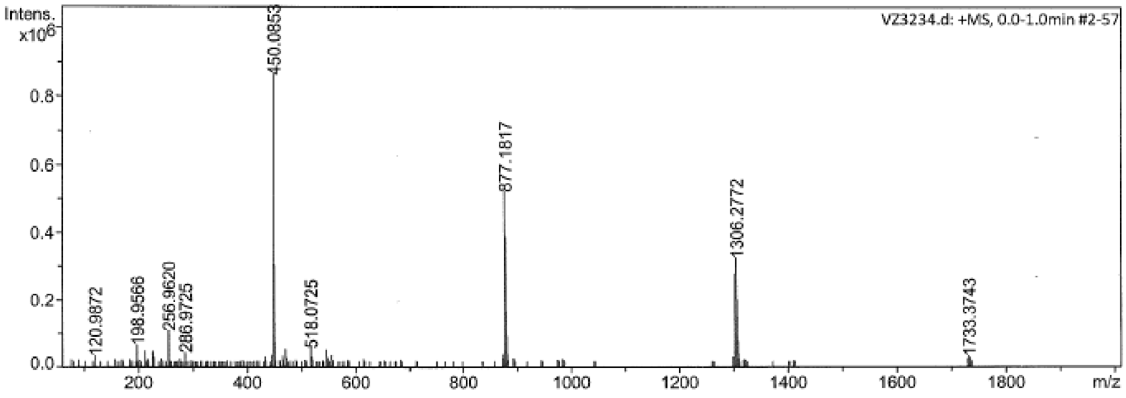

Bruker Compass DataAnalysis 4.4 printed: 5/23/2025 10:33:27 AM by: demo Page 1 of 1

Figure S131. HRMS spectrum of **9f**

Compound-ID	*E. coli* Growth [%]	*E. coli ΔTolC* Mean Growth* [%]	*E. coli ΔTolC* IC50 [µM]	*Klebsiella pneumoniae* Growth [%]	*S. aureus* Mean Growth*[%]	*S. aureus* IC50 [µM]	*L929* Mean Viability* [%]	L929 Viability IC50 [µM]
**2a**	> 100	103.78		> 90	102.71		99.97	
**2b**	> 100	102.12		> 90	102.09		70.92	
**2c**	> 100	32.46	11.44	> 90	26.43	7.7	1.07	7.06
**2d**	> 100	38.45	33.33	> 90	103.38	>50	1.61	13.2
**2e**	> 100	0.48	19.42	> 90	86.19	>50	1.05	17.6
**2i**	> 100	4.45	6.68	> 90	5.56	8.33	2.27	15.7
**2j**	> 100	55.95	27.08	> 90	2.26	3.56	1.11	11.8
**3a**	> 100	104.43		> 90	92.48		120.04	
**3b**	> 100	102.69		> 90	100.67		95	
**3c**	> 100	105.69		> 90	125.82		94.51	
**3d**	> 100	101.44		> 90	120.92		69.23	
**3e**	> 100	116.85		> 90	119.21		120.59	
**3ee**	> 100	103.77		> 90	105.71		105.33	
**3ff**	> 100	109.48		> 90	120.6		119.83	
**3f**	> 100	105.33		> 90	109.42		105.57	
**3gg**	> 100	103.82		> 90	118.25		104.17	
**3i**	> 100	103.47		> 90	113.65		107.49	
**3j**	> 100	103.02		> 90	115.19		101.75	
**4a**	> 100	105.12		> 90	79.99		121.18	
**4b**	> 100	94.08		> 90	81.12		121.51	
**4c**	> 100	78.64		> 90	86.02		109.36	
**4d**	> 100	96.71		> 90	90.58		122.43	
**4e**	> 100	88.25		> 90	76.19		96.33	
**4f**	> 100	54.06	>50	> 90	70.13	>50	99.21	36.5
**4g**	> 100	1.31	39.3	> 90	22.73		93.93	25
**4h**	> 100	10.25	>50	> 90	58.46	>50	58.52	32.2
**4i**	> 100	103.37		> 90	113.78		109.61	
**4j**	> 100	36.64	22.76	> 90	87.33	41.6	124.19	35.1
**7b**	> 100	94.02	>50	> 90	87.17	>50	6.61	11
**7f**	> 100	100.63		> 90	122.07		111.74	
**7i**	> 100	110.66		> 90	137.59		105.97	
**7ii**	> 100	104.73		> 90	118.03		97.47	
**5a**	> 100	104.15		> 90	94.16		116.95	
**5b**	> 100	101.77		> 90	97.67		106.19	
**5c**	> 100	95.24		> 90	99.33		86.86	
**5d**	> 100	90.03		> 90	93.45		109.47	
**5e**	> 100	105.44		> 90	96.13		91.58	
**5f**	> 100	99.45		> 90	101.67		111.52	
**5g**	> 100	108.89		> 90	124.13		92.87	
**5h**	> 100	100.56		> 90	107.32		95.28	
**5i**	> 100	88.95		> 90	113.77		67.67	
**5j**	> 100	78.88		> 90	115.82		90.94	
**8b**	> 100	104.38		> 90	96.6		117.03	
**8f**	> 100	96.8		> 90	146.74		102.16	
**8ii**	> 100	109,58		> 90	129.39		101.34	
**8i**	> 100	109.64		> 90	114.35		118.88	
**6a**	> 100	103.99		> 90	95.25		126.61	
**6b**	> 100	104.72		> 90	94.7		113.15	
**6c**	> 100	102.77		> 90	144,52		116.07	
**6d**	> 100	103.83		> 90	97.2		105.8	
**6dd**	> 100	102.05		> 90	97.34		109.52	
**6e**	> 100	101.84		> 90	94,79		119.89	
**6f**	> 100	101.11		> 90	105.86		112.92	
**6g**	> 100	100.43		> 90	127.07		67.81	

**6h**	> 100	104.39	> 90	101	115.83
**6i**	> 100	103.11	> 90	122.01	94.23
**6j**	> 100	106.71	> 90	102.53	113.02
**9b**	> 100	103.72	> 90	98.68	109.81
**9f**	> 100	106.46	> 90	122.76	121.34
**9ii**	> 100	107.94	> 90	131.45	113.07
**9i**	> 100	114.91	> 90	139.85	120.28

* Mean values are the average of 3 replicates, which were on 3 different microtiter plates.

Dose-response curves for the calculation of IC_{50}-values were generated for those compounds, for which growth of at least one bacterial strain or viability of the L929 cell line was less than 50%. The IC_{50}-values were calculated by nonlinear regression using the software GraphPad Prism.

Table S1: Primary screening data of all tested compounds and IC_{50}-values of those compounds, which showed a growth inhibitory effect > 50% in the primary screening
